# Operationalization of Artificial Intelligence Applications in the Intensive Care Unit: A Systematic Review

**DOI:** 10.1001/jamanetworkopen.2025.22866

**Published:** 2025-07-23

**Authors:** Willemijn E. M. Berkhout, Julia J. van Wijngaarden, Jessica D. Workum, Davy van de Sande, Denise E. Hilling, Christian Jung, Geert Meyfroidt, Diederik Gommers, Stefan N. R. Buijsman, Michel E. van Genderen

**Affiliations:** 1 Department of Adult Intensive Care, Erasmus MC University Medical Center, Rotterdam, the Netherlands; 2 Erasmus MC Datahub, University Center Rotterdam, Rotterdam, the Netherlands; 3 Department of Biomedical Engineering, Eindhoven University of Technology, Eindhoven, the Netherlands; 4 Department of Intensive Care, Elisabeth-TweeSteden Hospital, Tilburg, the Netherlands; 5 Department of Surgical Oncology and Gastrointestinal Surgery, Erasmus MC Cancer Center, University Medical Center Rotterdam, Rotterdam, the Netherlands; 6 Cardiovascular Research Institute Düsseldorf (CARID), Medical Faculty and University Hospital of Düsseldorf, Heinrich-Heine University Düsseldorf, Düsseldorf, Germany; 7 Department of Cardiology, Pulmonology, and Vascular Medicine, Heinrich-Heine-University Düsseldorf, Düsseldorf, Germany; 8 Department of Cellular and Molecular Medicine, Laboratory of Intensive Care Medicine, KU Leuven, Leuven, Belgium; 9 Department of Intensive Care Medicine, University Hospitals Leuven, Leuven, Belgium; 10 Faculty of Technology, Policy, and Management, Delft University of Technology, Delft, the Netherlands

## Abstract

**Importance:**

Artificial intelligence (AI) presents transformative opportunities to address the increasing challenges faced by health care systems globally. Particularly, in data-rich environments, such as intensive care units (ICUs), AI could assist in enhancing clinical decision-making, streamline workflows, and improve patient outcomes. Despite these promising applications, the practical implementation of AI in clinical settings remains limited.

**Objective:**

To systematically evaluate AI system operationalization in the ICU, focusing on the AI field’s progress over time, technical maturity, and risk of bias.

**Evidence Review:**

In this systematic review, 5 databases (Embase, MEDLINE ALL, Web of Science Core Collection, Cochrane Central Register of Controlled Trials, and Google Scholar) were searched for studies published from July 28, 2020, to June 10, 2024. Eligible studies evaluated AI applications designed for use within ICUs for adults (aged ≥16 years) and used data collected during ICU stays. Two reviewers independently screened titles and abstracts, with a third reviewer resolving disagreements. Data extraction included AI application aims, dataset origins, technology readiness level (TRL) categorization, and the use of reporting standards. Risk of bias was assessed using the PROBAST (Prediction Model Study Risk of Bias Assessment Tool).

**Findings:**

Of 17 401 screened records, 1263 studies met the inclusion criteria. A total of 936 studies (74% of all studies) were classified as TRL 4 or below, indicating early-stage development or initial validation. Among these, 447 (37%) used internal datasets, 562 (46%) used MIMIC (Medical Information Mart for Intensive Care) datasets (I-IV), and 78 (6%) used the open-source eICU Collaborative Research Database. External validation (TRL 5) was achieved by 24% of studies. Only 25 (2%) progressed to clinical integration (TRL≥6), with no studies reaching full implementation (TRL 9). Although approximately half of generative AI models reached a higher TRL (14 [47%] with TRL 5), none reached clinical integration. Additionally, only 207 studies (16%) referenced reporting standards, with adherence modestly increasing from 14% in 2021 to 23% in 2024. High risk of bias was identified in 581 of 1103 studies (53%), primarily due to methodologic shortcomings in the analysis domain.

**Conclusions and Relevance:**

Despite substantial growth in AI research within intensive care medicine in recent years, the transition from development to clinical implementation still remains limited and has made little progress over time. A paradigm shift is urgently required in the medical literature—one that moves beyond retrospective validation toward the operationalization and prospective testing of AI for tangible clinical impact.

## Key Points

**Question:**

What is the current state of artificial intelligence (AI) use in intensive care units (ICUs), focusing on AI model maturity and operationalization?

**Findings:**

In this systematic review of 1263 studies, 74% of studies remained in early development stages, whereas only 25 (2%) progressed to clinical integration. Reporting standards use was critically low, and more than half of the studies were identified as having a high risk of bias.

**Meaning:**

These results suggest that a paradigm shift is urgently required in the medical AI literature—one that moves beyond retrospective validation of AI models toward the operationalization and prospective testing of complete AI systems.

## Teaser

This systematic review provides a comprehensive evaluation of artificial intelligence applications’ readiness for clinical implementation in the intensive care unit (ICU) and potential key barriers.

## Introduction

Health care professionals worldwide face increasing pressures from an increase in patient load, staff shortages, and escalating costs.^1^ These challenges potentially compromise their ability to deliver consistent high-quality care. Without timely interventions, these challenges are expected to worsen, leading to increasing disparities in care access, increased administrative burdens, and declining job satisfaction among health care professionals. Artificial intelligence (AI) holds promise in addressing these challenges by optimizing workflows, supporting clinical decision-making, and improving patient outcomes.^2^

Consequently, AI research in health care, particularly in data-rich environments such as the intensive care unit (ICU), has increased rapidly.^3^ However, despite progress, clinical operationalization remains limited.^3^ An important factor in this limited integration is lack of external validation and prospective implementation studies. A recent review found that only 9% of the US Food and Drug Administration (FDA)–approved AI applications included prospective studies for postmarket surveillance, and less than 2% provided detailed scientific publications on safety and efficacy.^4^ Moreover, journal editors increasingly emphasize the value of prospective assessment when evaluating AI applications, reflecting the broader recognition that retrospective performance on historical datasets alone fails to capture clinical complexities.^5,6^ This finding highlights the need for robust oversight, rigorous evaluation, and transparent reporting to ensure responsible integration of AI solutions into everyday clinical practice. Additional factors contributing to limited implementation of AI into health care include inadequate workflow integration, complex and time-consuming compliance with the appropriate legislation, slow clinical adoption, and limited return on investment.^7^ Additionally, most funding is predominantly directed toward development of new AI models instead of implementation of existing ones.^8,9,10^

Given these challenges, continuous and up-to-date scientific evaluations of AI applications are essential to make sure we are progressing toward implementation. Traditional systematic reviews provide a reliable and comprehensive scientific overview of existing literature that guides practice, policy, and future research. However, they often struggle to keep pace with the rapid advancements in AI, resulting in outdated insights by the time they are published.^11,12^ In contrast, living systematic reviews (LSRs) offer a dynamic solution.^13,14^ They not only update the latest evidence but also highlight differences compared with the previous review, showcasing progression and persistent challenges over time. This approach is particularly beneficial in rapidly evolving fields, such as AI, or in dynamic clinical settings, such as in the ICU, where up-to-date knowledge is critical for operational AI models and clinical decision-making.

In June 2021, a systematic review underscored the need to advance AI models from development to clinical implementation in the ICU.^3^ In this comprehensive update, we examine the progression of AI models in the ICU over time, focusing on technical maturity, risk of bias, use of reporting standards, and geographic contributions. Given recent advancements in large language models (LLMs) and reinforcement learning, this review includes research on these types of AI. Leveraging these insights, the aim of this review is to provide a comprehensive evaluation of AI applications’ readiness for clinical implementation and potential key barriers.

## Methods

This systematic review used the Preferred Reporting Items for Systematic Reviews and Meta-Analyses (PRISMA) reporting guideline.^15^ Prior to the literature search, this review was registered in the Prospective Register of Systematic Reviews (PROSPERO) database.^16^ We continued a previous search^3^ in publicly available databases and update findings and publications, transforming this in an AI-ICU LSR. This approach is essential for ensuring the review remains current and relevant in the rapidly evolving field of AI in health care, incorporating previous findings, allowing for longitudinal comparisons over time, effectively capturing progress, and concurrently identifying key challenges.

### Search Strategy

The search was continued in 5 databases (Embase, MEDLINE ALL, Web of Science Core Collection, Cochrane Central Register of Controlled Trials, and Google Scholar) from July 28, 2020, to June 10, 2024. The search strategy was expanded with terms for generative AI and reinforcement learning due to recent advancements in these areas. The medical library of the Erasmus Medical Center assisted with the development of the search strategy. The databases were searched for keywords and synonyms for *intensive care unit*, *artificial intelligence*, and *decision support*. To capture the full scope of the development and deployment of generative AI in health care, the search strategy was modified to include non–decision support studies for generative AI. This adjustment was made given the anticipated use of generative AI for administrative purposes. The full search strategy is available in eAppendix 1 in Supplement 1.

### Inclusion Criteria

Publications were considered for inclusion if they evaluated an AI application designed for use within the ICU, focused exclusively on adult patients (aged ≥16 years), were available in English in full text, and were published as original research. Publications were excluded when the data used to train the model were not collected during ICU stay or when the scope of the model extended beyond the ICU. An AI application was defined as the outcome of an algorithm trained on large datasets. Only publications stating that the model belongs to the AI domain or its synonyms were accepted. Publications were eligible if they evaluated AI models for clinical impact. Both peer-reviewed literature and preprints were included in the analysis. Detailed inclusion criteria are provided in eAppendix 2 in Supplement 1.

### Study Selection

**Figure 1.**
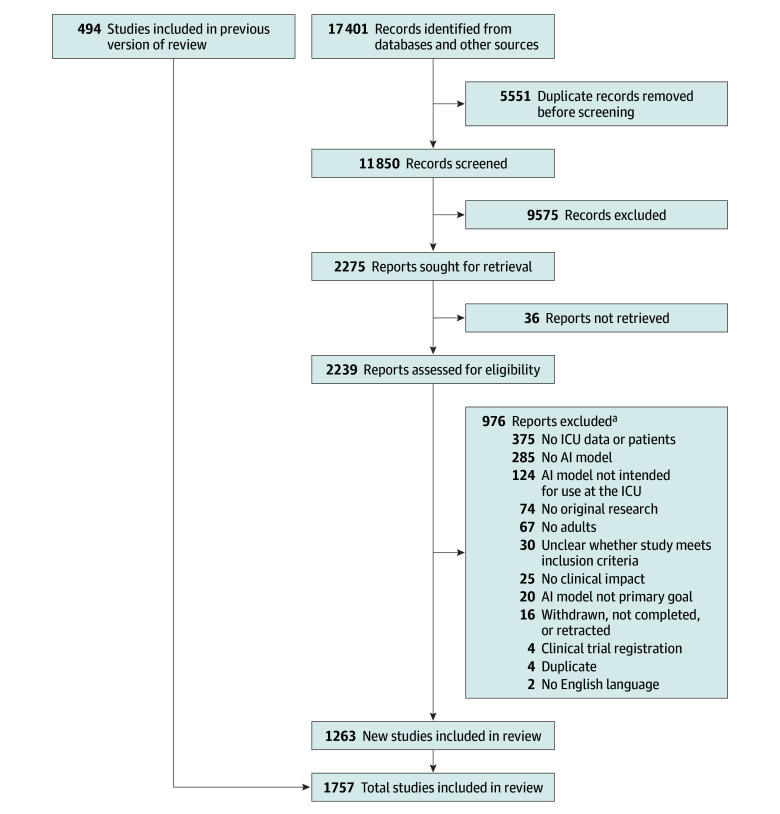
Flow Diagram of Study Review Process and the Exclusion of Studies AI indicates artificial intelligence; ICU, intensive care unit. ^a^The sum of the exclusion reasons is higher than the total number of excluded studies (n = 1012) because studies can be excluded based on multiple reasons.

The citations obtained from the search were imported into Endnote 20 (Clarivate Analytics) after removal of duplicates. Publications were then exported to Rayyan for screening.^17^ Rayyan is a web-based tool for title and abstract screening to improve quality and transparency in systematic reviews. Two authors (W.E.M.B. and J.J.v.W.) independently assessed eligibility of all title and abstracts. Disagreements were resolved by a third author (D.v.d.S.). The full texts of the selected articles were reviewed for inclusion by 3 authors (W.E.M.B., J.J.v.W., and J.D.W.) in Covidence systematic review software (Veritas Health Innovation) due to its preferred usability in customized data extraction templates compared with Rayyan. Reasons for exclusion during full-text screening were documented for each publication (Figure 1).

### Data Collection

A standardized data extraction template was created in Covidence systematic review software evaluating (1) aim of the AI model, (2) country of the lead author, (3) type of dataset used for the development and validation, (4) dataset and patient group size, (5) type of AI, and (6) reference to the use of a reporting standard. Detailed information is provided in eAppendix 3 in Supplement 1. The readiness of AI models for clinical implementation was assessed by mapping them to the appropriate technology readiness level (TRL), a concept originally developed by National Aeronautics and Space Administration and adapted for use in the ICU setting.^18^ The levels progress from the development of an AI model to the clinical implementation: problem identification (level 1), proposal of solution (level 2), model prototyping and development (levels 3 and 4), model validation (level 5), real-time testing (level 6), workflow integration (level 7), clinical testing (level 8), and integration in clinical practice (level 9) (eAppendix 4 in Supplement 1).

### Risk of Bias

We assessed the risk of bias (categorized as high, unclear, and low) using PROBAST (Prediction Model Study Risk of Bias Assessment Tool). This risk of bias tool is specifically designed for prediction model studies, making it more appropriate for studies involving AI models compared with other available risk of bias tools.^19^ We did not assess applicability because our focus was not on the value of AI for a specific therapeutic area.

### Statistical Analysis

Risk of bias and TRL assessments were performed on the combined set of studies from the 2021 review^3^ and the current search, whereas all other outcomes were calculated solely from studies newly identified in this living update. The aims of the AI models were presented against the TRLs with corresponding numbers and percentages. Risk of bias was plotted for the 4 PROBAST domains (categorized as participants, predictors, outcomes, and analysis), and the reported outcome was presented as percentages for each domain. The risk of bias identified in the previous review^1^ was added in a separate panel to facilitate a comparison across the different periods. The number of publications according to their TRL was plotted over the years, with the addition of data from the previous review,^3^ to optimize AI operationalization and AI publication evaluation over time for all publications. Given the aim of our review to provide a general overview of the current status of AI models rather than compare performance metrics or patient outcomes, meta-analysis and heterogeneity assessment were not applicable.

## Results

### Identification of Publications

A total of 17 401 publications were identified. After title and abstract screening, 2239 full-text publications were assessed for eligibility, of which 1263 met the inclusion criteria (Figure 1). The previous review^3^ (inception to July 2020) included 494 studies, indicating a 156% increase during the past 4 years, or a mean annualized growth rate of approximately 39%. The total number of studies identified for the sections that compare the previous review^3^ with the current review was 1757. For the current search, the primary reasons for exclusion were that the data were not exclusively from ICU patients (37%), no AI model was developed (28%), or the AI model was not intended for ICU use (12%). A total of 1233 studies of the current review included a discriminative AI model, of which 40 were a reinforcement learning model and 30 included a generative AI model, all of which used LLMs. The full list of included publications with corresponding study items of the current search is provided in eAppendix 5 in Supplement 1.

### Responsible Development and Deployment

**Figure 2.**
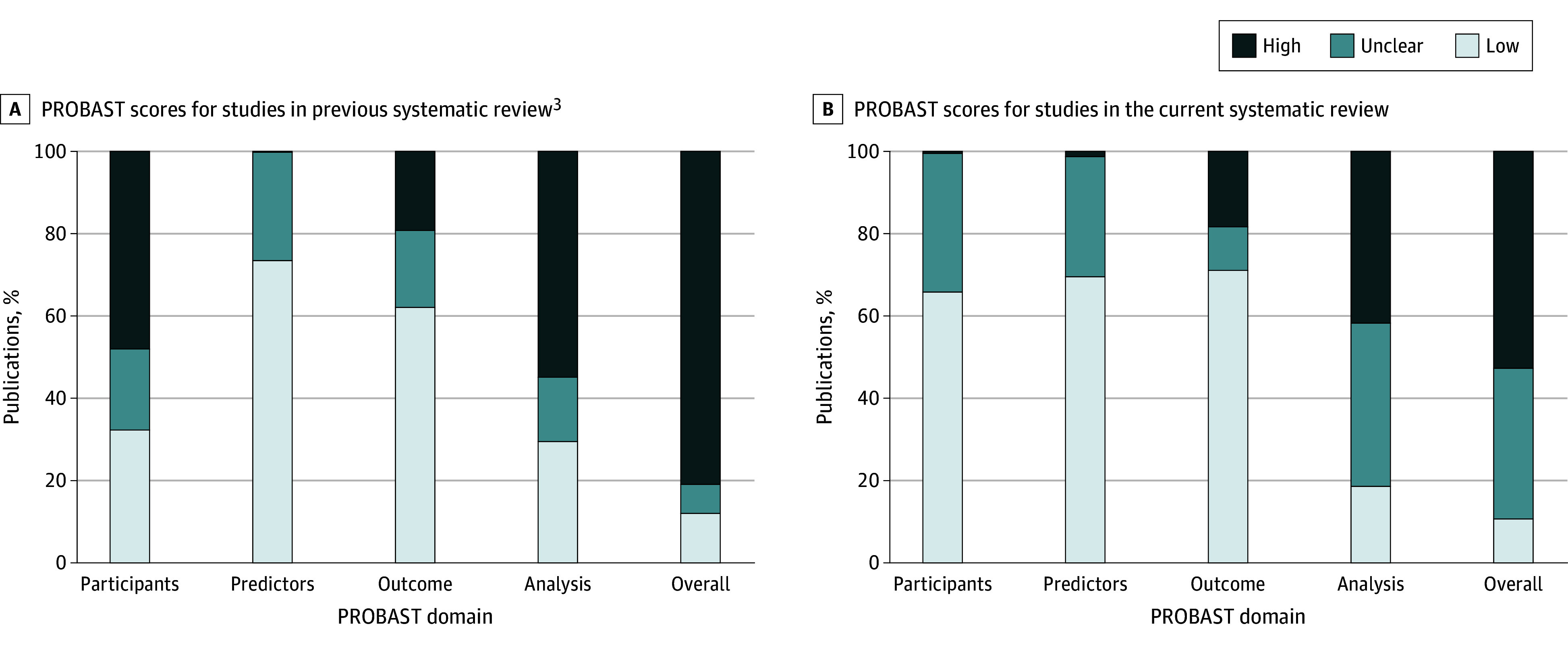
Risk of Bias According to the Different PROBAST (Prediction Model Study Risk of Bias Assessment Tool) Domains

Risk of bias assessment was performed for 1103 development studies using the PROBAST criteria (Figure 2). Overall, 581 of 1103 studies (53%) were rated as high risk of bias, primarily driven by the analysis domain (460 of 1103 [42%]) followed by the outcome domain (202 of 1103 [18%)), with minimal contributions from the predictors (14 of 1103 [1%]) and participants domain (5 of 1103 [<1%]). Of the 1103 studies, 372 (34%) had unclear risk of bias in the participants domain, 322 (29%) in the predictors domain, and 438 (40%) in the analysis domain. These findings reflect that the method was unclear for at least 1 item of these domains. Over time, the proportion of studies with a low risk of bias remained consistent, with 56 of 467 studies (12%) up to July 2020 and 118 of 1103 (11%) in the subsequent period. Although most studies in both searches were classified as having a high risk of bias (81% before July 2020 and 53% after) but decreased over time, the period after July 2020 saw a notable increase in the percentage of studies with an unclear risk of bias, increasing from 7% before July 2020 to 37% currently.

Only 207 of 1263 studies (16%) referenced the use of a reporting standard. Over time, the adoption of reporting standards showed a modest increase of 9 percentage points, increasing from 14% of studies in 2021 to 23% currently (eAppendix 6 and eFigure 1 in Supplement 1). Among the reporting standards, Transparent Reporting of a Multivariable Prediction Model for Individual Prognosis or Diagnosis (TRIPOD) and Strengthening the Reporting of Observational Studies in Epidemiology (STROBE) were the most commonly followed (eAppendix 6 and eFigure 2 in Supplement 1). The percentage of studies with an unclear risk of bias was 18% lower in studies that cited the use of reporting standards (47 of 188 [25%]) compared with studies that did not (391 of 915 [43%]) (eAppendix 6 and eFigure 3 in Supplement 1).

### Technology Readiness Level

**Figure 3.**
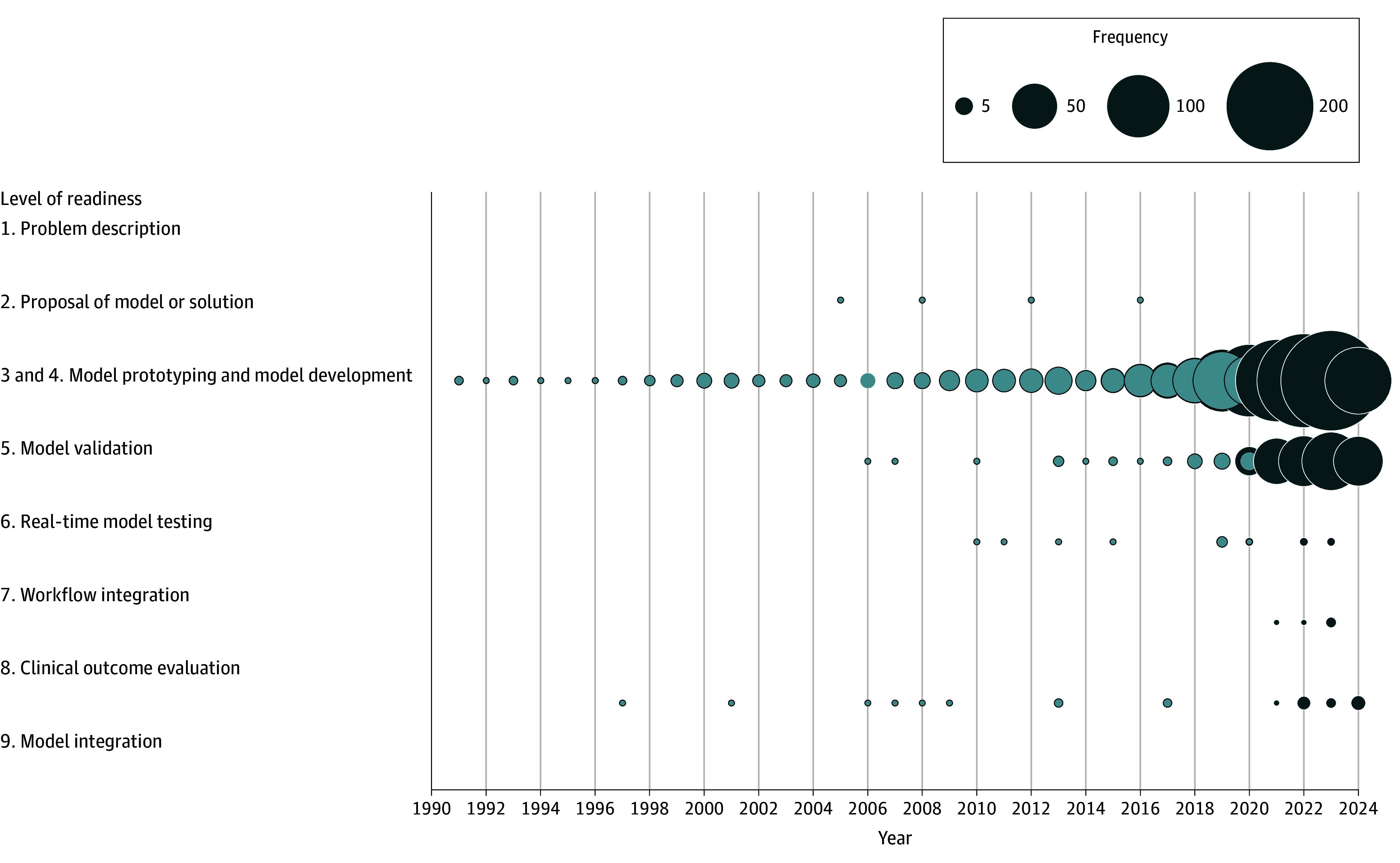
Studies Published According to Their Technology Readiness Level (TRL) and Year of Publication Light blue represents data until 2020; dark blue represents data from then onward (current findings). The total number of studies reporting on model development and prototyping (TRLs 3 and 4) increased rapidly from 184 studies in 2021 to 274 studies in 2023. Furthermore, the number of studies per year reporting on external validation (TRL 5) increased from 58 in 2021 to 93 in 2023.

Figure 3 shows the progression of AI models readiness over time. Of 1263 studies, 936 (74%) were categorized as level 4 or below on the level of readiness scale. Of the 1215 studies that developed an AI model, 447 studies (37%) used an internal dataset, defined as data collected from and restricted to the participating hospital’s own systems; 562 (46%) used a MIMIC (Medical Information Mart for Intensive Care) dataset (I, II, III or IV); and 78 (6%) used the open source eICU Collaborative Research Database dataset (eAppendix 5 and eFigure 4 in Supplement 1).

External validation (TRL 5), defined as the assessment of the algorithm’s performance in settings not considered during its development,^20^ was performed by 302 of 1263 studies (24%). Details are provided in eAppendix 6 and eFigure 5 in Supplement 1. Twenty-five of 1263 studies (2%) integrated the model into a clinical environment: 5 studies integrated the model without exposing the staff to the results (TRL 6), 5 studies assessed the feasibility of the integrated model (TRL 7), and 15 studies evaluated the model’s effectiveness (TRL 8). No studies were classified as TRL 9.

Among the 30 generative AI studies, 16 studies (53%) were classified as TRLs 3 and 4 and 14 studies (47%) as TRL 5. For the reinforcement learning studies, 33 studies (83%) were classified as TRLs 3 and 4 and 7 studies (17%) as TRL 5. No study was identified as having integrated an LLM (Table 1) or a reinforcement learning model into the clinical workflow.

**Table 1.** Aims of Generative AI Applications by Technology Readiness Level and by Their Intended Use

Aim of AI model	AI models, No. (%)				
	AI models with this aim	Technology readiness level			
		Intended for clinical use^a^		Not intended for clinical use^a^	
		3 and 4	5	3 and 4	5
Assessing clinical notes	10 (29)	1 (6)	1 (11)	4 (100)	4 (80)
Predicting mortality	5 (15)	3 (19)	2 (22)	0	0
Predicting complications	5 (15)	4 (25)	1 (11)	0	0
Diagnosing	3 (9)	2 (13)	1 (11)	0	0
Treatment recommendation	2 (6)	1 (6)	1 (11)	0	0
Predicting prognosis	2 (6)	0	2 (22)	0	0
Predicting length of stay	2 (6)	2 (13)	0	0	0
Predicting readmission	2 (6)	2 (13)	0	0	0
Predicting relevance of clinical information	1 (3)	1 (6)	0	0	0
Alarm management	1 (3)	0	1 (11)	0	0
Support in patient education	1 (3)	0	0	0	1 (20)
Total (accounting for multiple aims per article or model)^b^	34 (100)	16 (100)	9 (100)	4 (100)	5 (100)

Abbreviation: AI, artificial intelligence.

^a^ Intended for clinical use refers to AI applications designed to directly support clinical decision-making or patient care (ie, diagnostic tools, clinical decision support systems, and treatment recommendations). Not intended for clinical use encompasses AI tools primarily developed for administrative or logistical purposes, such as optimizing workflows or analyzing nonclinical data.

^b^ If a study described multiple aims, all aims were included. Percentages may not total 100 due to rounding.

In recent years, we observed an increase in studies that conducted external validation (Figure 3). The percentage of studies conducting external validation (TRL 5) increased from 14% in 2020 to 34% by 2024. Between 2021 and 2024, 17% of TRL 5 studies achieved a low risk of bias rating vs 9% for studies at TRL less than 5. Despite the increasing volume of published studies annually, the transition from development and validation-focused studies (TRLs ≤5) to those integrating AI models into practice (TRLs >5) has remained minimal, increasing only slightly from 1% in 2020 to 3% in 2024. Notably, no study reached TRL 9 before 2021, and this remained unchanged as of June 2024.

### Aim of AI Model Relative to TRL

The distribution of AI model aims across different TRLs is summarized in Table 2. Among studies with TRLs of 5 or less, the most common aims were predicting mortality (403 of 1238 [33%]) or complications (391 of 1238 [32%]). In contrast, for studies with TRLs greater than 5, the primary aims shifted toward analyzing videos or images (13 of 25 [52%]) and predicting complications (5 of 25 [20%]). Notably, studies with TRLs greater than 5 did not focus on predicting mortality (0 of 25 [0%]), highlighting a shift in aims as models progressed beyond the development phase.

**Table 2.** Aim of AI Applications by Technology Readiness Level

Aim of AI model	AI models, No. (%)					
	AI models with this aim	Technology readiness level				
		3 and 4	5	6	7	8
Predicting mortality^a^	403 (29)	300 (29)	103 (32)	0	0	0
Predicting complications	396 (29)	286 (28)	105 (32)	2 (40)	1 (20)	2 (13)
Predicting prognosis	74 (5)	58 (6)	14 (4)	1 (20)	0	1 (7)
Determining physiological values	72 (5)	56 (5)	16 (5)	0	0	0
Assessing videos and images^b^	62 (4)	37 (4)	12 (4)	1 (20)	3 (60)	9 (60)
Improving mechanical ventilation	53 (4)	42 (4)	8 (2)	0	0	3 (20)
Predicting length of stay	52 (4)	48 (5)	4 (1)	0	0	0
Classifying subpopulations	52 (4)	39 (4)	13 (4)	0	0	0
Treatment recommendation	49 (4)	36 (3)	13 (4)	0	0	0
Predicting need for resource	37 (3)	25 (2)	12 (4)	0	0	0
Predicting readmission	27 (2)	22 (2)	5 (2)	0	0	0
Diagnosing	25 (2)	24 (2)	1	0	0	0
Assessing clinical notes	17 (1)	10 (1)	7 (2)	0	0	0
Improving prognostic models or risk scoring system	13 (1)	11 (1)	2 (1)	0	0	0
Classification of signals	9 (1)	8 (1)	1	0	0	0
Predicting clinical score	8 (1)	5 (<1)	2 (1)	1 (20)	0	0
Predicting an event	8 (1)	7 (1)	1	0	0	0
Alarm management	6 (<10)	5 (<1)	1	0	0	0
Predicting medication administration	4 (<1)	3 (<1)	1	0	0	0
Predicting relevance of clinical information	3 (<1)	3 (<1)	0	0	0	0
Detecting spurious recorded values	3 (<1)	3 (<1	0	0	0	0
Determining physiological thresholds	2 (<1)	1 (<1)	1	0	0	0
Predicting omittable lab test	1 (<1)	0	1	0	0	0
Support in patient education	1 (<1)	0	1	0	0	0
Improving communication	1 (<1)	0	0	0	1 (20)	0
Total (accounting for multiple aims per article or model)^c^	1378 (100)	1029 (100)	324 (100)	5 (100)	5 (100)	15 (100)

Abbreviation: AI, artificial intelligence.

^a^ Predicting mortality was a key aim in studies with technology readiness levels less than 5 (403 of 1238 [33%]), whereas studies with technology readiness levels greater than 5 did not focus on this aim (0 of 25 [0%]).

^b^ Studies with technology readiness levels greater than 5 (13 of 25 [52%]) commonly focused on assessing videos and images (13 of 25 [52%]), whereas less than 5% (49 of 1238) of studies with technology readiness levels less than 5 addressed this aim.

^c^ If a study described multiple aims, all aims were included. Percentages may not total 100 due to rounding.

### Geographic Distribution of AI Level of Readiness

China and the US are the leading contributors to ICU-AI studies overall. For studies at TRLs of 5 or lower, China accounts for 367 of 1238 (30%) and the US for 295 of 1238 (24%) of contributions. However, when focusing on studies integrated into clinical workflows (TRLs >5), China’s contribution decreases substantially to 1 of 25 studies (4%), whereas the US remains a lead contributor at 9 of 25 studies (36%), followed by Taiwan at 3 of 25 studies (12%) (eAppendix 5 and eFigures 6 and 7 in Supplement 1).

## Discussion

Our systematic review found that despite the steep increase in research on AI in the ICU in recent years, most of this growth is driven by a notable increase in retrospective studies, indicating that AI models are increasingly developed but not used in clinical practice. Although the proportion of studies with a high risk of bias has decreased during the past few years, the number of studies with unknown risk has, conversely, increased, and the adoption of reporting standards remains minimal.

Despite a surge in AI research, the anticipated progression toward clinical implementation remains limited across all types of AI, including generative applications. This ongoing gap between development and implementation is concerning because it represents missed opportunities for improved patient care and inefficient use of resources. Key barriers include a lack of external validation, limited prospective evaluation, poor workflow integration, cumbersome legislation, slow clinical adoption, insufficient business cases, and inadequate funding.^3,7,21^ To address these challenges, we advocate for comprehensive strategies and collective implementation efforts that prioritize funding allocation, strong collaboration for external validation, prospective evaluation, and continuous monitoring. Such coordinated efforts are essential to facilitate the effective and sustainable adoption in clinical practice. Regular updates, such as this LSR, are crucial for tracking progress and identifying ongoing challenges, ensuring the responsible and impactful integration of AI systems to advance patient care.

Our findings align with a recent review by Han et al,^22^ which highlighted the scarcity of real-world evaluations and uneven geographic and specialty distributions in AI randomized trials. However, our review provides a more dynamic and actionable assessment through the use of technical maturity using TRL and comprehensive bias assessment, offering a clearer view of AI’s readiness for clinical adoption. By adopting an approach similar to an LSR, we capture not only the current state of AI research but also its evolution over time, identifying persistent gaps that must be addressed for successful clinical implementation.

One notable issue is the mismatch between the high number of registered clinical trials for AI-enabled medical devices and the limited clearance rates in national registries, such as by the FDA.^23^ This issue points to systemic hurdles in translating research into practice. Ethical checklists, such as those proposed by Ning et al,^24^ and the integration of implementation science principles, as suggested by van de Sande et al^25^ and Longhurst et al,^26^ will be critical to bridging the gap between development and real-world application.^25,26^ Together with frameworks, such as the STANDING Together consensus,^27^ for dataset transparency and networks focused on AI integration maturity in health care systems, such as the Coalition for Healthcare AI (CHAI)^28^ and the Trustworthy & Responsible AI Network Europe (TRAIN)–Europe,^29^ these initiatives provide a foundation for establishing robust principles and way of working for responsible AI implementation. Providing an overview not only of biases in the studies but also of the evolution of the use of reporting standards over time and the AI model aims spread across TRLs is critical to inform the focus of these networks and frameworks. Uniformity, structured insights, and clear overviews, such as those provided by this review, contribute significantly to aligning stakeholders and prioritizing actionable steps. Our review, for instance, highlights that although the adoption of reporting standards has increased modestly (only 9% during the review period of 3 years), significant gaps remain. Similarly, the proportion of studies with an unclear risk of bias has increased, reflecting continued shortcomings in methodologic transparency. This trend not only undermines confidence in model validity but also hinders reproducibility and efficient progression toward safe clinical implementation.^30^

In the context of emerging technologies such as AI, including the recent integration of generative AI into health care, it is essential to establish uniform assessments of the literature to ensure responsible implementation.^31^ Although recent publications addressing ethical considerations,^24^ evaluations,^32^ and bioethical principles^33^ offer valuable insights individually, they use diverse standards and frameworks while focusing on a similar challenge, making it difficult to form a cohesive overview of the field or identify critical gaps. By evaluating progression over time using consistent terms and metrics and framing this approach as an LSR design that dynamically incorporates new studies and tracks longitudinal trends, our review provides a standardized and comprehensive perspective on the field’s evolution. This approach is particularly well suited for a rapidly evolving technology such as AI, ensuring that assessments remain relevant and comparable over time. First, the LSR approach is gaining increasing attention and is likely to be applied in more innovative ways in the future.^34^ Second, AI must be regarded as a transformative technology with system-wide implications, similar to those experienced during the COVID-19 pandemic when LSRs were introduced.^35^ Third, by avoiding unnecessary duplication of the review processes and resource allocation, the LSR reduces the workload and time investment typically associated with repeated full reviews.^36^ This approach underscores the necessity of prospective clinical evaluations to bridge the gap between retrospective performance on historical datasets and real-time testing on live patient populations. Such evaluations are critical to ensuring that AI systems transition from theoretical promise to practical impact in a way that is safe, effective, and aligned with the core values of health care.

Bias and diversity remain pivotal challenges because health care AI models often rely on datasets that underrepresent marginalized groups, reinforcing historical inequities. For example, highlighted lower diagnosis rates for Black women and gender stereotyping in AI models.^37^ Addressing these gaps requires fairness audits, increased dataset diversity, and inclusive research practices, alongside transparency in reporting standards. Although our review included geographic distribution and a variety of datasets, it is not yet fully representative of global diversity, emphasizing the need for broader efforts to ensure equitable AI development. In addition, bias and diversity are critical issues that were not fully captured within PROBAST assessment of bias. Existing guidelines have improved the applicability and reporting of AI in health care but continue to fall short in addressing critical ethical considerations. These considerations include algorithmic registration, training and performance requirements, exploration of algorithmic bias, privacy preservation, and AI adoption criteria.

### Limitations

Some limitations of this study must be acknowledged. First, given the unprecedented number and rapid pace of AI-related prediction models entering the medical literature, our systematic review may not capture all health care AI models. We specifically focused on ICU-specific models, thereby excluding broader clinical applications that, although valuable, do not directly inform ICU care. For instance, a recent cluster-randomized trial applying machine learning to real-time nursing documentation significantly reduced inpatient deterioration risk.^38^ Nonetheless, this systematic review offers a comprehensive overview that, through the use of consistent search terms, provides valuable insight into the evolution of AI in health care over time. This allows us to track progress, transitions, and ongoing challenges in the field. Second, this review has identified several models using generative AI, reinforcement learning, unsupervised learning, and natural language processing. Although these models were included in the search, PROBAST was not applied to assess their risk of bias because PROBAST was specifically designed for traditional prediction model studies. TRIPOD-LLM was not used in the assessment because it was released after this study was performed.^31^ Third, PROBAST was not developed specifically for AI models. PROBAST specifically for AI is currently under development but was not available at the time this review was conducted.^39^ Fourth, this review did not follow individual AI models that proceed in successive TRLs over time. However, this insight might offer valuable information on how AI models should be adapted for different TRLs, identifying patterns and dependencies that guide toward more effective strategies in the field. Fifth, although several journal guidelines suggest a 2-year interval for evaluating the necessity of updates in living reviews, especially during crises such as the COVID-19 pandemic, this update comes after 4 years.^34^ For emerging technologies such as AI in health care, we believe, however, that regular updates are essential to track developments and address persistent challenges over time. Updates, such as those provided in this LSR, are vital for identifying gaps in the field.

## Conclusions

Our systematic review highlights a steep increase in AI research within intensive care medicine, largely driven by retrospective studies, but progress toward clinical implementation across all types of AI, including generative AI, remains limited. These findings suggest that a paradigm shift is urgently required to move toward the operationalization and prospective testing of AI applications to warrant their clinical benefit and impact. Living systematic AI reviews could provide an essential framework for tracking progress and identifying persistent gaps to expedite this transition.

## Notes

Supplement 1. **eAppendix 1.** Search Strategy **eAppendix 2.** Inclusion Criteria **eAppendix 3.** Data Collection **eAppendix 4.** Technology Readiness Level **eAppendix 5.** Study Items of Included Publications **eAppendix 6.** Results
eReferences

Supplement 2.
Data Sharing Statement
