# Supplementary material for: Operationalization of Artificial Intelligence Applications in the Intensive Care Unit: A Systematic Review
Source: JAMA Netw Open. 2025 Jul 23;8(7):e2522866. doi: 10.1001/jamanetworkopen.2025.22866 (PMC12287835; doi:10.1001/jamanetworkopen.2025.22866)
Supplement: Supplement 1. — eAppendix 1. Search Strategy eAppendix 2. Inclusion Criteria eAppendix 3. Data Collection eAppendix 4. Technology Readiness Level eAppendix 5. Study Items of Included Publications eAppendix 6. Results eReferences [file jamanetwopen-e2522866-s001.pdf]

Supplemental Online Content

Berkhout WEM, van Wijngaarden JJ, Workum JD, et al. Operationalization of artificial intelligence applications in the intensive care unit: a systematic review. *JAMA Netw Open*. 2025;8(7):e2522866. doi:10.1001/jamanetworkopen.2025.22866

eAppendix 1. Search Strategy
eAppendix 2. Inclusion Criteria
eAppendix 3. Data Collection
eAppendix 4. Technology Readiness Level
eAppendix 5. Study Items of Included Publications
eAppendix 6. Results
eReferences

This supplementary material has been provided by the authors to give readers additional information about their work.

© 2025 Berkhout WEM et al. *JAMA Network Open.*

eAppendix 1. Search strategy

The search from the previous systematic review published in 2021[1] was continued in the five databases from July 28, 2020 to June 10, 2024. The search strategy was expanded to include terms related to generative artificial intelligence (AI) and reinforcement learning, reflecting recent advancements in these fields. To cover the complete scope of the development and deployment of generative AI in healthcare, the strategy was adjusted to include non-decision support generative AI studies, acknowledging the potential use of generative AI for administrative tasks. The result presented in the table includes the records from the previous systematic review published in 2021[1].

Database searched	via	Years of coverage	Records	Records after duplicates removed
Medline ALL	Ovid	1946 - Present	4683	4619
Embase	Embase.com	1971 - Present	14448	10657
Web of Science Core Collection*	Web of Knowledge	1975 - Present	5752	2153
Cochrane Central Register of Controlled Trials	Wiley	1992 - Present	316	167
Other sources: Google Scholar**			847	709
**Total**			**26046**	**18305**

*Science Citation Index Expanded (1975-present) ; Social Sciences Citation Index (1975-present) ; Arts & Humanities Citation Index (1975-present) ; Conference Proceedings Citation Index- Science (1990-present) ; Conference Proceedings Citation Index- Social Science & Humanities (1990-present) ; Emerging Sources Citation Index (2005-present)

**Google Scholar was searched via "Publish or Perish" to download the results in EndNote.

No other database limits were used than those specified in the search strategies.

Embase

(('artificial intelligence'/exp OR 'machine learning'/exp OR 'natural language processing'/de OR 'generative pretrained transformer'/exp OR 'large language model'/exp OR (AI OR automated-reason* OR computer-heurist* OR ((natural) NEAR/3 (language) NEAR/3 (proces*)) OR ((bayes*) NEAR/3 (combiner* OR network*)) OR ((nearest) NEAR/3 (neighbo*)) OR ((random*) NEAR/3 (forest*)) OR ((support*) NEAR/6 (vector*) NEAR/6 (machine* OR active)) OR ((extract*) NEAR/6 (feature*) NEAR/6 (vector*)) OR ((artificial* OR ambient* OR machine* OR algorithm* OR neural) NEAR/3 (intelligen* OR network*)) OR ((automat*) NEAR/3 (pattern*) NEAR/3 (recogni*)) OR ((learn*) NEAR/3 (algori*)) OR ((machine* OR deep OR supervis* OR unsupervis* OR bayes OR reinforcement*) NEAR/3 (learn* OR vector*)) OR ((foundation*) NEXT/3 (model)) OR ((generat*) NEAR/6 (pretrain* OR pre-train*) NEAR/6 (transform*)) OR ((large*) NEAR/6 (languag*) NEAR/6 (model*)) OR chatGPT OR chat-GPT OR LLM):ab,ti,kw) AND ('intensive care'/exp OR 'intensive care unit'/exp OR 'critically ill patient'/de OR (ICU OR IC OR ((intensive OR critical) NEAR/3 (care OR therapy OR unit* OR patient* OR department*))):ab,ti,kw) AND ('decision support system'/exp OR 'computer assisted diagnosis'/exp OR 'prediction and forecasting'/exp OR 'predictive value'/de OR 'clinical assessment tool'/de OR 'decision support system'/exp OR 'hospital care'/de OR 'length of stay'/de OR 'hospitalization'/de OR (use OR uses OR role* OR predict* OR diagnos* OR assessment* OR support* OR effectiveness* OR application* OR prognos* OR forecast* OR ((decision*) NEAR/3 (system* OR support* OR make* OR making OR technique*))):ab,ti,kw) NOT ([Conference Abstract]/lim) NOT ((animal/exp OR animal*:de OR nonhuman/de) NOT ('human'/exp)) AND [English]/lim) OR (('generative pretrained transformer'/exp OR 'large language model'/exp OR (((foundation*) NEXT/3 (model)) OR ((generat*) NEAR/6 (pretrain* OR pre-train*) NEAR/6 (transform*)) OR ((large*) NEAR/6 (languag*) NEAR/6 (model*)) OR chatGPT OR chat-GPT OR LLM):ab,ti,kw) AND ('intensive care'/exp OR 'intensive care unit'/exp OR 'critically ill patient'/de OR (ICU OR IC OR ((intensive OR critical) NEAR/3 (care OR therapy OR unit* OR patient* OR department*))):ab,ti,kw) NOT ([Conference Abstract]/lim) NOT ((animal/exp OR animal*:de OR nonhuman/de) NOT ('human'/exp)) AND [English]/lim)

Medline(Ovid)

((exp "Artificial Intelligence"/ OR exp "Machine Learning"/ OR (AI OR automated-reason* OR computer-heurist* OR ((natural) ADJ3 (language) ADJ3 (proces*)) OR ((bayes*) ADJ3 (combiner* OR network*)) OR ((nearest) ADJ3 (neighbo*)) OR ((random*) ADJ3 (forest*)) OR ((support*) ADJ6 (vector*) ADJ6 (machine* OR active)) OR ((extract*) ADJ6 (feature*) ADJ6 (vector*)) OR ((artificial* OR ambient* OR machine* OR algorithm* OR neural) ADJ3 (intelligen* OR network*)) OR ((automat*) ADJ3 (pattern*) ADJ3 (recogni*)) OR ((learn*) ADJ3 (algori*)) OR ((machine* OR deep OR supervis* OR unsupervis* OR bayes OR reinforcement*) ADJ3 (learn* OR vector*)) OR ((foundation*) ADJ3 (model)) OR ((generat*) ADJ6 (pretrain* OR pre-train*) ADJ6 (transform*)) OR ((large*) ADJ6 (languag*) ADJ6 (model*)) OR chatGPT OR chat-GPT OR LLM).ab,ti,kf.) AND (exp "Intensive Care Units"/ OR exp "Critical Care"/ OR (ICU OR IC OR ((intensive OR critical) ADJ3 (care OR therapy OR unit* OR patient* OR department*))).ab,ti,kf.) AND ("Forecasting"/ OR "Hospitalization"/ OR "Predictive Value of Tests"/ OR exp "Diagnosis, Computer-Assisted"/ OR ("use" OR uses OR role* OR predict* OR diagnos* OR assessment* OR support* OR effectiveness* OR application* OR prognos* OR forecast* OR ((decision*) ADJ3 (system* OR support* OR make* OR making OR technique*))).ab,ti,kf.) NOT (news OR congres* OR abstract* OR book* OR chapter* OR dissertation abstract*).pt. NOT (exp animals/ NOT humans/) AND english.la.) OR (((((foundation*) ADJ3 (model)) OR ((generat*) ADJ6 (pretrain* OR pre-train*) ADJ6 (transform*)) OR ((large*) ADJ6 (languag*) ADJ6 (model*)) OR chatGPT OR chat-GPT OR LLM).ab,ti,kf.) AND (exp "Intensive Care Units"/ OR exp "Critical Care"/ OR (ICU OR IC OR ((intensive OR critical) ADJ3 (care OR therapy OR unit* OR patient* OR department*))).ab,ti,kf.) NOT (news OR congres* OR abstract* OR book* OR chapter* OR dissertation abstract*).pt. NOT (exp animals/ NOT humans/) AND english.la.)

Web-of-Science Core Collection

TS=(((AI OR automated-reason* OR computer-heurist* OR ((natural) NEAR/2 (language) NEAR/2 (proces*)) OR ((bayes*) NEAR/2 (combiner* OR network*)) OR ((nearest) NEAR/2 (neighbo*)) OR ((random*) NEAR/2 (forest*)) OR ((support*) NEAR/5 (vector*) NEAR/5 (machine* OR active)) OR ((extract*) NEAR/5 (feature*) NEAR/5 (vector*)) OR ((artificial* OR ambient* OR machine* OR algorithm* OR neural) NEAR/2 (intelligen* OR network*)) OR ((automat*) NEAR/2 (pattern*) NEAR/2 (recogni*)) OR ((learn*) NEAR/2 (algori*)) OR ((machine* OR deep OR supervis* OR unsupervis* OR bayes OR reinforcement*) NEAR/2 (learn* OR vector*)) OR ((foundation*) NEAR/2 (model)) OR ((generat*) NEAR/5 (pretrain* OR pre-train*) NEAR/5 (transform*)) OR ((large*) NEAR/5 (languag*) NEAR/5 (model*)) OR chatGPT OR chat-GPT OR LLM)) AND ((ICU OR IC OR ((intensive OR critical) NEAR/2 (care OR therapy OR unit* OR patient* OR department*)))) AND ((use OR uses OR role* OR predict* OR diagnos* OR assessment* OR support* OR effectiveness* OR application* OR prognos* OR forecast* OR ((decision*) NEAR/2 (system* OR support* OR make* OR making OR technique*)))) OR ((((foundation*) NEAR/2 (model)) OR ((generat*) NEAR/5 (pretrain* OR pre-train*) NEAR/5 (transform*)) OR ((large*) NEAR/5 (languag*) NEAR/5 (model*)) OR chatGPT OR chat-GPT OR LLM) AND ((ICU OR IC OR ((intensive OR critical) NEAR/2 (care OR therapy OR unit* OR patient* OR department*)))))) AND LA=(English) AND DT=(Article OR Review)

Cochrane Central

(((AI OR automated NEXT/1 reason* OR computer NEXT/1 heurist* OR ((natural) NEAR/3 (language) NEAR/3 (proces*)) OR ((bayes*) NEAR/3 (combiner* OR network*)) OR ((nearest) NEAR/3 (neighbo*)) OR ((random*) NEAR/3 (forest*)) OR ((support*) NEAR/6 (vector*) NEAR/6 (machine* OR active)) OR ((extract*) NEAR/6 (feature*) NEAR/6 (vector*)) OR ((artificial* OR ambient* OR machine* OR algorithm* OR neural) NEAR/3 (intelligen* OR network*)) OR ((automat*) NEAR/3 (pattern*) NEAR/3 (recogni*)) OR ((learn*) NEAR/3 (algori*)) OR ((machine* OR deep OR supervis* OR unsupervis* OR bayes OR reinforcement*) NEAR/3 (learn* OR vector*)) OR ((foundation*) NEXT/3 (model)) OR ((generat*) NEAR/6 (pretrain* OR pre-train*) NEAR/6 (transform*)) OR ((large*) NEAR/6 (languag*) NEAR/6 (model*)) OR chatGPT OR chat-GPT OR LLM):ab,ti,kw) AND ((ICU OR IC OR ((intensive OR critical) NEAR/3 (care OR therapy OR unit* OR patient* OR department*))):ab,ti,kw) AND ((use OR uses OR role* OR predict* OR diagnos* OR assessment* OR support* OR effectiveness* OR application* OR prognos* OR forecast* OR ((decision*) NEAR/3 (system* OR support* OR make* OR making OR technique*))):ab,ti,kw)) OR (((((foundation*) NEXT/3 (model)) OR ((generat*) NEAR/6 (pretrain* OR pre-train*) NEAR/6 (transform*)) OR ((large*) NEAR/6 (languag*) NEAR/6 (model*)) OR chatGPT OR chat-GPT OR LLM):ab,ti,kw) AND ((ICU OR IC OR ((intensive OR critical) NEAR/3 (care OR therapy OR unit* OR patient* OR department*))):ab,ti,kw))

© 2025 Berkhout WEM et al. *JAMA Network Open.*

Google Scholar

"artificial intelligence"|"deep|machine|reinforcement learning"|"foundation models"|"large language model"
"intensive care"|ICU

'artificial intelligence'|'deep|machine|reinforcement learning'|'foundation models'|'large language model'
'intensive care'|ICU

© 2025 Berkhout WEM et al. *JAMA Network Open.*

eAppendix 2. Inclusion criteria

Table S1. Definitions of inclusion criteria

**Inclusion criteria**	**Definition**
Study should develop or deploy an AI model	The model is the result of an algorithm trained on exposure to large amounts of data.<br><br>Only publications stating that the model belongs to the artificial intelligence domain, or any of its synonyms, were included.
AI model for the ICU	The study should mention that the AI model is intended for use in the ICU. Therefore, studies are excluded if AI algorithms are utilized for associations, correlations or identifying predictive features without mention of the use of a model for future purposes in the clinical setting.<br><br>Regarding development studies, the input data should be gathered during ICU stay and all involved patients should be ICU patients.<br><br>If the AI model was developed on data of a non-ICU population, but validated or tested on exclusively ICU patients, the article is included.
AI model for clinical impact	Model is designed to directly support clinical decision-making or patient care, such as diagnostic tools, clinical decision support systems, and treatment recommendations. Models that are not for clinical impact include AI tools primarily developed for administrative or logistical purposes, such as optimizing workflows or analyzing non-clinical data.<br><br>If the article describes a generative AI model, the model does not have to be designed for clinical impact.
AI model for adults	The involved patients should be 16 years or older.<br><br>If age is not mentioned in the method section, the article is included.<br><br>If both children and adults are included, the article will be included if there is a separate model for adults.
Original paper	The study should contain new generated data.

AI = artificial intelligence, ICU = intensive care unit.

© 2025 Berkhout WEM et al. *JAMA Network Open.*

eAppendix 3. Data collection

Table S1. The data options available per data extraction subject

Data extraction subject	Data options
Aim of AI model	▪ Predicting mortality<br>▪ Predicting complications<br>▪ Predicting prognosis<br>▪ Determining physiological values<br>▪ Assessing videos and images<br>▪ Improving mechanical ventilation<br>▪ Predicting length of stay<br>▪ Classifying sub-populations<br>▪ Treatment recommendation<br>▪ Predicting need for resource<br>▪ Predicting readmission<br>▪ Diagnosing<br>▪ Assessing clinical notes<br>▪ Improving prognostic models/risk scoring system<br>▪ Classification of signals<br>▪ Predicting clinical score<br>▪ Predicting an event<br>▪ Alarm management<br>▪ Predicting medication administration<br>▪ Predicting relevance of clinical information<br>▪ Detecting spurious recorded values<br>▪ Determining physiological thresholds<br>▪ Predicting omittable lab test<br>▪ Support in patient education<br>▪ Improving communication<br>If the aim did not meet one of the predefined categories, it was categorized as 'Other'. If multiple aims could be linked to the study, all aims were registered.
Country of lead author	Free text
Type of dataset	▪ MIMIC (I, II, III, IV)<br>▪ eICU-CRD<br>▪ AmsterdamUMCdb<br>▪ HiRID<br>▪ Internal dataset*<br>▪ No information<br>If the type of dataset did not meet one of the predefined categories, it was categorized as 'Other type of dataset'. If the final dataset was composed out of multiple datasets, all datasets were registered.
Dataset size**	▪ 0 – 100<br>▪ 100 – 1000<br>▪ 1.000 – 10.000<br>▪ 10.000 – 100.000<br>▪ 100.000+
External dataset size**	▪ 0 – 100<br>▪ 100 – 1000<br>▪ 1.000 – 10.000<br>▪ 10.000 – 100.000<br>▪ 100.000+
Patient group size**	Free text
Type of AI***	▪ Generative AI<br>▪ Generative AI (Large language model)<br>▪ Discriminative AI<br>▪ Discriminative AI (Reinforcement learning)

© 2025 Berkhout WEM et al. *JAMA Network Open.*

Reference to the use of a reporting standard	▪ None<br>▪ TRIPOD/ TRIPOD+AI<br>▪ STROBE<br>▪ RECORD<br>▪ STARD/ STARD-AI<br>▪ CONSORT/ CONSORT-AI<br>▪ DECIDE-AI<br>▪ SPIRIT-AI<br>If the type of reporting standard did not meet one of the predefined categories, it was categorized as ‘Other’. If multiple reporting standards were cited by the study, all reporting standards were registered.

The categories for the type of dataset is based on the review of Sauer et al.[2] * Internal dataset is defined as data collected from and restricted to the participating hospital's own systems, such as their Electronic Health Record, and not available through open-source repositories. ** The number of samples in the dataset utilized for development of the model (‘Dataset size’) or for external validation of the model (‘External dataset size), and the number of patients involved in studies with a Technology Readiness Level above 7 (‘Patient group size’). *** If the study describes the use of large language models or reinforcement learning, this is displayed in brackets. AI = artificial intelligence, TRIPOD = Transparent Reporting of a multivariable prediction model for Individual Prognosis Or Diagnosis, STROBE = STrengthening the Reporting of OBservational studies in Epidemiology, STARD = Standards for Reporting Diagnostic accuracy studies, RECORD = REporting of studies Conducted using Observational Routinely-collected health Data, CONSORT = Consolidated Standards of Reporting Trials, DECIDE-AI = Developmental and Exploratory Clinical Investigations of DEcision support systems driven by Artificial Intelligence, SPIRIT = Standard Protocol Items: Recommendations for Interventional Trials, MIMIC = Medical Information Mart for Intensive Care, eICU-CRD = eICU Collaborative Research Database.

© 2025 Berkhout WEM et al. *JAMA Network Open.*

eAppendix 4. Technology Readiness Level

Table S1. Definition of Technology Readiness Level

Technology Readiness Level	Clinical definition	Explanation[3]
1	Problem identification	Studies that describe a clinical problem without an AI based solution.
2	Proposal of solution	Studies that propose an AI based solution for an identified problem, but do not develop the solution.
3 & 4	Model prototyping and development	Studies that use a dataset to develop an AI model and/or use internal validation to evaluate model performance.
5	Model validation	Studies in which AI model performance is evaluated using a different dataset than used during development (temporal, geographical or domain external validation[4]). The validation is done 'offline', indicating there is no real time test in a clinical setting.
6	Real-time testing	Studies in which AI models are fed real-time data. The model is integrated into the workflow. The results are 'silent', indicating that the staff is not exposed to the result of the model.
7	Workflow integration	Studies describing feasibility of the AI model in the clinical setting. Representative studies include pilot studies, qualitative evaluations on user experience, and assessments of technical feasibility or model validity within the clinical workflow. The model is integrated into the workflow and results are visible to the staff.
8	Clinical testing	Studies evaluating the effectiveness of the model in the clinical setting. Representative studies include comparative evaluations between the AI model and a control group, reflecting routine practice, established benchmarks, or outcomes prior to implementation (pre-post implementation). The model is integrated into the workflow and results are visible to the staff.
9	Integration into clinical practice	Studies describing models fully implemented and utilized for a longer period.

AI = artificial intelligence, ICU = intensive care unit.

© 2025 Berkhout WEM et al. *JAMA Network Open.*

eAppendix 5. Study items of included publications

Table S1. Collected study items for all included studies

Ref.	Lead author	Year	Country of lead author	Type of AI	Aim of study (categorized)	Aim of study (detailed)	Technology Readiness Level	Reporting standards	Data source	External data source	Dataset size	External dataset size	Patient group size	PROBAST - Participants	PROBAST - Predictors	PROBAST- Outcomes	PROBAST - Analysis	Overall risk of bias
5	Abad	2021	Canada	Discriminative AI	Predicting mortality	Early prediction of mortality for intensive care unit patients	3&4	None	eICU-CRD	N/A	100.000+	N/A	N/A	Unclear	Low	Low	Low	Unclear
6	Abad	2021	Canada	Discriminative AI	Predicting prognosis	Predicting discharge destination of critically ill	5	None	eICU-CRD	eICU-CRD	10.000-100.000	10.000 - 100.000	N/A	Low	Low	Low	Low	Low
7	Abbasi	2023	United States	Discriminative AI	Predicting complications	Predicting patient outcomes following cardiac surgery	3&4	None	Other type of dataset	N/A	100.000+	N/A	N/A	Low	High	Unclear	High	High
8	Abdullahi	2024	United States	Generative AI (Large language model)	Diagnostic	Enhance diagnosis of rare and complex diseases for continuing medical education	5	None	N/A	MIMIC III	N/A	0-100	N/A	N/A	N/A	N/A	N/A	N/A
9	Abououf	2024	Abu Dhabi	Discriminative AI	Predicting complications	Event and anomaly detection	3&4	None	MIMIC III	N/A	10.000-100.000	N/A	N/A	N/A	N/A	N/A	N/A	N/A
10	Abromavicius	2020	Lithuania	Discriminative AI	Predicting complications	Early detection of sepsis	3&4	None	Other type of dataset	N/A	10.000-100.000	N/A	N/A	Low	High	High	High	High
11	Aden	2024	United Kingdom	Generative AI (Large language model)	Assessing clinical notes	Predicting International Classification of Disease codes for intensive care unit patients	3&4	None	MIMIC III	N/A	No information	N/A	N/A	Low	Low	Low	High	High
12	Afshin-Pour	2023	United States	Discriminative AI	Predicting complications	Predicting the onset of ARDS in the ICU for patients with severe hypoxemia	3&4	None	Internal dataset	N/A	1.000-10.000	N/A	N/A	Low	Low	High	High	High
13	Agmon	2022	Israel	Discriminative AI	Predicting readmissions; Predicting length of stay	Predicting hospital length of stay and intensive care unit readmission	3&4	None	MIMIC III	N/A	10.000-100.000	N/A	N/A	Low	Unclear	Low	Unclear	Unclear
14	Ahmad	2021	United States	Discriminative AI	Predicting mortality	Predicting mortality in paralytic ileus at ICU	3&4	None	MIMIC III	N/A	1.000-10.000	N/A	N/A	Low	Low	Low	High	High
15	Ahmed	2020	United States	Discriminative AI	Predicting mortality	Predicting mortality in trauma patients admitted to ICU	3&4	None	MIMIC III	N/A	1.000-10.000	N/A	N/A	Low	Low	Low	High	High
16	Aikodon	2024	United Kingdom	Discriminative AI	Predicting complications	Early detection of increased decompensation risk and pace	3&4	None	MIMIC IV	N/A	10.000-100.000	N/A	N/A	Low	Low	Low	Low	Low
17	Akerlund	2022	Sweden	Discriminative AI	Classifying sub-populations	Identify distinct endotypes of TBI	3&4	None	Other type of dataset	N/A	1.000-10.000	N/A	N/A	N/A	N/A	N/A	N/A	N/A
18	Akiki	2021	United States	Discriminative AI	Predicting mortality	Predicting mortality among wound patients in the ICU	3&4	MDAR framework	MIMIC III	N/A	1.000-10.000	N/A	N/A	Low	Low	Low	High	High
19	Alabdulhafith	2023	Saudi Arabia	Discriminative AI	Predicting readmissions	Predicting readmissions after 30 days of intensive care unit discharge	3&4	None	MIMIC III	N/A	10.000-100.000	N/A	N/A	Low	Low	Low	High	High
20	Ala-Kokko	2022	Finland	Discriminative AI	Predicting clinical score	Producing the C-Trend Index parameter of the slow-wave activity in EEG	6	None	N/A	Internal dataset	N/A	0-100	N/A	Unclear	Unclear	Low	Unclear	Unclear
21	Alam	2021	Bangladesh	Discriminative AI	Predicting mortality	Predicting intensive care unit mortality	3&4	None	MIMIC III	N/A	1.000-10.000	N/A	N/A	N/A	N/A	N/A	N/A	N/A
22	Al-Dailami	2022	China	Discriminative AI	Predicting mortality; Predicting length of stay	Predicting length of stay and mortality at the intensive care unit	3&4	None	MIMIC IV, eICU-CRD	N/A	10.000-100.000	N/A	N/A	Low	Low	Low	Unclear	Unclear
23	Alderden	2022	United States	Discriminative AI	Predicting complications	Predicting pressure injuries in COVID-19 critical care patients	3&4	None	Internal dataset	N/A	100-1000	N/A	N/A	Low	Low	Unclear	High	High
24	Aldewereld	2022	China	Discriminative AI	Classifying sub-populations	Phenotyping of inpatient sepsis	3&4	None	Other type of dataset	N/A	1.000-10.000	N/A	N/A	Low	Low	High	Low	High
25	Aldhoayan	2023	Saudi Arabia	Discriminative AI	Predicting deterioration	Predicting deterioration in ICU patients	3&4	None	Internal dataset	N/A	100-1000	N/A	N/A	Low	Low	High	Low	High

© 2025 Berkhout WEM et al. *JAMA Network Open.*

**26**	Alfieri	2021	Italy	Discriminative AI	Predicting complications	Predicting acute kidney injury	3&4	None	MIMIC III, eICU-CRD	N/A	100-1000	N/A	N/A	Low	Low	High	Low	High
**27**	Alfieri	2022	Italy	Discriminative AI	Predicting complications	Early detection of severe oliguric AKI in ICU patients	5	None	N/A	AmsterdamUMCdb	N/A	10.000 - 100.000	N/A	Low	Low	High	High	High
**28**	Alfieri	2023	Italy	Discriminative AI	Predicting complications	Early prediction of acute kidney injury (stage 2/3 KDIGO)	5	None	MIMIC-III, AmsterdamUMC	Other type of dataset, eICU-CRD	1.000-10.000	1.000 - 10.000	N/A	Low	Low	Low	Low	Low
**29**	Alge	2024	United States	Discriminative AI	Predicting complications	Predicting sepsis prognosis	3&4	None	Internal dataset	N/A	1.000-10.000	N/A	N/A	Low	Unclear	High	Unclear	High
**30**	Alghatani	2021	United States	Discriminative AI	Predicting mortality; Predicting length of stay	Predicting of ICU lenght of stay and mortality	3&4	None	MIMIC III	N/A	10.000-100.000	N/A	N/A	Unclear	Low	Unclear	Unclear	Unclear
**31**	Alghatani	2022	United States	Discriminative AI	Predicting readmissions; Predicting deterioration, Determining physiological values	Predicting readmission, predicting abnormality, predicting next-day vital sign measurements	3&4	None	MIMIC III	N/A	10.000-100.000	N/A	N/A	Low	Low	Low	High	High
**32**	Alharbi	2021	Saudi Arabia	Discriminative AI	Predicting physiological values	Predicting the heart rate	3&4	None	MIMIC II	N/A	No information	N/A	N/A	Unclear	Low	Low	High	High
**33**	Al-Hindawi	2024	United Kingdom	Discriminative AI	Predicting complications	Predicting delirium	5	CONSORT/ CONSORT-AI	Internal dataset	Internal dataset	0-100	0-100	N/A	N/A	N/A	N/A	N/A	N/A
**34**	Alizadeh	2023	Iran	Discriminative AI	Predicting clinical score	Predicting the outcome of patients after neurosurgery	3&4	None	Internal dataset	N/A	1.000-10.000	N/A	N/A	Low	Low	Low	High	High
**35**	Al-Mamun	2021	United States	Discriminative AI	Predicting mortality	Predicting in-patient mortality in critically ill patients	3&4	None	Internal dataset	N/A	100-1000	N/A	N/A	Low	Low	Low	High	High
**36**	Alourani	2023	Saudi Arabia	Discriminative AI	Predicting mortality	Prediction of mortality	3&4	None	MIMIC III	N/A	No information	N/A	N/A	Low	Low	Low	Unclear	Unclear
**37**	Alsinglawi	2020	Australia	Discriminative AI	Predicting length of stay	Predicting cardiovascular length of stay	3&4	None	MIMIC III	N/A	1.000-10.000	N/A	N/A	Unclear	Low	Low	Low	Unclear
**38**	Alsinglawi	2022	Australia	Discriminative AI	Predicting length of stay	Predicting length of stay for lung cancer patients during ICU hospitalisation	3&4	None	MIMIC III	N/A	100-1000	N/A	N/A	Low	Low	Low	High	High
**39**	Alves	2019	Brazil	Discriminative AI	Predicting mortality	Predicting mortality in intensive care unit patients	3&4	None	Other type of dataset	N/A	1.000-10.000	N/A	N/A	Low	Low	Low	Low	Low
**40**	Amacher	2024	Switzerland	Generative AI (Large language model)	Predicting mortality; Predicting deterioration	Predicting mortality and poor neurological outcome for cardiac arrest patients admitted to the intensive care unit	5	TRIPOD/ TRIPOD+AI; STROBE	N/A	Internal dataset	N/A	100-1.000	N/A	N/A	N/A	N/A	N/A	N/A
**41**	Amador	2022	Brazil	Discriminative AI	Predicting mortality; Predicting complications	Predicting severe complications in the ICU (delirium, central line-associated bloodstream infection, ventilator-associated pneumonia)	5	None	Internal dataset	Internal dataset	1.000-10.000	100-1.000	N/A	Low	Low	Low	High	High
**42**	Amer	2021	Belgium	Discriminative AI	Determining physiological values	Predicting vital signs in COVID-19 patients at the intensive care unit	3&4	None	Other type of dataset	N/A	100-1000	N/A	N/A	Unclear	Low	High	Unclear	High
**43**	Amiri	2023	Denmark	Discriminative AI	Predicting prognosis	Predicting consciousness in intensive care unit patients	3&4	None	Internal dataset	N/A	0-100	N/A	N/A	Low	Low	Low	Unclear	Unclear
**44**	Amiri	2023	Denmark	Discriminative AI	Predicting complications	Predicting consciousness level in acute disorders of consciousness patients	3&4	None	Internal dataset	N/A	0-100	N/A	N/A	Low	Low	Low	Low	Low
**45**	Amrollahi	2020	United States	Generative AI (Large language model)	Predicting complications	Prediction of sepsis	3&4	None	MIMIC III	N/A	10.000-100.000	N/A	N/A	Low	Unclear	Unclear	High	High
**46**	An	2021	China	Discriminative AI	Classifying sub-populations	Classifying patients admitted to the intensive care unit based on disease severity and care needs	3&4	None	Internal dataset	N/A	100-1000	N/A	N/A	Low	Low	High	High	High

© 2025 Berkhout WEM et al. *JAMA Network Open.*

**47**	Andersson	2021	Sweden	Discriminative AI	Predicting complications	Predicting neurological outcome post out-of-hospital cardiac arrest	3&4	TRIPOD/ TRIPOD+AI	Other type of dataset	N/A	100-1000	N/A	N/A	Low	Low	Low	High	High
**48**	Ang	2021	Singapore	Discriminative AI	Predicting mortality	Predicting mortality risk	3&4	None	MIMIC III	N/A	10.000-100.000	N/A	N/A	Unclear	Low	Low	Unclear	Unclear
**49**	Anitha	2023	India	Discriminative AI	Improving mechanical ventilation	Predicting and controlling pressure and volume settings in mechanical ventilators in ICU settings	3&4	None	Internal dataset	N/A	No information	N/A	N/A	Low	Low	Low	Unclear	Low
**50**	Anitha	2024	India	Discriminative AI	Improving mechanical ventilation	Predicting the appropriate pressure and volume control settings for patients treated with mechanical ventilation	3&4	None	Internal dataset	N/A	No information	N/A	N/A	Low	Low	Low	Unclear	Unclear
**51**	Annapragada	2021	United States	Discriminative AI	Predicting complications	Predicting hypoxemia events in intensive care unit patients	3&4	TRIPOD/ TRIPOD+AI	eICU-CRD	N/A	No information	N/A	N/A	Low	Low	High	Unclear	High
**52**	Aoki	2022	Canada	Discriminative AI	Predicting mortality; Predicting length of stay; Diagnosing	Predicting phenotype, length of stay and mortality for patients admitted to the intensive care unit	3&4	None	MIMIC III	N/A	No information	N/A	N/A	Low	Unclear	Unclear	Unclear	Unclear
**53**	Apalak	2022	United States	Discriminative AI	Predicting complications	Prediction of sepsis	3&4	None	Internal dataset	N/A	10.000-100.000	N/A	N/A	Low	High	Low	Low	High
**54**	Aref	2023	United States	Discriminative AI	Predicting complications	Predicting sepsis	3&4	None	Other type of dataset	N/A	100-1000	N/A	N/A	Low	Low	Low	High	High
**55**	Asgari	2022	Iran	Discriminative AI	Predicting mortality	Predicting mortality in the intensive care unit	3&4	None	Internal dataset	N/A	1.000-10.000	N/A	N/A	Low	Low	Low	High	High
**56**	Ashrafi	2024	United States	Discriminative AI	Predicting mortality	Predicting mortality for mechanically ventilated intensive care unit patients	3&4	TRIPOD/ TRIPOD+AI	MIMIC III	N/A	10.000-100.000	N/A	N/A	Unclear	Low	Low	Unclear	Unclear
**57**	Asrian	2024	United States	Discriminative AI	Predicting mortality	Predicting 1-year mortality following a hip fracture	3&4	None	MIMIC IV	N/A	1.000-10.000	N/A	N/A	Low	Low	Low	High	High
**58**	Asuroglu	2020	Turkey	Discriminative AI	Predicting health improvement	Providing a prognosis solution for sepsis	3&4	None	MIMIC III	N/A	1.000-10.000	N/A	N/A	Low	Low	Low	High	High
**59**	Athaya	2021	Korea	Discriminative AI	Detecting spurious recorded values	Detecting Photoplethysmogram signal artifacts	3&4	None	MIMIC II	N/A	100-1000	N/A	N/A	Unclear	Low	High	High	High
**60**	Athaya	2022	United States	Discriminative AI	Determining physiological values	Estimating blood pressure in intensive care unit patients	3&4	None	MIMIC III	N/A	No information	N/A	N/A	Unclear	Low	Low	Unclear	Unclear
**61**	Au-Yeung	2021	United States	Discriminative AI	Diagnostic	Predicting heart arrhythmias in the intensive care unit	3&4	None	Other type of dataset	N/A	1.000-10.000	N/A	N/A	Low	Low	High	High	High
**62**	Ayad	2022	Germany	Discriminative AI	Determining physiological values	Classifying abnormal lab values the next time the test is done	5	None	MIMIC III	eICU-CRD	10.000-100.000	10.000 - 100.000	N/A	Low	Low	High	Unclear	High
**63**	Bacariza	2023	Portugal	Discriminative AI	Assessing videos and images	Determining the left ventricular ejection fraction	8	None	N/A	N/A	N/A	N/A	95	N/A	N/A	N/A	N/A	N/A
**64**	Baedorf-Kassis	2023	United States	Discriminative AI	Improving mechanical ventilation	Detecting reverse triggering in mechanically ventilated patients	3&4	None	Internal dataset	N/A	0-100	N/A	N/A	Low	Low	High	Unclear	High
**65**	Bahador	2020	Finland	Discriminative AI	Detecting spurious recorded values	Detecting artifacts in EEG signals	3&4	None	Internal dataset	N/A	0-100	N/A	N/A	Low	Low	High	Unclear	High
**66**	Bai	2022	China	Discriminative AI	Classifying sub-populations	Cluster sepsis-associated acute respiratory distress syndrome (ARDS)	5	None	eICU-CRD	MIMIC IV	1.000-10.000	1.000 - 10.000	N/A	Low	Low	Low	High	High
**67**	Baker	2020	Australia	Discriminative AI	Predicting mortality	Predicting mortality risk in intensive care unit	3&4	None	MIMIC III	N/A	0-100	N/A	N/A	Low	Low	Low	Unclear	Unclear
**68**	Bakkes	2020	Italy	Discriminative AI	Improving mechanical ventilation	Automatic detection and classification of patient-ventilator asychrony	3&4	None	Internal dataset	N/A	1.000-10.000	N/A	N/A	Unclear	Low	Unclear	Unclear	Unclear
**69**	Bakkes	2023	Netherlands	Discriminative AI	Improving mechanical ventilation	Detecting patient-ventilator asynchrony	3&4	None	Internal dataset	N/A	1.000-10.000	N/A	N/A	Unclear	Low	Low	Unclear	Unclear
**70**	Baniasadi	2021	Iran	Discriminative AI	Predicting complications	Predicting sepsis in intensive care unit patients	3&4	None	Other type of dataset	N/A	10.000-100.000	N/A	N/A	Low	Low	High	High	High

© 2025 Berkhout WEM et al. *JAMA Network Open.*

**71**	Bao	2023	China	Discriminative AI	Predicting mortality	Predicting sepsis patients' mortality	5	None	MIMIC IV	eICU-CRD	10.000-100.000	1.000 - 10.000	N/A	Low	Low	High	Unclear	High
**72**	Barakat	2023	Germany	Discriminative AI	Determining physiological values	Predicting partial pressure of arterial oxygen and carbon dioxide, bicarbonate concentration and blood acidity level	3&4	None	MIMIC III	N/A	No information	N/A	N/A	Unclear	Unclear	Low	Unclear	Unclear
**73**	Baral	2021	Australia	Discriminative AI	Alarm reduction	Predicting false alarm for cardiac arrest in sepsis patients	3&4	None	MIMIC III	N/A	1.000-10.000	N/A	N/A	Low	Low	Low	High	High
**74**	Barchitta	2021	Italy	Discriminative AI	Predicting complications	Predicting healthcare-associated infections in ICU patients	3&4	None	Other type of dataset	N/A	10.000-100.000	N/A	N/A	Low	Low	Low	High	High
**75**	Barchitta	2021	Italy	Discriminative AI	Predicting mortality	Predicting mortality in intensive care unit patients	3&4	None	Other type of dataset	N/A	1.000-10.000	N/A	N/A	Low	Low	Low	High	High
**76**	Bardak	2021	Turkey	Discriminative AI	Predicting mortality; Predicting length of stay	Prediction of mortality and length of stay in intensive care patients	3&4	None	MIMIC III	N/A	10.000-100.000	N/A	N/A	Low	Low	Low	Unclear	Unclear
**77**	Bashar	2020	United States	Discriminative AI	Predicting complications	Detecting atrial fibrillation during sepsis in intensive care unit patients	3&4	None	MIMIC III	N/A	100-1000	N/A	N/A	Low	Low	Low	High	High
**78**	Bashar	2020	United States	Discriminative AI	Diagnostic	Detect atrial fibrillation from premature atrial contraction and premature ventricular contraction	3&4	None	MIMIC III	N/A	100-1000	N/A	N/A	Low	Low	High	High	High
**79**	Bashar	2021	United States	Discriminative AI	Predicting complications	Predicting atrial fibrillation in critically ill sepsis patients	5	None	Other type of dataset	MIMIC III	0-100	0-100	N/A	Unclear	Low	Low	High	High
**80**	Bashar	2021	United States	Discriminative AI	Predicting complications	Predicting atrial fibrillation in intensive care unit patients	3&4	None	MIMIC III	N/A	1.000-10.000	N/A	N/A	Unclear	Low	High	High	High
**81**	Bashar	2021	United States	Discriminative AI	Predicting complications	Detect atrial fibrillation from premature atrial contraction and premature ventricular contraction	3&4	None	MIMIC III	N/A	0-100	N/A	N/A	Low	Low	High	High	High
**82**	Bataille	2021	France	Discriminative AI	Predicting health improvement	Detecting fluid responsiveness in critically ill patients after standardised fluid challenge	5	STARD/ STARD-AI	Other type of dataset	Other type of dataset	0-100	0-100	N/A	Low	Low	Low	Unclear	Unclear
**83**	Bednarski	2022	United States	Discriminative AI	Predicting mortality; Predicting length of stay	Predicting length of stay and mortality	3&4	None	MIMIC III	N/A	10.000-100.000	N/A	N/A	Low	Low	Low	High	High
**84**	Begum	2023	India	Discriminative AI	Predicting complications	Predicting acute kidney disease	3&4	None	MIMIC III	N/A	10.000-100.000	N/A	N/A	Low	Low	Unclear	Unclear	Unclear
**85**	Bendavid	2022	Israel	Discriminative AI	Predicting need for resource	Predicting onset of invasive mechanical ventilation	5	None	MIMIC III	Internal dataset	10.000-100.000	1.000 - 10.000	N/A	Low	Low	Unclear	Unclear	Unclear
**86**	Bendavid	2023	Israel	Discriminative AI	Improving communication	Assessing eye movements of ventilated patients admitted to the intensive care unit for communication	7	None	N/A	N/A	N/A	N/A	N/A	N/A	N/A	N/A	N/A	N/A
**87**	Benyo	2023	Hungary	Discriminative AI	Determining physiological values	Predicting the patient's insulin sensitivity parameter:	3&4	None	Internal dataset	N/A	100-1000	N/A	N/A	N/A	N/A	N/A	N/A	N/A
**88**	Berge	2023	Norway	Discriminative AI	Predicting complications	Identifying patient allergies	7	None	N/A	N/A	N/A	N/A	N/A	N/A	N/A	N/A	N/A	N/A
**89**	Bernabei	2021	United States	Discriminative AI	Predicting complications	Predicting epilepsy and seizures in critically ill patients	3&4	None	Internal dataset	N/A	0-100	N/A	N/A	Low	Low	High	Unclear	High
**90**	Bernard	2023	South Korea	Discriminative AI	Determining physiological values	Estimating blood pressure for patients admitted to the intensive care unit	3&4	None	MIMIC II	N/A	No information	N/A	N/A	Unclear	Low	Low	Unclear	Unclear
**91**	Bhaskhar	2023	United States	Discriminative AI	Predicting complications	Predicting acute kidney injury in the intensive care unit	5	None	Internal dataset	Internal dataset	1.000-10.000	100-1.000	N/A	Low	Low	Low	High	High
**92**	Bhattacharya	2017	India	Discriminative AI	Predicting mortality	Predicting mortality in intensive care unit patients	3&4	None	Other type of dataset	N/A	1.000-10.000	N/A	N/A	Low	Low	Low	Low	Low

© 2025 Berkhout WEM et al. *JAMA Network Open.*

**93**	Bhattacharyay	2022	United Kingdom	Discriminative AI	Predicting prognosis	Predicting Glasgow Outcome Scale-Extended score in intensive care unit patients	3&4	None	Other type of dataset	N/A	0-100	N/A	N/A	Low	Low	Low	High	High
**94**	Bhattacharyay	2023	United Kingdom	Discriminative AI	Predicting prognosis	Predicting Glasgow Outcome Scale - Extended for traumatic brain injury patients	3&4	None	Other type of dataset	N/A	1.000-10.000	N/A	N/A	Low	Low	Low	High	High
**95**	Bhattacharyya	2022	United States	Discriminative AI	Predicting complications	Predicting delirium	3&4	None	MIMIC III, eICU-CRD	N/A	10.000-100.000	N/A	N/A	Low	Low	Low	Low	Low
**96**	Bi	2022	China	Discriminative AI	Predicting mortality	Predicting mortality for post cardiovascular surgery patients	3&4	TRIPOD/ TRIPOD+AI	eICU-CRD	N/A	1.000-10.000	N/A	N/A	Low	Low	Low	High	High
**97**	Bignoumba	2023	Estonia	Discriminative AI	Predicting mortality	Predicting mortality	3&4	None	MIMIC III	N/A	10.000-100.000	N/A	N/A	Unclear	Low	Low	Low	Unclear
**98**	Bishara	2021	United States	Discriminative AI	Predicting prognosis	Predicting transplantation of a liver graft	3&4	None	Other type of dataset	N/A	10.000-100.000	N/A	N/A	Low	Low	Low	High	High
**99**	Blaivas	2020	United States	Discriminative AI	Predicting complications; Predicting medication administration; Assessing videos and images	Predicting fluid responsiveness in critically ill patients using AI to assess IVC collapsibility on POCUS.	3&4	None	Other type of dataset	N/A	100-1000	N/A	N/A	Low	Low	Low	Unclear	Low
**100**	Blaivas	2021	United States	Discriminative AI	Assessing videos and images	Analyzing collapsibility of inferior vena cava in critically ill patients	3&4	None	Other type of dataset	N/A	100-1000	N/A	N/A	Unclear	Low	High	Unclear	High
**101**	Bo	2021	China	Discriminative AI	Assessing videos and images	Image diagnosis methd f atients with cerebral infarction	3&4	None	Internal dataset	N/A	0-100	N/A	N/A	Unclear	Low	Low	High	High
**102**	Bock	2022	Austria	Discriminative AI (Reinforcement Learning)	Treatment recommendation	Treatment of sepsis	3&4	None	MIMIC III	N/A	10.000-100.000	N/A	N/A	N/A	N/A	N/A	N/A	N/A
**103**	Bodenes	2022	France	Discriminative AI	Predicting mortality	Predicting mortality for intensive care unit patients	3&4	None	Other type of dataset	N/A	100-1000	N/A	N/A	Unclear	Low	Low	High	High
**104**	Boie	2022	Germany	Discriminative AI	Determining physiological values	Predicting activated partial thromboplastin time after heparin treatment in intensive care unit patients	3&4	None	Internal dataset	N/A	1.000-10.000	N/A	N/A	Low	Low	High	Unclear	High
**105**	Bollepalli	2021	United States	Discriminative AI	Diagnostic	Detection of arrhythmia	3&4	None	Other type of dataset	N/A	100-1000	N/A	N/A	Unclear	Low	Low	Unclear	Unclear
**106**	Bologheanu	2023	Austria	Discriminative AI (Reinforcement Learning)	Provide dosage recommendations	Derive the optimal steroid policy in septic patients	3&4	None	Internal dataset	N/A	1.000-10.000	N/A	N/A	N/A	N/A	N/A	N/A	N/A
**107**	Bolton	2022	United Kingdom	Discriminative AI	Predicting mortality; Predicting length of stay; Treatment recommendation	Predicting patient outcomes under the contrasting scenarios of stopping or continuing antibiotic treatment	5	None	MIMIC IV	MIMIC IV	10.000-100.000	1.000 - 10.000	N/A	Unclear	Low	Low	High	High
**108**	Bolton	2024	United Kingdom	Discriminative AI	Predicting health improvement	Predicting if a patient can switch from IV to oral antibiotic treatment	5	None	MIMIC III	eICU-CRD	1.000-10.000	1.000 - 10.000	N/A	Unclear	Low	Low	Unclear	Unclear
**109**	Bossavi	2023	China	Discriminative AI	Determining physiological values	Calculating blood pressure	3&4	None	MIMIC II	N/A	0-100	N/A	N/A	Low	Low	Low	Unclear	Unclear
**110**	Boussen	2022	France	Discriminative AI	Predicting complications; Predicting length of stay; Prediction deterioration	Asses triage, dynamic intubation needs and length of stay of COVID-19 patients	3&4	None	Internal dataset	N/A	100-1000	N/A	N/A	Unclear	Low	Low	High	High
**111**	Boussen	2024	France	Discriminative AI	Predicting mortality	Predicting mortality in ICU patients	3&4	None	Internal dataset	N/A	1.000-10.000	N/A	N/A	N/A	N/A	N/A	N/A	N/A
**112**	Boutin	2023	France	Discriminative AI	Predicting mortality; Predicting complications	Predict mortality or AKI for patients with sepsis	3&4	None	Internal dataset	N/A	0-100	N/A	N/A	Low	Low	Low	High	High
**113**	Brancato	2023	Netherlands	Discriminative AI	Predicting complications	Predicting AKI	3&4	None	MIMIC III	N/A	10.000-100.000	N/A	N/A	N/A	N/A	N/A	N/A	N/A
**114**	Brokowski	2022	United States	Discriminative AI	Predicting health improvement	Predicting antibiotic activity and susceptibility	3&4	None	MIMIC III	N/A	1.000-10.000	N/A	N/A	Low	Unclear	Unclear	High	High

© 2025 Berkhout WEM et al. *JAMA Network Open.*

**115**	Brossard	2023	France	Discriminative AI	Predicting prognosis	Predicting therapeutic intensity level for severe traumatic brain injury	5	None	Internal dataset	Internal dataset	0-100	0-100	N/A	Unclear	Low	Low	Unclear	Unclear
**116**	Brown	2023	United States	Discriminative AI	Assessing videos and images	Checking endotracheal tube placement	3&4	None	Internal dataset	N/A	1.000-10.000	N/A	N/A	Low	Low	High	Unclear	High
**117**	Bucklin	2023	United States	Discriminative AI	Predicting complications; Improving prognostic models/risk scoring system; Detecting spurious recorded values	to investigate the prevalence, severity, and risk factors of sleep-disordered breathing in ICU patients using a machine learning algorithm to process respiratory and oximetry signals, with a specific focus on detecting apnea-hypopnea index (AHI) and estimating hypoxic burden and periodic breathing.	3&4	STROBE	Internal dataset	N/A	100-1000	N/A	N/A	Low	Low	Low	Low	Low
**118**	Buell	2024	United States	Discriminative AI	Predicting mortality	Predicting the effect of lower versus higher SpO2 targets on mortality	5	None	Other type of dataset	Internal dataset	1.000-10.000	100-1.000	N/A	Low	Unclear	Low	Low	Unclear
**119**	Bunney	2022	United States	Discriminative AI	Predicting complications	Predicting seizures after intracerebral hemorrhage	5	None	Internal dataset	Internal dataset	100-1000	100-1.000	N/A	Low	Low	Low	Unclear	Unclear
**120**	Busch	2023	Germany	Discriminative AI	Assessing videos and images	Automatic anatomy segmentation of bedside CXRs in intensive care unit patients	5	None	Internal dataset	Internal dataset	1.000-10.000	0-100	N/A	Unclear	Low	High	High	High
**121**	Cai	2022	China	Discriminative AI	Predicting complications	Predicting acute kidney injury in patient with acute myocardial infarction	5	None	MIMIC IV	MIMIC III	1.000-10.000	1.000 - 10.000	N/A	Low	Unclear	High	Low	High
**122**	Caicedo-Torres	2022	New Zealand	Discriminative AI	Predicting mortality	Predicting intensive care unit mortality	3&4	None	MIMIC III	N/A	10.000-100.000	N/A	N/A	Low	Low	Low	Unclear	Unclear
**123**	Callcut	2021	United States	Discriminative AI	Predicting complications	Predict respiratory failure in intensive care unit patients	5	None	N/A	Internal dataset	N/A	1.000 - 10.000	N/A	Unclear	Low	Low	Low	Unclear
**124**	Campbell	2024	United States	Discriminative AI	Classifying sub-populations	Defining mortality risk profiles for trauma patients receiving extracorporeal membrane oxygenation	3&4	STROBE	Other type of dataset	N/A	1.000-10.000	N/A	N/A	N/A	N/A	N/A	N/A	N/A
**125**	Cao	2023	China	Discriminative AI	Predicting complications	Predicting risk of invasive fungal infection	3&4	TRIPOD/ TRIPOD+AI	MIMIC IV	N/A	10.000-100.000	N/A	N/A	Low	Low	Low	High	High
**126**	Carey	2022	United Kingdom	Discriminative AI (Reinforcement learning)	Provide dosage recommendations	Providing optimal dosage for vasopressor and IV fluid given to sepsis patients in critical care	3&4	None	MIMIC III	N/A	No information	N/A	N/A	N/A	N/A	N/A	N/A	N/A
**127**	Carra	2021	Belgium	Discriminative AI	Predicting complications	Predicting elevated intracranial pressure in traumatic brain injury patients	5	None	N/A	Other type of dataset	N/A	100-1.000	N/A	Unclear	Unclear	High	Unclear	High
**128**	Carra	2023	Italy	Discriminative AI	Predicting complications	Predicting episodes of intracranial pressure in patients with severe traumatic brain injury	5	TRIPOD/ TRIPOD+AI	Other type of dataset	Other type of dataset	100-1000	100-1.000	N/A	Low	Low	High	Unclear	High
**129**	Carrasco-Gómez	2021	Spain	Discriminative AI	Predicting prognosis	Predicting outcome of postanoxic coma of patients admitted to the intensive care unit	3&4	None	Internal dataset	N/A	100-1000	N/A	N/A	Unclear	Low	Low	High	High
**130**	Carvalho	2022	Taiwan	Discriminative AI	Assessing videos and images	Predicting the severity of the CT lesion in COVID-19 patients at the intensive care unit	3&4	None	Internal dataset	N/A	1.000-10.000	N/A	N/A	Low	Low	Low	Low	Low
**131**	Carvalho	2023	Portugal	Discriminative AI	Predicting readmissions	Predicting ICU readmission within 30 days using knowledge graph embeddings derived from semantic annotations of EHR data.	3&4	None	MIMIC III	N/A	10.000-100.000	N/A	N/A	Low	Low	Low	Low	Low

© 2025 Berkhout WEM et al. *JAMA Network Open.*

**132**	Celada-Bernal	2023	Spain	Discriminative AI	Predicting physiological values	Predicting medical test values of COVID-19 intensive care unit patients	3&4	None	Internal dataset	N/A	100-1000	N/A	N/A	Unclear	Low	High	Unclear	High
**133**	Cena	2021	Italy	Discriminative AI	Predicting mortality; Predicting need for resource	Predicting mortality and intubation for intensive care unit patients	3&4	STROBE	Internal dataset	N/A	100-1000	N/A	N/A	Low	Low	Low	High	High
**134**	Chakraborty	2020	India	Discriminative AI	Determining physiological values	Estimating blood pressure for intensive care unit patients	3&4	None	MIMIC, MIMIC II, Other type of dataset	N/A	100-1000	N/A	N/A	Unclear	Low	Low	Unclear	Unclear
**135**	Chakraborty	2024	India	Discriminative AI	Determining physiological values	Blood pressure and heart rate measurement from PPG signal	3&4	None	MIMIC II	N/A	1.000-10.000	N/A	N/A	N/A	N/A	N/A	N/A	N/A
**136**	Chakraborty	2024	India	Discriminative AI	Predicting complications	Identification of patients at risk for sepsis	3&4	None	Other type of dataset	N/A	10.000-100.000	N/A	N/A	Unclear	Unclear	Low	High	High
**137**	Chamanzar	2023	United States	Discriminative AI	Predicting complications	to establish the feasibility and quantify the performance of an automated SD detection method, WAVEFRONT, using noninvasive scalp EEG in patients with severe TBI who underwent DHC	3&4	None	Internal dataset	N/A	100-1000	N/A	N/A	Low	Low	Low	Low	Low
**138**	Chan	2022	Taiwan	Discriminative AI	Predicting mortality	Predicting 30-day, 90-day and 1-year mortality in critically ill ventilated patients	3&4	None	Internal dataset	N/A	1.000-10.000	N/A	N/A	Unclear	Unclear	Low	Unclear	Unclear
**139**	Chandar	2022	India	Discriminative AI	Alarm reduction	Lowering false alarms	3&4	None	MIMIC II	N/A	1.000-10.000	N/A	N/A	Unclear	Low	Low	High	High
**140**	Chang	2019	United States	Discriminative AI	Predicting complications	Predicting abnormal values for vital signs in intensive care unit patients	3&4	None	MIMIC III	N/A	No information	N/A	N/A	Unclear	Unclear	Unclear	High	High
**141**	Chang	2022	Taiwan	Discriminative AI	Predicting mortality	Predicting mortality for patients receiving renal replacement therapy for AKI	5	TRIPOD/ TRIPOD+AI	MIMIC III	eICU-CRD	1.000-10.000	1.000 - 10.000	N/A	Low	Low	Low	Low	Low
**142**	Chang	2024	United States	Discriminative AI	Determining physiological values	Forecasting heart rate, systolic blood pressure, and diastolic blood pressure in the intensive care unit	3&4	None	MIMIC III	N/A	10.000-100.000	N/A	N/A	Low	Low	High	Unclear	High
**143**	Cheema	2021	United States	Discriminative AI	Assessing videos and images	Providing guidance for obtaining point-of-care ultrasound cardiac images	7	None	N/A	N/A	N/A	N/A	N/A	N/A	N/A	N/A	N/A	N/A
**144**	Chen	2020	Australia	Discriminative AI	Predicting prognosis	Predicting illness severity of patients in the intensive care unit	3&4	None	MIMIC III	N/A	10.000-100.000	N/A	N/A	Low	Low	Low	Unclear	Unclear
**145**	Chen	2020	China	Discriminative AI	Predicting complications	Warning of citric acid overdose for patients with renal replacement therapy	5	None	Other type of dataset	Other type of dataset	1.000-10.000	100-1.000	N/A	Low	Low	High	High	High
**146**	Chen	2021	China	Discriminative AI	Predicting complications	Risk prediction of acute kidney injury	3&4	None	MIMIC III	N/A	10.000-100.000	N/A	N/A	Unclear	Unclear	Low	High	High
**147**	Chen	2021	China	Discriminative AI	Predicting mortality	Predicting mortality in COVID-19 patients admitted to the intensive care unit	3&4	TRIPOD/ TRIPOD+AI	Internal dataset	N/A	0-100	N/A	N/A	Unclear	Unclear	Low	Unclear	Unclear
**148**	Chen	2022	Taiwan	Discriminative AI	Improving mechanical ventilation	Predicting weaning time	3&4	None	Internal dataset	N/A	1.000-10.000	N/A	N/A	Low	Low	Low	Unclear	Unclear
**149**	Chen	2022	China	Discriminative AI	Improving mechanical ventilation	Detecting patient-ventilator asynchrony	3&4	None	Other type of dataset	N/A	0-100	N/A	N/A	Unclear	Low	High	Low	High
**150**	Chen	2022	Taiwan	Generative AI (Large language model)	Predicting length of stay	Predicting prolonged length of stay in the ICU	3&4	None	MIMIC IV	N/A	10.000-100.000	N/A	N/A	Unclear	Unclear	Low	High	High
**151**	Chen	2022	China	Discriminative AI	Predicting mortality; Predicting complications; Improving prognostic models/risk scoring system	Developing an autophagy-related genes classifier to diagnose sepsis, predict mortality, and evaluate the immune microenvironment in	3&4	None	Other type of dataset	N/A	No information	N/A	N/A	Unclear	Low	Low	Unclear	Unclear

© 2025 Berkhout WEM et al. *JAMA Network Open.*

						septic patients within ICU settings												
**152**	Chen	2022	China	Discriminative AI	Predicting mortality; Predicting complications	The study aims to develop and validate a real-time prediction model using Dynamic Bayesian Networks to predict physiological changes, organ dysfunctions, and mortality risk in critical trauma patients admitted to the ICU.	5	None	MIMIC III	Internal dataset	1.000-10.000	1.000 - 10.000	N/A	Low	Low	Low	Unclear	Unclear
**153**	Chen	2022	China	Discriminative AI	Predicting mortality	Predicting in-hospital mortality in intensive care unit patients	3&4	None	MIMIC III	N/A	1.000-10.000	N/A	N/A	Low	Low	Low	High	High
**154**	Chen	2022	Canada	Discriminative AI	Predicting complications	to detect atrial fibrillation (AF) in ICU patients using ECG telemetry data	3&4	None	Internal dataset	N/A	10.000-100.000	N/A	N/A	Low	Low	High	Unclear	High
**155**	Chen	2022	China	Discriminative AI	Predicting complications	Predicting sepsis	3&4	None	Other type of dataset	N/A	10.000-100.000	N/A	N/A	Unclear	Unclear	Unclear	High	High
**156**	Chen	2022	China	Discriminative AI	Predicting complications	Predicting sepsis within 5 hours preceding onset in ICU patients	5	TRIPOD/ TRIPOD+AI	MIMIC III	Internal dataset	1.000-10.000	0-100	N/A	Unclear	Low	Low	Unclear	Unclear
**157**	Chen	2023	United States	Discriminative AI	Predicting mortality; Predicting length of stay	Predicting length of stay and mortality in the intensive care unit	3&4	None	MIMIC IV	N/A	100.000+	N/A	N/A	Low	Low	Low	Low	Low
**158**	Chen	2023	Canada	Discriminative AI	Predicting complications	Predicting Atrial Fibrillation in intensive care unit patients	5	None	Other type of dataset	Internal dataset	10.000-100.000	100-1.000	N/A	Low	Low	Low	Low	Low
**159**	Chen	2023	China	Discriminative AI	Predicting complications	Predicting bleeding risk in intensive care unit patients treated with anti-thrombotic therapy	3&4	None	MIMIC III	N/A	10.000-100.000	N/A	N/A	Low	Low	Low	Low	Low
**160**	Chen	2023	Germany	Discriminative AI	Predicting mortality	Predicting mortality risk for critically ill COVID-19 patients in the ICU	3&4	None	Internal dataset	N/A	100-1000	N/A	N/A	Low	Low	Low	Low	Low
**161**	Chen	2023	China	Discriminative AI	Predicting complications	Predicting sepsis after liver transplantation	5	TRIPOD/ TRIPOD+AI	Internal dataset	Internal dataset	100-1000	100-1.000	N/A	Unclear	Unclear	Low	High	High
**162**	Chen	2023	China	Discriminative AI	Predicting mortality	Predicting in-hospital mortality for intensive care unit patients with heart failure	5	None	MIMIC IV	eICU-CRD	10.000-100.000	10.000 - 100.000	N/A	Low	Low	High	High	High
**163**	Chen	2024	China	Discriminative AI	Predicting complications	Predicting cerebral hemorrhage, heart failure, renal failure, pancreatitis, respiratory failure and ketoacidosis	3&4	None	MIMIC IV	N/A	100-1000	N/A	N/A	Low	Low	Low	Low	Low
**164**	Chen	2024	United States	Discriminative AI	Predicting length of stay	Predicting time until discharge from the intensive care unit for patients with intracerebral hemorrhage	3&4	None	MIMIC III	N/A	100-1000	N/A	N/A	Unclear	Low	High	Unclear	High
**165**	Chen	2024	China	Discriminative AI	Assessing videos and images	Predicting unplanned extubation in intensive care unit patients	3&4	None	Internal dataset	N/A	100-1000	N/A	N/A	Low	Low	Low	Low	Low
**166**	Cheng	2019	United States	Discriminative AI (Reinforcement learning)	Treatment recommendation	Recommending ordering lab tests	3&4	None	MIMIC III	N/A	1.000-10.000	N/A	N/A	Unclear	Low	Low	High	High
**167**	Cheng	2021	China	Discriminative AI	Determining physiological values	Predicting blood pressure waveforms for intensive care unit patients	3&4	None	MIMIC II	N/A	100.000+	N/A	N/A	Unclear	Low	Low	Unclear	Unclear
**168**	Cheng	2021	China	Discriminative AI	Predicting complications	Predicting patients that are at risk of hospital acquired thrombocytopenia after surgery	3&4	None	Internal dataset	N/A	10.000-100.000	N/A	N/A	Low	Unclear	High	Unclear	High
**169**	Cheng	2022	China	Discriminative AI	Predicting mortality	Predicting mortality for COVID-19 patients	3&4	None	Internal dataset	N/A	100-1000	N/A	N/A	N/A	N/A	N/A	N/A	N/A

© 2025 Berkhout WEM et al. *JAMA Network Open.*

**170**	Cheng	2022	China	Discriminative AI	Predicting complications	Predicting acute kidney injury in patients with acute pancreatitis admitted to the intensive care unit	3&4	None	Internal dataset	N/A	100-1000	N/A	N/A	Low	Unclear	Low	Unclear	Unclear
**171**	Cheng	2022	Taiwan	Discriminative AI	Improving mechanical ventilation	Predicting successful ventilator mode shifting for patients with mechanical ventilators for acute respiratory failure	3&4	None	Internal dataset	N/A	1.000-10.000	N/A	N/A	Low	Low	High	Unclear	High
**172**	Cheng	2023	Taiwan	Discriminative AI	Predicting mortality	Predicting 30-day mortality at sepsis diagnosis time in critically ill patients	3&4	TRIPOD/ TRIPOD+AI	MIMIC IV	N/A	10.000-100.000	N/A	N/A	Unclear	Unclear	Low	High	High
**173**	Cheng	2024	China	Discriminative AI	Predicting complications	Predicting risk of status epilepticus in intensive care unit patients	3&4	None	eICU-CRD	N/A	1.000-10.000	N/A	N/A	Low	Unclear	Low	High	High
**174**	Cherifa	2021	United States	Discriminative AI	Determining physiological values	Predicting mean arterial pressure and heart rate in critically ill patients	5	None	MIMIC III	Internal dataset	1.000-10.000	0-100	N/A	Unclear	Low	High	High	High
**175**	Chia	2021	Singapore	Discriminative AI	Predicting mortality	Predicting in-hospital mortality in the intensive care unit	3&4	None	Other type of dataset	N/A	10.000-100.000	N/A	N/A	Unclear	Low	Low	High	High
**176**	Chimbunde	2023	South Africa	Discriminative AI	Predicting mortality	Predicting COVID-19 mortality in intensive care unit patients	3&4	None	Internal dataset	N/A	100-1000	N/A	N/A	Unclear	Unclear	Low	High	High
**177**	Chiu	2022	Taiwan	Discriminative AI	Predicting mortality	Predicting mortality of heart failure patients	3&4	None	MIMIC III	N/A	1.000-10.000	N/A	N/A	Low	Low	Low	Low	Low
**178**	Chiu	2022	Taiwan	Discriminative AI	Predicting mortality	Predicting mortality of intensive care unit patients	3&4	None	MIMIC III	N/A	10.000-100.000	N/A	N/A	Low	Low	Low	High	Unclear
**179**	Chiu	2022	Taiwan	Discriminative AI	Predicting complications	Detecting hyperkalemia in intensive care unit patients	3&4	None	MIMIC III	N/A	1.000-10.000	N/A	N/A	Low	Low	Low	Low	Low
**180**	Chiu	2023	Taiwan	Discriminative AI	Predicting mortality	Predicting mortality for ICU patients by integrating structured and unstructured EHR data	3&4	None	MIMIC III	N/A	10.000-100.000	N/A	N/A	Low	Low	Low	Unclear	Unclear
**181**	Chiu	2023	Taiwan	Discriminative AI	Predicting complications	to develop a personalized machine-learning model to predict dysglycemia (both hyperglycemia and hypoglycemia) from ECG data	3&4	None	MIMIC III	N/A	0-100	N/A	N/A	Unclear	Low	Low	Unclear	Unclear
**182**	Cho	2023	Republic of Korea	Discriminative AI	Determining physiological values	Estimating blood pressure in intensive care unit patients	3&4	None	MIMIC	N/A	100.000+	N/A	N/A	Unclear	Low	Low	High	High
**183**	Choi	2020	South Korea	Discriminative AI	Predicting complications	Predicting oral-mucosal pressure injuries in intubated patients in the intensive care unit	3&4	None	Internal dataset	N/A	0-100	N/A	N/A	Low	Unclear	Low	High	High
**184**	Choi	2022	South Korea	Discriminative AI	Predicting mortality	Predicting mortality in intensive care unit patients	5	None	Internal dataset	Internal dataset	10.000-100.000	10.000 - 100.000	N/A	Low	Low	Low	High	High
**185**	Choi	2023	South Korea	Discriminative AI	Predicting complications	Predicting acute kidney injury in trauma patients	3&4	None	Internal dataset	N/A	100-1000	N/A	N/A	Low	Low	High	Low	High
**186**	Choi	2023	Korea	Discriminative AI	Provide dosage recommendations	Predicting warfarin discharge dosage	5	None	Internal dataset	MIMIC III	1.000-10.000	100-1.000	N/A	Low	Unclear	Low	Low	Unclear
**187**	Choi	2024	South Korea	Discriminative AI	Predicting mortality; Predicting length of stay	Predicting mortality, length of ICU stay and length of hospital stay in sepsis patients in the intensive care unit	3&4	None	Internal dataset	N/A	100-1000	N/A	N/A	Low	Low	Low	High	High
**188**	Chou	2022	Taiwan	Discriminative AI	Improving prognostic models/risk scoring system	Cardiac arrest survival post-resuscitation in-hospital score	3&4	None	Other type of dataset	N/A	100-1000	N/A	N/A	Low	Unclear	Low	Unclear	Unclear
**189**	Chou	2024	Taiwan	Discriminative AI	Predicting mortality	Predicting mortality of critically ill patients	3&4	None	Internal dataset	N/A	10.000-100.000	N/A	N/A	Low	Low	High	High	High
**190**	Chu	2023	United States	Discriminative AI	Determining physiological values	Estimating arterial systolic blood pressure (ASBP),	3&4	None	MIMIC III	N/A	1.000-10.000	N/A	N/A	Low	Low	Low	High	High

© 2025 Berkhout WEM et al. *JAMA Network Open.*

						arterial diastolic blood pressure (ADBP), and oxygen saturation (SpO2) using PPG signals in ICU patients												
**191**	Churpek	2021	United States	Discriminative AI	Predicting mortality; Improving prognostic models/risk scoring system	Developing and validating machine learning models, specifically eXtreme Gradient Boosting, to predict 28-day mortality in ICU patients with COVID-19	5	None	Internal dataset	Internal dataset	1.000-10.000	100-1.000	N/A	Low	Low	Low	Low	Low
**192**	Clare	2022	United States	Discriminative AI	Predicting complications	Detecting vasospasm via cerebral blood flow velocities in subarachnoid hemorrhage patients in a Neuro ICU setting	8	None	N/A	N/A	N/A	N/A	12	N/A	N/A	N/A	N/A	N/A
**193**	Clark	2023	United States	Discriminative AI	Predicting complications; Improving prognostic models/risk scoring system	Predicting the risk of discretionary prolonged postoperative mechanical ventilation (DPPMV) for adult patients undergoing surgery with general anesthesia and endotracheal intubation	3&4	TRIPOD/ TRIPOD+AI	Internal dataset	N/A	10.000-100.000	N/A	N/A	Low	Low	Low	Low	Low
**194**	Cleri	2023	United States	Discriminative AI	Predicting mortality; Improving prognostic models/risk scoring system	Predicting in-hospital survival and recovery of command following for severe traumatic brain injury patients	3&4	None	Internal dataset	N/A	100-1000	N/A	N/A	Low	Low	Low	Low	Low
**195**	Cohen	2021	Israel	Discriminative AI	Predicting mortality; Improving prognostic models/risk scoring system	Predicting ICU mortality for adult ICU patients	3&4	None	Other type of dataset	N/A	100.000+	N/A	N/A	Low	Low	Low	Low	Low
**196**	Cohen	2024	United States	Discriminative AI	Predicting complications	Predicting sepsis onset for intensive care unit patients	3&4	RECORD	MIMIC III	N/A	10.000-100.000	N/A	N/A	Low	Low	Low	Low	Low
**197**	Coombes	2021	United States	Discriminative AI	Predicting complications	Predicting delirium in the intensive care unit	3&4	TRIPOD/ TRIPOD+AI	MIMIC III	N/A	10.000-100.000	N/A	N/A	Low	Low	Low	High	High
**198**	Coombes	2022	United States	Discriminative AI	Predicting complications	Predicting the onset of infection for critically ill patients in the ICU using sequences of clinical events from electronic health records	3&4	None	MIMIC III	N/A	10.000-100.000	N/A	N/A	Low	Low	Low	High	High
**199**	Crespo-Diaz	2024	United States	Discriminative AI	Predicting prognosis	Predicting neurologic outcomes of extracorporeal cardiopulmonary resuscitation in patients at the cardiac intensive care unit	3&4	None	Internal dataset	N/A	100-1000	N/A	N/A	Low	Low	Low	High	High
**200**	Cuadrado	2023	United States	Discriminative AI	Predicting length of stay	Predicting discharge date for ICU patients	3&4	None	eICU-CRD	N/A	10.000-100.000	N/A	N/A	Low	Low	Low	Unclear	Unclear
**201**	Cui	2021	China	Discriminative AI	Predicting mortality	Predicting 1-year mortality in traumatic brain injury patients undergoing decompressive craniectomy	3&4	None	Internal dataset	N/A	100-1000	N/A	N/A	Low	Low	Low	High	High
**202**	Cui	2024	China	Discriminative AI	Predicting complications	Predicting the incidence of antibiotic-associated diarrhea (AAD) in elderly ICU patients	3&4	TRIPOD/ TRIPOD+AI	Internal dataset	N/A	100-1000	N/A	N/A	Low	Low	Low	Low	Low
**203**	Cysneiros	2024	Portugal	Discriminative AI	Predicting mortality	Predicting all-cause mortality of SARS-CoV-2-associated acute respiratory distress syndrome	3&4	None	Internal dataset	N/A	100-1000	N/A	N/A	Low	Low	Low	Unclear	Unclear

© 2025 Berkhout WEM et al. *JAMA Network Open.*

**204**	Daenen	2023	Netherlands	Discriminative AI	Predicting mortality	Predicting mortality in COVID-19 patients in the intensive care unit	5	None	N/A	Internal dataset	N/A	0-100	N/A	Low	Low	Low	High	High
**205**	Dai	2023	Taiwan	Discriminative AI	Predicting complications	Predicting sepsis in the intensive care unit	3&4	None	Other type of dataset	N/A	10.000-100.000	N/A	N/A	Unclear	Unclear	High	Low	High
**206**	Dai	2024	Taiwan	Discriminative AI	Assessing videos and images	Assessing sedation status	3&4	None	Internal dataset	N/A	1.000-10.000	N/A	N/A	Low	Low	Low	Unclear	Unclear
**207**	Dala Ali	2023	Saudi Arabia	Discriminative AI	Predicting medication administration; Improving prognostic models/risk scoring system	Predicting inadequate empiric antimicrobial therapy for ICU sepsis patients and comparing performance of artificial neural network vs logistic regression models	3&4	None	Internal dataset	N/A	100-1000	N/A	N/A	Low	Low	Low	High	High
**208**	Dam	2022	Netherlands	Discriminative AI	Predicting health improvement	Predicting success of prone positioning after 4 hours based on improvements in PaO2/FiO2 ratio, ventilatory ratio, respiratory system compliance, or mechanical power in mechanically ventilated critically ill COVID-19 patients	3&4	None	Other type of dataset	N/A	1.000-10.000	N/A	N/A	Low	Low	Low	High	High
**209**	Danilatou	2022	United States	Discriminative AI	Predicting mortality; Improving prognostic models/risk scoring system	Predicting early and late mortality for critically ill patients with venous thromboembolism and/or cancer using machine learning models and comparing their performance to traditional clinical scoring systems.	5	None	MIMIC III	eICU-CRD	1.000-10.000	1.000 - 10.000	N/A	Low	Low	Low	Low	Low
**210**	Danilovich	2021	Russia	Discriminative AI	Assessing videos and images	Detecting patient positions for patients admitted to the intensive care unit	3&4	None	Internal dataset	N/A	100.000+	N/A	N/A	Unclear	Low	High	Unclear	High
**211**	Darabi	2020	United States	Discriminative AI	Predicting mortality; Predicting readmissions; Predicting length of stay	Predicting mortality, readmission and length of stay of intensive care unit patients	3&4	None	MIMIC III	N/A	No information	N/A	N/A	Unclear	Low	Low	Unclear	Unclear
**212**	Das	2024	Germany	Discriminative AI	Predicting complications	Predicting early onset of sepsis in intensive care unit patients	3&4	None	Other type of dataset	N/A	10.000-100.000	N/A	N/A	Low	Low	High	Unclear	High
**213**	Dave	2023	Canada	Discriminative AI	Assessing videos and images	Distinguishing between normal and abnormal lung parenchyma on lung ultrasound in critically ill patients	6	STROBE	N/A	N/A	N/A	N/A	N/A	N/A	N/A	N/A	N/A	N/A
**214**	de Haro	2024	Canada	Discriminative AI	Improving mechanical ventilation	Developing and validating supervised artificial intelligence algorithms (convolutional neural network and recurrent neural network) for identifying and classifying airway pressure deformation patterns during square-flow assisted ventilation in critically ill adult patients under mechanical ventilation	3&4	None	Internal dataset	N/A	0-100	N/A	N/A	Unclear	Low	Low	Unclear	Unclear
**215**	de Hond	2023	Netherlands	Discriminative AI	Predicting mortality; Predicting readmissions	Predicting readmission or death within 7 days after ICU discharge for adult patients admitted to the ICU for longer than 12 hours	5	TRIPOD/ TRIPOD+AI	Internal dataset	Internal dataset	10.000-100.000	10.000 - 100.000	N/A	Low	Low	Low	Low	Low
**216**	de Jonge	2022	Netherlands	Discriminative AI	Improving prognostic models/risk	Predicting change in quality of life 1 year	3&4	None	Internal dataset	N/A	1.000-10.000	N/A	N/A	Low	Low	Low	Low	Low

© 2025 Berkhout WEM et al. *JAMA Network Open.*

					scoring system; Predicting health improvement	after ICU admission for adult ICU survivors												
**217**	de Oliveira Gomes	2023	Brazil	Discriminative AI	Predicting mortality	Predicting mortality after myocardial injury	3&4	None	Internal dataset	N/A	1.000-10.000	N/A	N/A	Low	Low	Low	Low	Low
**218**	De Silva	2021	Australia	Discriminative AI	Predicting mortality	Predicting 1-year all-cause mortality for patients with diabetes mellitus following critical care	3&4	None	MIMIC III	N/A	100.000+	N/A	N/A	Low	Low	Low	Low	Low
**219**	Deasy	2019	United Kingdom	Discriminative AI	Predicting mortality	Predicting mortality in intensive care unit patients	3&4	None	MIMIC III	N/A	No information	N/A	N/A	Low	Low	Low	High	High
**220**	Deasy	2020	United Kingdom	Discriminative AI	Predicting mortality; Improving prognostic models/risk scoring system	Predicting in-hospital mortality for critically ill patients in the ICU using uncurated patient data from the MIMIC-III database to make dynamic predictions throughout a patient's ICU stay	3&4	None	MIMIC III	N/A	10.000-100.000	N/A	N/A	Low	Low	Low	High	High
**221**	Delgado	2021	Spain	Discriminative AI	Predicting mortality; Predicting prognosis, Diagnostic	Predicting mortality at the intensive care unit, destination at ICU discharge and cause of death	3&4	None	Internal dataset	N/A	1.000-10.000	N/A	N/A	Unclear	Unclear	Low	Unclear	Unclear
**222**	Den Hengst	2024	Netherlands	Discriminative AI (Reinforcement Learning)	Improving mechanical ventilation	Optimization of settings in invasive mechanical ventilation	3&4	None	MIMIC III	N/A	1.000-10.000	N/A	N/A	Low	Unclear	Low	Unclear	Unclear
**223**	Deng	2022	China	Discriminative AI	Predicting mortality; Predicting readmissions; Predicting length of stay	Predicting mortality, prolonged length of stay and 30-day readmission in intensive care unit patients	3&4	None	MIMIC IV	N/A	10.000-100.000	N/A	N/A	N/A	N/A	N/A	N/A	N/A
**224**	Deng	2023	Malaysia	Discriminative AI	Predicting mortality	Predicting in-hospital mortality	3&4	None	eICU-CRD	N/A	10.000-100.000	N/A	N/A	Low	Low	Low	High	High
**225**	Deng	2024	China	Discriminative AI	Predicting mortality	Predicting mortality for critically ill patients with upper gastrointestinal bleeding	5	None	MIMIC IV	eICU-CRD	1.000-10.000	100-1.000	N/A	N/A	N/A	N/A	N/A	N/A
**226**	Dhamala	2019	United States	Discriminative AI	Predicting complications	Predicting acute hypotensive episodes in intensive care unit patients	3&4	None	eICU-CRD	N/A	10.000-100.000	N/A	N/A	Unclear	Unclear	Unclear	High	High
**227**	Ding	2021	China	Discriminative AI	Predicting mortality	Predicting in-hospital mortality in acute pancreatitis	3&4	None	AmsterdamUMCdb	N/A	100-1000	N/A	N/A	Low	Low	Low	High	High
**228**	Ding	2021	United States	Discriminative AI	Classifying sub-populations	Identifying phenotypes of sepsis in intensive care unit	3&4	None	MIMIC III	N/A	1.000-10.000	N/A	N/A	Low	Unclear	High	High	High
**229**	Ding	2023	United States	Discriminative AI	Predicting complications	Detecting atrial fibrillation	5	None	Internal dataset	Internal dataset, Other type of dataset, MIMIC III	100.000+	No information; 1.000 - 10.000	N/A	N/A	N/A	N/A	N/A	N/A
**230**	Dong	2024	China	Discriminative AI	Predicting mortality	Predicting risk of death in sepsis patients with acute kidney injury at the intensive care unit	5	TRIPOD/ TRIPOD+AI	MIMIC IV	Internal dataset, MIMIC III	1.000-10.000	1.000 - 10.000	N/A	Low	Unclear	Low	Unclear	Unclear
**231**	Du	2021	Singapore	Discriminative AI	Predicting mortality	Predicting in-hospital mortality of clostriodiodes difficile infection in intensive care unit patients	3&4	None	MIMIC III	N/A	1.000-10.000	N/A	N/A	Low	Low	Low	High	High
**232**	Du	2021	Singapore	Discriminative AI	Predicting complications	Predicting acute kidney injury in critical care	3&4	None	MIMIC III	N/A	No information	N/A	N/A	Unclear	Unclear	Unclear	Unclear	Unclear
**233**	Duanmu	2022	United States	Discriminative AI	Predicting mortality; Predicting need for resource	Predicting in-hospital mortality and duration of invasive mechanical ventilation for COVID-19 patients	3&4	STROBE	No information	N/A	100-1000	N/A	N/A	Unclear	Unclear	Low	Unclear	Unclear
**234**	Dung-Hung	2022	Taiwan	Discriminative AI	Predicting complications	Predicting onset of hemodynamic instability in critical care	5	None	N/A	Internal dataset	N/A	10.000 - 100.000	N/A	Low	Low	Low	Low	Low

© 2025 Berkhout WEM et al. *JAMA Network Open.*

**235**	Dupont	2024	France	Discriminative AI	Diagnostic	Predicting post-traumatic stress disorder in family members of patients admitted to the intensive care unit	3&4	TRIPOD/ TRIPOD+AI	Other type of dataset	N/A	1.000-10.000	N/A	N/A	Low	Low	Low	High	High
**236**	Edinburgh	2021	United Kingdom	Discriminative AI	Alarm reduction	Detecting artefacts in arterial blood pressure measurements at the intensive care unit	3&4	None	Internal dataset	N/A	10.000-100.000	N/A	N/A	Unclear	Low	High	Low	High
**237**	Eghbali	2021	United Kingdom	Discriminative AI (Reinforcement Learning)	Provide dosage recommendations	Framework for sedative-hypnotics dosing	3&4	None	MIMIC IV	N/A	1.000-10.000	N/A	N/A	N/A	N/A	N/A	N/A	N/A
**238**	Eickelberg	2020	United States	Discriminative AI	Predicting complications	Predicting bacterial infection risk in intensive care unit patients	3&4	None	MIMIC III	N/A	10.000-100.000	N/A	N/A	Low	Low	Low	Low	Low
**239**	Eickelberg	2023	United States	Discriminative AI	Predicting complications	Predicting bacterial infection for critically ill patients	5	TRIPOD/ TRIPOD+AI	Other type of dataset, MIMIC III	Other type of dataset	10.000-100.000	1.000 - 10.000	N/A	Low	Unclear	Low	Unclear	Unclear
**240**	Eini-Porat	2024	Israel	Discriminative AI	Determining physiological values	Predicting vital sign trajectories	3&4	None	MIMIC III	N/A	100-1000	N/A	N/A	Unclear	Low	High	Unclear	High
**241**	Elaanba	2023	Morocco	Discriminative AI	Assessing videos and images	Detecting tube errors on Chest-X-ray	3&4	None	Other type of dataset	N/A	10.000-100.000	N/A	N/A	Low	Low	Low	High	High
**242**	El-ganainy	2020	Norway	Discriminative AI	Determining physiological values	Predicting the mean arterial pressure	3&4	None	Internal dataset	N/A	0-100	N/A	N/A	Low	Low	High	High	High
**243**	El-Hajj	2021	United Kingdom	Discriminative AI	Determining physiological values	Predicting blood pressure for patients at the intensive care unit	3&4	None	MIMIC II	N/A	No information	N/A	N/A	Unclear	Low	Low	High	High
**244**	El-Hajj	2021	United Kingdom	Discriminative AI	Determining physiological values	Estimating systolic and diastolic blood pressure for intensive care unit patients	3&4	None	MIMIC II	N/A	No information	N/A	N/A	Unclear	Low	Low	Unclear	Unclear
**245**	Elhazmi	2022	Saudi Arabia	Discriminative AI	Predicting mortality	Predicting mortality in critically ill COVID-19 patients admitted to the intensive care unit	3&4	None	Internal dataset	N/A	1.000-10.000	N/A	N/A	Low	Low	Unclear	Unclear	Unclear
**246**	El-Manzalawy	2021	United States	Discriminative AI	Predicting mortality	Predicting in-hospital mortality for adult patients admitted to the intensive care unit	3&4	None	MIMIC III	N/A	10.000-100.000	N/A	N/A	Unclear	Low	Low	Unclear	Unclear
**247**	Elmer	2023	United States	Discriminative AI	Predicting health improvement	Predicting time to awakening in comatose adults resuscitated from cardiac arrest	3&4	TRIPOD/ TRIPOD+AI	Internal dataset	N/A	1.000-10.000	N/A	N/A	Low	Unclear	Low	High	High
**248**	El-Rashidy	2020	Egypt	Discriminative AI	Predicting mortality	Predicting mortality at the intensive care unit	3&4	None	MIMIC III	N/A	10.000-100.000	N/A	N/A	Low	Low	Low	High	High
**249**	El-Rashidy	2022	Egypt	Discriminative AI	Predicting complications	Predicting sepsis in the intensive care unit	3&4	None	MIMIC III	N/A	1.000-10.000	N/A	N/A	Low	Low	Low	Low	Low
**250**	El-Rashidy	2023	Egypt	Discriminative AI	Predicting complications	Predicting gestational diabetes mellitus in pregnant patients admitted to the intensive care unit	3&4	None	MIMIC III	N/A	10.000-100.000	N/A	N/A	Low	Unclear	Low	High	High
**251**	Eltaybani	2023	Japan	Generative AI (Large language model)	Improving research	Enhancing research	5	None	N/A	Other type of dataset	N/A	0-100	N/A	N/A	N/A	N/A	N/A	N/A
**252**	Embad	2023	United States	Discriminative AI	Predicting mortality	Predicting mortality of hemorrhagic stroke patients	3&4	MINIMAR standard	MIMIC III	N/A	100-1000	N/A	N/A	Low	Low	Low	Unclear	Unclear
**253**	Eskandari	2023	Iran	Discriminative AI	Predicting complications	Predicting sepsis in intensive care unit patients	3&4	None	Other type of dataset	N/A	10.000-100.000	N/A	N/A	Unclear	Unclear	Unclear	High	High
**254**	Essay	2023	United States	Discriminative AI	Improving mechanical ventilation	Predicting failure of noninvasive respiratory support in intensive care unit patients	3&4	TRIPOD/ TRIPOD+AI	Internal dataset	N/A	10.000-100.000	N/A	N/A	Low	Low	Low	Unclear	Unclear
**255**	Fabregat	2021	Spain	Discriminative AI	Improving mechanical ventilation	Predicting extubation outcome in critical patients with invasive mechanical ventilation	3&4	None	Internal dataset	N/A	1.000-10.000	N/A	N/A	Low	Unclear	Low	High	High
**256**	Fachet	2023	Germany	Discriminative AI	Predicting complications	Predicting complication patterns in polytrauma patients	3&4	STROBE	Other type of dataset	N/A	100-1000	N/A	N/A	Low	Low	Low	Unclear	Unclear

© 2025 Berkhout WEM et al. *JAMA Network Open.*

**257**	Faghihpirayesh	2022	United States	Discriminative AI	Predicting complications	Automatic detection of EEG Epileptiform Abnormalities for TBI patients	3&4	None	Other type of dataset	N/A	1.000-10.000	N/A	N/A	Unclear	Low	Low	Unclear	Unclear
**258**	Fahmi	2020	Indonesia	Discriminative AI	Assessing videos and images	Automatic detection of a brain tumor	3&4	None	Internal dataset	N/A	0-100	N/A	N/A	Unclear	Low	Low	High	High
**259**	Falter	2023	Belgium	Discriminative AI	Predicting complications	Investigating methods for automatic classi-fication of disease in unstructured medical records using NLP and to compare these to conventional ICD coding.	3&4	None	eICU-CRD, MIMIC III	N/A	10.000-100.000	N/A	N/A	N/A	N/A	N/A	N/A	N/A
**260**	Fan	2021	China	Discriminative AI	Determining physiological values	Estimating continuous blood pressure	3&4	None	MIMIC II	N/A	10.000-100.000	N/A	N/A	Unclear	Low	High	Low	High
**261**	Fan	2022	China	Discriminative AI	Predicting prognosis	Predicting prolonged intensive care unit stay in critical patients with spinal cord injury	3&4	None	eICU-CRD, MIMIC III, MIMIC IV	N/A	1.000-10.000	N/A	N/A	Unclear	Low	Low	Unclear	Unclear
**262**	Fan	2023	China	Discriminative AI	Predicting mortality	Predicting mortality in sepsis-associated acute kidney injury patients admitted to the intensive care unit	5	None	MIMIC IV	Internal dataset	1.000-10.000	0-100	N/A	Low	Low	Low	High	High
**263**	Fan	2023	China	Discriminative AI	Predicting prognosis	Predicting discharge destination of spinal cord injury patients in the intensive care unit	3&4	None	eICU-CRD, MIMIC III	N/A	1.000-10.000	N/A	N/A	Low	Unclear	Unclear	Unclear	Unclear
**264**	Fan	2023	China	Discriminative AI	Predicting complications	Predicting acute kidney injury for patients with diabetic ketoacidosis in the intensive care unit	3&4	None	MIMIC IV	N/A	1.000-10.000	N/A	N/A	Unclear	Low	Low	High	High
**265**	Faris Ali	2023	United Arab Emirates	Discriminative AI	Determining physiological values	Estimating blood pressure	3&4	None	MIMIC II	N/A	0-100	N/A	N/A	Unclear	Low	Low	Unclear	Unclear
**266**	Farzaneh	2023	United States	Discriminative AI	Predicting complications	Detecting acute respiratory distress syndrome in intensive care unit patients	5	None	Internal dataset	Internal dataset	1.000-10.000	100-1.000	N/A	Low	Low	High	Unclear	High
**267**	Fathi	2022	Iran	Discriminative AI	Predicting mortality	Predicting 1-month mortality of postsurgical intensive care patients	3&4	None	Internal dataset	N/A	100-1000	N/A	N/A	Low	Unclear	Low	High	High
**268**	Fathi	2022	Iran	Discriminative AI	Predicting clinical score	Predicting Glasgow Coma Scale score in patients admitted to the intensive care unit	3&4	None	Internal dataset	N/A	1.000-10.000	N/A	N/A	Low	Unclear	Low	High	High
**269**	Faucoz	2023	France	Discriminative AI	Assessing videos and images	Classifying lung ultrasonography images frames for COVID-19 patients with acute respiratory failure at the intensive care unit	3&4	None	Internal dataset	N/A	1.000-10.000	N/A	N/A	Low	Low	High	Unclear	High
**270**	Feng	2021	China	Discriminative AI	Improving mechanical ventilation	Predicting late noninvasive ventilation failure in intensive care unit patients	3&4	None	MIMIC III	N/A	1.000-10.000	N/A	N/A	Low	Low	Low	Low	Low
**271**	Feng	2023	China	Discriminative AI (Reinforcement learning)	Treatment recommendation	Providing optimal noninvasive ventilation switching regime for patients admitted to the intensive care unit	3&4	None	MIMIC III	N/A	10.000-100.000	N/A	N/A	N/A	N/A	N/A	N/A	N/A
**272**	Feng	2023	United States	Discriminative AI	Treatment recommendation	Suggesting treatment of vasopressors and mechanical ventilation for sepsis patients at the intensive care unit	3&4	None	MIMIC III	N/A	10.000-100.000	N/A	N/A	Unclear	Low	Low	High	High
**273**	Feng	2024	China	Discriminative AI	Predicting mortality	Predicting mortality in sepsis patients at the intensive care unit	3&4	None	MIMIC III	N/A	10.000-100.000	N/A	N/A	N/A	N/A	N/A	N/A	N/A
**274**	Feretzakis	2021	Greece	Discriminative AI	Predicting complications	Predicting antimicrobial resistance in intensive care unit patients	3&4	None	Internal dataset	N/A	1.000-10.000	N/A	N/A	Unclear	Low	Low	Unclear	Unclear
**275**	Festor	2022	United Kingdom	Discriminative AI (Reinforcement learning)	Provide dosage recommendations	Suggesting a dosing strategy for intravenous fluids and/or vasopressors in sepsis pattients	5	None	N/A	MIMIC III	N/A	No information	N/A	N/A	N/A	N/A	N/A	N/A

© 2025 Berkhout WEM et al. *JAMA Network Open.*

**276**	Fitzgerald	2021	Australia	Discriminative AI	Determining physiological values	Predicting blood glucose in intensive care unit patients	3&4	None	MIMIC III	N/A	10.000-100.000	N/A	N/A	Unclear	Low	High	Unclear	Unclear
**277**	Fitzgerald	2023	Australia	Discriminative AI	Determining physiological values	Forecasting blood glucose in the intensive care unit	3&4	None	MIMIC IV	N/A	100.000+	N/A	N/A	Unclear	Low	Low	Unclear	Unclear
**278**	Fleuren	2021	Netherlands	Discriminative AI	Improving mechanical ventilation	Predicting extubation failure in COVID-19 patients	3&4	TRIPOD/ TRIPOD+AI	Other type of dataset	N/A	1.000-10.000	N/A	N/A	Low	Low	Low	High	High
**279**	Fleuren	2021	Netherlands	Discriminative AI	Predicting mortality; Predicting prognosis	Predicting intensive care unit (ICU) mortality, ICU-free days in 30 days and ventilator-free days in 30 days	3&4	TRIPOD/ TRIPOD+AI	Other type of dataset	N/A	100-1000	N/A	N/A	Low	Low	Low	High	High
**280**	Fong	2024	United States	Discriminative AI	Predicting complications	Predicting intracranial pressure in intensive care unit patients	5	TRIPOD/ TRIPOD+AI	eICU-CRD	MIMIC III	10.000-100.000	1.000 - 10.000	N/A	Low	Low	High	Unclear	Unclear
**281**	Fonseca	2023	Portugal	Discriminative AI	Predicting mortality	Predicting mortality for traumatic brain injury patients at the intensive care unit	3&4	None	MIMIC III	N/A	1.000-10.000	N/A	N/A	Low	Low	Low	Unclear	Unclear
**282**	Forte	2021	Netherlands	Discriminative AI	Classifying sub-populations	Improve the characterizing of risk groups in critical care	3&4	None	Other type of dataset	N/A	100-1000	N/A	N/A	N/A	N/A	N/A	N/A	N/A
**283**	Franca	2024	Brazil	Discriminative AI	Predicting need for resource	Predicting the use of renal replacement therapy in critically ill patients	5	TRIPOD/ TRIPOD+AI	Internal dataset	Internal dataset	10.000-100.000	100-1.000	N/A	Low	Low	Low	Low	Low
**284**	Franca	2024	Brazil	Discriminative AI	Predicting complications	Predicting the use of renal replacement therapy in critically ill patients	5	None	Internal dataset	Internal dataset	10.000-100.000	1.000 - 10.000	N/A	Unclear	Unclear	Low	Unclear	Unclear
**285**	Fransén	2022	Sweden	Discriminative AI	Predicting complications	Predicting mortality in burn patients at the intensive care unit	3&4	None	Internal dataset	N/A	0-100	N/A	N/A	Unclear	Low	Low	High	High
**286**	Fritsch	2024	Germany	Discriminative AI	Improving mechanical ventilation	Predicting the duration of unassisted spontaneous breathing in patients during prolonged weaning for intensive care unit patients	3&4	None	Internal dataset	N/A	10.000-100.000	N/A	N/A	Unclear	Low	Low	Unclear	Unclear
**287**	Fu	2021	United States	Discriminative AI	Predicting deterioration	Predicting the composite outcome death, cardiac arrest and renal replacement therapy for intensive care unit patients	3&4	None	Internal dataset	N/A	1.000-10.000	N/A	N/A	Low	Unclear	Low	High	High
**288**	Fu	2022	China	Discriminative AI	Predicting complications	Predicting intracranial infection in patients under external ventricular drainage and neurological intensive care	3&4	None	Internal dataset	N/A	100-1000	N/A	N/A	Unclear	Unclear	Low	Unclear	Unclear
**289**	Fu	2024	China	Discriminative AI	Predicting need for resource	Predicting 48-h intubation risk in intensive care unit patients	5	None	MIMIC IV	MIMIC III	100-1000	100-1.000	N/A	Low	Unclear	Low	Unclear	Unclear
**290**	Fuadah	2022	Korea	Discriminative AI	Classifying sub-populations; Determining physiological values	Classification of blood pressure on photoplethysmogram	3&4	None	MIMIC III	N/A	10.000-100.000	N/A	N/A	Unclear	Low	Unclear	Low	Unclear
**291**	Fuest	2023	Germany	Discriminative AI	Classifying sub-populations	Clustering of intensive care unit patients for recommendations for mobilization	3&4	None	Internal dataset	N/A	100-1000	N/A	N/A	N/A	N/A	N/A	N/A	N/A
**292**	Fujarski	2022	Germany	Discriminative AI	Predicting complications	Predicting acute kidney injury in the intensive care unit	3&4	None	AmsterdamUMCdb	N/A	No information	N/A	N/A	Unclear	Unclear	Unclear	Unclear	Unclear
**293**	Futoma	2020	United States	Discriminative AI (Reinforcement Learning)	Treatment recommendation	Treatment recommendation for patients with acute hypotension	3&4	None	MIMIC III	N/A	10.000-100.000	N/A	N/A	N/A	N/A	N/A	N/A	N/A
**294**	Gakuba	2024	France	Generative AI (Large language model)	Predicting prognosis	Predicting the 6-month outcome prognosis of traumatic brain injury patients admitted to the intensive care unit	5	TRIPOD/ TRIPOD+AI	N/A	Internal dataset	N/A	0-100	N/A	N/A	N/A	N/A	N/A	N/A
**295**	Gandin	2021	Italy	Discriminative AI	Predicting mortality	Predicting mortality in patients with cardiovascular	3&4	None	MIMIC III	N/A	10.000-100.000	N/A	N/A	Unclear	Low	Low	High	High

© 2025 Berkhout WEM et al. *JAMA Network Open.*

						diseases admitted to the intensive care unit												
**296**	Ganglberger	2023	United States	Discriminative AI	Predicting clinical score	Measuring sleep state in the intensive care unit	5	STROBE	Other type of dataset	Other type of dataset	100.000+	No information	N/A	Low	Low	High	High	High
**297**	Ganzert	2002	Germany	Discriminative AI	Classification of signals, Determining physiological values	Classifying methods for pressure-volume curves and predicting volume given the pressure in ventilated intensive care unit patients	3&4	None	Internal dataset	N/A	No information	N/A	N/A	Unclear	Unclear	Low	High	High
**298**	Gao	2021	United States	Discriminative AI	Predicting complications	Predicting sepsis in the intensive care unit	3&4	None	eICU-CRD	N/A	1.000-10.000	N/A	N/A	Low	Unclear	High	Unclear	High
**299**	Gao	2021	China	Discriminative AI	Predicting complications	Predicting acute kidney injury in the intensive care unit	3&4	None	MIMIC III	N/A	10.000-100.000	N/A	N/A	Low	Low	Low	Unclear	Unclear
**300**	Gao	2021	China	Discriminative AI	Assessing clinical notes	Extracting sentiment information in nursing notes	5	None	N/A	MIMIC III	N/A	1.000 - 10.000	N/A	N/A	N/A	N/A	N/A	N/A
**301**	Gao	2022	United States	Discriminative AI	Classifying sub-populations	Predicting clinical state of patient-day	3&4	None	Other type of dataset	N/A	100-1000	N/A	N/A	Low	Unclear	Low	Unclear	Unclear
**302**	Gao	2022	United States	Generative AI (Large language model)	Assessing clinical notes	Summarizing patients' main problems from daily progress notes.	3&4	None	MIMIC III	N/A	No information	N/A	N/A	Unclear	Unclear	Low	Unclear	Unclear
**303**	Gao	2023	China	Discriminative AI	Predicting complications	Predicting acute kidney injury after cardiac surgery	3&4	TRIPOD/ TRIPOD+AI	Internal dataset	N/A	10.000-100.000	N/A	N/A	Low	Unclear	Low	Low	Unclear
**304**	Gao	2024	China	Discriminative AI	Predicting mortality; Predicting length of stay	Predicting length of stay and mortality for COVID-19 intensive care unit patients	3&4	None	Other type of dataset	N/A	100-1000	N/A	N/A	N/A	N/A	N/A	N/A	N/A
**305**	Gao	2024	China	Generative AI (Large language model)	Predicting mortality	Predicting in-hospital mortality in heart failure patients admitted to the intensive care unit	5	TRIPOD/ TRIPOD+AI	MIMIC III, MIMIC IV	MIMIC IV, eICU-CRD	10.000-100.000	1.000 - 10.000	N/A	Low	Low	Low	Unclear	Unclear
**306**	Gao	2024	China	Discriminative AI	Predicting mortality	Predicting in-hospital mortality for critically ill patients with sepsis-associated acute kidney injury	3&4	None	MIMIC IV	N/A	10.000-100.000	N/A	N/A	Low	Unclear	Unclear	Unclear	Unclear
**307**	Gao	2024	United States	Discriminative AI	Predicting mortality	Predicting sepsis mortality in the intensive care unit	3&4	None	MIMIC IV	N/A	1.000-10.000	N/A	N/A	N/A	N/A	N/A	N/A	N/A
**308**	Ge	2022	China	Discriminative AI	Predicting complications	Predicting sepsis-associated acute brain injury in intensive care unit patients	3&4	None	MIMIC III	N/A	10.000-100.000	N/A	N/A	Low	High	High	Low	High
**309**	Ge	2022	United States	Discriminative AI	Predicting mortality	Predicting mortality of patients admitted to the intensive care unit	3&4	None	Internal dataset	N/A	1.000-10.000	N/A	N/A	Low	Low	Low	Unclear	Unclear
**310**	George	2021	United States	Discriminative AI	Predicting mortality	Predicting mortality in patients requiring more than 7 days of mechanical ventilation	3&4	JMIR Guidelines for Developing and Reporting Machine Learning Predictive Models in Biomedical Research	MIMIC III	N/A	1.000-10.000	N/A	N/A	Low	Unclear	Low	Unclear	Unclear
**311**	Getzen	2022	United States	Generative AI (Large language model)	Diagnosing	Predicting diabetes, chronic kidney disease, heart failure, lipid metabolism disorder, fluid / electrolyte disorder and cardiac dysrhythmia in patients admitted to the intensive care unit	3&4	None	MIMIC III	N/A	1.000-10.000	N/A	N/A	Unclear	Low	Low	Unclear	Unclear
**312**	Ghanvatkar	2024	Singapore	Discriminative AI	Predicting complications	Predicting sepsis in the intensive care unit	3&4	None	Other type of dataset	N/A	10.000-100.000	N/A	N/A	Unclear	Unclear	Unclear	Unclear	Unclear
**313**	Ghassemi	2012	United States	Discriminative AI	Predicting mortality	Predicting mortality in the intensive care unit	3&4	None	MIMIC II	N/A	10.000-100.000	N/A	N/A	Low	Low	Low	Unclear	Unclear

© 2025 Berkhout WEM et al. *JAMA Network Open.*

**314**	Ghassemi	2014	United States	Discriminative AI	Predicting mortality	Predicting mortality for intensive care unit patients	3&4	None	MIMIC II	N/A	10.000-100.000	N/A	N/A	Low	Unclear	Low	Unclear	Unclear
**315**	Ghassemi	2015	United States	Discriminative AI	Predicting mortality	Predicting mortality	3&4	None	MIMIC II	N/A	10.000-100.000	N/A	N/A	Unclear	Unclear	Low	Unclear	Unclear
**316**	Ghassemi	2017	United States	Discriminative AI	Predicting need for resource	Predicting need for intensive care unit interventions	3&4	None	MIMIC III	N/A	10.000-100.000	N/A	N/A	Low	Low	Low	Unclear	Unclear
**317**	Ghavidel	2023	Iran	Discriminative AI	Predicting mortality	Predicting mortality for patients after cardiac surgery	3&4	None	Internal dataset	N/A	1.000-10.000	N/A	N/A	Unclear	Low	Low	High	High
**318**	Ghazi	2022	United Kingdom	Discriminative AI	Predicting mortality	ICU mortality	3&4	None	Other type of dataset	N/A	10.000-100.000	N/A	N/A	Unclear	Low	Low	Low	Unclear
**319**	Ghias	2022	Pakistan	Discriminative AI	Predicting complications	Predicting sepsis in intensive care unit patients	3&4	None	Other type of dataset	N/A	1.000-10.000	N/A	N/A	Low	Unclear	Low	Low	Unclear
**320**	Gholamzadeh	2023	Iran	Discriminative AI	Predicting complications	Predicting risk of sepsis in intensive care unit patients	3&4	None	MIMIC III	N/A	1.000-10.000	N/A	N/A	Low	Unclear	Unclear	Unclear	Unclear
**321**	Ghorbani	2020	Netherlands	Discriminative AI	Predicting mortality	Predicting mortality	3&4	None	Internal dataset	N/A	1.000-10.000	N/A	N/A	Unclear	Low	Low	Low	Unclear
**322**	Ghosh	2021	United States	Discriminative AI	Determining physiological thresholds	Predicting baseline serum creatinine in intensive care unit patients	5	None	Internal dataset	MIMIC III	10.000-100.000	1.000 - 10.000	N/A	Low	Low	Low	Unclear	Unclear
**323**	Ghosheh	2023	United Kingdom	Discriminative AI	Predicting complications	Predicting the onset of hospital-acquired infections for patients admitted to the intensive care unit	3&4	None	Internal dataset	N/A	100-1000	N/A	N/A	Unclear	Low	Low	Unclear	Unclear
**324**	Giampanis	2021	United States	Discriminative AI	Predicting the need for resource	Predicting unexpected mechanical ventilation	3&4	None	MIMIC III	N/A	10.000-100.000	N/A	N/A	Unclear	Low	Low	High	High
**325**	Giang	2021	United States	Discriminative AI	Predicting complications	Predicting ventilator-associated pneumonia in mechanically ventilated patients admitted to the intensive care unit	3&4	None	MIMIC III	N/A	1.000-10.000	N/A	N/A	Low	Low	Low	Low	Low
**326**	Giri	2023	India	Discriminative AI	Improving mechanical ventilation	Predicting the ventilation mode	3&4	None	Internal dataset	N/A	100-1000	N/A	N/A	Low	Low	High	Unclear	High
**327**	Goh	2021	Singapore	Discriminative AI	Predicting complications	Diagnosis of sepsis in intensive care unit patients	3&4	None	Internal dataset	N/A	100-1000	N/A	N/A	Unclear	Unclear	Low	High	High
**328**	Gomes	2021	Germany	Discriminative AI	Predicting mortality; Predicting complications	Predicting in-hospital mortality, stroke/TIA, major vascular complications, paravalvular leakage and new pacemaker implantations in patients undergoing TAVI	3&4	None	Internal dataset	N/A	100-1000	N/A	N/A	Unclear	Unclear	Low	High	High
**329**	Gonçalves	2014	Portugal	Discriminative AI	Predicting complications	Predicting sepsis level and treatment plan in intensive care unit patients with sepsis	3&4	None	Internal dataset	N/A	100-1000	N/A	N/A	Unclear	Unclear	Unclear	Unclear	Unclear
**330**	Gong	2021	China	Discriminative AI	Predicting complications	Predicting acute kidney injury in critical care	3&4	None	MIMIC III	N/A	10.000-100.000	N/A	N/A	Low	Low	High	Low	High
**331**	Gong	2023	China	Discriminative AI (Reinforcement Learning)	Treatment recommendation	Developing personalized sedation and ventilation protocols	3&4	None	MIMIC III	N/A	No information	N/A	N/A	N/A	N/A	N/A	N/A	N/A
**332**	Gong	2023	United States	Discriminative AI	Predicting complications	Predicting intensive care delirium	5	TRIPOD/ TRIPOD+AI	eICU-CRD	MIMIC III, MIMIC IV	10.000-100.000	10.000 - 100.000	N/A	Unclear	Low	Low	Low	Unclear
**333**	Gong	2023	China	Discriminative AI	Predicting readmissions	Predicting intensive care unit readmission after liver transplantation	3&4	None	Internal dataset	N/A	100-1000	N/A	N/A	Low	Unclear	Low	Unclear	Unclear
**334**	Gonzalez-Novoa	2021	Spain	Discriminative AI	Predicting mortality	Predicting mortality in intensive care unit patients	3&4	None	MIMIC III	N/A	1.000-10.000	N/A	N/A	Unclear	Low	Low	Unclear	Unclear
**335**	González-Nóvoa	2023	Spain	Discriminative AI	Predicting length of stay	Predicting length of stay in the intensive care unit	3&4	None	MIMIC III	N/A	10.000-100.000	N/A	N/A	Low	Low	Low	High	High
**336**	González-Nóvoa	2023	Spain	Discriminative AI	Predicting readmissions	Predicting intensive care unit early readmission	3&4	None	MIMIC III	N/A	10.000-100.000	N/A	N/A	Low	Low	Low	High	High

© 2025 Berkhout WEM et al. *JAMA Network Open.*

**337**	Gordon	2021	United States	Discriminative AI	Classifying sub-populations	Identify intracranial mass effect	3&4	None	Internal dataset	N/A	1.000-10.000	N/A	N/A	N/A	N/A	N/A	N/A	N/A
**338**	Gordon	2022	United States	Discriminative AI	Predicting complications	Predicting intracranial mass effect in intensive care unit patients	3&4	None	Internal dataset	N/A	1.000-10.000	N/A	N/A	Low	Low	Low	Unclear	Unclear
**339**	Gortzis	2008	Greece	Discriminative AI	Predicting mortality	Predicting survival at the intensive care unit	3&4	None	Internal dataset	N/A	100-1000	N/A	N/A	Unclear	Unclear	Low	High	High
**340**	Gosset	2021	Belgium	Discriminative AI	Predicting complications	Identifying bacteremia in intensive care unit patients	5	None	Internal dataset	Internal dataset	100-1000	0-100	N/A	Low	Low	Low	High	High
**341**	Gourdeau	2022	Canada	Discriminative AI	Assessing videos and images	Predicting outcome (remission or mortality) of mechanical ventilation in intensive care unit patients	3&4	STARD/ STARD-AI	Internal dataset	N/A	100-1000	N/A	N/A	Low	Low	Low	High	High
**342**	Gourdeau	2022	Canada	Discriminative AI	Assessing videos and images	Predicting COVID-19 radiological trajectory in COVID-19 patients admitted to the intensive care unit	3&4	STARD/ STARD-AI	Internal dataset	N/A	100-1000	N/A	N/A	Low	Low	High	High	High
**343**	Grapin	2024	France	Discriminative AI	Classifying sub-populations	Identifying subgroups of patients admitted at the intensive care unit with COVID-19 pneumonia	3&4	None	Internal dataset	N/A	100-1000	N/A	N/A	N/A	N/A	N/A	N/A	N/A
**344**	Greco	2022	Italy	Discriminative AI	Predicting mortality	Predicting mortality in COVID-19 patients admitted to the intensive care unit	3&4	STROBE	Internal dataset	N/A	1.000-10.000	N/A	N/A	Unclear	Low	Low	Low	Unclear
**345**	Grolleau	2024	France	Discriminative AI (Reinforcement learning)	Treatment recommendation	Estimating strategies for renal replacement therapy initiation in intensive care unit patients with acute kidney injury	5	None	MIMIC III	Other type of dataset	1.000-10.000	1.000 - 10.000	N/A	N/A	N/A	N/A	N/A	N/A
**346**	Gu	2023	United States	Discriminative AI	Predicting complications	Predicting hospital-acquired pressure injuries at the intensive care unit	3&4	None	MIMIC III	N/A	1.000-10.000	N/A	N/A	Unclear	Low	Low	High	High
**347**	Gu	2024	China	Discriminative AI	Predicting mortality	Predicting mortality within 7 days in subarachonoid hemorrhage in intensive care unit patients	5	None	MIMIC III	Internal dataset	100-1000	100-1.000	N/A	Low	Low	Low	Unclear	Unclear
**348**	Guan	2023	China	Discriminative AI	Predicting complications	Predicting venous thromboembolism in critically ill patients	5	None	eICU-CRD	eICU-CRD	100.000+	10.000 - 100.000	N/A	Low	Low	Low	Low	Low
**349**	Gulamali	2024	United States	Discriminative AI	Determining physiological values	Predicting increased intracranial pressure in intensive care unit patients	5	TRIPOD/ TRIPOD+AI	MIMIC III	Internal dataset	100-1000	0-100	N/A	Low	Unclear	Low	High	High
**350**	Guo	2021	China	Discriminative AI	Predicting mortality	Predicting mortality	3&4	None	MIMIC III	N/A	1.000-10.000	N/A	N/A	Unclear	Unclear	Low	Unclear	Unclear
**351**	Guo	2022	China	Discriminative AI	Predicting mortality	Predicting survival in sepsis patients in the intensive care unit	5	RECORD	MIMIC III	MIMIC IV	1.000-10.000	No information	N/A	N/A	N/A	N/A	N/A	N/A
**352**	Guo	2022	Canada	Discriminative AI	Predicting mortality; Predicting complications; Predicting need for resource	Predicting mortality, long length of stay, sepsis and invasive ventilation at the intensive care unit	3&4	None	MIMIC IV	N/A	10.000-100.000	N/A	N/A	Low	Low	Low	High	High
**353**	Guo	2022	China	Discriminative AI (Reinforcement Learning)	Treatment recommendation	Treatment strategies for coronary heart diseases	3&4	None	MIMIC III	N/A	10.000-100.000	N/A	N/A	Low	Low	Unclear	Unclear	Unclear
**354**	Guo	2023	China	Discriminative AI	Predicting mortality	Predicting intensive care unit mortality for sepsis-associated encephalopathy patients	5	TRIPOD/ TRIPOD+AI	MIMIC IV	eICU-CRD	1.000-10.000	1.000 - 10.000	N/A	Low	Unclear	Low	High	High
**355**	Guo	2023	China	Discriminative AI	Predicting mortality	Predicting 28-day mortality in critically ill elderly patients with colorectal cancer	5	None	eICU-CRD	Internal dataset, MIMIC IV	100-1000	100-1.000	N/A	Low	Low	Low	Low	Low
**356**	Guo	2024	China	Discriminative AI	Predicting mortality	Predicting mortality in patients with traumatic brain injury	3&4	None	MIMIC III	N/A	1.000-10.000	N/A	N/A	Low	Low	Low	High	High

© 2025 Berkhout WEM et al. *JAMA Network Open.*

**357**	Gupta	2021	United States	Discriminative AI	Predicting mortality	Predicting mortality in sepsis patients admitted to the intensive care unit	3&4	None	MIMIC III	N/A	1.000-10.000	N/A	N/A	Unclear	Low	Low	High	High
**358**	Gupta	2022	India	Discriminative AI	Determining physiological values	Estimating blood pressure for intensive care unit patients	3&4	None	MIMIC I	N/A	100.000+	N/A	N/A	Unclear	Low	Low	High	High
**359**	Habibzadeh	2023	Iran	Discriminative AI	Predicting need for resource	Predicting the need for neurosurgical intervention after moderate traumatic brain injury in the intensive care unit	3&4	None	Internal dataset	N/A	100-1000	N/A	N/A	Low	Low	Unclear	High	High
**360**	Hadler	2023	United States	Discriminative AI	Predicting deterioration	Predicting patients in the intensive care unit remaining alert and without delirium for at least two more days	3&4	None	Internal dataset	N/A	1.000-10.000	N/A	N/A	Low	Unclear	Unclear	Unclear	Unclear
**361**	Hagan	2020	United Kingdom	Discriminative AI	Determining physiological values	Predicting the tidal volume per ideal body weight in intensive care unit patients	3&4	None	Internal dataset	N/A	No information	N/A	N/A	Unclear	Low	Unclear	Unclear	Unclear
**362**	Hamar	2024	Hungary	Discriminative AI	Predicting mortality	Predicting mortality in intensive care unit patients with COVID-19	3&4	None	Internal dataset	N/A	100-1000	N/A	N/A	Low	Low	Low	Unclear	Unclear
**363**	Hameed	2022	Pakistan	Discriminative AI	Predicting mortality	Predicting mortality in acute pancreatitis patients	3&4	None	eICU-CRD, MIMIC III, MIMIC IV	N/A	1.000-10.000	N/A	N/A	Unclear	Unclear	Low	High	High
**364**	Hamzi	2024	France	Discriminative AI	Classifying sub-populations	Identifying phenotypes in intensive cardiac care unit patients	3&4	STROBE	Other type of dataset	N/A	1.000-10.000	N/A	N/A	N/A	N/A	N/A	N/A	N/A
**365**	Han	2022	United States	Discriminative AI	Predicting health improvement	Identification of patient social determinants of health	3&4	TRIPOD/ TRIPOD+AI	MIMIC III	N/A	1.000-10.000	N/A	N/A	N/A	N/A	N/A	N/A	N/A
**366**	Han	2022	Japan	Discriminative AI	Assessing clinical notes	Extracting subjective words from reports of intensive care unit patients	3&4	None	MIMIC III	N/A	10.000-100.000	N/A	N/A	N/A	N/A	N/A	N/A	N/A
**367**	Han	2024	China	Discriminative AI	Predicting mortality	Predicting mortality in intensive care unit patients with orthopaedic trauma	5	TRIPOD/ TRIPOD+AI	MIMIC III	Internal dataset	1.000-10.000	100-1.000	N/A	Unclear	Unclear	Low	High	High
**368**	Händel	2023	Germany	Discriminative AI	Determining physiological values	Predicting arterial partial pressure or oxygen, arterial partial pressure of carbon dioxide and respiratory system compliance in intensive care unit patients	5	None	MIMIC III	eICU-CRD	100.000+	100.000+	N/A	Unclear	Unclear	Unclear	Unclear	Unclear
**369**	Hanin	2022	Germany	Discriminative AI	Predicting prognosis	Predicting the outcome of status epilepticus patients in intensive care unit using the Rankin Scale	3&4	STROBE	Internal dataset	N/A	0-100	N/A	N/A	Low	Unclear	Low	High	High
**370**	Haredasht	2023	Belgium	Discriminative AI	Predicting mortality	Predicting mortality for patients with acute kidney injury in the intensive care unit	5	None	Internal dataset	Internal dataset	100-1000	0-100	N/A	Low	Low	Low	Unclear	Unclear
**371**	Harerimana	2021	Korea	Discriminative AI	Predicting mortality; Predicting length of stay	Predicting in-hospital mortality and length of stay	3&4	None	MIMIC III	N/A	10.000-100.000	N/A	N/A	Low	Low	Low	Unclear	Unclear
**372**	Harerinmana	2022	Rwanda	Discriminative AI	Predicting mortality; Predicting length of stay	Predicting length of stay, in-hospital mortality and 24 hours mortality	3&4	None	MIMIC III	N/A	10.000-100.000	N/A	N/A	Unclear	Unclear	Low	Unclear	Unclear
**373**	Hasegawa	2020	Japan	Discriminative AI	Predicting complications	Predicting sepsis-induced coagulopathy progression in intensive care unit patients	3&4	TRIPOD/ TRIPOD+AI	Other type of dataset	N/A	1.000-10.000	N/A	N/A	Low	Low	High	Unclear	High
**374**	Hashir	2020	Canada	Discriminative AI	Predicting mortality	Predicting in-hospital mortality for patients admitted to the intensive care unit	3&4	None	MIMIC III	N/A	10.000-100.000	N/A	N/A	Low	Low	Low	Unclear	Unclear
**375**	Hassanzadeh	2023	Iran	Discriminative AI	Predicting mortality	Predicting hospital mortality in trauma patients admitted to the intensive care unit	3&4	None	Internal dataset	N/A	100-1000	N/A	N/A	Low	Unclear	Low	Unclear	Unclear

© 2025 Berkhout WEM et al. *JAMA Network Open.*

**376**	He	2020	China	Discriminative AI	Predicting complications	Predicting sepsis in intensive care unit patients	3&4	None	Other type of dataset, No information	N/A	10.000-100.000	N/A	N/A	Low	Unclear	Unclear	Unclear	Unclear
**377**	He	2021	China	Discriminative AI	Predicting complications	Predicting the occurrence of acute kidney disease	5	TRIPOD/ TRIPOD+AI	Internal dataset	MIMIC III	100-1000	100-1.000	N/A	Low	Low	Unclear	Unclear	Unclear
**378**	Heffernan	2022	Australia	Discriminative AI	Predicting mortality	Predicting in-hospital mortality in intensive care unit patients	3&4	None	Other type of dataset	N/A	100.000+	N/A	N/A	Low	Unclear	Low	Unclear	Unclear
**379**	Hegselmann	2022	Germany	Discriminative AI	Predicting readmissions	Predicting 3 day intensive care unit readmission	5	TRIPOD/ TRIPOD+AI	Internal dataset	MIMIC IV	10.000-100.000	10.000 - 100.000	N/A	Low	Low	Low	High	High
**380**	Heimark	2023	Norway	Discriminative AI	Determining physiological values	Predicting blood pressure in critically ill intensive care unit patients	3&4	None	Internal dataset	N/A	0-100	N/A	N/A	Low	Low	Low	Unclear	Unclear
**381**	Hempel	2023	Germany	Discriminative AI	Predicting length of stay	Predicting length of stay in the intensive care unit	3&4	None	MIMIC IV	N/A	10.000-100.000	N/A	N/A	Low	Low	Low	High	High
**382**	Hern	2023	Spain	Discriminative AI	Predicting complications	Predicting antibiotic resistance in pseudomonas aeruginosa nosocomial infections at the intensive care unit	3&4	None	Internal dataset	N/A	100-1000	N/A	N/A	Unclear	Low	Low	High	High
**383**	Herzog	2021	Germany	Discriminative AI	Predicting complications	Predicting kidney failure, mortality and the development of chronic kidney disease in intensive care unit patients with COVID-19	3&4	None	Internal dataset	N/A	0-100	N/A	N/A	Low	Unclear	Unclear	High	High
**384**	Hill	2021	United States	Discriminative AI	Determining physiological values	Imputing continuous arterial blood pressure non-invasively in intensive care unit patients	5	JMIR Guidelines for Developing and Reporting Machine Learning Predictive Models in Biomedical Research	MIMIC III	Internal dataset	No information	No information	N/A	Unclear	Low	Low	Unclear	Unclear
**385**	Hirano	2021	Japan	Discriminative AI	Predicting complications	Predicting positive methicilin-resistant Staphlococcus aureus screening during mechanical ventilation	3&4	None	MIMIC IV	N/A	100-1000	N/A	N/A	Low	Low	Low	High	High
**386**	Hirzallah	2023	United States	Discriminative AI	Assessing videos and images	Automatic optic nerve sheath diameter measurement	3&4	None	Internal dataset	N/A	100-1000	N/A	N/A	Unclear	Low	Low	Unclear	Unclear
**387**	Ho	2023	United States	Discriminative AI	Predicting complications	Detect hospital-acquired pressure injury	3&4	None	MIMIC III	N/A	No information	N/A	N/A	Unclear	Low	Unclear	Unclear	Unclear
**388**	Holder	2021	United States	Discriminative AI	Predicting need for resource	Predicting future vasopressor use among sepsis patients	5	TRIPOD/ TRIPOD+AI	Internal dataset	Internal dataset	1.000-10.000	1.000 - 10.000	N/A	Low	Low	Low	Unclear	Unclear
**389**	Holmgren	2019	Sweden	Discriminative AI	Predicting mortality	Predicting 30-day mortality for ICU patients	3&4	None	Internal dataset	N/A	100.000+	N/A	N/A	Low	Low	Low	Unclear	Unclear
**390**	Hong	2022	China	Discriminative AI	Classifying sub-populations	Identifying phenogroups in patients admitted for heart failure with normal renal function	5	None	Internal dataset	MIMIC III	1.000-10.000	1.000 - 10.000	N/A	N/A	N/A	N/A	N/A	N/A
**391**	Hong	2023	China	Discriminative AI	Predicting complications	Predicting low cardiac output syndrome	3&4	None	Internal dataset	N/A	1.000-10.000	N/A	N/A	Low	Low	High	High	High
**392**	Horton	2022	United States	Discriminative AI	Predicting complications	Predicting hypoglycemia in the intensive care unit	5	TRIPOD/ TRIPOD+AI	Internal dataset	MIMIC III	10.000-100.000	1.000 - 10.000	N/A	Low	Unclear	Low	High	High
**393**	Hossein Abad	2020	Canada	Discriminative AI	Predicting mortality	Predicting mortality for critically ill patients	3&4	None	eICU-CRD	N/A	100.000+	N/A	N/A	Unclear	Low	Low	Low	Unclear
**394**	Hou	2020	China	Discriminative AI	Predicting mortality	Predicting 30-day mortality for intensive care unit patients with sepsis	3&4	None	MIMIC III	N/A	1.000-10.000	N/A	N/A	Low	Low	Low	High	High
**395**	Hripcsak	2022	United States	Discriminative AI	Determining physiological values	Predicting glucose in neurological intensive care unit patients	3&4	None	Internal dataset	N/A	No information	N/A	N/A	Low	Unclear	Unclear	Unclear	Unclear

© 2025 Berkhout WEM et al. *JAMA Network Open.*

**396**	Hsu	2023	United States	Discriminative AI	Predicting mortality; Predicting health improvement	Predicting survival and favorable neurological outcome in the hyperacute phase of adult patients in the intensive care unit receiving targeted temperature management	3&4	None	eICU-CRD	N/A	100-1000	N/A	N/A	Low	Low	Low	High	High
**397**	Hu	2022	China	Discriminative AI	Predicting mortality	Predicting in-hospital mortality in acute kidney injury	3&4	TRIPOD/ TRIPOD+AI	MIMIC IV	N/A	10.000-100.000	N/A	N/A	Unclear	Low	Low	High	High
**398**	Hu	2022	China	Discriminative AI	Classifying sub-populations	Identifying sepsis subphenotypes	3&4	STROBE	MIMIC IV	N/A	1.000-10.000	N/A	N/A	Low	Low	Low	Low	Low
**399**	Hu	2022	China	Discriminative AI	Predicting complications	Predicting feeding intolerance in intensive care unit patients with sepsis	5	None	Internal dataset	Internal dataset	100-1000	0-100	N/A	N/A	N/A	N/A	N/A	N/A
**400**	Hu	2022	China	Discriminative AI	Predicting deterioration	Predicting life-threatening events in intensive care unit patients	3&4	None	Internal dataset	N/A	1.000-10.000	N/A	N/A	Low	Unclear	Low	High	High
**401**	Hu	2022	China	Discriminative AI	Predicting mortality	Predicting mortality in septic patients readmitted to the intensive care unit	3&4	TRIPOD/ TRIPOD+AI	MIMIC IV	N/A	1.000-10.000	N/A	N/A	N/A	N/A	N/A	N/A	N/A
**402**	Hu	2022	China	Discriminative AI	Predicting mortality	Predicting in-hospital mortality in sepsis patients at the intensive care unit	3&4	TRIPOD/ TRIPOD+AI	MIMIC IV	N/A	1.000-10.000	N/A	N/A	Low	Low	Low	High	High
**403**	Hu	2022	China	Discriminative AI	Predicting mortality	Predicting mortality in intensive care unit patients with acute respiratory distress syndrome	3&4	None	Internal dataset	N/A	100-1000	N/A	N/A	Low	Low	Low	High	High
**404**	Hu	2023	China	Discriminative AI	Predicting mortality	Predicting in-hospital mortality from ischemic stroke in the intensive care unit	5	TRIPOD/ TRIPOD+AI	MIMIC IV	eICU-CRD	1.000-10.000	1.000 - 10.000	N/A	Unclear	Low	Low	High	High
**405**	Hu	2023	India	Discriminative AI	Predicting complications	Predicting hypertension	3&4	None	MIMIC III	N/A	100-1000	N/A	N/A	Low	Low	Low	High	High
**406**	Hu	2023	China	Discriminative AI	Classifying sub-populations	Identifying phenotypes of intensive care unit onset sepsis	3&4	RECORD	MIMIC IV	N/A	1.000-10.000	N/A	N/A	Low	Low	Low	Unclear	Unclear
**407**	Hu	2024	United States	Discriminative AI	Predicting complications	Predicting cardiogenic shock in cardiac intensive care unit patients	5	TRIPOD/ TRIPOD+AI	MIMIC III	Internal dataset	1.000-10.000	100-1.000	N/A	Low	Low	Low	Unclear	Unclear
**408**	Hu	2024	China	Discriminative AI	Predicting mortality	Predicting mortality in the intensive care unit	3&4	TRIPOD/ TRIPOD+AI	MIMIC IV	N/A	10.000-100.000	N/A	N/A	Low	Low	Low	High	High
**409**	Huang	2021	China	Discriminative AI	Predicting mortality	Predicting mortality of acute kidney injury in critical care patients	5	TRIPOD/ TRIPOD+AI	MIMIC III	Internal dataset, eICU-CRD	1.000-10.000	1.000 - 10.000	N/A	Low	Low	High	Unclear	High
**410**	Huang	2021	Taiwan	Discriminative AI	Predicting complications	Predicting acute kidney injury in intensive care unit patients	3&4	None	Internal dataset	N/A	1.000-10.000	N/A	N/A	Low	Unclear	Low	Unclear	Unclear
**411**	Huang	2021	China	Discriminative AI	Predicting mortality	Predicting in-hospital mortality for patients with acute respiratory distress syndrome at the intensive care unit	5	STROBE	MIMIC III	eICU-CRD	1.000-10.000	100-1.000	N/A	N/A	N/A	N/A	N/A	N/A
**412**	Huang	2021	United States	Discriminative AI	Predicting mortality; Predicting length of stay	Predicting length of stay and mortality for intensive care unit patients	3&4	None	MIMIC III	N/A	1.000-10.000	N/A	N/A	Unclear	Low	Low	High	High
**413**	Huang	2022	China	Discriminative AI	Assessing videos and images	Detecting atelectasis	5	None	Other type of dataset	Internal dataset	10.000-100.000	100-1.000	N/A	Low	Low	Low	Unclear	Unclear
**414**	Huang	2022	China	Discriminative AI	Predicting mortality	Predicting mortality in patients with acute myocardial infarction at the intensive care unit	3&4	None	MIMIC III, internal dataset	N/A	1.000-10.000	N/A	N/A	Low	Low	Low	High	High
**415**	Huang	2022	China	Discriminative AI	Predicting mortality	Predicting mortality in intensive care unit patients	3&4	None	MIMIC III	N/A	10.000-100.000	N/A	N/A	Low	Low	Low	High	High
**416**	Huang	2022	China	Discriminative AI	Predicting mortality	Predicting 28-day in-hospital mortality for elderly patients with	3&4	None	eICU-CRD	N/A	1.000-10.000	N/A	N/A	Low	Unclear	Low	Unclear	Unclear

© 2025 Berkhout WEM et al. *JAMA Network Open.*

						ischemic stroke in the intensive care unit												
**417**	Huang	2022	Taiwan	Discriminative AI	Assessing videos and images	Detecting endotracheal tube tip and carina and the distance between them in intubated intensive care unit patients	5	None	Internal dataset	Internal dataset	1.000-10.000	100-1.000	N/A	Low	Low	Low	High	High
**418**	Huang	2023	Taiwan	Discriminative AI	Predicting mortality	Predicting mortality of patients within six months and two years	3&4	None	MIMIC III	N/A	10.000-100.000	N/A	N/A	Low	Low	Low	Unclear	Unclear
**419**	Huang	2023	China	Discriminative AI	Determining physiological values	Continuous blood pressure monitoring	3&4	None	MIMIC III	N/A	100.000+	N/A	N/A	N/A	N/A	N/A	N/A	N/A
**420**	Huang	2023	Taiwan	Discriminative AI	Improving mechanical ventilation	Predicting successful extubation in intensive care unit patients	3&4	None	Internal dataset	N/A	100-1000	N/A	N/A	Low	Low	High	Unclear	High
**421**	Huang	2023	Belgium	Discriminative AI	Predicting health improvement	Predicting acute kidney injury recovery in critically ill patients	3&4	TRIPOD/ TRIPOD+AI	Other type of dataset	N/A	1.000-10.000	N/A	N/A	Low	Unclear	Low	Unclear	Unclear
**422**	Huang	2023	Belgium	Discriminative AI	Determining physiological values	Predicting creatinine clearance in critically ill adults	5	TRIPOD/ TRIPOD+AI	Other type of dataset	Internal dataset	10.000-100.000	1.000 - 10.000	N/A	Low	Low	Low	Low	Unclear
**423**	Huang	2023	Taiwan	Discriminative AI	Predicting complications	Predicting acute kidney injury in critically ill patients	5	TRIPOD/ TRIPOD+AI	Internal dataset	Internal dataset	No information	No information	N/A	Low	Low	Low	Low	Low
**424**	Huang	2023	China	Discriminative AI	Predicting complications	Predicting mechanical ventilation-associated severe acute kidney injury in intensive care unit patients	5	None	MIMIC IV	MIMIC IV, eICU-CRD	1.000-10.000	1.000 - 10.000	N/A	Low	Unclear	High	Unclear	High
**425**	Huang	2023	China	Discriminative AI	Predicting mortality	Predicting in-hospital mortality for hypertensive ischemic or hemorrhagic stroke patients in the intensive care unit	5	None	eICU-CRD	MIMIC IV	1.000-10.000	1.000 - 10.000	N/A	Unclear	Low	High	Unclear	Unclear
**426**	Huang	2023	China	Discriminative AI	Predicting mortality	Predicting in-hospital mortality in lung cancer patients admitted to the intensive care unit	5	STROBE	MIMIC IV	eICU-CRD	1.000-10.000	1.000 - 10.000	N/A	Low	Unclear	Low	Low	Unclear
**427**	Hui	2023	Australia	Discriminative AI	Predicting complications	Predicting bleeding after cardiac surgery	3&4	None	Internal dataset	N/A	1.000-10.000	N/A	N/A	Low	Low	Low	Unclear	Unclear
**428**	Hung	2022	Taiwan	Discriminative AI	Predicting mortality	Predicting in-hospital mortality for intensive care unit patients receiving continuous renal replacement therapy	3&4	None	Internal dataset	N/A	1.000-10.000	N/A	N/A	Low	Low	Low	Unclear	Unclear
**429**	Hunter	2020	United States	Discriminative AI	Predicting complications	Predicting flash and prolonged capillary refill in patients in the intensive care unit	5	None	Internal dataset	Internal dataset	100-1000	0-100	N/A	Low	Low	Low	Unclear	Unclear
**430**	Huo	2024	Germany	Discriminative AI	Classification of signals	Classifying normal signals, artifacts and drainages in high-resolution intracranial pressure monitoring data	5	TRIPOD/ TRIPOD+AI	Internal dataset	Internal dataset	0-100	0-100	N/A	Low	Low	High	High	High
**431**	Hur	2020	Korea	Discriminative AI	Predicting complications	Predicting diagnosis of congestive heart failure and unplanned cardiac surgery in patients with cardiovascular diseases	3&4	None	MIMIC III	N/A	10.000-100.000	N/A	N/A	Low	Unclear	Low	Unclear	Unclear
**432**	Hur	2021	Korea	Discriminative AI	Predicting complications	Prediction of delirium	5	TRIPOD/ TRIPOD+AI	Other type of dataset	MIMIC III	10.000-100.000	1.000 - 10.000	N/A	Low	Low	Low	Low	Low
**433**	Hur	2021	Korea	Discriminative AI	Improving mechanical ventilation	Predicting unplanned extubation in intensive care unit patients	3&4	TRIPOD/ TRIPOD+AI	Internal dataset	N/A	1.000-10.000	N/A	N/A	Low	Low	Unclear	High	High
**434**	Hüser	2024	Switzerland	Discriminative AI	Predicting complications; Improving mechanical ventilation; Predicting need for resource	Predicting respiratory failure, ventilation need and extubation readiness	5	None	HiRID	AmsterdamUMCdb	10.000-100.000	1.000 - 10.000	N/A	Low	Low	Low	High	High

© 2025 Berkhout WEM et al. *JAMA Network Open.*

**435**	Hutanu	2023	Romania	Discriminative AI	Predicting mortality	Predicting in-hospital mortality in critically ill SARS-CoV-2 patients	3&4	None	Internal dataset	N/A	100-1000	N/A	N/A	Low	Unclear	Low	High	High
**436**	Hwang	2023	Korea	Discriminative AI	Determining physiological values	Estimating respiratory rate in intensive care unit patients	5	None	Internal dataset	Other type of dataset	1.000-10.000	10.000 - 100.000	N/A	Low	Low	Low	Unclear	Unclear
**437**	Hyun	2020	South Korea	Discriminative AI	Classifying sub-populations	Identifying subgroups for patients admitted to the intensive care unit	3&4	None	Internal dataset	N/A	1.000-10.000	N/A	N/A	N/A	N/A	N/A	N/A	N/A
**438**	Ismukhamedova	2024	Kazakhstan	Discriminative AI	Diagnostic	Predicting diabetes in patients admitted to the intensive care unit	3&4	None	MIMIC III	N/A	10.000-100.000	N/A	N/A	Unclear	Unclear	Unclear	Unclear	Unclear
**439**	Itzhak	2023	Israel	Discriminative AI	Predicting complications	Predicting acute hypertensive episodes in critically ill patients	3&4	None	MIMIC III	N/A	1.000-10.000	N/A	N/A	Unclear	Low	High	Unclear	High
**440**	Iwase	2022	Japan	Discriminative AI	Predicting mortality; Predicting length of stay	Predicting intensive care unit mortality and length of stay	3&4	None	Internal dataset	N/A	10.000-100.000	N/A	N/A	Unclear	Low	Low	High	High
**441**	Iwashyna	2020	United States	Discriminative AI	Predicting mortality	Predicting 30-days mortality in intensive care unit patients	3&4	TRIPOD/ TRIPOD+AI	Internal dataset	N/A	100.000+	N/A	N/A	Unclear	Unclear	Low	Low	Unclear
**442**	Jagesar	2024	Netherlands	Discriminative AI	Predicting mortality	Predicting intensive care unit mortality	3&4	TRIPOD/ TRIPOD+AI	AmsterdamUMCdb	N/A	1.000-10.000	N/A	N/A	Unclear	Low	Low	Low	Unclear
**443**	Jajcay	2023	Slovakia	Discriminative AI	Predicting complications	Predicting cardiogenic shock for intensive cardiac care unit patients with acute coronary syndrome	3&4	None	MIMIC III	N/A	1.000-10.000	N/A	N/A	Unclear	Unclear	Low	Unclear	Unclear
**444**	Jamshidi	2021	Iran	Discriminative AI	Predicting mortality	Predicting mortality for COVID-19 patients in the intensive care unit	3&4	None	Internal dataset	N/A	100-1000	N/A	N/A	Low	Low	Low	High	High
**445**	Jang	2021	Korea	Discriminative AI	Classification of signals	Extracting electrocardiogram features for intensive care unit patients	3&4	None	Other type of dataset	N/A	No information	N/A	N/A	N/A	N/A	N/A	N/A	N/A
**446**	Jaotombo	2023	France	Discriminative AI	Predicting length of stay	Predicting length of stay of patients admitted to the intensive care unit	3&4	None	MIMIC III	N/A	10.000-100.000	N/A	N/A	Low	Unclear	Low	Unclear	Unclear
**447**	Jentzer	2021	United States	Discriminative AI	Predicting complications	Identifying left ventricular systolic dysfunction in intensive care unit patients	5	None	N/A	Internal dataset	N/A	100-1.000	N/A	Low	Low	Low	Low	Low
**448**	Jentzer	2022	United States	Discriminative AI	Classifying sub-populations	Identifying phenotypes in patients with cardiogenic shock in mixed cardiac intensive care unit	3&4	None	Internal dataset	N/A	1.000-10.000	N/A	N/A	N/A	N/A	N/A	N/A	N/A
**449**	Jeon	2021	South Korea	Discriminative AI	Predicting complications	Predicting sepsis in severe burn patients in the intensive care unit	3&4	None	Internal dataset	N/A	100-1000	N/A	N/A	Low	Unclear	Low	High	High
**450**	Jeon	2023	South Korea	Discriminative AI	Predicting mortality	Predicting intensive care unit mortality in pneumonia patients	3&4	TRIPOD/ TRIPOD+AI	Internal dataset	N/A	100-1000	N/A	N/A	Unclear	Unclear	Unclear	High	High
**451**	Jia	2021	United Kingdom	Discriminative AI	Predicting complications	Predicting weaning from mechanical ventilation for intensive care unit patients	3&4	None	MIMIC III	N/A	1.000-10.000	N/A	N/A	Low	Low	Unclear	High	High
**452**	Jiang	2021	China	Discriminative AI	Predicting complications	Predicting acute hypotensive episode (AHE) in critically ill patients	3&4	None	MIMIC II	N/A	1.000-10.000	N/A	N/A	Unclear	Low	High	Unclear	High
**453**	Jiang	2021	China	Discriminative AI	Predicting mortality	Predicting mortality for sepsis survivors with ICU readmission	3&4	None	MIMIC III	N/A	1.000-10.000	N/A	N/A	N/A	N/A	N/A	N/A	N/A
**454**	Jiang	2022	China	Discriminative AI	Predicting complications	Detecting pressure injuries	3&4	None	Internal dataset	N/A	100-1000	N/A	N/A	Unclear	Low	Low	Low	Unclear
**455**	Jiang	2022	China	Discriminative AI	Predicting mortality	Predicting in-hospital mortality for intensive care unit patients with fractures of the pelvis and acetabulum	3&4	None	MIMIC III	N/A	100-1000	N/A	N/A	Low	Unclear	High	High	High
**456**	Jiang	2022	China	Discriminative AI	Predicting need for resource	Predicting enteral nutrition support in neurosurgical intensive care patients	3&4	None	Internal dataset	N/A	100-1000	N/A	N/A	Low	Unclear	High	Low	Unclear

© 2025 Berkhout WEM et al. *JAMA Network Open.*

**457**	Jiang	2022	China	Discriminative AI	Predicting complications	Predicting sepsis-associated (severe) thrombocytopenia in intensive care unit patients	5	TRIPOD/ TRIPOD+AI	Internal dataset	MIMIC III	1.000-10.000	100-1.000	N/A	Unclear	Unclear	Low	Unclear	Unclear
**458**	Jiang	2022	China	Discriminative AI	Predicting complications	Predicting persistent acute kidney injury in postoperative intensive care unit patients	5	TRIPOD/ TRIPOD+AI	Internal dataset	MIMIC III	100-1000	1.000 - 10.000	N/A	Low	Unclear	Low	Unclear	Unclear
**459**	Jiang	2023	China	Discriminative AI	Predicting mortality	Predicting intensive care unit mortality	3&4	None	MIMIC III	N/A	1.000-10.000	N/A	N/A	Low	Low	Low	Low	Low
**460**	Jiang	2023	China	Discriminative AI	Predicting health improvement	Predicting furosemide responsiveness in patients with oliguric acute kidney injury	5	TRIPOD/ TRIPOD+AI	MIMIC IV	eICU-CRD	1.000-10.000	1.000 - 10.000	N/A	Unclear	Unclear	Unclear	Unclear	Unclear
**461**	Jiang	2023	United States	Discriminative AI	Classifying sub-populations	Identifying subphenotypes in critically ill patients with thrombocytopenia	3&4	STROBE	Internal dataset	N/A	1.000-10.000	N/A	N/A	N/A	N/A	N/A	N/A	N/A
**462**	Jiang	2023	China	Discriminative AI	Predicting complications	Predicting sepsis and septic death in the intensive care unit	3&4	None	MIMIC IV	N/A	1.000-10.000	N/A	N/A	Low	Low	Unclear	High	High
**463**	Jiang	2024	China	Discriminative AI	Predicting complications	Predicting acute respiratory distress syndrome in patients with sepsis in the intensive care unit	3&4	TRIPOD/ TRIPOD+AI	Internal dataset	N/A	100-1000	N/A	N/A	Low	Low	Low	High	High
**464**	Jiang	2024	United States	Discriminative AI	Classifying sub-populations	Predicting sepsis sub-phenotypes in intensive care unit patients	3&4	None	MIMIC IV	N/A	No information	N/A	N/A	Unclear	Low	Low	High	High
**465**	Jing	2021	United States	Discriminative AI	Classification of signals	Clustering continuous EEG records from critically ill patients	3&4	None	Internal dataset	N/A	No information	N/A	N/A	N/A	N/A	N/A	N/A	N/A
**466**	Jing	2022	China	Discriminative AI	Predicting mortality; Predicting complications	Diagnose sepsis and predict mortality at ICU admission	3&4	None	Internal dataset	N/A	0-100	N/A	N/A	Low	Low	Low	High	High
**467**	Job	2024	Australia	Discriminative AI (Reinforcement Learning)	Treatment recommendation	Treatment recommendation for patients with Acute Respiratory Distress Syndrome (ARDS) that has led to Sepsis	5	None	Other type of dataset	MIMIC III	0-100	100-1.000	N/A	N/A	N/A	N/A	N/A	N/A
**468**	Johansson	2024	Denmark	Discriminative AI	Predicting mortality	Predicting 30-day mortality in sepsis or septic shock patients admitted to the intensive care unit	3&4	None	Internal dataset	N/A	0-100	N/A	N/A	Low	Low	Low	High	High
**469**	Johnson	2017	United States	Discriminative AI	Predicting mortality	Predicting intensive care unit mortality	3&4	None	MIMIC III	N/A	10.000-100.000	N/A	N/A	Low	Low	Low	Unclear	Unclear
**470**	Johnson	2021	United Kingdom	Discriminative AI	Predicting mortality	Predicting mortality of intensive care unit patients	3&4	None	MIMIC III	N/A	10.000-100.000	N/A	N/A	Low	Low	Low	Unclear	Unclear
**471**	Johnsson	2020	Sweden	Discriminative AI	Predicting prognosis	Predicting functional outcome for out-of-hospital cardiac arrest survivors	3&4	None	Other type of dataset	N/A	100-1000	N/A	N/A	Low	Low	Low	High	High
**472**	Jonas	2022	Switzerland	Discriminative AI	Predicting mortality; Predicting prognosis	Predicting mortality and favorable outcome in intensive care unit patients with acute consciousness impairment	3&4	None	Other type of dataset	N/A	100-1000	N/A	N/A	Low	Low	Low	Unclear	Unclear
**473**	Jung	2022	Germany	Discriminative AI	Predicting mortality	Predicting 30-day mortality in elderly patients critically ill with COVID-19	5	None	Other type of dataset	Other type of dataset	No information	No information	N/A	Low	Low	Low	Unclear	Unclear
**474**	Jung	2023	South Korea	Discriminative AI	Predicting complications	Predicting serious intracranial hypertension in intensive care unit patients	5	None	Internal dataset	MIMIC III, Other type of dataset, Internal dataset	100-1000	1.000 - 10.000	N/A	Low	Unclear	High	Unclear	Unclear
**475**	Kaewprag	2015	United States	Discriminative AI	Predicting complications	Predicting pressure ulcers in the intensive care unit	3&4	None	Internal dataset	N/A	1.000-10.000	N/A	N/A	Low	Unclear	Low	High	High
**476**	Kamaleswaran	2020	United States	Discriminative AI	Predicting health improvement	Predicting volume responsiveness among sepsis patients at the intensive care unit	3&4	None	MIMIC III	N/A	10.000-100.000	N/A	N/A	Unclear	Unclear	Low	High	High

© 2025 Berkhout WEM et al. *JAMA Network Open.*

**477**	Kamaleswaran	2021	United States	Discriminative AI	Predicting complications	Predicting sepsis in liver-transplantation patients in the intensive care unit	3&4	None	Internal dataset	N/A	1.000-10.000	N/A	N/A	Unclear	Unclear	Unclear	High	High
**478**	Kamboj	2023	Canada	Discriminative AI	Determining physiological values	Predicting blood pressure after nitroglycerin infusion dose titration at the intensive care unit	5	None	eICU-CRD	eICU-CRD	No information	No information	N/A	Low	Low	Unclear	Unclear	Unclear
**479**	Kamel Rahimi	2023	Australia	Discriminative AI	Predicting complications	Predicting acute kidney injury in intensive care unit patients	3&4	TRIPOD/ TRIPOD+AI	MIMIC IV	N/A	10.000-100.000	N/A	N/A	Low	Low	High	High	High
**480**	Kamio	2023	Japan	Discriminative AI	Predicting mortality; Predicting need for resource	Predicting in-hospital mortality, initiation of acute renal replacement therapy and mechanical ventilation in patients with acute heart failure receiving furosemide the in intensive care unit	3&4	None	Internal dataset	N/A	100-1000	N/A	N/A	Low	Low	Low	High	High
**481**	Kang	2019	Korea	Discriminative AI	Predicting mortality	Predicting mortality in the intensive care unit for patients undergoing continuous renal replacement therapy	3&4	None	Internal dataset	N/A	1.000-10.000	N/A	N/A	Low	Low	Low	High	High
**482**	Kang	2022	Korea	Discriminative AI	Predicting complications	Predicting hemorrhage in the intensive care unit patients	5	None	MIMIC III	eICU-CRD, MIMIC IV	1.000-10.000	1.000 - 10.000	N/A	Low	Low	Low	High	High
**483**	Kang	2020	China	Discriminative AI	Predicting mortality	Predicting intensive care unit mortality	5	None	eICU-CRD	MIMIC III	100.000+	10.000 - 100.000	N/A	Low	Low	Low	Low	Low
**484**	Kara	2021	United States	Discriminative AI	Assessing videos and images	Identifying and localizing the position of an endotracheal tube relative to the carina	3&4	None	Other type of dataset	N/A	10.000-100.000	N/A	N/A	Unclear	Low	High	Unclear	High
**485**	Karabacak	2023	United States	Discriminative AI	Predicting complications	Predicting prolonged intensive care unit length of stay for patients with traumatic cervical spinal cord injury	3&4	TRIPOD/ TRIPOD+AI; JMIR Guidelines for Developing and Reporting Machine Learning Predictive Models in Biomedical Research	Other type of dataset	N/A	10.000-100.000	N/A	N/A	Low	Unclear	Low	Low	Unclear
**486**	Karabacak	2023	United States	Discriminative AI	Predicting complications	Predicting prolonged length of stay in the intensive care unit in acute traumatic epidural hematoma	3&4	TRIPOD/ TRIPOD+AI; JMIR Guidelines for Developing and Reporting Machine Learning Predictive Models in Biomedical Research	Other type of dataset	N/A	1.000-10.000	N/A	N/A	Low	Unclear	Low	Low	Unclear
**487**	Karabacak	2024	United States	Discriminative AI	Predicting complications	Predicting prolonged intensive care unit length of stay for patients with thoracolumbar spinal cord injuries	3&4	TRIPOD/ TRIPOD+AI; JMIR guidelines	Other type of dataset	N/A	10.000-100.000	N/A	N/A	Low	Low	Low	High	High
**488**	Karboub	2022	Morocco	Discriminative AI	Predicting prognosis	Predicting discharge	3&4	None	MIMIC III	N/A	100.000+	N/A	N/A	Low	High	Low	High	High
**489**	Karri	2022	Australia	Discriminative AI	Predicting need for resource	Predicting short-term requirements for invasive ventilation in patients with COVID-19 admitted to the ICU	3&4	STROBE	Internal dataset	N/A	100-1000	N/A	N/A	Low	Low	Low	Unclear	Unclear

© 2025 Berkhout WEM et al. *JAMA Network Open.*

**490**	Kashou	2021	United States	Discriminative AI	Assessing videos and images	Predicting left ventricular ejection fraction in cardiac intensive care unit patients	5	None	N/A	Internal dataset	N/A	1.000 - 10.000	N/A	Unclear	Low	Low	Low	Unclear
**491**	Katuwal	2016	United States	Discriminative AI	Predicting mortality	Predicting mortality at the intensive care unit	3&4	None	MIMIC II	N/A	1.000-10.000	N/A	N/A	Low	Unclear	Low	High	High
**492**	Katz	2022	Germany	Discriminative AI	Predicting mortality	Predicting mortality for intensive care unit patients with necrotizing soft tissue infections	3&4	None	Other type of dataset	N/A	100-1000	N/A	N/A	Low	Low	Low	Unclear	Unclear
**493**	Kaya	2023	Turkey	Discriminative AI	Predicting complications	Predicting sepsis for patients at the intensive care unit	3&4	None	Internal dataset	N/A	100-1000	N/A	N/A	Unclear	Unclear	Unclear	Unclear	Unclear
**494**	Ke	2022	China	Discriminative AI	Predicting mortality	Predicting in-hospital mortality in elderly patients with sepsis in the intensive care unit	3&4	TRIPOD/ TRIPOD+AI	MIMIC IV	N/A	10.000-100.000	N/A	N/A	Low	Unclear	Low	Unclear	Unclear
**495**	Keats	2024	United States	Discriminative AI	Classifying sub-populations	Classifying IV medication administration patterns in patients at the intensive care unit	3&4	STROBE	Internal dataset	N/A	100-1000	N/A	N/A	N/A	N/A	N/A	N/A	N/A
**496**	Kennedy	2023	United States	Generative AI (Large language model)	Assessing clinical notes	Capturing provider sentiment in intensive care unit notes	5	TRIPOD/ TRIPOD+AI	MIMIC III	Internal dataset	1.000-10.000	1.000 - 10.000	N/A	N/A	N/A	N/A	N/A	N/A
**497**	Kessler	2023	Germany	Discriminative AI	Predicting readmissions	Predicting readmission to the cardiovascular intensive care unit	3&4	None	MIMIC III	N/A	10.000-100.000	N/A	N/A	Unclear	Unclear	Low	Unclear	Unclear
**498**	Khadanga	2019	United States	Discriminative AI	Predicting mortality; Predicting length of stay; Assessing clinical notes	Predicting in-hospital mortality, modeling decompensation and forecasting length of stay	3&4	None	MIMIC III	N/A	10.000-100.000	N/A	N/A	Unclear	Unclear	Low	Unclear	Unclear
**499**	Khader	2023	Germany	Discriminative AI	Predicting mortality	Predicting mortality in the intensive care unit	3&4	None	MIMIC IV	N/A	1.000-10.000	N/A	N/A	Low	Low	Low	Unclear	Unclear
**500**	Khader	2023	Germany	Discriminative AI	Diagnosing	Predicting diagnosis of up to 25 different pathologic conditions in intensive care unit patients	3&4	None	MIMIC IV, Internal dataset	N/A	10.000-100.000	N/A	N/A	Unclear	Unclear	Low	High	High
**501**	Khodadadi	2023	Germany	Discriminative AI	Predicting mortality; Predicting readmissions	Predicting mortality and readmission at the intensive care unit	3&4	None	MIMIC III	N/A	10.000-100.000	N/A	N/A	Unclear	Unclear	Low	Unclear	Unclear
**502**	Khope	2022	India	Discriminative AI	Diagnosis	Predicting diagnosis	3&4	None	MIMIC III	N/A	10.000-100.000	N/A	N/A	Unclear	Unclear	Unclear	Unclear	Unclear
**503**	Khope	2022	India	Discriminative AI	Predicting prognosis	Predicting risk for intensive care unit patients	3&4	None	MIMIC III	N/A	No information	N/A	N/A	Unclear	High	Unclear	High	High
**504**	Kikutani	2023	Japan	Discriminative AI	Predicting complications	Predicting complications after extubation in intensive care unit patients	3&4	None	Internal dataset	N/A	0-100	N/A	N/A	Unclear	Low	Low	Unclear	Unclear
**505**	Kim	2021	Korea	Discriminative AI	Predicting mortality	Predicting 30-day mortality of patients receiving mechanical ventilation in the intensive care unit	5	None	Internal dataset	Internal dataset	10.000-100.000	1.000 - 10.000	N/A	Low	Low	Low	High	High
**506**	Kim	2022	United States	Discriminative AI	Predicting complications	Predicting delirium in intensive care unit patients	3&4	None	Internal dataset	N/A	0-100	N/A	N/A	Low	Low	Low	High	High
**507**	Kim	2022	United States	Discriminative AI	Predicting mortality; Predicting clinical score	Predicting survival, discharge location and neurological status for patients admitted to the intensive care unit with cardiac arrest	5	None	eICU-CRD	MIMIC III	1.000-10.000	0-100	N/A	Unclear	Unclear	Low	High	High
**508**	Kim	2022	South Korea	Discriminative AI	Predicting complications	Predicting delirium in intensive care unit	3&4	None	Internal dataset	N/A	1.000-10.000	N/A	N/A	Low	Low	Low	High	High
**509**	Kim	2022	Korea	Discriminative AI	Predicting complications	Predicting sepsis in intensive care unit patients	3&4	None	MIMIC III	N/A	10.000-100.000	N/A	N/A	Low	Low	Low	Low	Low
**510**	Kim	2023	Korea	Discriminative AI	Predicting complications	Predicting sepsis and septic shock in intensive care unit patients	3&4	TRIPOD/ TRIPOD+AI	Internal dataset	N/A	10.000-100.000	N/A	N/A	Low	Low	Low	Low	Low

© 2025 Berkhout WEM et al. *JAMA Network Open.*

**511**	Kim	2023	Korea	Discriminative AI	Predicting complications	Predicting cardiac arrest in intensive care unit patients	5	None	MIMIC IV	eICU-CRD	1.000-10.000	1.000 - 10.000	N/A	Low	Low	Low	High	High
**512**	Kim	2023	Korea	Discriminative AI	Improving mechanical ventilation	Predicting success of spontaneous breathing trials	5	None	Internal dataset	Internal dataset	No information	No information	N/A	Low	Unclear	Low	High	High
**513**	Kim	2023	Korea	Discriminative AI	Improving mechanical ventilation	Predicting successful weaning for patients at the intensive care unit	3&4	JMIR Guidelines for Developing and Reporting Machine Learning Predictive Models in Biomedical Research	MIMIC IV	N/A	10.000-100.000	N/A	N/A	Low	Low	Low	Unclear	Unclear
**514**	Kim	2024	Korea	Discriminative AI	Determining physiological values	Predicting vancomycin therapeutic drug monitoring levels in patients	5	None	Internal dataset	Internal, MIMIC IV	100-1000	1.000 - 10.000	N/A	Low	Low	Low	High	High
**515**	Kim	2024	Korea	Discriminative AI	Predicting complications	Predicting pressure ulcers in patients admitted to the intensive care unit	5	None	MIMIC IV	Internal dataset	10.000-100.000	1.000 - 10.000	N/A	Low	Unclear	High	Unclear	High
**516**	King	2023	United States	Generative AI (Large language model)	Predicting relevance of clinical information	Automatic checklist prompting using a voice-based digital assistant	3&4	None	Internal dataset	N/A	100-1000	N/A	N/A	N/A	N/A	N/A	N/A	N/A
**517**	King	2023	United States	Discriminative AI	Predicting need for resource	Predicting whether an intensive care unit patient will receive albumin	3&4	None	Internal dataset	N/A	100.000+	N/A	N/A	Unclear	Unclear	Unclear	Unclear	Unclear
**518**	Kline	2020	Canada	Discriminative AI	Predicting mortality	Predicting mortality in intensive care unit patients	3&4	None	MIMIC III	N/A	10.000-100.000	N/A	N/A	Low	Low	Low	Low	Low
**519**	Knox	2022	United States	Discriminative AI	Determining physiological values	Predicting left ventricular longitudinal strain in sepsis patients admitted to the intensive care unit	3&4	None	Internal dataset	N/A	100-1000	N/A	N/A	Unclear	Unclear	Low	Unclear	Unclear
**520**	Ko	2023	Korea	Discriminative AI	Predicting mortality	Predicting mortality at the intensive care unit	5	None	Internal dataset	Internal dataset, MIMIC III	1.000-10.000	1.000 - 10.000	N/A	Low	Low	Low	High	High
**521**	Kobayashi	2021	Japan	Discriminative AI	Determining physiological values	Determining pain in intensive care unit patients	3&4	None	Internal dataset	N/A	10.000-100.000	N/A	N/A	Low	Low	Low	Unclear	Unclear
**522**	Kobayashi	2023	Japan	Discriminative AI	Predicting complications	Predicting pain in critically ill patients	3&4	None	Internal dataset	N/A	10.000-100.000	N/A	N/A	Low	Low	Low	Unclear	Unclear
**523**	Kohn	2023	United States	Discriminative AI	Predicting mortality	Predicting intensive care unit mortality	5	None	Internal dataset	Internal dataset	No information	No information	N/A	Low	Low	Low	Low	Low
**524**	Kolisnyk	2023	Canada	Discriminative AI	Assessing videos and images	Predicting neurologic recovery in unresponsive patients with severe brain injury in the intensive care unit	3&4	None	Internal dataset	N/A	100-1000	N/A	N/A	Low	Low	Low	High	High
**525**	Kong	2020	China	Discriminative AI	Predicting mortality	Predicting hospital mortality in patients with sepsis in the intensive care unit	3&4	None	MIMIC III	N/A	10.000-100.000	N/A	N/A	Low	Low	Low	Low	Low
**526**	Kramer	2024	United States	Discriminative AI	Predicting prognosis	Predicting severity of intensive care unit patients	8	None	N/A	N/A	N/A	N/A	4146	N/A	N/A	N/A	N/A	N/A
**527**	Krieg	2024	Germany	Discriminative AI	Predicting complications	Predicting elevated intracranial pressure for patients with nonpenetrating traumatic brain injuries	3&4	STARD/ STARD-AI	Internal dataset	N/A	100-1000	N/A	N/A	Low	Low	Low	High	High
**528**	Krishnan	2019	India	Discriminative AI	Predicting mortality	Predicting intensive care unit mortality	3&4	None	MIMIC III	N/A	1.000-10.000	N/A	N/A	Low	Unclear	Low	Unclear	Unclear
**529**	Krishnan	2023	Georgia	Discriminative AI	Predicting complications	Detecting acute respiratory failure in critically ill sepsis patients	3&4	None	Internal dataset	N/A	0-100	N/A	N/A	Unclear	Low	Low	Unclear	Unclear
**530**	Kudo	2021	Japan	Discriminative AI	Classifying sub-populations	Examine phenotypes of sepsis with coagulopathy and thrombodulin treatment	5	None	Other type of dataset	Other type of dataset	1.000-10.000	1.000 - 10.000	N/A	Low	Low	Low	Low	Low

© 2025 Berkhout WEM et al. *JAMA Network Open.*

**531**	Kugler	2024	Germany	Discriminative AI	Predicting deterioration	Predicting COVID-19 severity in intensive care unit patients	3&4	None	Other type of dataset	N/A	100-1000	N/A	N/A	Low	Unclear	Unclear	Unclear	Unclear
**532**	Kurtz	2022	Brazil	Discriminative AI	Predicting mortality; Predicting length of stay	Predicting 30-day mortality and (prolonged) length of stay in patients admitted to the intensive care unit with acute stroke	3&4	None	Internal dataset	N/A	10.000-100.000	N/A	N/A	Low	Low	Low	Low	Unclear
**533**	Kwak	2021	China	Discriminative AI	Predicting need for resource	Predicting the need for vasopressors in the intensive care unit	3&4	None	MIMIC III, eICU-CRD	N/A	10.000-100.000	N/A	N/A	Low	Low	Low	Unclear	Unclear
**534**	La Cava	2021	United States	Discriminative AI	Predicting complications	Predicting septic shock in critical care patients	3&4	None	MIMIC III	N/A	10.000-100.000	N/A	N/A	Unclear	Low	Unclear	Unclear	Unclear
**535**	Lal	2020	United States	Discriminative AI	Predicting complications	Predicting the response to treatment in the intensive care unit for sepsis patients	6	None	No information	N/A	No information	N/A	N/A	Unclear	Unclear	Unclear	Unclear	Unclear
**536**	Lancia	2024	Netherlands	Discriminative AI	Predicting complications	Predicting healthcare-associated infections in patients admitted to the intensive care unit	3&4	None	Other type of dataset	N/A	1.000-10.000	N/A	N/A	Low	Low	Low	High	High
**537**	Lau	2024	China	Discriminative AI	Predicting mortality	Predicting hospital mortality for intensive care unit patients	3&4	None	Internal dataset	N/A	10.000-100.000	N/A	N/A	Low	Low	Low	Low	Low
**538**	Le	2020	United States	Discriminative AI	Predicting complications	Predicting acute respiratory distress syndrome in the intensive care unit	3&4	None	MIMIC III	N/A	1.000-10.000	N/A	N/A	Low	Low	Low	Unclear	Unclear
**539**	Le	2021	United States	Discriminative AI	Predicting complications	Predicting acute kidney injury in the intensive care unit	3&4	None	MIMIC III	N/A	10.000-100.000	N/A	N/A	Low	Unclear	Low	High	High
**540**	Lee	2020	Korea	Discriminative AI	Predicting mortality	Predicting in-hospital mortality for intensive care unit patients	3&4	None	MIMIC III	N/A	10.000-100.000	N/A	N/A	Low	Low	Low	Unclear	Unclear
**541**	Lee	2020	Korea	Discriminative AI	Predicting complications	Predicting sepsis in intensive care unit patients	5	None	Other type of dataset	No information	No information	No information	N/A	Unclear	Low	High	Unclear	High
**542**	Lee	2022	Korea	Discriminative AI	Predicting need for resource	Predicting the possibility of red blood cell transfusion for major medical diseases in the intensive care unit	3&4	None	MIMIC III	N/A	10.000-100.000	N/A	N/A	Low	Low	Low	High	High
**543**	Lee	2022	Taiwan	Discriminative AI	Predicting complications	Predicting hemorrhage in trauma patients admitted to the intensive care unit	3&4	None	Internal dataset	N/A	1.000-10.000	N/A	N/A	Low	Low	Low	High	High
**544**	Lee	2023	Korea	Discriminative AI	Predicting complications	Predicting in-hospital cardiac arrest in the intensive care unit	3&4	None	Other type of dataset	N/A	1.000-10.000	N/A	N/A	Unclear	Unclear	Unclear	Unclear	Unclear
**545**	Lee	2023	Korea	Discriminative AI	Predicting mortality	Predicting mortality in patients undergoing venovenous extracorporeal membrane oxygenation	5	None	Other type of dataset	Internal dataset	100-1000	0-100	N/A	Unclear	Low	Low	High	High
**546**	Lee	2024	Korea	Discriminative AI	Diagnosing	Predicting diagnosis of intensive care unit patients	3&4	None	MIMIC III	N/A	1.000-10.000	N/A	N/A	Unclear	Unclear	High	Unclear	High
**547**	Lei	2022	China	Discriminative AI	Predicting complications	Identify the biological signature of persistent critical illness	5	None	MIMIC III	Internal dataset	1.000-10.000	100-1.000	N/A	Low	Low	Low	Low	Low
**548**	Lei	2023	China	Discriminative AI	Predicting mortality	Predicting in-hospital mortality among critically ill patients with hip fracture	5	None	MIMIC III	eICU-CRD	100-1000	100-1.000	N/A	Low	Unclear	Low	High	High
**549**	Leitner	2022	United States	Discriminative AI	Determining physiological values	Estimating blood pressure in intensive care unit patients	3&4	None	MIMIC III	N/A	No information	N/A	N/A	Low	Low	Low	Unclear	Unclear
**550**	Lemmon	2023	Canada	Discriminative AI	Predicting mortality; Predicting complications; Predicting need for resource	Predicting long length of stay, in-hospital mortality, initiation of invasive ventilation and sepsis in intensive care unit patients	3&4	None	MIMIC IV	N/A	10.000-100.000	N/A	N/A	Low	Low	Unclear	Unclear	Unclear
**551**	Lengerich	2022	United States	Discriminative AI	Predicting mortality	Predicting in-hospital mortality for intensive care unit patients	3&4	None	MIMIC IV	N/A	10.000-100.000	N/A	N/A	Unclear	Low	Low	High	High

© 2025 Berkhout WEM et al. *JAMA Network Open.*

**552**	Lenivtceva	2022	Russia	Discriminative AI	Predicting mortality; Predicting complications	Predicting mortality and complications for patients after thoracic aneurysm surgeries	3&4	None	Internal dataset	N/A	100-1000	N/A	N/A	Unclear	Low	Low	Unclear	Unclear
**553**	Levi	2021	Italy	Discriminative AI	Predicting complications	Predicting rebleeding in patients with gastrointestinal bleeding	3&4	None	MIMIC II, eICU-CRD	N/A	10.000-100.000	N/A	N/A	Low	Low	High	Low	High
**554**	Li	2018	United States	Discriminative AI	Predicting complications	Predicting acute kidney injury in the intensive care unit	3&4	None	MIMIC III	N/A	10.000-100.000	N/A	N/A	Low	Low	High	Low	High
**555**	Li	2020	Canada	Discriminative AI	Predicting mortality; Diagnostic, Predicting an event	Predicting intensive care unit mortality and EHR codes	3&4	None	MIMIC III	N/A	No information	N/A	N/A	Unclear	Low	Low	Unclear	Unclear
**556**	Li	2020	Taiwan	Discriminative AI	Determining physiological values	Estimating systolic and diastolic blood pressure values in intensive care unit patients	3&4	None	MIMIC II	N/A	100.000+	N/A	N/A	Low	Low	Low	Low	Low
**557**	Li	2020	United States	Discriminative AI	Predicting complications	Predicting sepsis severity for intensive care unit patients	5	None	MIMIC III	eICU-CRD	10.000-100.000	10.000 - 100.000	N/A	Low	Low	High	High	High
**558**	Li	2021	China	Discriminative AI	Predicting health improvement; Provide dosage recommendations	Develop and validate a machine learning–based model to predict heparin treatment outcomes and to provide dosage recommendations to clinicians.	5	None	MIMIC III	Internal dataset	100-1000	1.000 - 10.000	N/A	Unclear	Low	Low	Low	Unclear
**559**	Li	2021	China	Discriminative AI	Predicting mortality	Predicting in-hospital mortality in intensive care unit patients with sepsis	3&4	None	MIMIC III	N/A	1.000-10.000	N/A	N/A	Low	Unclear	Low	Unclear	Unclear
**560**	Li	2022	China	Discriminative AI	Predicting mortality	Predicting in-hospital mortality for elderly patients with acute kidney disease	5	TRIPOD/ TRIPOD+AI	MIMIC IV	eICU-CRD	1.000-10.000	100-1.000	N/A	Unclear	Low	Low	Unclear	Unclear
**561**	Li	2022	United States	Discriminative AI	Predicting omittable lab test	Predicting unnecessary laboratory tests in intensive care units	5	None	N/A	Internal dataset	N/A	10.000 - 100.000	N/A	Low	Unclear	Unclear	Unclear	Unclear
**562**	Li	2022	China	Discriminative AI	Predicting complications	Predicting prolonged mechanical ventilation in patients with congestive heart failure	5	TRIPOD/ TRIPOD+AI	MIMIC IV	eICU-CRD	1.000-10.000	1.000 - 10.000	N/A	Low	Unclear	Low	Low	Unclear
**563**	Li	2022	China	Discriminative AI	Predicting mortality	Predicting mortality in intensive care unit patients with heart failure	3&4	None	eICU-CRD	N/A	1.000-10.000	N/A	N/A	Unclear	Low	Low	Unclear	Unclear
**564**	Li	2023	Japan	Discriminative AI	Predicting complications	Classifying brain-death/coma	3&4	None	Internal dataset	N/A	1.000-10.000	N/A	N/A	Low	Unclear	Low	Unclear	Unclear
**565**	Li	2023	China	Discriminative AI	Predicting mortality	Predicting in-hospital mortality in sepsis patients in the intensive care unit	3&4	None	MIMIC IV	N/A	10.000-100.000	N/A	N/A	Low	Low	Low	Low	Low
**566**	Li	2023	China	Discriminative AI	Predicting complications	Predicting acute kidney injury after cardiac surgery	5	TRIPOD/ TRIPOD+AI	Other type of dataset	Internal dataset, MIMIC-IV	1.000-10.000	1.000 - 10.000	N/A	Low	Low	Low	High	High
**567**	Li	2023	China	Discriminative AI	Predicting mortality	Predicting in-hospital mortality	5	TRIPOD/ TRIPOD+AI	MIMIC IV	eICU-CRD	1.000-10.000	1.000 - 10.000	N/A	Low	Low	Low	High	High
**568**	Li	2023	China	Discriminative AI	Diagnostic	Predicting heart failure in intensive care unit patients	3&4	None	MIMIC III	N/A	10.000-100.000	N/A	N/A	Unclear	Unclear	Low	Unclear	Unclear
**569**	Li	2023	China	Discriminative AI	Predicting mortality	Predicting mortality in critically ill patients with sepsis-associated acute kidney injury	3&4	None	MIMIC IV	N/A	1.000-10.000	N/A	N/A	Unclear	Low	Unclear	High	High
**570**	Li	2023	China	Discriminative AI	Predicting mortality	Predicting in-hospital mortality in patients with chronic kidney disease admitted to the intensive care unit	3&4	None	MIMIC IV	N/A	1.000-10.000	N/A	N/A	Low	Low	Low	High	High
**571**	Li	2023	China	Discriminative AI	Predicting complications	Predicting sepsis in trauma patients admitted to the intensive care unit	3&4	None	MIMIC IV	N/A	1.000-10.000	N/A	N/A	N/A	N/A	N/A	N/A	N/A

© 2025 Berkhout WEM et al. *JAMA Network Open.*

**572**	Li	2024	China	Discriminative AI	Predicting complications	Predicting postoperative delirium in cardiac valve surgery patients	3&4	TRIPOD/ TRIPOD+AI	Internal dataset	N/A	100-1000	N/A	N/A	Unclear	Low	Low	Unclear	Unclear
**573**	Li	2024	China	Discriminative AI	Improving mechanical ventilation	Predicting weaning outcome in mechanically ventilated patients in the intensive care unit	3&4	None	Internal dataset	N/A	0-100	N/A	N/A	Unclear	Unclear	Unclear	Unclear	Unclear
**574**	Li	2024	China	Discriminative AI	Predicting complications	Predicting acute kidney injury in intensive care unit patients	3&4	None	MIMIC IV	N/A	10.000-100.000	N/A	N/A	Low	Low	Low	Low	Low
**575**	Li	2024	China	Discriminative AI	Predicting mortality	Predicting in-hospital mortality in critically ill patients with congestive heart failure combined with chronic kidney disease	3&4	None	MIMIC IV	N/A	1.000-10.000	N/A	N/A	Low	Low	Low	Low	Low
**576**	Li	2024	China	Discriminative AI	Predicting mortality; Predicting complications	Predicting acute kidney disease in elderly patients with acute kidney injury in the intensive care unit and predicting mortality in elderly patients with acute kidney disease in the intensive care unit	5	JMIR Guidelines for Developing and Reporting Machine Learning Predictive Models in Biomedical Research	MIMIC IV	Internal dataset	1.000-10.000	100-1.000	N/A	Low	Low	Low	Low	Low
**577**	Li	2024	China	Discriminative AI	Predicting need for resource	Predicting intubation for intensive care unit patients	3&4	None	MIMIC III	N/A	1.000-10.000	N/A	N/A	Low	Unclear	High	High	High
**578**	Li	2020	China	Discriminative AI	Predicting complications	Predicting sepsis in intensive care unit patients	5	TRIPOD/ TRIPOD+AI	Other type of dataset	Other type of dataset	10.000-100.000	10.000 - 100.000	N/A	Low	Low	High	Low	Unclear
**579**	Liang	2022	China	Discriminative AI	Predicting complications	Predicting carbapenem-resistant gram-negative bacterial carriage in the intensive care unit	3&4	None	Internal dataset	N/A	1.000-10.000	N/A	N/A	Unclear	Unclear	Low	High	High
**580**	Liang	2022	China	Discriminative AI	Predicting complications	Predicting ventilator-associated pneumonia in critical care patients	3&4	None	MIMIC III	N/A	10.000-100.000	N/A	N/A	Unclear	Unclear	Low	Unclear	Unclear
**581**	Liang	2022	China	Discriminative AI	Predicting need for resource	Predicting noninvasive ventilation failure in intensive care unit patients	3&4	None	Internal dataset	N/A	1.000-10.000	N/A	N/A	Unclear	Unclear	Low	High	High
**582**	Liang	2024	China	Discriminative AI	Predicting complications	Predicting carbapenem-resistant gram-negative bacterial bloodstream infection in the intensive care unit	3&4	TRIPOD/ TRIPOD+AI	Internal dataset	N/A	100-1000	N/A	N/A	Unclear	Low	Low	High	High
**583**	Liang	2024	China	Discriminative AI	Predicting complications	Predicting successful weaning from renal replacement therapy in critically ill patients with acute kidney injury	3&4	None	Internal dataset	N/A	100-1000	N/A	N/A	Low	Low	Low	High	High
**584**	Liao	2022	Taiwan	Discriminative AI	Improving mechanical ventilation	Predicting successful weaning in respiratory care center patients	8	None	Internal dataset	N/A	100-1000	N/A	115	Low	Low	Low	High	High
**585**	Liao	2023	United States	Discriminative AI	Predicting mortality; Predicting complications	Predicting in-hospital mortality, acute respiratory failure and shock	5	None	MIMIC IV	eICU-CRD	10.000-100.000	10.000 - 100.000	N/A	Low	Unclear	Unclear	Unclear	Unclear
**586**	Lichtner	2021	Germany	Discriminative AI	Predicting mortality	Predicting mortality in critically ill non-COVID-19 viral pneumonia patients	5	None	Internal dataset	Internal dataset	No information	No information	N/A	Unclear	Unclear	Low	Unclear	Unclear
**587**	Liliopoulos	2024	United Kingdom	Discriminative AI	Predicting complications	Predicting sepsis in intensive care unit patients	3&4	None	Other type of dataset	N/A	100.000+	N/A	N/A	Low	Unclear	Low	Unclear	Unclear
**588**	Lilly	2023	United States	Discriminative AI	Predicting need for resource	Predicting episodes of intubation and administration of vasopressors	5	TRIPOD/ TRIPOD+AI	Internal dataset	eICU-CRD	10.000-100.000	1.000 - 10.000	N/A	Low	Low	Low	Low	Low
**589**	Lim	2024	Korea	Discriminative AI	Predicting mortality	Predicting mortality in critically ill patients	5	None	Internal dataset	AmsterdamUMCdb, MIMIC III, eICU-CRD	100.000+	100.000+	N/A	Low	Low	Low	Unclear	Unclear

© 2025 Berkhout WEM et al. *JAMA Network Open.*

**590**	Lin	2021	Taiwan	Discriminative AI	Improving mechanical ventilation	Predicting successful weaning among patients requiring prolonged mechanical ventilation	3&4	None	Internal dataset	N/A	100-1000	N/A	N/A	Low	Unclear	Low	Unclear	Unclear
**591**	Lin	2023	China	Discriminative AI (Reinforcement learning)	Treatment recommendation	Treatment plan generation for sepsis patients	3&4	None	MIMIC III	N/A	10.000-100.000	N/A	N/A	Unclear	Low	High	High	High
**592**	Lin	2023	Taiwan	Discriminative AI	Predicting complications; Determining physiological values	Predicting blood glucose concentration for patients in the intensive care unit	3&4	None	Internal dataset	N/A	1.000-10.000	N/A	N/A	Unclear	Low	Low	High	High
**593**	Lin	2024	China	Discriminative AI	Predicting complications	Predicting the outcome of acute respiratory distress syndrome of critically ill patients	5	None	eICU-CRD	MIMIC IV	1.000-10.000	1.000 - 10.000	N/A	Low	Unclear	High	High	High
**594**	Lin	2024	China	Discriminative AI	Predicting mortality	Predicting 30-day mortality of intensive care unit patients	5	None	MIMIC IV	Internal dataset	1.000-10.000	100-1.000	N/A	Unclear	Unclear	Low	Unclear	Unclear
**595**	Lin	2024	China	Discriminative AI	Predicting complications	Predicting risk of stroke in coronary artery disease patients receiving coronary revascularization admitted to the intensive care unit	3&4	None	MIMIC IV	N/A	1.000-10.000	N/A	N/A	N/A	N/A	N/A	N/A	N/A
**596**	Lin	2024	China	Discriminative AI	Predicting complications	Predicting acute kidney injury in critically ill patients with acute pancreatitis	3&4	None	MIMIC IV	N/A	1.000-10.000	N/A	N/A	Low	Unclear	Low	High	High
**597**	Lin	2024	Taiwan	Discriminative AI	Improving mechanical ventilation	Predicting weaning success in intensive care unit patients	8	None	Internal dataset	N/A	No information	N/A	131, 2405	N/A	N/A	N/A	N/A	N/A
**598**	Ling	2023	China	Discriminative AI	Predicting mortality	Predicting mortality of intensive care unit patients with postoperative acute respiratory distress syndrome	3&4	None	MIMIC III	N/A	1.000-10.000	N/A	N/A	Unclear	Unclear	Low	High	High
**599**	Lintu	2023	India	Discriminative AI	Predicting mortality	Predicting intensive care unit mortality	3&4	None	MIMIC II	N/A	1.000-10.000	N/A	N/A	Unclear	Unclear	Low	High	High
**600**	Liu	2018	United States	Discriminative AI	Predicting mortality	Predicting mortality of intensive care unit patients	3&4	None	eICU-CRD	N/A	100.000+	N/A	N/A	Low	Low	Low	High	High
**601**	Liu	2021	China	Discriminative AI	Predicting mortality	Predicting post-discharge mortality for intensive care unit patients	3&4	None	MIMIC III	N/A	10.000-100.000	N/A	N/A	Low	Low	Low	Low	Low
**602**	Liu	2021	China	Discriminative AI	Improving mechanical ventilation	Predicting weaning success in patients with sepsis	5	None	MIMIC IV	eICU-CRD	1.000-10.000	1.000 - 10.000	N/A	N/A	N/A	N/A	N/A	N/A
**603**	Liu	2021	United States	Discriminative AI	Predicting complications	Predicting sepsis at the intensive care unit	5	None	Internal dataset	eICU-CRD	100-1000	1.000 - 10.000	N/A	Low	Low	Low	Low	Low
**604**	Liu	2021	China	Discriminative AI	Predicting mortality	Predicting mortality in intensive care unit patients with Rhabdomyolysis	3&4	None	MIMIC III, eICU-CRD	N/A	100-1000	N/A	N/A	Low	Unclear	Low	High	High
**605**	Liu	2021	China	Discriminative AI	Predicting mortality	Predicting mortality of patients with acute kidney injury in the intensive care unit	3&4	None	eICU-CRD	N/A	1.000-10.000	N/A	N/A	Low	Low	High	High	High
**606**	Liu	2021	China	Discriminative AI	Predicting complications	Predicting tachycardia onset in intensive care unit patients	3&4	None	MIMIC III	N/A	1.000-10.000	N/A	N/A	Low	Low	Low	Low	Low
**607**	Liu	2021	United States	Discriminative AI	Predicting mortality	Predicting inpatient mortality in intensive care unit patients	3&4	None	MIMIC III	N/A	10.000-100.000	N/A	N/A	Low	Low	Low	Low	Low
**608**	Liu	2022	China	Discriminative AI	Predicting deterioration	Predicting multiple organ dysfunction syndrome in trauma patients	3&4	None	MIMIC III	N/A	1.000-10.000	N/A	N/A	Unclear	Unclear	Low	High	High
**609**	Liu	2022	China	Discriminative AI	Predicting mortality	Prediction of mortality for patients with acute pancreatitis	3&4	None	MIMIC III	N/A	100-1000	N/A	N/A	Unclear	Unclear	Low	High	High
**610**	Liu	2022	China	Discriminative AI	Predicting mortality	Predicting mortality based on the SOFA score	5	TRIPOD/ TRIPOD+AI	MIMIC III, eICU-CRD	Internal dataset, MIMIC IV	10.000-100.000	10.000 - 100.000	N/A	Unclear	Unclear	Low	Unclear	Unclear

© 2025 Berkhout WEM et al. *JAMA Network Open.*

**611**	Liu	2022	Taiwan	Discriminative AI	Improving mechanical ventilation	Predicting optimal timing of weaning from mechanical ventilation for intensive care unit patients	8	None	Internal dataset	N/A	1.000-10.000	N/A	167, 171	Low	Low	Low	Low	Low
**612**	Liu	2022	China	Discriminative AI	Predicting mortality	Predicting mortality in intensive care unit patients	3&4	None	MIMIC III	N/A	10.000-100.000	N/A	N/A	N/A	N/A	N/A	N/A	N/A
**613**	Liu	2022	China	Discriminative AI	Predicting mortality	Predicting mortality in critically ill patients with embolic stroke	5	TRIPOD/ TRIPOD+AI	MIMIC IV	eICU-CRD	1.000-10.000	100-1.000	N/A	Low	Low	Low	Unclear	Unclear
**614**	Liu	2022	China	Discriminative AI	Predicting complications	Predicting acute kidney injury in patients with heart failure at the intensive care unit	3&4	None	MIMIC IV	N/A	1.000-10.000	N/A	N/A	N/A	N/A	N/A	N/A	N/A
**615**	Liu	2023	China	Discriminative AI	Predicting mortality	Predicting mortality for older patients with multiple organ dysfunction syndrome	5	None	MIMIC III, eICU-CRD	AmsterdamUMCdb, MIMIC IV	10.000-100.000	1.000 - 10.000	N/A	Low	Low	High	Low	High
**616**	Liu	2023	China	Discriminative AI	Predicting complications	Predict sepsis for intensive care unit patients with acute pancreatitis	3&4	None	MIMIC III, MIMIC IV	N/A	1.000-10.000	N/A	N/A	Low	Low	High	Low	High
**617**	Liu	2023	China	Discriminative AI	Predicting complications	Predicting multiple organ dysfunction syndrome in intensive care unit patients	5	None	MIMIC III	MIMIC IV	100.000+	100.000+	N/A	Low	Unclear	Low	High	High
**618**	Liu	2023	United States	Discriminative AI	Treatment recommendation	Antibiotic treatment recommendation for sepsis patients admitted to the intensive care unit	5	None	MIMIC III	AmsterdamUMCdb	10.000-100.000	1.000 - 10.000	N/A	Low	Low	High	High	High
**619**	Liu	2023	Taiwan	Discriminative AI	Predicting complications	Predicting acute kidney injury in intensive care unit patients	3&4	None	MIMIC IV	N/A	10.000-100.000	N/A	N/A	Low	Unclear	Low	Unclear	Unclear
**620**	Liu	2023	China	Discriminative AI	Predicting mortality	Predicting in-hospital mortality for older adults in the intensive care unit	5	TRIPOD/ TRIPOD+AI	MIMIC IV, eICU-CRD	AmsterdamUMCdb, MIMIC IV, eICU-CRD	10.000-100.000	10.000 - 100.000	N/A	Low	Low	Unclear	Low	Unclear
**621**	Liu	2023	United States	Discriminative AI	Assessing videos and images	Predicting survival to hospital discharge and awakening from coma in comatose patients	3&4	None	Internal dataset	N/A	1.000-10.000	N/A	N/A	Unclear	Unclear	Low	Unclear	Unclear
**622**	Liu	2023	United States	Discriminative AI	Predicting complications	Predicting delirium in the intensive care unit	3&4	None	Internal dataset	N/A	100.000+	N/A	N/A	Unclear	Low	High	High	High
**623**	Liu	2023	United Kingdom	Discriminative AI	Classifying sub-populations	Clustering organ failure in patients admitted to the intensive care unit	3&4	None	MIMIC III	N/A	1.000-10.000	N/A	N/A	Low	Low	Low	High	High
**624**	Liu	2023	China	Discriminative AI	Classifying sub-populations	Identifying subphenotypes in heterogeneous patients with chronic critical illness in the intensive care unit	5	STROBE	MIMIC III, MIMIC IV	eICU-CRD	1.000-10.000	1.000 - 10.000	N/A	Low	Low	Low	Unclear	Unclear
**625**	Liu	2024	China	Discriminative AI	Determining physiological values	Predicting blood pressure for intensive care unit patients	3&4	None	MIMIC III	N/A	No information	N/A	N/A	Low	Low	High	Low	High
**626**	Liu	2024	China	Generative AI (Large language model)	Predicting complications	Predicting heart failure, respiratory failure and kidney failure in patients admitted to the intensive care unit	5	None	MIMIC IV	eICU-CRD	No information	1.000 - 10.000	N/A	Low	Unclear	Low	Low	Unclear
**627**	Liu	2024	China	Discriminative AI	Predicting mortality	Predicting in-hospital mortality in intensive care unit patients with sepsis-associated encephalopathy	5	TRIPOD/ TRIPOD+AI	MIMIC IV	eICU-CRD	1.000-10.000	1.000 - 10.000	N/A	N/A	N/A	N/A	N/A	N/A
**628**	Liu	2024	China	Discriminative AI	Predicting mortality	Predicting mortality of sepsis-induced coagulopathy in the intensive care unit	3&4	None	MIMIC IV	N/A	1.000-10.000	N/A	N/A	N/A	N/A	N/A	N/A	N/A
**629**	Liu	2024	China	Discriminative AI	Predicting readmissions	Predicting readmission in liver transplant patients	3&4	None	Internal dataset	N/A	100-1000	N/A	N/A	Low	Unclear	Unclear	High	High
**630**	Liu	2024	China	Discriminative AI (Reinforcement learning)	Treatment recommendation	Recommendation of heparin treatment for sepsis patients at the intensive care unit	5	None	MIMIC IV	eICU-CRD	100-1000	1.000 - 10.000	N/A	Low	Low	Low	Unclear	Unclear

© 2025 Berkhout WEM et al. *JAMA Network Open.*

						after abdominal surgery												
**631**	Lombardi	2022	Italy	Discriminative AI	Predicting complications	Predicting sepsis	3&4	None	MIMIC III	N/A	100-1000	N/A	N/A	Unclear	Low	Low	High	High
**632**	Long	2023	China	Discriminative AI	Determining physiological values	Estimating the blood pressure in intensive care unit patients	5	None	MIMIC II	MIMIC III	100.000+	100.000+	N/A	Unclear	Low	Low	High	High
**633**	Loo	2021	Malaysia	Discriminative AI	Improving mechanical ventilation	Quantifying the severity of asynchrony breathing during mechanical ventilation	5	None	Other type of dataset	Other type of dataset	100.000+	0-100	N/A	N/A	N/A	N/A	N/A	N/A
**634**	Lorenzoni	2021	Italy	Discriminative AI	Predicting mortality	Predicting intensive care unit mortality in COVID-19 patients	5	None	Internal dataset	Internal dataset	100-1000	100-1.000	N/A	Unclear	Low	Low	Unclear	Unclear
**635**	Loutati	2024	Israel	Discriminative AI	Predicting mortality	Predicting mortality for patients with acute coronary syndrome	3&4	None	Internal dataset	N/A	1.000-10.000	N/A	N/A	Unclear	Low	Low	High	High
**636**	Lozano Gómez	2023	Spain	Discriminative AI	Predicting mortality	Predicting mortality in acute coronary syndrome in the intensive care unit	3&4	None	Other type of dataset	N/A	10.000-100.000	N/A	N/A	Unclear	Low	Low	High	High
**637**	Lu	2021	China	Discriminative AI	Predicting mortality	Predicting 28-day mortality of patients with sepsis-induced coagulopathy	3&4	TRIPOD/ TRIPOD+AI	MIMIC III	N/A	1.000-10.000	N/A	N/A	Low	Low	High	Low	High
**638**	Lu	2021	United States	Discriminative AI (Reinforcement learning)	Treatment recommendation	Recommending stabilization treatment strategies for septic patients in the intensive care unit	3&4	None	MIMIC III	N/A	100.000+	N/A	N/A	N/A	N/A	N/A	N/A	N/A
**639**	Lu	2022	China	Discriminative AI	Predicting complications	Predicting sepsis-associated encephalopathy in patients with sepsis admitted to the intensive care unit	3&4	None	MIMIC IV	N/A	1.000-10.000	N/A	N/A	Low	Unclear	Low	Unclear	Unclear
**640**	Lu	2023	China	Discriminative AI	Predicting complications	Predicting postoperative atrial fibrillation after cardiac surgery	3&4	None	Internal dataset	N/A	1.000-10.000	N/A	N/A	Low	Unclear	Low	High	High
**641**	Lu	2024	China	Discriminative AI	Predicting complications	Predicting acute kidney injury in intensive care unit patients with cerebral infarction	5	None	Internal dataset	Internal dataset	1.000-10.000	100-1.000	N/A	Low	Low	High	High	High
**642**	Lucini	2020	Canada	Discriminative AI	Predicting complications	Predicting delirium in the intensive care unit	3&4	None	Internal dataset	N/A	No information	N/A	N/A	Low	Unclear	Low	High	High
**643**	Lucini	2023	Canada	Discriminative AI	Predicting complications	Predicting delirium for patients admitted to the intensive care unit	3&4	TRIPOD/ TRIPOD+AI	Internal dataset	N/A	100.000+	N/A	N/A	Low	Low	Low	Low	Low
**644**	Luethi	2022	Switzerland	Discriminative AI	Predicting mortality	Predicting mortality in critically ill patients	3&4	TRIPOD/ TRIPOD+AI; STROBE	Internal dataset	N/A	1.000-10.000	N/A	N/A	Low	Low	Low	Low	Unclear
**645**	Luo	2016	United States	Discriminative AI	Predicting mortality	Predicting mortality in intensive care unit patients	3&4	None	MIMIC II	N/A	No information	N/A	N/A	Low	Unclear	Low	Unclear	Unclear
**646**	Luo	2021	China	Discriminative AI	Predicting complications	Predicting deep venous thromboembolism for patients admitted to the neurological intensive care unit	3&4	None	Internal dataset	N/A	100-1000	N/A	N/A	Low	Low	Low	Low	Low
**647**	Luo	2021	China	Discriminative AI	Predicting mortality	Predicting mortality in intensive care unit patients	5	None	MIMIC III	eICU-CRD	10.000-100.000	1.000 - 10.000	N/A	Low	Low	Low	Low	Low
**648**	Luo	2021	China	Discriminative AI	Predicting complications	Predicting persistent acute kidney injury in septic patients with acute kidney injury	3&4	None	MIMIC III	N/A	1.000-10.000	N/A	N/A	Low	Low	Low	High	High
**649**	Luo	2022	China	Discriminative AI	Predicting mortality	Predicting hospital mortality risk for heart failure patients in intensive care unit	5	None	MIMIC III	eICU-CRD	1.000-10.000	1.000 - 10.000	N/A	Unclear	Low	Low	Unclear	Unclear
**650**	Luo	2022	China	Discriminative AI	Predicting mortality	Predicting mortality in critically ill patients with sepsis-associated acute kidney injury	5	None	MIMIC IV	eICU-CRD	10.000-100.000	1.000 - 10.000	N/A	Low	Low	Low	Low	Low
**651**	Luo	2023	China	Discriminative AI	Predicting complications	Predicting acute kidney injury in intensive care unit patients	3&4	None	MIMIC III	N/A	10.000-100.000	N/A	N/A	Unclear	Unclear	High	Low	High

© 2025 Berkhout WEM et al. *JAMA Network Open.*

**652**	Luo	2023	China	Discriminative AI	Assessing videos and images	Predicting mortality for critically ill patients at risk of hypoperfusion	3&4	None	Internal dataset	N/A	100-1000	N/A	N/A	Unclear	Unclear	Low	High	High
**653**	Lyra	2020	Australia	Discriminative AI	Assessing videos and images	Contactless monitoring of the vital signs of a patient	3&4	None	Internal dataset	N/A	0-100	N/A	N/A	Low	Low	Low	Unclear	Unclear
**654**	Lyu	2024	Switzerland	Discriminative AI	Predicting complications	Predicting acute kidney injury in critically ill patients	3&4	None	HiRID	N/A	No information	N/A	N/A	Unclear	Unclear	Unclear	Unclear	Unclear
**655**	Ma	2021	China	Discriminative AI	Classifying sub-populations	Identifying subclasses of septic shock for patients admitted to the intensive care unit	5	None	Internal dataset	eICU-CRD	1.000-10.000	1.000 - 10.000	N/A	Low	Unclear	Low	High	High
**656**	Ma	2023	China	Discriminative AI	Predicting mortality	Predict in-hospital mortality in solid cancer patients admitted to the ICU with sepsis	3&4	TRIPOD/ TRIPOD+AI	MIMIC IV	N/A	1.000-10.000	N/A	N/A	N/A	N/A	N/A	N/A	N/A
**657**	Ma	2023	China	Discriminative AI	Predicting complications	Predicting organ failure in intensive care unit patients	5	TRIPOD/ TRIPOD+AI	MIMIC III	eICU-CRD	100.000+	100.000+	N/A	Low	Low	High	High	High
**658**	Ma	2023	China	Discriminative AI	Predicting mortality	Predicting intensive care unit mortality	3&4	None	MIMIC III	N/A	1.000-10.000	N/A	N/A	Low	Low	Low	Low	Low
**659**	Maddali	2022	United States	Discriminative AI	Classifying sub-populations	Classifying acute respiratory distress syndrome subphenotypes in the intensive care unit	5	None	Other type of dataset	Other type of dataset	1.000-10.000	100-1.000	N/A	Unclear	Unclear	Unclear	Unclear	Unclear
**660**	Madden	2023	Ireland	Generative AI (Large language model)	Assessing clinical notes	Generating and analyzing summaries	5	None	N/A	Other type of dataset	N/A	0-100	N/A	N/A	N/A	N/A	N/A	N/A
**661**	Magunia	2021	Germany	Discriminative AI	Predicting mortality	Predicting mortality in intensive care unit patients with SARS-CoV-2	3&4	None	Internal dataset	N/A	1.000-10.000	N/A	N/A	Low	Unclear	Low	High	High
**662**	Mahardika	2023	Korea	Discriminative AI	Determining physiological values	Estimating blood pressure in intensive care unit patients	3&4	None	MIMIC III	N/A	10.000-100.000	N/A	N/A	Unclear	Low	Low	Unclear	Unclear
**663**	Mahbub	2022	United States	Discriminative AI	Predicting mortality	Predicting mortality in adult ICU patients	3&4	None	MIMIC III	N/A	10.000-100.000	N/A	N/A	N/A	N/A	N/A	N/A	N/A
**664**	Mahendra	2021	United States	Discriminative AI	Predicting mortality	Predicting mortality at the intensive care unit	5	None	Internal dataset	MIMIC III	10.000-100.000	10.000 - 100.000	N/A	Low	Low	Low	Unclear	Unclear
**665**	Maheshwarappa	2021	India	Discriminative AI	Assessing videos and images	Detecting ejection fraction in COVID-19 patients admitted to the intensive care unit	8	None	N/A	N/A	N/A	N/A	96	N/A	N/A	N/A	N/A	N/A
**666**	Mahmud	2022	Bangladesh	Discriminative AI	Predicting prognosis	Predicting severity of hypoxemia for intensive care unit patients	3&4	None	Other type of dataset	N/A	10.000-100.000	N/A	N/A	Unclear	Low	Low	Unclear	Unclear
**667**	Mahmud	2023	Qatar	Discriminative AI	Determining physiological values	Estimating arterial blood pressure waveforms	3&4	None	MIMIC III	N/A	100.000+	N/A	N/A	Unclear	Low	Low	High	High
**668**	Maiello	2021	Germany	Discriminative AI	Assessing videos and images	Quantifying lung volumes, aeration compartments and lung recruitability in intensive care unit patients	3&4	None	Other type of dataset	N/A	0-100	N/A	N/A	Unclear	Low	High	Unclear	High
**669**	Majhi	2023	India	Discriminative AI	Predicting mortality	Predicting mortality in intensive care unit patients	3&4	None	Other type of dataset	N/A	10.000-100.000	N/A	N/A	Unclear	Low	Low	Low	Unclear
**670**	Majhi	2023	India	Discriminative AI	Predicting mortality	Predicting intensive care unit mortality	3&4	None	Other type of dataset, MIMIC III	N/A	10.000-100.000	N/A	N/A	Low	Unclear	Low	Unclear	Unclear
**671**	Malayeri	2022	Iran	Discriminative AI	Determining physiological values	Estimating cuffless blood pressure	3&4	None	MIMIC II	N/A	No information	N/A	N/A	Unclear	Unclear	Low	Unclear	Unclear
**672**	Mamandipoor	2021	Germany	Discriminative AI	Predicting mortality	Predicting mortality of mechanically ventilated patients admitted to the intensive care unit	3&4	STROBE	Other type of dataset	N/A	10.000-100.000	N/A	N/A	Low	Unclear	Low	Low	Unclear
**673**	Mamandipoor	2022	Italy	Discriminative AI	Determining physiological values	Predicting blood lactate values in patients admitted to the intensive care unit	5	STROBE	eICU-CRD	MIMIC III	10.000-100.000	10.000 - 100.000	N/A	Low	Unclear	Low	Unclear	Unclear
**674**	Mandel	2022	Germany	Discriminative AI	Predicting complications	Predicting hemodynamic and pulmonary	3&4	None	Internal dataset	N/A	1.000-10.000	N/A	N/A	Low	Low	High	Unclear	High

© 2025 Berkhout WEM et al. *JAMA Network Open.*

						decompensation in patients admitted to the intensive care unit												
**675**	Manni	2022	United States	Discriminative AI	Predicting complications	Predicting hemodynamic instability on intensive care unit patients	3&4	None	eICU-CRD	N/A	10.000-100.000	N/A	N/A	Unclear	Unclear	Unclear	High	High
**676**	Mansour	2022	United States	Discriminative AI	Assessing videos and images	Predicting hypoxic-ischemic brain injury after cardiac arrest	3&4	None	Internal dataset	N/A	0-100	N/A	N/A	Low	Low	High	Low	High
**677**	Mansouri	2022	Iran	Discriminative AI	Predicting mortality	Early mortality prediction of intensive care unit patients	3&4	None	MIMIC III	N/A	10.000-100.000	N/A	N/A	Low	Unclear	Low	High	High
**678**	Mantena	2022	United States	Discriminative AI	Predicting complications	Predicting hypoglycemia in critically ill patients	3&4	None	eICU-CRD	N/A	10.000-100.000	N/A	N/A	Unclear	Low	High	Low	High
**679**	Mao	2022	Taiwan	Discriminative AI	Assessing videos and images	Detecting malposition of the endotracheal tube in intensive care unit patients	3&4	None	Internal dataset	N/A	1.000-10.000	N/A	N/A	Low	Low	Low	High	High
**680**	Mao	2023	China	Discriminative AI	Predicting mortality	Predicting mortality in patients with spontaneous intracerebral hemorrhage in the intensive care unit	5	None	MIMIC IV	Other type of dataset	1.000-10.000	100-1.000	N/A	Unclear	Low	High	Unclear	High
**681**	Maray	2022	Spain	Discriminative AI	Predicting complications	Predicting Linezolid induced thrombocytopenia in intensive care unit patients	3&4	None	MIMIC III	N/A	100-1000	N/A	N/A	Low	Unclear	Unclear	High	High
**682**	Martinez	2020	United States	Discriminative AI	Predicting mortality	Predicting intensive care unit mortality	3&4	None	MIMIC III	N/A	No information	N/A	N/A	Unclear	Low	Low	High	High
**683**	Martinez	2022	China	Discriminative AI	Predicting mortality	Predicting mortality in COVID-19 critically ill patients	5	None	Internal dataset	Internal dataset	0-100	0-100	N/A	Unclear	Low	Low	Unclear	Unclear
**684**	Martinez	2022	United States	Discriminative AI	Determining physiological values	Estimating blood pressure for patients at the intensive care unit	3&4	None	MIMIC III	N/A	No information	N/A	N/A	Low	Low	Low	Unclear	Unclear
**685**	Martinez	2023	Canada	Discriminative AI	Predicting prognosis	Classifying severity of sepsis and septic shock in intensive care unit patients	3&4	None	Internal dataset	N/A	100-1000	N/A	N/A	Unclear	Low	Low	High	High
**686**	Martínez-Agüero	2022	Spain	Discriminative AI	Predicting complications	Predicting antimicrobial multidrug resistance in the intensive care unit	3&4	None	Internal dataset	N/A	100-1000	N/A	N/A	Unclear	Low	Low	Unclear	Unclear
**687**	Mashraqi	2022	Saudi Arabia	Discriminative AI	Predicting complications	Predicting liver damage in COVID-19 patients admitted to the intensive care unit	3&4	None	Internal dataset	N/A	100-1000	N/A	N/A	Unclear	Low	High	High	High
**688**	Mataczynski	2022	Poland	Discriminative AI	Classification of signals	Classifying intracranial pressure pulse waveforms in intensive care unit patients	3&4	None	Internal dataset	N/A	10.000-100.000	N/A	N/A	Low	Low	High	High	High
**689**	Mathis	2022	United States	Discriminative AI	Predicting deterioration	Predicting postoperative deterioration in cardiac surgery patients at the intensive care unit	3&4	STROBE, JMIR Guidelines for Developing and Reporting Machine Learning Predictive Models in Biomedical Research	Internal dataset	N/A	1.000-10.000	N/A	N/A	Low	Unclear	Unclear	Unclear	Unclear
**690**	Mattia	2022	France	Discriminative AI	Diagnostic	Discriminating patients in postanoxic coma from controls	3&4	None	Internal dataset	N/A	0-100	N/A	N/A	Low	Low	Low	Unclear	Unclear
**691**	Maviglia	2022	Italy	Discriminative AI	Classifying sub-populations	Classifying patient admitted to the intensive care unit receiving antibiotic therapy	3&4	None	Internal dataset	N/A	1.000-10.000	N/A	N/A	N/A	N/A	N/A	N/A	N/A
**692**	Mbous	2023	United States	Discriminative AI	Predicting mortality; Predicting length of stay; Predicting need for resource	Predicting mortality, length of stay and need for mechanical ventilation	3&4	STROBE	Internal dataset	N/A	100-1000	N/A	N/A	Low	Low	Low	Unclear	Unclear

© 2025 Berkhout WEM et al. *JAMA Network Open.*

**693**	Mears	2024	United States	Discriminative AI	Assessing videos and images	Guiding during cardiac point-of-care ultrasound in the neurologic intensive care unit	8	None	N/A	N/A	N/A	N/A	153	N/A	N/A	N/A	N/A	N/A
**694**	Mejia-Mejia	2021	United Kingdom	Discriminative AI	Determining physiological values, Classification of signals	Classifying blood pressure in intensive care unit patients	3&4	None	MIMIC II	N/A	1.000-10.000	N/A	N/A	Unclear	Low	High	Unclear	High
**695**	Menguy	2023	France	Discriminative AI	Improving mechanical ventilation	Predicting extubation success in intensive care unit patients	3&4	None	Other type of dataset	N/A	100-1000	N/A	N/A	Low	Unclear	Low	High	High
**696**	Mercier	2023	Canada	Discriminative AI	Predicting complications	Predicting diuretic resistance in intensive care unit patients	5	None	eICU-CRD	MIMIC III	10.000-100.000	1.000 - 10.000	N/A	Low	Unclear	Low	Low	Unclear
**697**	Miao	2024	China	Discriminative AI	Predicting readmissions	Predicting readmission risk in intracerebral hemorrhage patients at the intensive care unit	3&4	None	MIMIC IV, MIMIC III	N/A	1.000-10.000	N/A	N/A	Low	Low	Low	High	High
**698**	Michalak	2021	United States	Discriminative AI	Classification of signals	Classifying intensive care unit patients' sedation depth	3&4	None	Internal dataset	N/A	100-1000	N/A	N/A	Unclear	Low	High	High	High
**699**	Mirtchouk	2021	United States	Discriminative AI	Predicting mortality	Predicting mortality in intensive care unit patients	3&4	None	MIMIC III	N/A	No information	N/A	N/A	Unclear	Unclear	Low	Unclear	Unclear
**700**	Mirzakhani	2022	Iran	Discriminative AI	Predicting mortality	Predicting intensive care unit mortality	5	None	Internal dataset	No information	100-1000	No information	N/A	Low	Low	Low	High	High
**701**	Mitra	2021	United States	Discriminative AI	Improving prognostic models/risk scoring system	Assess NLP's effectiveness in identifying SBDH in medical records and its role in evaluating risk factors for nonfatal overdoses in ICU patients.	3&4	None	MIMIC III	N/A	10.000-100.000	N/A	N/A	N/A	N/A	N/A	N/A	N/A
**702**	Mo	2023	China	Discriminative AI	Predicting complications	Predicting acute respiratory distress syndrome in patients admitted to the intensive care unit with community-acquired pneumonia	3&4	None	Internal dataset	N/A	1.000-10.000	N/A	N/A	Unclear	Low	Low	High	High
**703**	Moghadam	2020	United States	Discriminative AI	Predicting complications	Predicting hypotensive events in the intensive care unit	3&4	None	MIMIC III	N/A	100.000+	N/A	N/A	Unclear	Unclear	High	Low	High
**704**	Moghadam	2021	United States	Discriminative AI	Predicting complications	Predicting hypotension in the intensive care unit	3&4	None	MIMIC III	N/A	10.000-100.000	N/A	N/A	Unclear	Low	High	Unclear	High
**705**	Moghadam	2023	United States	Discriminative AI	Predicting complications	Predicting hypotension in the intensive care unit	3&4	None	MIMIC III	N/A	100.000+	N/A	N/A	Unclear	Low	High	Unclear	High
**706**	Mohammed	2021	United States	Discriminative AI	Predicting complications	Predicting sepsis in intensive care unit patients	3&4	None	Internal dataset	N/A	1.000-10.000	N/A	N/A	Low	Low	Low	High	High
**707**	Mohedano-Munoz	2023	Spain	Discriminative AI	Predicting complications	Predicting multidrug-resistant bacteria in intensive care unit patients	3&4	None	Internal dataset	N/A	No information	N/A	N/A	Unclear	Unclear	Low	Unclear	Unclear
**708**	Mollura	2020	Italy	Discriminative AI	Predicting complications	Predicting septic shock development in intensive care unit patients	3&4	None	MIMIC III	N/A	100-1000	N/A	N/A	Unclear	High	Low	High	High
**709**	Mollura	2021	United States	Discriminative AI	Predicting complications	Predicting sepsis	3&4	None	MIMIC III	N/A	100-1000	N/A	N/A	N/A	N/A	N/A	N/A	N/A
**710**	Mollura	2022	United Kingdom	Discriminative AI (Reinforcement learning)	Treatment recommendation	Predict the optimal dosage of fluids and/or vasopressors in order to improve 90-day patients' survival	3&4	None	MIMIC III	N/A	10.000-100.000	N/A	N/A	Low	Low	Low	High	High
**711**	Mollura	2024	Italy	Discriminative AI	Predicting mortality	Predicting mortality in intensive care unit patients with systematic inflammatory response syndrome or sepsis	3&4	None	Internal dataset	N/A	1.000-10.000	N/A	N/A	Low	Low	Low	Unclear	Unclear
**712**	Momo	2024	Belgium	Discriminative AI	Predicting length of stay	Predicting length of stay for patients admitted to the intensive care unit	3&4	None	eICU-CRD	N/A	100.000+	N/A	N/A	Low	Unclear	Low	Unclear	Unclear

© 2025 Berkhout WEM et al. *JAMA Network Open.*

**713**	Montazeri	2023	Iran	Discriminative AI	Predicting mortality; Predicting length of stay	Predicting length of stay and mortality rate	3&4	TRIPOD/ TRIPOD+AI	Internal dataset	N/A	100-1000	N/A	N/A	Unclear	Low	Unclear	High	High
**714**	Montomoli	2021	Italy	Discriminative AI	Predicting prognosis	Predicting the change in patient's Sequential Organ Failure Assessment score at the intensive care unit	3&4	None	Internal dataset	N/A	100-1000	N/A	N/A	Unclear	Unclear	Unclear	Unclear	Unclear
**715**	Mora-Jimenez	2021	Spain	Discriminative AI	Predicting complications	Predicting development of a multi-drug resistance germ during intensive care unit admission	3&4	None	Internal dataset	N/A	1.000-10.000	N/A	N/A	Unclear	Low	Unclear	High	High
**716**	Morales	2024	United States	Discriminative AI	Assessing clinical notes	Adjudicating bilateral infiltrates, risk factors and cardiac failure in acute hypoxemic respiratory failure requiring mechanical ventilation at the intensive care unit	5	None	Internal dataset	MIMIC III	1.000-10.000	100-1.000	N/A	N/A	N/A	N/A	N/A	N/A
**717**	Morales Chacon	2022	Cuba	Discriminative AI	Predicting mortality	Predicting mortality for COVID-19 patients with Neurological disease	3&4	None	Internal dataset	N/A	1.000-10.000	N/A	N/A	Low	Unclear	High	Unclear	High
**718**	Moridani	2015	Iran	Discriminative AI	Predicting mortality	Predicting mortality for cardiovascular patients admitted to the intensive care unit	3&4	None	MIMIC II	N/A	No information	N/A	N/A	Unclear	Unclear	Low	High	High
**719**	Morik	1999	Germany	Discriminative AI	Predicting mortality	Changes in patient vital signs	3&4	None	Internal dataset	N/A	100-1000	N/A	N/A	Unclear	Low	Low	High	High
**720**	Morisson	2023	France	Discriminative AI	Predicting the need for resource	Predict veno-arterial ECMO implantation after post-cardiotomy low cardiac output syndrome	5	TRIPOD/ TRIPOD+AI	Internal dataset	Internal dataset	100-1000	100-1.000	N/A	Unclear	Low	Low	High	High
**721**	Moro	2024	United States	Discriminative AI	Predicting mortality	Predicting mortality in lung transplant patients in the intensive care unit	3&4	None	Other type of dataset	N/A	10.000-100.000	N/A	N/A	Low	Low	Low	High	High
**722**	Moser	2022	Switzerland	Discriminative AI	Predicting mortality	Predicting mortality for patients admitted to the intensive care unit	5	TRIPOD/ TRIPOD+AI	Internal dataset	Internal dataset	No information	No information	N/A	Unclear	Low	Low	High	High
**723**	Mroueh	2022	France	Discriminative AI	Predicting complications	Detection of atrial fibrillation	3&4	None	MIMIC III	N/A	100-1000	N/A	N/A	Low	Low	Low	Unclear	Unclear
**724**	Mugisha	2022	Japan	Discriminative AI	Predicting mortality	Predicting mortality for ICU patients	3&4	None	MIMIC III	N/A	10.000-100.000	N/A	N/A	N/A	N/A	N/A	N/A	N/A
**725**	Mulkey	2023	United States	Discriminative AI	Predicting complications	Predicting delirium in mechanically ventilated critically ill older patients	3&4	None	Internal dataset	N/A	No information	N/A	N/A	Low	Low	Low	Unclear	Unclear
**726**	Murray	2024	United States	Discriminative AI	Predicting mortality	Predicting mortality for ICU patients	5	CONSORT/ CONSORT-AI-AI	Internal dataset	Other type of dataset	100-1000	1.000 - 10.000	N/A	Low	Low	Low	Low	Low
**727**	Musalia	2023	United Kingdom	Discriminative AI	Assessing videos and images	Speech/phrase recognition for patients with tracheostomies	7	DECIDE-AI	Internal dataset	N/A	0-100	N/A	N/A	N/A	N/A	N/A	N/A	N/A
**728**	Mutlu	2024	Turkey	Discriminative AI	Predicting complications	Predicting sepsis in intensive care unit patients	3&4	None	Other type of dataset	N/A	1.000-10.000	N/A	N/A	Low	Unclear	Unclear	Unclear	Unclear
**729**	Nair	2023	United States	Discriminative AI	Determining physiological values	Computing the intracranial pressure	3&4	None	MIMIC III	N/A	100.000+	N/A	N/A	Low	Low	Low	Unclear	Unclear
**730**	Nair	2024	United States	Discriminative AI	Determining physiological values	Generating intracranial pressure waveforms	3&4	None	MIMIC III	N/A	0-100	N/A	N/A	Low	Low	High	Low	High
**731**	Nakanishi	2024	Japan	Discriminative AI	Predicting complications	Predicting acute hypotensive events in intensive care unit patients	5	TRIPOD/ TRIPOD+AI	Internal dataset	MIMIC III	1.000-10.000	1.000 - 10.000	N/A	Unclear	Low	Low	Unclear	Unclear
**732**	Nallabasannagaar	2020	United States	Discriminative AI	Predicting mortality; Predicting length of stay	Predicting in-hospital mortality and length of stay longer than or equal to 7 days for intensive care unit patients	3&4	None	MIMIC III	N/A	10.000-100.000	N/A	N/A	Low	Low	Low	Unclear	Unclear
**733**	Naqvi	2022	Pakistan	Discriminative AI	Predicting complications	Predicting risk of liver failure in the intensive care unit	5	None	MIMIC III	eICU-CRD	No information	10.000 - 100.000	N/A	Unclear	Unclear	Low	High	High

© 2025 Berkhout WEM et al. *JAMA Network Open.*

**734**	Narula	2021	Switzerland	Discriminative AI	Classification of signals	Detecting bursts in EEG signals in intensive care unit patients	3&4	None	Internal dataset	N/A	No information	N/A	N/A	Unclear	Low	High	Low	Unclear
**735**	Nayebi	2023	United States	Discriminative AI	Predicting mortality	Predicting mortality in the intensive care unit	3&4	None	MIMIC III	N/A	10.000-100.000	N/A	N/A	Unclear	Unclear	Low	High	High
**736**	Nazir	2021	France	Discriminative AI	Assessing videos and images	Analysing intensive care unit patient's chest wall morphological changes	5	None	N/A	Internal dataset	N/A	No information	N/A	Unclear	Low	High	Unclear	High
**737**	Nemeth	2021	United States	Discriminative AI	Predicting complications	Predicting risk of shock in intensive care unit patients	3&4	None	MIMIC	N/A	10.000-100.000	N/A	N/A	Unclear	Low	Low	High	High
**738**	Nesaragi	2020	India	Discriminative AI	Predicting complications	Predicting sepsis in intensive care unit patients	3&4	None	Other type of dataset	N/A	10.000-100.000	N/A	N/A	Unclear	Low	Unclear	Unclear	Unclear
**739**	Nesaragi	2021	India	Discriminative AI	Predicting complications	Predicting the onset of sepsis in intensive care patients	3&4	None	Other type of dataset	N/A	10.000-100.000	N/A	N/A	Unclear	Low	High	Unclear	High
**740**	Nesaragi	2021	India	Discriminative AI	Predicting complications	Predicting sepsis in intensive care unit patients	3&4	None	Other type of dataset	N/A	10.000-100.000	N/A	N/A	Unclear	Low	High	Unclear	High
**741**	Nestor	2019	Canada	Discriminative AI	Predicting mortality; Predicting length of stay	Predicting mortality and length of stay in intensive care unit patients	5	None	MIMIC III	MIMIC III	No information	No information	N/A	Low	Low	Low	Unclear	Unclear
**742**	Neyra	2023	United States	Discriminative AI	Predicting mortality; Predicting complications	Predicting mortality and major adverse kidney events for patients admitted to the intensive care unit with acute kidney injury	5	None	Internal dataset	Internal dataset	1.000-10.000	1.000 - 10.000	N/A	Low	Low	Low	Low	Low
**743**	Ng	2021	Malaysia	Discriminative AI	Improving mechanical ventilation	Calculating the asynchronous index for ventilated patients admitted to the intensive care unit	5	None	N/A	Internal dataset	N/A	100.000+	N/A	N/A	N/A	N/A	N/A	N/A
**744**	Nguyen-Duc	2022	Australia	Discriminative AI	Assessing videos and images	Predicting clinical workload and patient activity in intensive care unit patients	3&4	None	Internal dataset	N/A	1.000-10.000	N/A	N/A	Unclear	Low	High	Unclear	High
**745**	Nie	2020	China	Discriminative AI	Predicting mortality	Predicting mortality in cerebral hemorrhage patients in the intensive care unit	3&4	None	MIMIC III	N/A	100-1000	N/A	N/A	Unclear	Unclear	Unclear	Unclear	Unclear
**746**	Nie	2022	China	Discriminative AI	Predicting complications	Predicting circulatory failure status in intensive care unit patients	3&4	None	HiRID	N/A	No information	N/A	N/A	Low	Unclear	Unclear	High	High
**747**	Nie	2024	China	Discriminative AI	Predicting mortality; Predicting length of stay	Predicting in-hospital mortality and length of stay for intensive care unit patients	3&4	None	MIMIC IV	N/A	10.000-100.000	N/A	N/A	Low	Unclear	Low	High	High
**748**	Ning	2023	China	Discriminative AI	Predicting mortality	Predicting intensive care unit mortality	3&4	None	MIMIC IV	N/A	1.000-10.000	N/A	N/A	Low	Low	Low	Unclear	Unclear
**749**	Nistal-Nuño	2022	Spain	Discriminative AI	Predicting mortality	Predicting in-hospital and 1-year mortality for patients admitted to the intensive care unit	3&4	None	MIMIC III	N/A	1.000-10.000	N/A	N/A	Low	Unclear	Low	High	High
**750**	Nistal-Nuño	2023	Spain	Discriminative AI	Predicting mortality	Predicting in-hospital mortality for intensive care unit patients with respirator and/or intrathoracic neoplasm	3&4	None	MIMIC III	N/A	1.000-10.000	N/A	N/A	Low	Low	Low	High	High
**751**	Nistal-Nuño	2022	Spain	Discriminative AI	Predicting mortality	Predicting mortality for patients admitted to the Cardiac Surgery Recovery Unit and Coronary Care Unit	3&4	None	MIMIC IV	N/A	10.000-100.000	N/A	N/A	Low	Low	Low	High	High
**752**	Niu	2023	China	Discriminative AI	Predicting readmissions	Predicting readmission to the intensive care unit	3&4	None	MIMIC III	N/A	1.000-10.000	N/A	N/A	Low	Unclear	Low	Unclear	Low
**753**	Nori	2017	Japan	Discriminative AI	Predicting mortality	Predicting intensive care unit mortality	3&4	None	Internal dataset	N/A	100-1000	N/A	N/A	Low	Low	Low	High	High
**754**	Nourelahi	2022	Iran	Discriminative AI	Predicting prognosis	Predicting favorable or unfavorable outcomes after 6 months in	3&4	None	Internal dataset	N/A	1.000-10.000	N/A	N/A	Unclear	Low	Low	High	High

© 2025 Berkhout WEM et al. *JAMA Network Open.*

						severe traumatic brain injury patients												
**755**	Nowak	2023	Germany	Discriminative AI	Assessing videos and images	Diagnosing X-ray images of intensive care unit patients	3&4	None	Internal dataset	N/A	10.000-100.000	N/A	N/A	Low	Low	High	Unclear	High
**756**	Nowakowska	2023	Poland	Discriminative AI	Predicting complications	Predicting depression and delirium in cardiovascular diseased patients	3&4	None	Internal dataset	N/A	100-1000	N/A	N/A	N/A	N/A	N/A	N/A	N/A
**757**	Nuno	2020	Spain	Discriminative AI	Predicting complications	Prediction of risk of nosocomial infection	3&4	None	Other type of dataset	N/A	No information	N/A	N/A	Low	Unclear	Unclear	High	High
**758**	Nuno	2021	Spain	Discriminative AI	Predicting mortality	Prediction of all-cause mortality at an ICU	3&4	None	MIMIC III	N/A	1.000-10.000	N/A	N/A	Low	Low	Low	Unclear	Unclear
**759**	Nuryani	2023	Indonesia	Discriminative AI	Predicting complications	Detecting hypertension in intensive care unit patients	3&4	None	MIMIC II	N/A	10.000-100.000	N/A	N/A	Low	Unclear	Unclear	High	High
**760**	Oliver	2023	France	Discriminative AI	Assessing videos and images	Assessing endotracheal tube placement on intensive care unit chest radiographs	5	CONSORT/ CONSORT-AI-AI	Internal dataset	Other type of dataset	1.000-10.000	100-1.000	N/A	Unclear	Low	High	High	High
**761**	Orangi-Fard	2022	United States	Discriminative AI	Predicting readmissions	Predicting ICU 30-day readmission risk using natural language processing and machine learning	3&4	None	MIMIC III	N/A	1.000-10.000	N/A	N/A	N/A	N/A	N/A	N/A	N/A
**762**	Ortega-Martorell	2023	United Kingdom	Discriminative AI	Predicting complications	Predicting atrial fibrillation in sepsis patients at the intensive care unit	3&4	None	AmsterdamUMCdb	N/A	1.000-10.000	N/A	N/A	Low	Unclear	Low	Unclear	Unclear
**763**	Osathitporn	2023	Thailand	Discriminative AI	Determining physiological values	Estimating respiratory rate in intensive care unit patients	5	None	No information	Other type of dataset	No information	No information	N/A	Unclear	Low	Low	Unclear	Unclear
**764**	Otaguro	2021	Japan	Discriminative AI	Improving mechanical ventilation	Predicting successful extubation of mechanical ventilated patients at the intensive care unit	3&4	None	Internal dataset	N/A	100-1000	N/A	N/A	Low	Unclear	Low	High	High
**765**	Othman	2023	France	Discriminative AI	Predicting medication administration	Limiting the risk of prescription errors or drug iatrogeny	3&4	None	MIMIC III	N/A	10.000-100.000	N/A	N/A	Low	Low	Low	High	High
**766**	Ouyang	2023	China	Discriminative AI	Predicting mortality	Predicting mortality in patients in the intensive care unit with cerebral infarction	5	TRIPOD/ TRIPOD+AI	eICU-CRD	MIMIC III	1.000-10.000	1.000 - 10.000	N/A	Unclear	Low	Low	Low	Low
**767**	Ovcharenko	2023	Russia	Discriminative AI	Predicting mortality	Predicting COVID-19 mortality in patients at the intensive care unit	3&4	None	Other type of dataset	N/A	100-1000	N/A	N/A	Unclear	Low	Low	High	High
**768**	Padrão	2022	United States	Discriminative AI	Classifying sub-populations	Distinguishing phenotypes of sickle cell disease patients admitted to the intensive care unit	3&4	None	Internal dataset	N/A	100-1000	N/A	N/A	N/A	N/A	N/A	N/A	N/A
**769**	Pai	2021	Taiwan	Discriminative AI	Predicting complications	Predicting bloodstream infections	3&4	None	Internal dataset	N/A	1.000-10.000	N/A	N/A	Unclear	Low	Low	High	High
**770**	Pai	2022	Taiwan	Discriminative AI	Improving mechanical ventilation	Predicting extubation in critically ill ventilated patients	3&4	None	Internal dataset	N/A	1.000-10.000	N/A	N/A	Unclear	Low	Low	Low	Unclear
**771**	Pais	2024	India	Discriminative AI	Treatment recommendation	Predicting medication prescription errors in the intensive care unit	3&4	None	MIMIC IV	N/A	No information	N/A	N/A	Low	Low	High	Low	High
**772**	Pal	2022	India	Generative AI (Large language model)	Predicting complications	Predicting the development of an abnormal shock index in intensive care unit patients	3&4	None	MIMIC III	N/A	100-1000	N/A	N/A	Unclear	Unclear	High	High	High
**773**	Palmowski	2024	Italy	Discriminative AI	Predicting mortality	Predicting 30-day mortality in sepsis patients in the intensive care unit	5	None	Other type of dataset	AmsterdamUMCdb	100-1000	1.000 - 10.000	N/A	Low	Low	Low	High	High
**774**	Pan	2020	China	Discriminative AI	Predicting mortality	Predicting mortality for intensive care unit patients with COVID-19	3&4	TRIPOD/ TRIPOD+AI	Internal dataset	N/A	100-1000	N/A	N/A	Unclear	Low	Low	High	High
**775**	Pan	2021	China	Discriminative AI	Improving mechanical ventilation	Detecting patient-ventilator asynchrony in mechanical ventilation	3&4	None	Internal dataset	N/A	100.000+	N/A	N/A	Low	Low	Low	Low	Low

© 2025 Berkhout WEM et al. *JAMA Network Open.*

**776**	Pan	2023	China	Discriminative AI	Improving mechanical ventilation	Predicting extubation failure among invasively ventilated patients at the intensive care unit	3&4	None	Internal dataset	N/A	100-1000	N/A	N/A	Low	Low	Low	Unclear	Unclear
**777**	Pan	2023	China	Discriminative AI	Predicting complications	Predicting in-hospital mortality in intensive care unit patients with sepsis	3&4	None	MIMIC IV	N/A	10.000-100.000	N/A	N/A	Low	Low	High	High	High
**778**	Pan	2023	China	Discriminative AI	Predicting complications	Predicting acute kidney injury in the intensive care unit	3&4	None	MIMIC III	N/A	1.000-10.000	N/A	N/A	Unclear	Low	Low	Unclear	Unclear
**779**	Pan	2024	United States	Discriminative AI	Predicting complications	Predicting the onset risk of sepsis and acute kidney injury	3&4	None	eICU-CRD	N/A	10.000-100.000	N/A	N/A	Low	Low	Low	High	High
**780**	Pan	2024	China	Discriminative AI	Predicting mortality	Predicting mortality in intensive care unit patients	5	None	MIMIC III	Internal dataset	10.000-100.000	100-1.000	N/A	Unclear	Low	High	High	High
**781**	Pang	2022	China	Discriminative AI	Predicting mortality	Predicting in-hospital mortality in intensive care unit patients	3&4	None	MIMIC IV	N/A	10.000-100.000	N/A	N/A	Low	Unclear	Low	Unclear	Unclear
**782**	Pankaj	2023	India	Discriminative AI	Determining physiological values	Classifying arterial blood pressure in intensive care unit patients	3&4	None	MIMIC II, MIMIC III	N/A	10.000-100.000	N/A	N/A	Unclear	Low	Low	Unclear	Unclear
**783**	Paragliola	2023	Italy	Discriminative AI	Predicting deterioration	Identifying high-risk hypertensive patients	3&4	None	Internal dataset	N/A	10.000-100.000	N/A	N/A	Unclear	Low	Unclear	Unclear	Unclear
**784**	Parente	2021	Germany	Discriminative AI	Predicting complications	Predicting sepsis severity in intensive care unit patients	3&4	None	Internal dataset	N/A	1.000-10.000	N/A	N/A	Unclear	Unclear	Unclear	Unclear	Unclear
**785**	Park	2021	Korea	Discriminative AI	Improving mechanical ventilation	Predicting discontinuation of mechanical ventilation	3&4	None	Internal dataset	N/A	0-100	N/A	N/A	Low	Low	High	Unclear	High
**786**	Park	2022	Korea	Discriminative AI	Predicting clinical score	Predicting Medical Research Council (MRC) scale for neurological conditions and muscle strength	3&4	None	Internal dataset	N/A	100-1000	N/A	N/A	Low	Low	Low	High	High
**787**	Park	2022	Korea	Discriminative AI	Predicting mortality	Predicting mortality after burn surgery in critically ill burn patients admitted to the intensive care unit	3&4	None	Internal dataset	N/A	100-1000	N/A	N/A	Low	Unclear	Low	Unclear	Unclear
**788**	Park	2023	Korea	Discriminative AI	Improving mechanical ventilation	Predicting weaning outcomes	3&4	TRIPOD/ TRIPOD+AI	Internal dataset	N/A	100-1000	N/A	N/A	Low	Low	Low	Unclear	Unclear
**789**	Park	2023	Korea	Discriminative AI	Improving mechanical ventilation	Predicting weaning success in intensive care unit patients	3&4	TRIPOD/ TRIPOD+AI	Internal dataset	N/A	100-1000	N/A	N/A	Low	Low	Low	High	High
**790**	Parker	2021	United States	Discriminative AI	Assessing videos and images	Defining laryngeal lesions found in patients post-extubation at the intensive care unit	3&4	None	Internal dataset	N/A	100-1000	N/A	N/A	Low	Low	Low	High	High
**791**	Patel	2021	United Kingdom	Discriminative AI	Predicting mortality	Predicting mortality at the intensive care unit for COVID-19 patients	3&4	STROBE	Internal dataset	N/A	100-1000	N/A	N/A	Low	Unclear	Low	High	High
**792**	Pattalung	2021	Thailand	Discriminative AI	Predicting mortality	Predicting mortality in intensive care unit patients	3&4	None	eICU-CRD	N/A	10.000-100.000	N/A	N/A	Low	Low	Low	High	High
**793**	Pattharanitima	2021	United States	Discriminative AI	Predicting health improvement	Predicting renal replacement therapy-free survival in critically ill patients with acute kidney injury	3&4	TRIPOD/ TRIPOD+AI	MIMIC III	N/A	100-1000	N/A	N/A	Low	Low	Low	High	High
**794**	Pattharanitima	2021	Thailand	Discriminative AI	Classifying sub-populations	Classifying patients with lactic acidosis in the intensive care unit	3&4	None	MIMIC III	N/A	1.000-10.000	N/A	N/A	N/A	N/A	N/A	N/A	N/A
**795**	Pattharanitima	2021	Thailand	Discriminative AI	Predicting mortality	Predicting mortality in lactic acidosis patients admitted to the intensive care unit	3&4	TRIPOD/ TRIPOD+AI	MIMIC III	N/A	1.000-10.000	N/A	N/A	Low	Low	Low	High	High
**796**	Paul	2021	Singapore	Discriminative AI	Predicting mortality	Predicting intensive care unit in-hospital mortality	3&4	None	MIMIC III	N/A	10.000-100.000	N/A	N/A	Low	Low	Low	High	High
**797**	Pease	2022	United States	Discriminative AI	Predicting mortality; Predicting prognosis	Predicting mortality and unfavorable outcomes in patients with severe traumatic brain injury	5	TRIPOD/ TRIPOD+AI; STARD/ STARD-AI	Internal dataset	Other type of dataset	100-1000	100-1.000	N/A	Low	Unclear	Unclear	High	High

© 2025 Berkhout WEM et al. *JAMA Network Open.*

**798**	Peine	2021	Germany	Discriminative AI (Reinforcement learning)	Treatment recommendation	Suggesting mechanical ventilation regime for critically-ill patients	5	None	MIMIC III	eICU-CRD	10.000-100.000	10.000 - 100.000	N/A	N/A	N/A	N/A	N/A	N/A
**799**	Penarrubia	2023	France	Discriminative AI	Assessing videos and images	Determining alveolar recruitment on CT for patients with severe acute respiratory distress syndrome at the intensive care unit	5	None	N/A	Internal dataset	N/A	0-100	N/A	Low	Low	High	High	High
**800**	Peng	2018	United Kingdom	Discriminative AI (Reinforcement learning)	Treatment recommendation	Suggesting optimal treatment for patients with sepsis at the intensive care unit	3&4	None	MIMIC III	N/A	10.000-100.000	N/A	N/A	Low	Unclear	High	Low	High
**801**	Peng	2022	China	Discriminative AI	Predicting complications	Predicting acute kidney injury in patients with congestive heart failure	5	TRIPOD/ TRIPOD+AI	MIMIC III	eICU-CRD	1.000-10.000	1.000 - 10.000	N/A	Low	Low	Low	High	High
**802**	Peng	2022	China	Discriminative AI	Predicting mortality	Predicting 28-day in-hospital mortality in critically ill patients with heart failure combined with hypertension	5	None	MIMIC IV	eICU-CRD	10.000-100.000	No information	N/A	Low	Low	Low	Low	Low
**803**	Peng	2022	China	Discriminative AI	Predicting mortality	Predicting 30-day mortality in patients with sepsis-associated encephalopathy in the intensive care unit	3&4	TRIPOD/ TRIPOD+AI	MIMIC IV	N/A	1.000-10.000	N/A	N/A	Low	Unclear	Low	Low	Unclear
**804**	Peng	2023	China	Discriminative AI	Predicting complications	Predicting severe acute kidney injury (stage 3 or greater) in patients with TBI	5	None	MIMIC IV	eICU-CRD	100-1000	100-1.000	N/A	N/A	N/A	N/A	N/A	N/A
**805**	Pennati	2023	Italy	Discriminative AI	Assessing videos and images	Classifying lung recruitment respondence in acute respiratory distress syndrome patients admitted to the intensive care unit	3&4	None	Internal dataset	N/A	100-1000	N/A	N/A	Unclear	Unclear	Low	Unclear	Unclear
**806**	Penny-Dimri	2023	Australia	Discriminative AI	Predicting mortality; Predicting complications	Predicting adverse postoperative outcomes in cardiac surgery for patients admitted to the intensive care unit	5	None	Other type of dataset	MIMIC III	100.000+	1.000 - 10.000	N/A	Unclear	Unclear	Low	High	High
**807**	Peres	2022	Brazil	Discriminative AI	Predicting length of stay	Predicting intensive care unit length of stay	5	None	Internal dataset	Internal dataset	10.000-100.000	100.000+	N/A	Low	Low	Low	Unclear	Unclear
**808**	Peres	2023	Brazil	Discriminative AI	Predicting need for resource	Providing a resource use indicator for patients admitted to the intensive care unit	3&4	None	Internal dataset	N/A	10.000-100.000	N/A	N/A	Low	Low	Low	Unclear	Unclear
**809**	Peronnet	2024	France	Discriminative AI	Predicting deterioration	Predicting worsening of patients with sepsis at the intensive care unit	3&4	None	Other type of dataset	N/A	100-1000	N/A	N/A	Low	Low	Low	Unclear	Unclear
**810**	Persson	2021	Sweden	Discriminative AI	Predicting complications	Predicting sepsis in intensive care unit patients	3&4	None	MIMIC III	N/A	10.000-100.000	N/A	N/A	N/A	N/A	N/A	N/A	N/A
**811**	Persson	2023	Sweden	Discriminative AI	Predicting complications	Predicting the onset of acute kidney injury in intensive care unit patients	3&4	None	MIMIC IV	N/A	10.000-100.000	N/A	N/A	Low	Low	High	High	High
**812**	Persson	2024	Sweden	Discriminative AI	Predicting complications	Predicting sepsis in intensive care unit patients	8	None	N/A	N/A	N/A	N/A	304	Low	Low	High	Low	High
**813**	Pessoa	2022	Brazil	Discriminative AI	Predicting complications	Predicting septic and hypovolemic shock in intensive care unit patients	3&4	None	Internal dataset	N/A	100-1000	N/A	N/A	Low	Low	Low	High	High
**814**	Petrov	2023	United States	Discriminative AI	Predicting complications	Predicting intracranial pressure crises in patients with severe traumatic brain injury	3&4	None	Internal dataset	N/A	1.000-10.000	N/A	N/A	Unclear	Low	High	High	High
**815**	Pettinati	2020	United States	Discriminative AI	Predicting complications	Predicting sepsis in intensive care unit patients	3&4	None	MIMIC III	N/A	1.000-10.000	N/A	N/A	Unclear	Low	Unclear	High	High
**816**	Pham	2022	Netherlands	Discriminative AI	Predicting prognosis	Predicting outcome of postanoxic coma in patients admitted to the intensive care unit	5	None	Internal dataset	Internal dataset	100-1000	100-1.000	N/A	Low	Low	Low	Unclear	Unclear

© 2025 Berkhout WEM et al. *JAMA Network Open.*

**817**	Phetrittikun	2023	Thailand	Discriminative AI	Predicting complications	Predicting acid-base and potassium imbalances	3&4	None	Internal dataset	N/A	1.000-10.000	N/A	N/A	Unclear	Unclear	Unclear	High	High
**818**	Piedrafita	2022	France	Discriminative AI	Predicting complications	Predicting acute kidney injury	5	None	Internal dataset	Other type of dataset	100-1000	1.000 - 10.000	N/A	Unclear	Low	Low	High	High
**819**	Pinevich	2022	United States	Discriminative AI	Predicting complications	Detecting shock in the intensive care unit	6	None	N/A	N/A	N/A	N/A	N/A	N/A	N/A	N/A	N/A	N/A
**820**	Pinto	2021	Switzerland	Discriminative AI	Predicting complications	Predicting cardiac injury and dysfunction in patients with septic shock	3&4	STROBE	Other type of dataset	N/A	0-100	N/A	N/A	Low	Unclear	Low	High	High
**821**	Pirracchio	2016	United States	Discriminative AI	Predicting mortality	Predicting intensive care unit mortality	3&4	None	MIMIC II	N/A	10.000-100.000	N/A	N/A	Low	Unclear	Low	High	High
**822**	Pirracchio	2020	United States	Discriminative AI	Treatment recommendation	Estimate the individual treatment effect of corticosteroids for patients with septic shock	5	TRIPOD/ TRIPOD+AI	Other type of dataset	Other type of dataset	1.000-10.000	0-100	N/A	Low	Unclear	Low	High	High
**823**	Pishgar	2022	United States	Discriminative AI	Predicting readmissions	Predicting 30-day readmission for intensive care unit patients with heart failure	3&4	TRIPOD/ TRIPOD+AI	MIMIC III	N/A	1.000-10.000	N/A	N/A	Low	Unclear	Low	Unclear	Unclear
**824**	Ponce	2020	Brazil	Discriminative AI	Determining physiological values	Predicting resting energy expenditure for acute kidney injury patients on dialysis and mechanical ventilation	3&4	None	Internal dataset	N/A	100-1000	N/A	N/A	Low	Low	Unclear	Unclear	Unclear
**825**	Ponthongmak	2023	Thailand	Generative AI (Large language model)	Assessing clinical notes	Providing automated international classification of diseases coding from discharge summary texts	5	TRIPOD/ TRIPOD+AI	Internal dataset	MIMIC III	10.000-100.000	10.000 - 100.000	N/A	Low	Low	Low	High	High
**826**	Popkes	2019	United Kingdom	Discriminative AI	Predicting mortality	Predicting mortality in intensive care unit patients	3&4	None	MIMIC III	N/A	10.000-100.000	N/A	N/A	Unclear	Low	Low	Low	Unclear
**827**	Potter	2024	United States	Discriminative AI	Classifying sub-populations	Identifying delirium subtypes in patients admitted to the intensive care unit	3&4	None	Other type of dataset	N/A	100-1000	N/A	N/A	N/A	N/A	N/A	N/A	N/A
**828**	Poulsen	2022	United States	Discriminative AI	Predicting complications	Classification of opioid use disorder	3&4	None	MIMIC III	N/A	100-1000	N/A	N/A	N/A	N/A	N/A	N/A	N/A
**829**	Prasad	2022	United States	Discriminative AI (Reinforcement learning)	Treatment recommendation	Providing recommendation for the route and dose of an electrolyte for intensive care unit patients	3&4	None	Internal dataset	N/A	10.000-100.000	N/A	N/A	N/A	N/A	N/A	N/A	N/A
**830**	Prasad	2017	United States	Discriminative AI (Reinforcement learning)	Treatment recommendation	A decision support tool that predicts time-to-extubation readiness and to recommend a personalized regime of sedation dosage and ventilator support	3&4	None	MIMIC II	N/A	1.000-10.000	N/A	N/A	N/A	N/A	N/A	N/A	N/A
**831**	Qi	2022	China	Discriminative AI	Predicting mortality	Predicting in-hospital mortality in septic patients with diabetes admitted to the intensive care unit	5	RECORD	MIMIC IV	Other type of dataset, eICU-CRD	1.000-10.000	100-1.000	N/A	Low	Low	Low	High	High
**832**	Qian	2021	China	Discriminative AI	Predicting complications	Predicting acute kidney injury in the intensive care unit	3&4	None	MIMIC III	N/A	10.000-100.000	N/A	N/A	Low	Low	High	Low	High
**833**	Qiu	2021	China	Discriminative AI	Predicting readmissions	Predicting readmission of intensive care unit patients	3&4	None	Internal dataset	N/A	1.000-10.000	N/A	N/A	Unclear	Low	Low	Low	Unclear
**834**	Qiu	2022	China	Discriminative AI (Reinforcement learning)	Provide dosage recommendations	Provide heparin dosing	3&4	None	MIMIC III	N/A	100-1000	N/A	N/A	N/A	N/A	N/A	N/A	N/A
**835**	Qiu	2023	China	Discriminative AI	Improving mechanical ventilation	Prediction of patient-specific ventilator parameters	3&4	None	MIMIC III	N/A	10.000-100.000	N/A	N/A	Unclear	Unclear	Low	High	High
**836**	Quesado	2022	Portugal	Discriminative AI	Diagnostic	Predicting COVID-19 diagnosis in intensive care unit patients	3&4	None	Internal dataset	N/A	No information	N/A	N/A	Unclear	Unclear	Low	Unclear	Unclear

© 2025 Berkhout WEM et al. *JAMA Network Open.*

**837**	Quoc	2023	Vietnam	Discriminative AI	Predicting complications	Predicting antibiotic resistance in intensive care unit patients	3&4	None	Internal dataset	N/A	1.000-10.000	N/A	N/A	Low	Unclear	Low	Unclear	Unclear
**838**	Radhakrishnan	2022	India	Discriminative AI	Improving mechanical ventilation	Predicting inspired oxygen, mechanical ventilator modes and PEEP in patients at the intensive care unit	3&4	None	Internal dataset	N/A	No information	N/A	N/A	Low	Low	Low	High	High
**839**	Rafiei	2024	United States	Discriminative AI	Predicting complications	Predicting fluid overload in the intensive care unit	3&4	STROBE	Internal dataset	N/A	No information	N/A	N/A	Low	High	Low	High	High
**840**	Raghu	2017	United Kingdom	Discriminative AI (Reinforcement Learning)	Treatment recommendation	Deduce treatment policies for septic patients	3&4	None	MIMIC III	N/A	10.000-100.000	N/A	N/A	N/A	N/A	N/A	N/A	N/A
**841**	Raghu	2018	United Kingdom	Discriminative AI (Reinforcement Learning)	Treatment recommendation	Discover treatment policies for sepsis patients	3&4	None	MIMIC III	N/A	10.000-100.000	N/A	N/A	N/A	N/A	N/A	N/A	N/A
**842**	Rahman	2021	United States	Discriminative AI	Predicting need for resource	Predicting hemodynamic interventions in intensive care unit patients	5	TRIPOD/ TRIPOD+AI	eICU-CRD	MIMIC III	100.000+	10.000 - 100.000	N/A	Low	Unclear	Low	Low	Unclear
**843**	Raj	2022	Finland	Discriminative AI	Predicting mortality	Predicting mortality after traumatic brain injury in intensive care unit patients	5	None	Internal dataset	eICU-CRD, internal dataset	1.000-10.000	100-1.000	N/A	Low	Low	Low	High	High
**844**	Rajendran	2023	United States	Discriminative AI	Predicting complications	Predicting sepsis and acute kidney injury in the intensive care unit	3&4	None	eICU-CRD	N/A	10.000-100.000	N/A	N/A	Unclear	Unclear	Unclear	Unclear	Unclear
**845**	Rajendran	2024	United States	Discriminative AI	Classifying sub-populations	Defining subphenotypes in sepsis patients at the intensive care unit	5	None	eICU-CRD, MIMIC IV	Other type of dataset	10.000-100.000	1.000 - 10.000	N/A	N/A	N/A	N/A	N/A	N/A
**846**	Ramakrishnaiah	2024	Australia	Discriminative AI	Predicting mortality; Predicting length of stay	Predicting intensive care unit mortality and length of stay for patients with sepsis	3&4	None	MIMIC IV, eICU-CRD	N/A	No information	N/A	N/A	Unclear	Unclear	Low	Unclear	Unclear
**847**	Ramos	2021	Portugal	Discriminative AI	Predicting complications	Predicting the onset of septic shock in intensive care unit patients	3&4	None	MIMIC III	N/A	10.000-100.000	N/A	N/A	Low	Unclear	Unclear	High	High
**848**	Rangan	2022	United States	Discriminative AI	Predicting complications	Predicting sepsis in intensive care unit patients	5	None	Other type of dataset	Other type of dataset	1.000-10.000	1.000 - 10.000	N/A	Low	Low	Low	High	High
**849**	Rank	2020	Germany	Discriminative AI	Predicting complications	Predicting acute kidney injury for patients admitted to the intensive care unit after cardiothoracic surgery	3&4	TRIPOD/ TRIPOD+AI	Internal dataset	N/A	1.000-10.000	N/A	N/A	Low	Low	Low	Unclear	Unclear
**850**	Rannon	2023	Israel	Discriminative AI	Predicting an event	Predicting resistance to antibiotic treatment for patients admitted to the intensive care unit	3&4	None	MIMIC III	N/A	100-1000	N/A	N/A	Low	Low	Low	High	High
**851**	Raphaeli	2023	Israel	Discriminative AI	Predicting complications	Predicting early enteral nutrition failure in the intensive care unit	3&4	TRIPOD/ TRIPOD+AI	Internal dataset	N/A	1.000-10.000	N/A	N/A	Low	Low	Unclear	High	High
**852**	Rastegar	2021	New Zealand	Discriminative AI	Determining physiological values	Estimating systolic blood pressure and diastolic blood pressure in intensive care unit patients	3&4	None	MIMIC III	N/A	10.000-100.000	N/A	N/A	Unclear	Low	Low	Unclear	Unclear
**853**	Rauseo	2022	Italy	Discriminative AI	Predicting mortality	Predicting in-hospital mortality in intensive care unit patients with SARS-CoV-2 infection	3&4	None	Internal dataset	N/A	100-1000	N/A	N/A	Low	Unclear	Low	High	High
**854**	Rayan	2021	Egypt	Discriminative AI	Predicting complications	Predicting sepsis in the intensive care unit	5	None	Other type of dataset	Other type of dataset	10.000-100.000	10.000 - 100.000	N/A	Unclear	Unclear	Unclear	High	High
**855**	Reamaroon	2021	United States	Discriminative AI	Assessing videos and images	Detecting acute respiratory distress syndrome	3&4	None	Internal dataset	N/A	100-1000	N/A	N/A	Unclear	Low	High	High	High
**856**	Rehm	2020	United States	Discriminative AI	Predicting complications; Improving mechanical ventilation	Predicting patient-ventilator asynchrony and detecting acute respiratory distress syndrome in the intensive care unit	3&4	None	Internal dataset	N/A	1.000-10.000	N/A	N/A	Low	Low	Low	High	High

© 2025 Berkhout WEM et al. *JAMA Network Open.*

**857**	Rehm	2021	United States	Discriminative AI	Diagnosing	Classifying between patients with and without acute respiratory distress syndrome for patients admitted tot he intensive care unit	3&4	None	Internal dataset	N/A	100-1000	N/A	N/A	Unclear	Low	High	High	High
**858**	Ren	2022	United States	Discriminative AI	Predicting complications; Determining physiological values	Predicting PaO2 from SpO2 values and moderate to severe hypoxemic respiratory failure for patients admitted to the intensive care unit	5	None	MIMIC III	Internal dataset	10.000-100.000	100-1.000	N/A	Low	Low	Low	High	High
**859**	Ren	2022	China	Discriminative AI	Predicting mortality	Predicting intensive care unit mortality	3&4	None	Other type of dataset	N/A	100.000+	N/A	N/A	Unclear	Unclear	Low	Low	Unclear
**860**	Ren	2022	China	Discriminative AI	Improving mechanical ventilation	Classifying sputum deposition for mechanically ventilated patients	3&4	None	Internal dataset	N/A	100-1000	N/A	N/A	Unclear	Low	Low	High	High
**861**	Ren	2023	China	Discriminative AI	Predicting complications	Classifying sputum deposition categories	3&4	None	Internal dataset	N/A	10.000-100.000	N/A	N/A	Unclear	Low	Low	High	High
**862**	Ren	2023	China	Discriminative AI	Predicting mortality	Predicting in-hospital mortality of intensive care unit patients with acute pancreatitis	5	None	MIMIC IV	eICU-CRD	100-1000	100-1.000	N/A	Unclear	Low	Unclear	Unclear	Unclear
**863**	Rezaei	2023	Canada	Discriminative AI	Predicting mortality	Predicting mortality in intensive care unit patients	3&4	None	MIMIC IV	N/A	10.000-100.000	N/A	N/A	Unclear	Unclear	Unclear	Unclear	Unclear
**864**	Rhodes	2023	United States	Discriminative AI	Predicting prognosis	Predicting restricted mean residual life of septic patients in the intensive care unit	3&4	None	MIMIC III	N/A	10.000-100.000	N/A	N/A	N/A	N/A	N/A	N/A	N/A
**865**	Rhodes	2024	United States	Discriminative AI (Reinforcement learning)	Treatment recommendation	Providing optimal treatment regime for septic patients in the intensive care unit	3&4	None	MIMIC III	N/A	10.000-100.000	N/A	N/A	Unclear	Unclear	Unclear	Unclear	Unclear
**866**	Rigo-Bonnin	2022	Spain	Discriminative AI	Predicting mortality	Predicting mortality in patients with severe acute respiratory syndrome coronavirus 2 admitted to the intensive care unit	5	None	Internal dataset	Internal dataset	100-1000	0-100	N/A	Low	Unclear	Low	High	High
**867**	Rodríguez	2021	Spain	Discriminative AI	Classifying sub-populations	Deriving clinical COVID-19 phenotypes at intensive care unit admission	3&4	None	Internal dataset	N/A	1.000-10.000	N/A	N/A	N/A	N/A	N/A	N/A	N/A
**868**	Rodríguez	2024	Spain	Discriminative AI	Classifying sub-populations	Classifying phenotypes and subgroups in patients with COVID-19 in the intensive care	5	STROBE	N/A	Other type of dataset	N/A	1.000 - 10.000	N/A	N/A	N/A	N/A	N/A	N/A
**869**	Rodriguez-Obregon	2023	Mexico	Discriminative AI	Assessing videos and images	Providing volumetric estimation of COVID-19 lesions in patients admitted to the intensive care unit	3&4	None	Internal dataset	N/A	100-1000	N/A	N/A	N/A	N/A	N/A	N/A	N/A
**870**	Roggeveen	2021	Netherlands	Discriminative AI (Reinforcement Learning)	Treatment recommendation	Optimizing haemodynamic treatment of patients with sepss	5	None	MIMIC III	AmsterdamUMCdb	1.000-10.000	1.000 - 10.000	N/A	N/A	N/A	N/A	N/A	N/A
**871**	Roggeveen	2024	Netherlands	Discriminative AI (Reinforcement learning)	Treatment recommendation	Optimising mechanical ventilator settings in critically ill patients with severe COVID-19	3&4	None	Other type of dataset	N/A	1.000-10.000	N/A	N/A	N/A	N/A	N/A	N/A	N/A
**872**	Romanov	2024	Israel	Discriminative AI	Predicting an event	Predicting the next therapeutic actions for intensive care unit patients with hypokalemia and hypoglycemia	3&4	None	MIMIC IV	N/A	No information	N/A	N/A	N/A	N/A	N/A	N/A	N/A
**873**	Romanowski	2022	United States	Generative AI (Large language model)	Assessing clinical notes	Extracting social determinants of health from clinical text	3&4	None	MIMIC III, Other type of dataset, Internal dataset	N/A	No information	No information	N/A	N/A	N/A	N/A	N/A	N/A
**874**	Rong	2022	China	Discriminative AI	Predicting mortality	Predicting 30-day mortality of elderly patients with cardiogenic shock	5	None	MIMIC III	Internal dataset	100-1000	100-1.000	N/A	Low	Low	Low	High	High
**875**	Roquencourt	2023	France	Discriminative AI	Diagnostic	Predicting diagnosis of COVID-19	3&4	None	Internal dataset	N/A	0-100	N/A	N/A	Unclear	Unclear	Low	Unclear	Unclear

© 2025 Berkhout WEM et al. *JAMA Network Open.*

**876**	Rosenfeld	2022	Israel	Discriminative AI	Predicting complications	Predicting hypotensive episode in trauma patients admitted to the intensive care unit	3&4	None	MIMIC III	N/A	10.000-100.000	N/A	N/A	Low	Unclear	High	High	High
**877**	Roy	2021	United Kingdom	Discriminative AI	Predicting mortality; Predicting complications; Predicting length of stay	Predicting acute kidney injury, continuous renal replacement therapy, mechanical ventilation, vasoactive medications, mortality, and length of stay for patients admitted to the intensive care unit	3&4	None	MIMIC III	N/A	10.000-100.000	N/A	N/A	Low	Unclear	High	High	High
**878**	Ruan	2023	China	Discriminative AI	Predicting mortality	Predicting mortality in mechanically ventilated patients admitted to the intensive care unit	3&4	None	Internal dataset	N/A	1.000-10.000	N/A	N/A	Low	Unclear	Low	Unclear	Unclear
**879**	Rubinos	2022	United States	Discriminative AI	Predicting complications	Predicting ventriculopertoneal shunt dependency after subarachnoid hemorrhage	3&4	STROBE	Other type of dataset	N/A	0-100	N/A	N/A	Unclear	Low	Low	Unclear	Unclear
**880**	Ruyssinck	2016	Belgium	Discriminative AI	Predicting length of stay	Predicting length of stay in the intensive care unit	3&4	None	Internal dataset	N/A	10.000-100.000	N/A	N/A	Low	Low	High	Unclear	High
**881**	Ryan	2020	United States	Discriminative AI	Predicting mortality	Predicting mortality in COVID-19, pneumonia, and mechanically ventilated patients	5	None	MIMIC	Internal dataset	10.000-100.000	100-1.000	N/A	Unclear	Unclear	High	Unclear	High
**882**	Ryan	2022	United States	Discriminative AI	Predicting complications	Predicting acute kidney injury after cardiac surgery in the intensive care unit	3&4	None	MIMIC IV	N/A	1.000-10.000	N/A	N/A	Low	Low	Low	Unclear	Unclear
**883**	Sabeti	2021	United States	Discriminative AI	Predicting complications	Detecting acute respiratory distress syndrome	3&4	None	Internal dataset	N/A	100-1000	N/A	N/A	Unclear	Low	Unclear	Unclear	Unclear
**884**	Safaei	2022	United States	Discriminative AI	Predicting mortality	Predicting intensive care unit mortality in different disease groups	3&4	None	eICU-CRD	N/A	No information	N/A	N/A	Unclear	Low	Low	Unclear	Unclear
**885**	Sakagianni	2023	Greece	Discriminative AI	Predicting mortality	Predicting intensive care unit mortality	3&4	None	Internal dataset	N/A	100-1000	N/A	N/A	Unclear	Unclear	Low	High	High
**886**	Sakagianni	2023	Greece	Discriminative AI	Predicting mortality	Predicting COVID-19 mortality in the intensive care unit	3&4	None	Internal dataset	N/A	100-1000	N/A	N/A	Unclear	Unclear	Low	High	High
**887**	Sakhaee	2022	Iran	Discriminative AI	Predicting mortality	Predicting mortality in intensive care unit patients with opioid overdose	3&4	None	Internal dataset	N/A	0-100	N/A	N/A	Unclear	Unclear	Low	High	High
**888**	Saleh	2021	Egypt	Discriminative AI	Determining physiological values	Predicting systolic blood pressure for intensive care unit patients	3&4	None	MIMIC II	N/A	No information	N/A	N/A	Unclear	Low	High	Unclear	High
**889**	Salimi	2023	Iran	Discriminative AI	Predicting readmissions	Predicting readmission of cardiovascular patients admitted to the coronary care unit	5	STROBE	Internal dataset	Internal dataset	100-1000	0-100	N/A	Unclear	Unclear	Low	High	High
**890**	Salman	2020	United States	Discriminative AI	Predicting mortality	Predicting mortality in intensive care unit patients with myocardial infarction	3&4	None	MIMIC III	N/A	1.000-10.000	N/A	N/A	Low	Low	Low	Unclear	Unclear
**891**	Samadani	2023	United States	Discriminative AI	Predicting complications	Predicting ventilator-associated pneumonia in intensive care unit patients	3&4	None	eICU-CRD	N/A	10.000-100.000	N/A	N/A	Unclear	Low	Low	Low	Unclear
**892**	Samadi	2022	Germany	Discriminative AI	Predicting mortality	Predict vital status of COVID-19 patients requiring mechanical ventilation at the ICU	3&4	None	Internal dataset	N/A	0-100	N/A	N/A	Low	Low	Unclear	High	High
**893**	Samadi	2024	Germany	Discriminative AI	Predicting mortality	Predicting intensive care unit mortality	5	TRIPOD/ TRIPOD+AI	Internal dataset	Internal dataset	1.000-10.000	100-1.000; 1.000 - 10.000	N/A	Low	Low	Low	High	High
**894**	Samy	2023	India	Discriminative AI	Predicting length of stay	Predicting length of stay	3&4	None	MIMIC III	N/A	10.000-100.000	N/A	N/A	Unclear	Unclear	Unclear	Unclear	Unclear
**895**	Saner	2024	Germany	Discriminative AI	Predicting clinical score	Determining the SOFA score	3&4	None	Other type of dataset	N/A	0-100	N/A	N/A	N/A	N/A	N/A	N/A	N/A

© 2025 Berkhout WEM et al. *JAMA Network Open.*

**896**	Sanfilippo	2023	Italy	Discriminative AI	Assessing videos and images	Determining inferior vena cava distensibility	8	None	N/A	N/A	N/A	N/A	33	N/A	N/A	N/A	N/A	N/A
**897**	Sarkar	2023	China	Discriminative AI	Determining physiological values	Predicting blood pressure in intensive care unit patients	5	None	MIMIC II	MIMIC-III	No information	10.000 - 100.000	N/A	Unclear	Low	Low	Unclear	Unclear
**898**	Sarraf	2024	United States	Discriminative AI	Predicting mortality	Predicting mortality of sepsis patients admitted to the intensive care unit	5	TRIPOD/ TRIPOD+AI	MIMIC IV	Internal dataset	10.000- 100.000	0-100	N/A	Unclear	Unclear	Low	High	High
**899**	Sato	2021	Japan	Discriminative AI	Predicting complications	Predicting acute kidney injury in the intensive care unit	3&4	None	eICU-CRD	N/A	1.000- 10.000	N/A	N/A	Low	Low	High	High	High
**900**	Savage	2023	United States	Generative AI (Large language model)	Treatment recommendation	Identifying patients appropriate for a deep vein thrombosis prophylaxis	3&4	None	MIMIC III	N/A	100-1000	N/A	N/A	N/A	N/A	N/A	N/A	N/A
**901**	Savareh	2021	Iran	Discriminative AI	Predicting length of stay	Predicting length of stay in a neurosurgical intensive care unit	3&4	None	Internal dataset	N/A	1.000- 10.000	N/A	N/A	Low	Unclear	Low	High	High
**902**	Sayed	2021	Spain	Discriminative AI	Predicting prognosis	Predicting acute respiratory distress syndrome severity	3&4	None	MIMIC III	N/A	1.000- 10.000	N/A	N/A	Low	Low	Unclear	High	High
**903**	Sayed	2021	Spain	Discriminative AI	Improving mechanical ventilation	Predicting mechanical ventilation duration in acute respiratory distress syndrome in the intensive care unit	5	None	MIMIC III	eICU-CRD	1.000- 10.000	1.000 - 10.000	N/A	Low	Unclear	Low	Unclear	Unclear
**904**	Scales	2022	Canada	Discriminative AI	Predicting deterioration	Predicting time to death after withdrawal of life-sustaining measures	3&4	None	Other type of dataset	N/A	100-1000	N/A	N/A	High	Low	Low	Unclear	Unclear
**905**	Scheibner	2022	United States	Discriminative AI	Predicting complications	Predicting vasopressin responsiveness in patients with septic shock	5	TRIPOD/ TRIPOD+AI	Internal dataset	MIMIC III	100-1000	100-1.000	N/A	Low	Low	Low	Low	Low
**906**	Schmidt	2021	France	Discriminative AI	Predicting mortality	Predicting 90-day mortality of patients with COVID-19 at the intensive care unit	3&4	None	Internal dataset	N/A	1.000- 10.000	N/A	N/A	Low	Unclear	Low	Unclear	Unclear
**907**	Schmidt	2023	Germany	Discriminative AI	Predicting mortality	Predicting mortality in burned patients at the intensive care unit	3&4	None	Other type of dataset	N/A	1.000- 10.000	N/A	N/A	Low	Low	Low	High	High
**908**	Schulz	2023	Germany	Discriminative AI	Predicting complications; Classifying sub-populations	Predicting the severity of pulmonary edema	3&4	None	Internal dataset	N/A	100-1000	N/A	N/A	N/A	N/A	N/A	N/A	N/A
**909**	Schvetz	2021	Israel	Discriminative AI	Predicting complications	Predicting intensive care unit acquired sepsis	3&4	None	MIMIC III	N/A	1.000- 10.000	N/A	N/A	Low	Low	High	Unclear	High
**910**	Schwager	2021	United States	Discriminative AI	Predicting complications	Predicting acute kidney injury in intensive care unit patients	3&4	None	Internal dataset	N/A	10.000- 100.000	N/A	N/A	Low	Low	Low	Low	Low
**911**	Schwager	2023	United States	Discriminative AI	Predicting complications	To predict the time of diagnosis of AKI	3&4	None	Internal dataset	N/A	10.000- 100.000	N/A	N/A	Low	Low	Low	High	High
**912**	Schwager	2023	United States	Discriminative AI	Predicting prognosis	Predicting duration of ventilation in the intensive care unit	5	None	eICU-CRD	eICU-CRD	100.000+	10.000 - 100.000	N/A	Low	Low	Low	High	High
**913**	Schwager	2024	United States	Discriminative AI	Predicting need for resource	Predicting utilization of invasive and non-invasive ventilation during intensive care unit stay	5	None	eICU-CRD	Internal dataset	100.000+	10.000 - 100.000	N/A	Low	Low	Low	High	High
**914**	Schweingruber	2022	Germany	Discriminative AI	Predicting complications	Predicting intracranial hypertension	5	None	Internal dataset	MIMIC, eICU-CRD	1.000- 10.000	1.000 - 10.000	N/A	Unclear	Low	Unclear	High	High
**915**	Schweingruber	2024	Germany	Discriminative AI	Predicting complications	Predicting ventricular peritoneal shunt dependency in aneurysmal subarachnoid haemorrhage patients	3&4	RECORD	Internal dataset	N/A	100-1000	N/A	N/A	Unclear	Unclear	Unclear	Unclear	Unclear
**916**	Searle	2021	United Kingdom	Generative AI (Large language model)	Assessing clinical notes	Quantifying information redundancy in clinical text	5	None	MIMIC III	Internal dataset	No information	No information	N/A	N/A	N/A	N/A	N/A	N/A
**917**	Selcuk	2022	Turkey	Discriminative AI	Predicting mortality	Predicting sepsis mortality in the intensive care unit	3&4	None	Internal dataset	N/A	100-1000	N/A	N/A	Low	Low	Low	High	High
**918**	Selvaskandan	2023	United Kingdom	Discriminative AI	Treatment recommendation	Predict the need for acute renal	3&4	None	No information	N/A	10.000- 100.000	N/A	N/A	Unclear	Low	Low	High	High

© 2025 Berkhout WEM et al. *JAMA Network Open.*

						replacement therapy among patients with AKI											
**919**	Semeraro	2024	Italy	Generative AI (Large language model)	Treatment recommendation	Recommending treatment for temperature control after cardiac arrest	5	None	N/A	Other type of dataset	N/A	0-100	N/A	N/A	N/A	N/A	N/A	N/A
**920**	Sena	2023	United States	Discriminative AI	Predicting clinical score	Predicting diurnal pain classification of patients in the intensive care unit	3&4	None	Internal dataset	N/A	1.000-10.000	N/A	N/A	Low	Low	High	High	High
**921**	Sena	2024	Brazil	Discriminative AI	Predicting prognosis	Predicting acuity state in intensive care unit patients	3&4	None	Internal dataset	N/A	100-1000	N/A	N/A	Low	Low	Low	Unclear	Unclear
**922**	Serafim	2024	United States	Discriminative AI	Predicting mortality	Predicting in-hospital mortality in critically ill patients with cirrhosis and sepsis	3&4	None	Internal dataset	N/A	100-1000	N/A	N/A	Low	Unclear	Low	Unclear	Unclear
**923**	Serviá	2020	Spain	Discriminative AI	Predicting mortality	Predicting in-hospital mortality of critically traumatic patients in the intensive care unit	3&4	None	Other type of dataset	N/A	1.000-10.000	N/A	N/A	Low	Low	Low	Unclear	Unclear
**924**	Seymour	2022	United States	Discriminative AI	Predicting complications	Predicting hematoma expansion following intracranial hemorrhage at the intensive care unit	3&4	None	Internal dataset	N/A	100-1000	N/A	N/A	Unclear	Low	High	High	High
**925**	Shaikh	2022	United States	Discriminative AI	Assessing videos and images	Automating left ventricular outflow tract velocity time integral	7	None	N/A	N/A	N/A	N/A	N/A	N/A	N/A	N/A	N/A	N/A
**926**	Shankar	2023	Canada	Discriminative AI	Predicting an event	Predicting patient trajectory in intensive care unit patients	3&4	None	MIMIC IV	N/A	No information	N/A	N/A	Low	Low	High	High	High
**927**	Shapiro	2022	Israel	Discriminative AI	Treatment recommendation	Predicting treatment in hypokalemia, hypoglycemia and hypotension	3&4	None	MIMIC IV	N/A	No information	N/A	N/A	Unclear	Unclear	Unclear	Unclear	Unclear
**928**	Sharafoddini	2021	Canada	Discriminative AI	Classifying sub-populations	Identifying subpopulations of septic patients at the intensive care unit	3&4	None	MIMIC III	N/A	1.000-10.000	N/A	N/A	N/A	N/A	N/A	N/A	N/A
**929**	Sharma	2022	United Kingdom	Discriminative AI	Predicting mortality; Classifying sub-populations	Predicting in-hospital mortality, predicting decompensation and classifying phenotypes	3&4	None	MIMIC III	N/A	No information	N/A	N/A	Unclear	Low	Low	Unclear	Unclear
**930**	Sharma	2022	India	Discriminative AI	Predicting complications	Predicting sepsis for patients admitted to the intensive care unit	3&4	None	Internal dataset	N/A	No information	N/A	N/A	Unclear	Unclear	Unclear	Unclear	Unclear
**931**	Shashikumar	2021	United States	Discriminative AI	Predicting complications	Predicting sepsis in intensive care unit patients	5	None	Internal dataset	Internal dataset, MIMIC III	10.000-100.000	10.000 - 100.000	N/A	Low	Low	High	Unclear	High
**932**	Shashikumar	2021	United States	Discriminative AI	Predicting need for resource	Predicting the need for mechanical ventilation for intensive care unit patients (with COVID-19)	5	TRIPOD/ TRIPOD+AI	Internal dataset	Internal dataset	10.000-100.000	1.000 - 10.000	N/A	Low	Low	High	Unclear	High
**933**	Shawwa	2021	United States	Discriminative AI	Predicting complications	Predicting acute kidney injury in critically ill patients	5	None	Internal dataset	MIMIC III	10.000-100.000	10.000 - 100.000	N/A	Low	Unclear	Low	Unclear	Unclear
**934**	She	2023	China	Discriminative AI	Predicting complications	Predicting acute kidney injury in patients with intracerebral hemorrhage admitted to the intensive care unit	3&4	None	MIMIC III	N/A	1.000-10.000	N/A	N/A	Low	Unclear	Unclear	Unclear	Unclear
**935**	She	2024	China	Discriminative AI	Predicting mortality	Predicting mortality in patients with epilepsy admitted to the intensive care unit	5	None	MIMIC IV	eICU-CRD	100-1000	1.000 - 10.000	N/A	Low	Low	High	Low	High
**936**	Sheetrit	2023	Israel	Discriminative AI	Predicting readmissions	Predicting unplanned readmission in the intensive care unit	3&4	None	MIMIC III	N/A	10.000-100.000	N/A	N/A	Low	Unclear	Low	Unclear	Unclear
**937**	Sheikh	2024	United States	Generative AI (Large language model)	Support in patient education	Responding to patient education questions on acute kidney injury and continuous renal replacement therapy	5	None	N/A	Internal dataset	N/A	0-100	N/A	N/A	N/A	N/A	N/A	N/A
**938**	Sheikh	2024	United States	Generative AI (Large language model)	Alarm management	Providing support with continuous renal replacement therapy alarm management	5	None	N/A	Other type of dataset	N/A	0-100	N/A	N/A	N/A	N/A	N/A	N/A

© 2025 Berkhout WEM et al. *JAMA Network Open.*

**939**	Sheikhalishahi	2023	United Kingdom	Discriminative AI	Predicting complications	Predicting delirium in critical care	3&4	None	MIMIC III, eICU-CRD	N/A	10.000-100.000	N/A	N/A	Low	Low	Low	Unclear	Unclear
**940**	Sheikhalishahi	2024	Germany	Discriminative AI	Improving mechanical ventilation	Predicting successful weaning from mechanical ventilation in the intensive care unit	3&4	None	eICU-CRD, MIMIC IV	N/A	10.000-100.000	N/A	N/A	Low	Low	High	Unclear	High
**941**	Shi	2021	United States	Discriminative AI	Predicting mortality	Predicting mortality in intensive care unit patients	3&4	None	MIMIC III	N/A	10.000-100.000	N/A	N/A	Unclear	Unclear	Low	Unclear	Unclear
**942**	Shi	2021	United States	Discriminative AI	Predicting complications	Predicting acute kidney injury	5	None	Other type of dataset	Other type of dataset	100-1000	100-1.000	N/A	Low	Low	High	High	High
**943**	Shi	2021	China	Discriminative AI	Predicting mortality	Predicting mortality at the intensive care unit	3&4	None	MIMIC III	N/A	No information	N/A	N/A	Low	Low	Low	Unclear	Unclear
**944**	Shi	2022	United States	Discriminative AI	Predicting readmissions	Predicting 7-day intensive care unit readmission	5	None	Other type of dataset	MIMIC IV	1.000-10.000	10.000 - 100.000	N/A	Low	Low	Low	High	High
**945**	Shi	2023	China	Discriminative AI	Predicting mortality	Predicting intensive care unit mortality	3&4	None	MIMIC III	N/A	No information	N/A	N/A	Low	Low	High	Low	Low
**946**	Shi	2023	China	Discriminative AI	Predicting complications	Predicting acute kidney injury in patients with gastrointestinal bleeding admitted to the intensive care unit	5	None	eICU-CRD	MIMIC IV	1.000-10.000	1.000 - 10.000	N/A	Unclear	Unclear	High	High	High
**947**	Shi	2024	China	Discriminative AI	Predicting complications	Predicting acute kidney injury in patients diagnosed with sepsis in critical care	3&4	None	MIMIC IV	N/A	10.000-100.000	N/A	N/A	Unclear	Unclear	Low	Unclear	Unclear
**948**	Shickel	2021	United States	Discriminative AI	Predicting prognosis	Predicting successful or unsuccessful hospital discharge of patients admitted to the intensive care unit	3&4	None	Internal dataset	N/A	0-100	N/A	N/A	Unclear	Low	Low	High	High
**949**	Shickel	2022	United States	Discriminative AI	Predicting mortality; Predicting readmissions; Assessing clinical notes	Predicting ICU patient outcomes, including mortality and readmission, from EHR data.	3&4	None	Internal dataset	N/A	10.000-100.000	N/A	N/A	N/A	N/A	N/A	N/A	N/A
**950**	Shin	2022	South Korea	Discriminative AI	Predicting mortality	Predicting mortality in the intensive care unit	3&4	None	MIMIC III	N/A	10.000-100.000	N/A	N/A	Low	Low	Low	Unclear	Unclear
**951**	Shin	2023	Korea	Discriminative AI	Predicting complications	Predicting in-hospital cardiac arrest in het emergency department-based intensive care units	5	None	Internal dataset	Internal dataset	100-1000	1.000 - 10.000	N/A	Unclear	Low	Low	Low	Unclear
**952**	Shoeibi	2023	Iran	Discriminative AI	Determining physiological values	Predicting blood pressure in intensive care unit patients	3&4	None	MIMIC II	N/A	10.000-100.000	N/A	N/A	Unclear	Low	Low	High	High
**953**	Shung	2021	United States	Discriminative AI	Predicting need for resource	Predicting need for red blood cell transfusion for patients with acute gastrointestinal bleeding admitted to the intensive care unit	5	None	MIMIC III	eICU-CRD	1.000-10.000	1.000 - 10.000	N/A	Unclear	Low	Low	High	High
**954**	Si	2019	United States	Generative AI (Large language model)	Assessing clinical notes	Improving clinical concept extraction on ICU patient records	3&4	None	MIMIC III	N/A	No information	N/A	N/A	N/A	N/A	N/A	N/A	N/A
**955**	Sikora	2023	United States	Discriminative AI	Improving mechanical ventilation	Predicting prolonged duration of mechanical ventilation	3&4	None	Internal dataset	N/A	100-1000	N/A	N/A	Low	Low	Low	High	High
**956**	Sikora	2023	United States	Discriminative AI	Predicting complications	Predicting fluid overload in intensive care unit patients	3&4	STROBE	Internal dataset	N/A	100-1000	N/A	N/A	Low	Low	Low	High	High
**957**	Sikora	2023	United States	Discriminative AI	Classifying sub-populations	Identifying pharmacophenotyes in intensive care unit patients	3&4	None	Internal dataset	N/A	100-1000	N/A	N/A	N/A	N/A	N/A	N/A	N/A
**958**	Silva	2022	Portugal	Generative AI (Large language model)	Predicting readmissions; Diagnosing	Predicting readmission and diagnoses codes on future admission of intensive care unit patients	3&4	None	MIMIC III	N/A	10.000-100.000	N/A	N/A	N/A	N/A	N/A	N/A	N/A
**959**	Silva	2023	Brazil	Discriminative AI	Determining physiological values	Predicting inspiratory muscle pressure waveforms for	5	None	N/A	Internal dataset	N/A	0-100	N/A	Unclear	Low	High	Unclear	High

© 2025 Berkhout WEM et al. *JAMA Network Open.*

						detection of asynchronies in ventilated patients at the intensive care unit												
**960**	Silveira	2022	Brazil	Discriminative AI	Predicting mortality	Predicting hospital mortality in intensive care unit patients	3&4	None	Other type of dataset	N/A	1.000-10.000	N/A	N/A	Unclear	Low	Low	High	High
**961**	Šín	2022	Czech Republic	Discriminative AI	Predicting complications	Predicting pressure ulcer in intensive care unit patients	3&4	None	MIMIC IV	N/A	1.000-10.000	N/A	N/A	Unclear	Low	Unclear	Unclear	Unclear
**962**	Singh	2021	Japan	Discriminative AI	Predicting mortality; Predicting complications; Predicting length of stay	Predicting sepsis, length of stay and mortality for patients admitted to the intensive care unit	5	None	Other type of dataset	Other type of dataset	10.000-100.000	10.000 - 100.000	N/A	Unclear	Low	High	Unclear	High
**963**	Singh	2022	India	Discriminative AI	Predicting complications	Predicting different stages of sepsis in intensive care unit patients	3&4	None	Internal dataset	N/A	1.000-10.000	N/A	N/A	Unclear	Unclear	Unclear	Unclear	Unclear
**964**	Singhal	2021	United States	Discriminative AI	Predicting complications	Predicting acute respiratory distress syndrome among critically ill adults with COVID-19	5	None	Internal dataset	Internal dataset	10.000-100.000	100-1.000	N/A	Low	Unclear	Unclear	High	High
**965**	Siu	2020	China	Discriminative AI	Predicting need for resource	Predicting the need for intubation for intensive care unit patients	3&4	None	MIMIC III, eICU-CRD	N/A	10.000-100.000	N/A	N/A	Low	Low	Low	Low	Low
**966**	Siuba	2024	United States	Discriminative AI	Classifying sub-populations	Determining subphenotypes of patients with ARDS	5	STROBE	N/A	Other type of dataset	N/A	1.000 - 10.000	N/A	N/A	N/A	N/A	N/A	N/A
**967**	Sjoding	2021	United States	Discriminative AI	Assessing videos and images	Detecting acute respiratory distress syndrome on chest radiographs	5	None	Internal dataset, Other type of dataset	Internal dataset	100.000+	1.000 - 10.000	N/A	Low	Low	Low	Unclear	Unclear
**968**	Smit	2022	Netherlands	Discriminative AI	Predicting mortality	Predicting mortality in COVID-19 patients in the intensive care unit	3&4	None	Other type of dataset	N/A	1.000-10.000	N/A	N/A	Unclear	Low	Low	Low	Unclear
**969**	Soares	2024	Brazil	Discriminative AI	Assessing videos and images	Assisting in the assessment of extravascular pulmonary water and the inferior vena cava collapsibility index in intensive care unit patients with acute kidney injury undergoing kidney replacement therapy	8	None	N/A	N/A	N/A	N/A	50	N/A	N/A	N/A	N/A	N/A
**970**	Soliman	2024	United States	Discriminative AI	Predicting complications	Predicting the onset of septic shock in intensive care unit patients	3&4	None	Internal dataset	N/A	100-1000	N/A	N/A	Unclear	Low	High	Unclear	High
**971**	Sottile	2023	United States	Discriminative AI	Improving mechanical ventilation	Identifying ventilator dyssynchrony	3&4	None	Internal dataset	N/A	1.000-10.000	N/A	N/A	Low	Low	High	High	High
**972**	Soundoulounaki	2023	Greece	Discriminative AI	Improving mechanical ventilation	Identifying weak inspiratory efforts during pressure support ventilation for intensive care unit patients	3&4	None	Internal dataset	N/A	10.000-100.000	N/A	N/A	Unclear	Low	High	High	High
**973**	Srimedha	2022	India	Discriminative AI	Predicting complications	Predicting sepsis in intensive care unit patients	3&4	None	Other type of dataset	N/A	10.000-100.000	N/A	N/A	Unclear	High	Low	Low	Unclear
**974**	Srivastava	2023	Singapore	Discriminative AI	Predicting complications	Predicting sepsis or acute respiratory distress syndrome for intensive care unit patients	3&4	None	MIMIC III	N/A	10.000-100.000	N/A	N/A	Unclear	Low	Unclear	Unclear	Unclear
**975**	Steinmeyer	2020	Germany	Discriminative AI	Predicting mortality	Predicting mortality in intensive care unit	3&4	None	Other type of dataset	N/A	10.000-100.000	N/A	N/A	Unclear	Low	Low	Unclear	Unclear
**976**	Stenwig	2022	Norway	Discriminative AI	Predicting mortality	Predicting hospital mortality in intensive care unit patients	3&4	None	eICU-CRD	N/A	10.000-100.000	N/A	N/A	Low	Low	Low	High	High
**977**	Stenwig	2023	Norway	Discriminative AI	Predicting mortality	Predicting hospital mortality in intensive care unit patients	3&4	None	eICU-CRD	N/A	10.000-100.000	N/A	N/A	Low	Low	Low	Unclear	Unclear
**978**	Stephens	2023	Australia	Discriminative AI	Predicting mortality	Predicting in-hospital mortality for patients treated with venoarterial extracorporeal	5	TRIPOD/ TRIPOD+AI	Other type of dataset	Other type of dataset	10.000-100.000	1.000 - 10.000	N/A	Low	Low	Low	Low	Low

© 2025 Berkhout WEM et al. *JAMA Network Open.*

						membrane oxygenation												
**979**	Strickler	2023	United States	Discriminative AI	Predicting complications	Predicting sepsis in intensive care unit patients	3&4	None	Other type of dataset	N/A	10.000-100.000	N/A	N/A	Unclear	Unclear	Low	Unclear	Unclear
**980**	Strodthoff	2021	Germany	Discriminative AI	Assessing videos and images	Predicting absolute volume, absolute flow, normalized airway pressure, normalized arterial blood pressure and absolute transpulmonary pressure in acute respiratory distress patients admitted to the intensive care unit	3&4	None	Other type of dataset	N/A	100-1000	N/A	N/A	Low	Low	Unclear	Unclear	Unclear
**981**	Su	2020	China	Discriminative AI	Classifying sub-populations	Identifying clinical phenotypes for critically ill patients with mechanical ventilation	5	None	Internal dataset	MIMIC III	1.000-10.000	1.000 - 10.000	N/A	Low	Low	Low	High	High
**982**	Su	2021	China	Discriminative AI	Predicting mortality; Predicting length of stay; Predicting prognosis	Predicting mortality, severity and length of stay in sepsis patients at the intensive care unit	3&4	None	Internal dataset	N/A	1.000-10.000	N/A	N/A	Low	Unclear	Low	High	High
**983**	Su	2022	China	Discriminative AI	Predicting mortality	Predicting 30-day mortality in sepsis patients	3&4	None	MIMIC III	N/A	1.000-10.000	N/A	N/A	Unclear	Unclear	Low	High	High
**984**	Su	2022	China	Discriminative AI (Reinforcement Learning)	Treatment recommendation	Guiding the direction of fluid therapy for sepsis patents	3&4	None	Other type of dataset	N/A	1.000-10.000	N/A	N/A	Low	Unclear	High	Low	High
**985**	Su	2023	China	Discriminative AI	Predicting mortality	Predicting risk of death in patients with systemic lupus erythematosus in the intensive care unit	5	TRIPOD/ TRIPOD+AI	MIMIC IV	MIMIC III	100-1000	100-1.000	N/A	N/A	N/A	N/A	N/A	N/A
**986**	Su	2024	United States	Discriminative AI	Predicting complications; Predicting need for resource	Predicting pulmonary edema, need for mechanical ventilation, administration of diuretics and need for dialysis for sepsis patients in intensive care unit	3&4	None	MIMIC IV	N/A	1.000-10.000	N/A	N/A	Low	Unclear	Low	High	High
**987**	Su	2024	China	Discriminative AI	Predicting mortality	Predicting mortality in sepsis patients in the intensive care unit	5	None	Internal dataset	Internal dataset	1.000-10.000	100-1.000	N/A	N/A	N/A	N/A	N/A	N/A
**988**	Sun	2021	China	Discriminative AI (Reinforcement learning)	Determining physiological thresholds	Personalized vital signs control for sepsis and heart failure	3&4	None	MIMIC III	N/A	10.000-100.000	N/A	N/A	N/A	N/A	N/A	N/A	N/A
**989**	Sun	2021	United States	Discriminative AI	Predicting complications	Predicting acute hypotensive episodes in intensive care unit patients	3&4	None	MIMIC III	N/A	10.000-100.000	N/A	N/A	Unclear	Low	Low	High	High
**990**	Sun	2021	China	Discriminative AI	Determining physiological values	Predicting blood pressure level for intensive care unit patients	3&4	None	MIMIC	N/A	100-1000	N/A	N/A	Unclear	Unclear	Low	Unclear	Unclear
**991**	Sun	2022	China	Discriminative AI	Treatment recommendation, Diagnostic	Predicting diagnosis and medication recommendation for patients admitted to the intensive care unit	3&4	None	MIMIC III	N/A	No information	N/A	N/A	High	Low	High	High	High
**992**	Sun	2022	China	Discriminative AI	Predicting mortality	Predicting mortality in heart failure patients with hypoxic hepatitis admitted to the intensive care unit	5	None	MIMIC IV	eICU-CRD	100-1000	100-1.000	N/A	Low	Low	Low	High	High
**993**	Sun	2023	China	Discriminative AI	Diagnosing	Predicting sepsis and eight diseases in intensive care unit patients	3&4	None	Other type of dataset	N/A	10.000-100.000	N/A	N/A	Unclear	Low	Low	Unclear	Unclear
**994**	Sun	2024	China	Discriminative AI	Predicting complications	Predicting early onset of acute kidney injury in critically ill patients	5	None	Internal dataset	Internal dataset	100-1000	100-1.000	N/A	Unclear	Unclear	Unclear	Unclear	Unclear
**995**	Sun	2023	China	Generative AI (Large language model)	Predicting mortality; Predicting readmissions	Predicting readmission and mortality for intensive care unit patients	3&4	None	eICU-CRD	N/A	No information	N/A	N/A	Unclear	Low	Low	Low	Unclear

© 2025 Berkhout WEM et al. *JAMA Network Open.*

**996**	Sung	2020	Taiwan	Discriminative AI	Predicting complications	Detection of seizures in cardiac arrest survivors	3&4	None	Internal dataset	N/A	100-1000	N/A	N/A	Low	Low	Low	Unclear	Unclear
**997**	Sung	2021	Korea	Discriminative AI	Predicting mortality; Predicting complications	Predicting mortality, sepsis and acute kidney injury for intensive care unit patients	5	TRIPOD/ TRIPOD+AI	Internal dataset	Internal dataset	10.000-100.000	1.000 - 10.000	N/A	Low	Low	High	High	High
**998**	Sushil	2024	United States	Generative AI (Large language model)	Assessing clinical notes	Extracting study variables from intensive care unit patients	3&4	None	MIMIC III	N/A	100-1000	N/A	N/A	Low	Low	High	High	High
**999**	Susianti	2024	Indonesia	Discriminative AI	Predicting complications	Predicting acute kidney injuring in sepsis patients admitted to the intensive care unit the prediction of Acute kidney injury	3&4	None	Internal dataset	N/A	100-1000	N/A	N/A	Low	Unclear	Unclear	High	High
**1000**	Tajgardoon	2020	United States	Discriminative AI	Predicting relevance of clinical information	Predicting relevant information a physician seeks in clinical context in the intensive care unit	3&4	None	Internal dataset	N/A	100-1000	N/A	N/A	Unclear	Unclear	High	Unclear	High
**1001**	Takahashi	2023	Japan	Discriminative AI	Predicting mortality; Predicting prognosis	Predicting in-hospital mortality and neurological outcomes in patients with out-of-hospital cardiac arrest	3&4	TRIPOD/ TRIPOD+AI	Internal dataset	N/A	100-1000	N/A	N/A	Low	Low	Low	Unclear	Unclear
**1002**	Takkavatakarn	2024	United States	Discriminative AI	Classifying sub-populations	Predicting acute kidney injury in patients with sepsis in the intensive care unit	5	None	MIMIC IV	eICU-CRD	1.000-10.000	1.000 - 10.000	N/A	N/A	N/A	N/A	N/A	N/A
**1003**	Tamboli	2024	United States	Discriminative AI (Reinforcement learning)	Treatment recommendation	Recommending sepsis treatment in intensive care unit patients	3&4	None	MIMIC III	N/A	10.000-100.000	N/A	N/A	N/A	N/A	N/A	N/A	N/A
**1004**	Tan	2023	China	Discriminative AI	Classifying sub-populations	Identifying acute kidney injury subphenotypes in the intensive care unit	3&4	None	MIMIC III	N/A	10.000-100.000	N/A	N/A	Low	Unclear	Unclear	Low	Unclear
**1005**	Tan	2023	United States	Discriminative AI	Improving mechanical ventilation	Predicting ventilator associated complications	3&4	None	MIMIC III	N/A	1.000-10.000	N/A	N/A	Low	Unclear	High	High	High
**1006**	Tan	2024	United States	Generative AI (Large language model)	Predicting complications	Predicting acute kidney injury and continuous renal replacement therapy for patients admitted to the intensive care unit	3&4	None	MIMIC IV	N/A	10.000-100.000	N/A	N/A	N/A	N/A	N/A	N/A	N/A
**1007**	Tanaka	2021	Japan	Discriminative AI	Assessing videos and images	Classifying radiograph readings as atelectasis, pleural effusion, pneumonia, or no emergency	3&4	None	Internal dataset	N/A	100-1000	N/A	N/A	Unclear	Low	High	Unclear	High
**1008**	Tang	2020	United States	Discriminative AI	Predicting mortality; Predicting complications	Predicting in-hospital mortality, acute respiratory failure and shock in patients admitted to the intensive care unit	3&4	None	MIMIC III	N/A	1.000-10.000	N/A	N/A	Low	Low	Low	Low	Low
**1009**	Tang	2020	United States	Discriminative AI	Predicting mortality	Predicting mortality in intensive care unit patients	3&4	None	MIMIC III	N/A	10.000-100.000	N/A	N/A	Low	Unclear	Low	High	High
**1010**	Tang	2022	China	Discriminative AI	Determining physiological values	Reconstructing electrocardiogram signals in intensive care unit patients	3&4	None	MIMIC III	N/A	No information	N/A	N/A	Low	Low	Low	High	High
**1011**	Tang	2023	Australia	Discriminative AI	Assessing videos and images	Evaluating the performance of a deep learning model in detecting and classifying the position of central venous catheters, endotracheal tubes, and nasogastric tubes on chest X-rays, with a focus on assessing hidden stratification across different device subtypes.	3&4	None	Internal dataset	N/A	1.000-10.000	N/A	N/A	Low	Unclear	Low	Unclear	Unclear

© 2025 Berkhout WEM et al. *JAMA Network Open.*

**1012**	Tang	2024	China	Discriminative AI	Predicting complications	Predicting sepsis in intensive care unit patients	5	None	eICU-CRD	MIMIC III	10.000-100.000	10.000 - 100.000	N/A	Low	Low	Low	Low	High
**1013**	Tang	2024	China	Discriminative AI	Predicting complications	Predicting onset of delirium in elderly patients following intensive care unit admission	3&4	TRIPOD/ TRIPOD+AI	MIMIC IV	N/A	1.000-10.000	N/A	N/A	Low	Unclear	Low	High	High
**1014**	Tang	2024	China	Discriminative AI	Predicting mortality	Predicting in-hospital mortality in elderly patients with sepsis-associated acute kidney injury	3&4	None	MIMIC IV	N/A	10.000-100.000	N/A	N/A	Unclear	Low	Low	High	High
**1015**	Tao	2023	China	Discriminative AI	Predicting prognosis	Predicting prognosis of comatose patients in the intensive care unit	3&4	None	Internal dataset	N/A	0-100	N/A	N/A	Unclear	Low	Low	High	High
**1016**	Tardiveau	2022	France	Discriminative AI	Predicting mortality	Predicting 28-day mortality of critically ill COVID-19 patients	3&4	None	Other type of dataset	N/A	100-1000	N/A	N/A	Low	Low	Low	High	High
**1017**	Tariq	2023	United States	Discriminative AI	Predicting need for resource	Predicting risk of blood transfusion	5	None	Internal dataset	MIMIC IV	10.000-100.000	0-100	N/A	Unclear	Unclear	Unclear	Unclear	Unclear
**1018**	Tasken	2024	Norway	Discriminative AI	Assessing videos and images	Detecting and tracking the mitral annulus in transesophageal echocardiography in intensive care unit patients	8	None	Internal dataset	Internal dataset	100.000+	0-100	50	Unclear	Low	High	Unclear	High
**1019**	Tasnim	2023	Bangladesh	Discriminative AI	Predicting mortality	Predicting mortality for heart failure patients at the intensive care unit	3&4	None	MIMIC III	N/A	1.000-10.000	N/A	N/A	Unclear	Unclear	Low	Unclear	Unclear
**1020**	Tavakolian	2023	Iran	Discriminative AI	Predicting length of stay	Predicting length of stay in intensive care unit patients	3&4	None	MIMIC III	N/A	1.000-10.000	N/A	N/A	Unclear	Unclear	Low	Unclear	Unclear
**1021**	Tehrany	2023	Iran	Discriminative AI	Predicting complications	Investigating the risk predictions of hospital-acquired pressure injury (HAPI) in ICU patients using various machine learning algorithms	3&4	None	No information	N/A	No information	N/A	N/A	Unclear	High	Low	High	High
**1022**	Tewarie	2023	Netherlands	Discriminative AI	Predicting prognosis	Predicting clinical outcome in patients with moderate to severe traumatic brain injury	3&4	None	Internal dataset	N/A	100-1000	N/A	N/A	Low	Low	Low	Low	Low
**1023**	Thoral	2021	Netherlands	Discriminative AI	Predicting mortality; Predicting readmissions	Predicting ICU readmission and/or death within 7 days of ICU discharge for adult ICU patients to optimize timing of ICU discharge	5	None	AmsterdamUMCdb	Internal dataset	10.000-100.000	1.000 - 10.000	N/A	Low	Low	Low	Low	Low
**1024**	Thorsen-Meyer	2020	Denmark	Discriminative AI	Predicting mortality	Predicting 90-day mortality for patients in the intensive care unit	5	TRIPOD/ TRIPOD+AI	Internal dataset	Internal dataset	10.000-100.000	1.000 - 10.000	N/A	Low	Low	Low	Unclear	Unclear
**1025**	Thorsen-Meyer	2022	Denmark	Discriminative AI	Predicting mortality; Improving prognostic models/risk scoring system	Predicting survival probability at different time points for adult ICU patients using heterogeneous electronic health record data	3&4	TRIPOD/ TRIPOD+AI	Internal dataset	N/A	10.000-100.000	N/A	N/A	Low	Low	Low	Low	Low
**1026**	Tian	2022	China	Discriminative AI	Predicting mortality	Predicting 3-month mortality for non-traumatic neurological ICU patients using a machine learning model based on QEEG parameters, APACHE II score, and clinical data.	3&4	None	Internal dataset	N/A	100-1000	N/A	N/A	Low	Low	Low	High	High
**1027**	Tian	2023	China	Discriminative AI	Predicting complications	Predicting delirium in intensive care unit patients	3&4	None	MIMIC III	N/A	1.000-10.000	N/A	N/A	Unclear	Unclear	Low	Unclear	Unclear
**1028**	Tian	2023	China	Discriminative AI	Predicting complications	Predicting acute kidney injury in patients with liver cirrhosis in the intensive care unit	3&4	None	MIMIC III, MIMIC IV	N/A	100-1000	N/A	N/A	Low	Low	Low	Low	Low

© 2025 Berkhout WEM et al. *JAMA Network Open.*

**1029**	Tianshu	2020	China	Discriminative AI	Predicting mortality; Predicting length of stay; Diagnosing	Predicting mortality, length of stay and disease classification for intensive care unit patients	3&4	None	MIMIC III	N/A	No information	N/A	N/A	Low	Low	Low	High	High
**1030**	Toh	2023	Singapore	Discriminative AI	Predicting mortality; Predicting complications	Predicting intracranial hypertensive episodes and mortality in critically ill neurosurgical patients using intracranial pressure variability (ICPV) and recurrent neural network-long short-term memory (RNN-LSTM) models	3&4	None	eICU-CRD	N/A	100-1000	N/A	N/A	Low	Low	Low	Low	Low
**1031**	Tol	2024	Netherlands	Discriminative AI	Predicting complications	To evaluate the performance of the Hypotension Prediction Index (HPI) in predicting hypotension in spontaneously breathing, non-ventilated patients in the Post-Anesthesia Care Unit (PACU) and ICU settings.	5	None	N/A	Other type of dataset	N/A	100-1000	N/A	Low	Low	Low	Unclear	Low
**1032**	Toledo	2024	Brazil	Discriminative AI	Predicting need for resource	Predict bed bath time for ICU patients	3&4	None	Internal dataset	N/A	0-100	N/A	N/A	Unclear	Low	Low	High	High
**1033**	Tootooni	2024	United States	Discriminative AI	Predicting complications	Predicting risk of inappropriate vancomycin trough levels for intensive care unit patients	3&4	None	Internal dataset	N/A	1.000-10.000	N/A	N/A	Unclear	Low	Low	Low	Unclear
**1034**	Tran	2023	United States	Discriminative AI	Predicting complications	Predicting onset of sepsis among patients at the intensive care unit	3&4	None	MIMIC IV	N/A	10.000-100.000	N/A	N/A	Low	Low	Low	High	High
**1035**	Triana	2021	United States	Discriminative AI	Predicting length of stay	Predicting length of stay of coronary artery bypass grafting patients	3&4	TRIPOD/ TRIPOD+AI	Other type of dataset	N/A	1.000-10.000	N/A	N/A	Unclear	Unclear	Low	Unclear	Unclear
**1036**	Tsai	2022	Taiwan	Discriminative AI	Predicting complications	Predicting the appropriateness of endotracheal tube placement in critically ill patients based on the distance from key anatomical points on chest X-rays.	3&4	None	Internal dataset	N/A	100-1000	N/A	N/A	Low	Low	Low	Low	Low
**1037**	Tsai	2022	Taiwan	Discriminative AI	Predicting relevance of clinical information	Identifying medication triggers associated with early palliative care team consultation in ICU patients undergoing withdrawal from life-sustaining treatments	3&4	None	Internal dataset	N/A	100-1000	N/A	N/A	N/A	N/A	N/A	N/A	N/A
**1038**	Tsiklidis	2022	United States	Discriminative AI	Predicting mortality	Predicting patient mortality for trauma patients in the intensive care unit	3&4	None	MIMIC III	N/A	1.000-10.000	N/A	N/A	Low	Low	Low	Unclear	Unclear
**1039**	Tu	2023	Taiwan	Discriminative AI	Predicting mortality	Predicting mortality in the intensive care unit for patients with traumatic brain injury	3&4	TRIPOD/ TRIPOD+AI	Internal dataset	N/A	1.000-10.000	N/A	N/A	Low	Low	Low	High	High
**1040**	Urner	2021	Canada	Discriminative AI	Predicting complications	To determine whether routinely collected clinical variables can predict diaphragmatic contractility and stratify the risk of diaphragm atrophy (> 10% decrease in thickness from baseline) in mechanically ventilated patients.	3&4	None	Internal dataset	N/A	100-1000	N/A	N/A	Low	Low	Low	Low	Low
**1041**	Vagliano	2021	Netherlands	Discriminative AI	Predicting complications	Predicting acute kidney injury in intensive care unit patients	3&4	None	MIMIC III	N/A	10.000-100.000	N/A	N/A	Low	Low	Low	High	High

© 2025 Berkhout WEM et al. *JAMA Network Open.*

**1042**	Vagliano	2022	Netherlands	Discriminative AI	Predicting mortality	Predict mortality for COVID-19 patients admitted to the intensive care unit	5	None	Internal dataset	Internal dataset	100-1000	100-1.000	N/A	N/A	N/A	N/A	N/A	N/A
**1043**	Vagliano	2022	Netherlands	Discriminative AI	Predicting mortality	Predicting in-hospital mortality of triage COVID-19 patients at intensive care unit admission	3&4	TRIPOD/ TRIPOD+AI	Other type of dataset	N/A	1.000-10.000	N/A	N/A	Low	Low	High	Unclear	High
**1044**	Vagliano	2022	Netherlands	Discriminative AI	Predicting complications	To externally validate five machine learning models (logistic regression, random forest, gradient boosted trees, and two variants of LSTM) to predict Acute Kidney Injury (AKI) in ICU patients. The models were initially developed on the MIMIC dataset and validated on the eICU-CRD dataset.	5	None	MIMIC III	eICU-CRD	10.000-100.000	100.000+	N/A	Unclear	Low	Low	High	High
**1045**	Vagliano	2022	Netherlands	Discriminative AI	Predicting complications	Developing and internally validating an LSTM-based AI model to predict acute kidney injury (AKI) in ICU patients using clinical variables and notes.	3&4	None	MIMIC III	N/A	10.000-100.000	N/A	N/A	Low	Low	Low	Low	Low
**1046**	Vagliano	2023	Netherlands	Discriminative AI	Predicting mortality; Classifying sub-populations	Find clinical subgroups for COVID-19 patients at the ICU	3&4	TRIPOD/ TRIPOD+AI	Internal dataset	N/A	10.000-100.000	N/A	N/A	Low	Low	Low	Low	Low
**1047**	Vaid	2021	United States	Discriminative AI	Predicting complications	Predicting treatment with dialysis or death	5	None	Internal dataset	Internal dataset	1.000-10.000	No information	N/A	Low	Low	Low	Unclear	Unclear
**1048**	Valderrama	2022	Canada	Discriminative AI	Determining physiological values	Predicting abnormal laboratory blood test results in the intensive care unit	3&4	None	Internal dataset	N/A	10.000-100.000	N/A	N/A	Low	Low	High	Unclear	High
**1049**	van de Kamp	2024	Netherlands	Discriminative AI	Improving mechanical ventilation	Automatic detection of patient-ventilator asynchrony	3&4	None	Internal dataset	N/A	1.000-10.000	N/A	N/A	Unclear	Low	Low	High	High
**1050**	van de Sande	2020	Netherlands	Discriminative AI	Predicting complications	Predicting risk of thromboembolic complications of COVID-19 patients	3&4	TRIPOD/ TRIPOD+AI	Internal dataset	N/A	100-1000	N/A	N/A	Low	Low	Low	High	High
**1051**	van der Ven	2022	Netherlands	Discriminative AI	Predicting complications	Predicting hypotension in mechanically ventilated patients with COVID-19 admitted to the intensive care unit	5	STROBE, STARD/ STARD-AI	N/A	Internal dataset	N/A	0-100	N/A	Low	Low	High	Unclear	High
**1052**	Varudo	2022	Switzerland	Discriminative AI	Assessing videos and images	Assessment of left ventricular ejection fraction	8	None	N/A	N/A	N/A	N/A	95	N/A	N/A	N/A	N/A	N/A
**1053**	Vázquez	2021	Mexico	Discriminative AI	Predicting mortality	Predicting in-hospital mortality for acute coronary syndrome patients in the intensive care unit	3&4	None	MIMIC III	N/A	1.000-10.000	N/A	N/A	Unclear	Low	Low	High	High
**1054**	Venturini	2024	Belgium	Discriminative AI	Predicting need for resource	Predicting the time-to-intubation after critical care admission	3&4	None	Internal dataset	N/A	1.000-10.000	N/A	N/A	Low	Low	Low	High	High
**1055**	Verhaeghe	2022	Belgium	Discriminative AI	Determining physiological values	Predicting piperacillin plasma concentration in critically ill patients	5	None	Internal dataset	Internal dataset	100-1000	0-100	N/A	Unclear	Low	Low	Low	Unclear
**1056**	Verhaeghe	2023	Belgium	Discriminative AI	Predicting complications	Predicting risk of artrial fibrillation for intensive care unit patients	5	None	AmsterdamUMCdb	Internal dataset, MIMIC-IV	10.000-100.000	10.000 - 100.000	N/A	Low	Low	High	Unclear	High
**1057**	Verma	2020	India	Discriminative AI	Predicting mortality	Predicting mortality in intensive care unit patients	3&4	None	Other type of dataset	N/A	1.000-10.000	N/A	N/A	Unclear	Unclear	Low	High	Unclear
**1058**	Vigia	2023	Portugal	Discriminative AI	Predicting complications	To predict pancreas allograft rejection in patients undergoing simultaneous pancreas-kidney transplantation.	3&4	None	Internal dataset	N/A	0-100	N/A	N/A	Low	Low	Low	Low	Low
**1059**	Vigneron	2022	France	Discriminative AI	Assessing videos and images	Measuring muscle areas normalized by	5	None	N/A	Internal dataset	N/A	100-1.000	N/A	Low	Low	High	Unclear	High

© 2025 Berkhout WEM et al. *JAMA Network Open.*

						height in intensive care unit patients												
**1060**	Villar	2023	Spain	Discriminative AI	Predicting mortality	Predicting intensive care unit death in patients with acute respiratory distress syndrome	5	TRIPOD/ TRIPOD+AI	Other type of dataset	Other type of dataset	1.000-10.000	100-1.000	N/A	Low	Low	Low	High	High
**1061**	Villar	2024	Spain	Discriminative AI	Predicting need for resource	Predicting the length of mechanical ventilation in acute respiratory disease syndrome	5	TRIPOD/ TRIPOD+AI	Other type of dataset	Other type of dataset	1.000-10.000	100-1.000	N/A	Low	Low	Low	High	High
**1062**	Vornhagen	2023	United States	Discriminative AI	Predicting complications	Predicting infection in Klebsiella-colonized ICU patients based on gut microbiota composition using machine learning models.	3&4	None	Internal dataset	N/A	100-1000	N/A	N/A	Low	Low	Low	Low	Low
**1063**	Wang	2020	United States	Discriminative AI	Predicting mortality	Predicting mortality for ICU patients	3&4	None	MIMIC III	N/A	10.000-100.000	N/A	N/A	Low	Low	Low	Unclear	Low
**1064**	Wang	2020	United Kingdom	Discriminative AI	Diagnostic	Predicting multiple disease risks for patients	3&4	None	MIMIC III	N/A	1.000-10.000	N/A	N/A	Low	Unclear	Unclear	High	High
**1065**	Wang	2020	China	Discriminative AI	Predicting readmissions	Predicting intensive care unit readmission	3&4	None	Internal dataset	N/A	1.000-10.000	N/A	N/A	Low	Low	Low	High	High
**1066**	Wang	2020	China	Discriminative AI	Predicting complications	Predicting acute kidney injury 24 or 48 hours ahead in intensive care unit patients	5	None	Internal dataset	MIMIC III	10.000-100.000	10.000 - 100.000	N/A	Low	Low	Low	Unclear	Unclear
**1067**	Wang	2021	China	Discriminative AI	Predicting complications	Predicting early onset of sepsis in intensive care unit patients	3&4	None	Internal dataset	N/A	1.000-10.000	N/A	N/A	Low	Unclear	Unclear	Unclear	Unclear
**1068**	Wang	2021	China	Discriminative AI	Assessing videos and images	Monitoring neuroendocrine changes in patients with severe brain injury using transcranial Doppler ultrasound enhanced	3&4	None	Internal dataset	N/A	0-100	N/A	N/A	Unclear	Low	Low	High	High
**1069**	Wang	2022	China	Discriminative AI	Assessing videos and images	Image segmentation in liver computed tomography image segmentation and analyzing the clinical features of acute liver injury caused by sepsis	3&4	None	Internal dataset	N/A	0-100	N/A	N/A	Low	Unclear	High	Unclear	High
**1070**	Wang	2022	United States	Discriminative AI	Predicting mortality	Predicting in-hospital mortality in sepsis patients in the intensive care unit	3&4	None	MIMIC III	N/A	1.000-10.000	N/A	N/A	Low	Low	Low	High	High
**1071**	Wang	2022	China	Discriminative AI	Predicting length of stay	Predicting ICU length of stay for heart transplantation patients	3&4	None	Internal dataset	N/A	100-1000	N/A	N/A	Unclear	Low	Low	High	High
**1072**	Wang	2022	China	Discriminative AI	Predicting mortality	Predicting 30-day mortality in sepsis patients at the intensive care unit	3&4	None	MIMIC IV	N/A	10.000-100.000	N/A	N/A	Low	Unclear	Low	High	High
**1073**	Wang	2022	China	Discriminative AI	Improving mechanical ventilation	Predicting mechanical ventilation duration for acute respiratory distress syndrome patients	5	None	MIMIC IV	AmsterdamUMCdb, eICU-CRD	1.000-10.000	1.000 - 10.000	N/A	Low	Unclear	Low	Low	Unclear
**1074**	Wang	2022	China	Discriminative AI	Predicting mortality	Predicting in-hospital mortality among patients with acute myocardial infarction	3&4	TRIPOD/ TRIPOD+AI	MIMIC IV	N/A	1.000-10.000	N/A	N/A	Low	Low	Low	Low	Low
**1075**	Wang	2022	China	Discriminative AI	Improving mechanical ventilation	Predicting noninvasive ventilation failure after extubation	5	TRIPOD/ TRIPOD+AI	eICU-CRD	Other type of dataset	100-1000	100-1.000	N/A	Low	Low	Low	Low	Low
**1076**	Wang	2022	China	Discriminative AI	Predicting mortality	Predicting death probability of intensive care unit patients	3&4	None	MIMIC III	N/A	10.000-100.000	N/A	N/A	Unclear	Unclear	Low	Unclear	Unclear
**1077**	Wang	2022	United States	Discriminative AI	Predicting complications	Predicting sepsis in intensive care unit patients	3&4	None	Other type of dataset	N/A	10.000-100.000	N/A	N/A	Unclear	Low	Unclear	Unclear	Unclear

© 2025 Berkhout WEM et al. *JAMA Network Open.*

**1078**	Wang	2022	China	Discriminative AI	Predicting mortality	Predicting time to death in coronary care unit patients	3&4	None	MIMIC III	N/A	1.000-10.000	N/A	N/A	Low	Low	Low	High	High
**1079**	Wang	2023	China	Discriminative AI	Predicting mortality	Predicting mortality of hemorrhagic stroke patients	3&4	TRIPOD/ TRIPOD+AI	MIMIC IV	N/A	1.000-10.000	N/A	N/A	Low	Low	Low	High	High
**1080**	Wang	2023	China	Discriminative AI	Predicting length of stay	Predicting length of stay of patients undergoing craniotomy and requiring intensive care unit treatments	3&4	None	Internal dataset	N/A	100-1000	N/A	N/A	Low	Low	Low	Low	Low
**1081**	Wang	2023	China	Discriminative AI	Predicting complications	Predicting multidrug-resistant organism (MDRO) infection	3&4	None	Internal dataset	N/A	100-1000	N/A	N/A	Low	High	High	High	High
**1082**	Wang	2023	United States	Discriminative AI	Classifying sub-populations	Defining physiological phenotypes	3&4	TRIPOD/ TRIPOD+AI	Internal dataset	N/A	100-1000	N/A	N/A	N/A	N/A	N/A	N/A	N/A
**1083**	Wang	2023	China	Discriminative AI	Predicting mortality	Predicting 28-day mortality of septic patients with atrial fibrillation	5	TRIPOD/ TRIPOD+AI	MIMIC IV	MIMIC III	1.000-10.000	1.000 - 10.000	N/A	Unclear	Low	Unclear	High	High
**1084**	Wang	2023	Taiwan	Discriminative AI	Predicting complications	Predicting delirium in critically ill adult patients	3&4	TRIPOD/ TRIPOD+AI	Internal dataset	N/A	1.000-10.000	N/A	N/A	Low	Low	Low	Low	Low
**1085**	Wang	2023	China	Discriminative AI	Classifying sub-populations	Classifying cardiogenic shock phenotypes in the intensive care unit	3&4	None	eICU-CRD	N/A	10.000-100.000	N/A	N/A	Unclear	Low	Low	Unclear	Unclear
**1086**	Wang	2023	China	Discriminative AI	Predicting need for resource	Predicting requirement for invasive mechanical ventilation in intensive care unit patients	3&4	None	MIMIC III	N/A	1.000-10.000	N/A	N/A	Low	Low	Low	Low	Low
**1087**	Wang	2023	China	Discriminative AI	Predicting need for resource	Predicting enteral nutrition initiation for patients in the intensive care unit	3&4	TRIPOD/ TRIPOD+AI	MIMIC IV	N/A	10.000-100.000	N/A	N/A	N/A	N/A	N/A	N/A	N/A
**1088**	Wang	2023	China	Discriminative AI	Predicting complications	Predicting ventilator associated pneumonia in traumatic brain injury patients	3&4	None	MIMIC III	N/A	100-1000	N/A	N/A	Low	Unclear	Low	Unclear	Unclear
**1089**	Wang	2023	China	Discriminative AI	Predicting mortality	Predicting 30-day mortality of critical pulmonary embolism patients	3&4	None	MIMIC IV	N/A	1.000-10.000	N/A	N/A	Unclear	Low	Low	Unclear	Unclear
**1090**	Wang	2023	China	Discriminative AI	Predicting mortality	Predicting mortality in pneumonia patients on admission to the intensive care unit	5	None	MIMIC IV	eICU-CRD	1.000-10.000	10.000 - 100.000	N/A	Unclear	Low	High	Unclear	High
**1091**	Wang	2023	China	Discriminative AI	Predicting complications	Predicting acute respiratory distress syndrome in traumatic brain injury patients	3&4	None	MIMIC III	N/A	100-1000	N/A	N/A	Low	Low	High	High	High
**1092**	Wang	2023	China	Discriminative AI	Predicting mortality	Predicting mortality in geriatric traumatic brain injury patients	3&4	None	MIMIC III	N/A	100-1000	N/A	N/A	N/A	N/A	N/A	N/A	N/A
**1093**	Wang	2023	United States	Discriminative AI	Predicting prognosis	Predicting prognosis of herpes simplex virus pneumonia	3&4	None	Internal dataset	N/A	100-1000	N/A	N/A	Low	Unclear	Low	Unclear	Unclear
**1094**	Wang	2023	Sweden	Discriminative AI	Treatment recommendation	Preventing mortality of intensive care unit patients by suggesting counterfactual configurations of patient history	3&4	None	MIMIC III	N/A	1.000-10.000	N/A	N/A	Low	Unclear	Low	Unclear	Unclear
**1095**	Wang	2023	China	Discriminative AI	Predicting complications	Predicting major adverse kidney events in elderly patients in critical care	3&4	None	Internal dataset	N/A	1.000-10.000	N/A	N/A	Low	Low	Low	High	High
**1096**	Wang	2024	China	Discriminative AI	Assessing videos and images	Providing segmentation of physiological state images and classification of patients' disease level	3&4	None	No information	N/A	No information	N/A	N/A	Unclear	Unclear	High	Unclear	Unclear
**1097**	Wang	2024	China	Discriminative AI	Predicting complications	Predicting the probability of pelvic inflammatory disease progression to sepsis	3&4	TRIPOD/ TRIPOD+AI	MIMIC IV	N/A	1.000-10.000	N/A	N/A	Low	Low	Low	Low	Low
**1098**	Wang	2024	China	Discriminative AI	Improving mechanical ventilation	Predicting high-flow nasal oxygen failure in acute respiratory failure	3&4	None	Internal dataset	N/A	100-1000	N/A	N/A	Low	Unclear	Low	High	High

© 2025 Berkhout WEM et al. *JAMA Network Open.*

**1099**	Wang	2024	Austria	Discriminative AI	Predicting complications	Predicting intensive care unit-acquired weakness	3&4	None	Internal dataset	N/A	1.000-10.000	N/A	N/A	Low	Low	Low	High	High
**1100**	Wanyan	2021	United States	Discriminative AI	Predicting mortality	Predicting in-hospital mortality in intensive care unit patients	3&4	None	MIMIC III	N/A	1.000-10.000	N/A	N/A	Unclear	Unclear	Low	Unclear	Unclear
**1101**	Wanyan	2021	United States	Discriminative AI	Predicting mortality	Predicting mortality in COVID-19 in the intensive care unit	3&4	None	Internal dataset	N/A	1.000-10.000	N/A	N/A	Unclear	Low	Low	High	High
**1102**	Wei	2022	China	Discriminative AI	Predicting complications	Predicting acute kidney injury progression in critically ill patients	3&4	None	MIMIC III	N/A	10.000-100.000	N/A	N/A	Low	Low	High	Low	High
**1103**	Wei	2023	China	Discriminative AI	Predicting complications	Predicting acute kidney injury in patients with acute respiratory distress syndrome	5	None	MIMIC III	MIMIC IV	100-1000	100-1.000	N/A	Low	Unclear	Low	Unclear	Unclear
**1104**	Weisman Raymond	2023	Israel	Discriminative AI	Predicting an event	Predicting hypoglycemia, hypokalemia and hypotension treatment in the intensive care unit	3&4	None	MIMIC IV	N/A	No information	N/A	N/A	Unclear	Unclear	Unclear	Unclear	Unclear
**1105**	Wen	2024	China	Discriminative AI	Predicting mortality	Prediction of 28-day mortality in patients with Sepsis-Associated Liver Injury	5	TRIPOD/ TRIPOD+AI	MIMIC IV	MIMIC III	1.000-10.000	100-1.000	N/A	Unclear	Low	Low	High	High
**1106**	Weng	2017	United States	Discriminative AI (Reinforcement learning)	Treatment recommendation	Recommending optimal targeted blood glucose levels	3&4	None	MIMIC III	N/A	1.000-10.000	N/A	N/A	N/A	N/A	N/A	N/A	N/A
**1107**	Wernly	2021	Sweden	Discriminative AI	Predicting mortality	Predicting intensive care unit mortality in sepsis or septic shock patients	3&4	None	eICU-CRD	N/A	1.000-10.000	N/A	N/A	Low	Low	Low	Unclear	Unclear
**1108**	Whitlock	2021	United States	Discriminative AI	Predicting need for resource	Predicting discharge to a postacute care facility	3&4	None	Internal dataset	N/A	100-1000	N/A	N/A	Unclear	Low	Low	High	High
**1109**	Wieringa	2023	Netherlands	Discriminative AI	Predicting complications; Predicting medication administration	Developing models to predict beta-lactam target non-attainment within 12–36 hours after antibiotic initiation in ICU patients	5	TRIPOD/ TRIPOD+AI	Internal dataset	Internal dataset	100-1000	100-1.000	N/A	Low	Low	Low	Low	Low
**1110**	Woillard	2020	Switzerland	Discriminative AI	Determining physiological values	Estimating glomerular filtration rate estimation in intensive care unit patients	3&4	None	Other type of dataset	N/A	0-100	N/A	N/A	Unclear	Unclear	High	Unclear	High
**1111**	Wollborn	2021	United States	Discriminative AI	Predicting complications	Predicting Capillary Leak Syndrome in surgical critical ill patients	3&4	TRIPOD/ TRIPOD+AI	Internal dataset	N/A	100-1000	N/A	N/A	Low	Unclear	Low	High	High
**1112**	Wollborn	2023	United States	Discriminative AI	Predicting mortality; Predicting complications	Predicting acute kidney injury, mortality, low P/F ratio, dependence on vasoactive drugs and ECMO dependence	3&4	STROBE	Internal dataset	N/A	No information	N/A	N/A	Low	Unclear	Unclear	High	High
**1113**	Wong	2023	United States	Discriminative AI	Assessing videos and images	Checking endotracheal tube placement on chest x-rays	8	None	N/A	N/A	N/A	N/A	214	N/A	N/A	N/A	N/A	N/A
**1114**	Wu	2021	China	Discriminative AI	Predicting mortality	Predict hospital death for patients with acute mesentric ischemia	3&4	TRIPOD/ TRIPOD+AI	MIMIC III	N/A	100-1000	N/A	N/A	N/A	N/A	N/A	N/A	N/A
**1115**	Wu	2021	China	Discriminative AI	Predicting prognosis	Predicting prolonged length of intensive care unit stay	5	None	eICU-CRD	MIMIC III	100.000+	10.000 - 100.000	N/A	Low	Low	Low	High	High
**1116**	Wu	2021	China	Discriminative AI	Predicting mortality	Predicting in-hospital mortality in intensive care patients with sepsis	5	TRIPOD/ TRIPOD+AI	MIMIC III	Other type of dataset	1.000-10.000	1.000 - 10.000	N/A	Unclear	Low	High	Unclear	High
**1117**	Wu	2022	Taiwan	Discriminative AI	Assessing videos and images	Classifying pain in critically ill patients	3&4	None	Internal dataset	N/A	0-100	N/A	N/A	Unclear	Unclear	Unclear	Unclear	Unclear
**1118**	Wu	2022	China	Discriminative AI	Predicting complications	Predicting inhalation-induced acute respiratory distress syndrome	5	STROBE	eICU-CRD	MIMIC-III, Other type of dataset	0-100	0-100	N/A	Low	Unclear	Low	Low	Unclear
**1119**	Wu	2023	United States	Discriminative AI	Predicting deterioration	Predicting deterioration in	6	STROBE	N/A	N/A	N/A	N/A	N/A	Low	Unclear	High	High	High

© 2025 Berkhout WEM et al. *JAMA Network Open.*

						critically injured patients												
**1120**	Wu	2023	China	Discriminative AI	Predicting complications	Predicting incidence and severity of acute respiratory distress syndrome for intensive care unit patients	3&4	None	eICU-CRD	N/A	1.000-10.000	N/A	N/A	Low	Unclear	High	High	High
**1121**	Wu	2023	China	Discriminative AI	Predicting complications	Predicting acute kidney injury in patients in the neurointensive care unit after brain surgery	3&4	TRIPOD/ TRIPOD+AI	Internal dataset	N/A	100-1000	N/A	N/A	Low	Low	Unclear	High	High
**1122**	Wu	2023	China	Discriminative AI	Predicting complications	Predicting cognitive impairment related to post intensive care syndrome	3&4	JMIR Guidelines for Developing and Reporting Machine Learning Predictive Models in Biomedical Research	Internal dataset	N/A	100-1000	N/A	N/A	N/A	N/A	N/A	N/A	N/A
**1123**	Wu	2023	United States	Discriminative AI	Predicting complications	Predicting acute kidney injury in intensive care unit patients	3&4	None	MIMIC III	N/A	10.000-100.000	N/A	N/A	Unclear	Low	Low	High	High
**1124**	Wu	2023	Taiwan	Discriminative AI	Assessing videos and images	Predicting mortality in COVID-19 patients at the intensive care unit	3&4	None	Internal dataset	N/A	0-100	N/A	N/A	Low	Low	Low	Low	Low
**1125**	Wu	2024	Netherlands	Discriminative AI	Predicting complications	Predicting the onset of sepsis in intensive care patients	5	TRIPOD/ TRIPOD+AI	Other type of dataset	Internal dataset	1.000-10.000	100-1.000	N/A	Low	Unclear	High	Unclear	High
**1126**	Wu	2024	United States	Discriminative AI	Predicting mortality	Predicting ICU mortality or 90-day mortality in critically ill patients with COVID-19	3&4	None	Other type of dataset	N/A	100-1000	N/A	N/A	Unclear	Low	Low	Unclear	Unclear
**1127**	Wu	2024	Taiwan	Discriminative AI	Predicting an event	Monitoring brain anomalies	3&4	None	Internal dataset	N/A	0-100	N/A	N/A	Unclear	Low	High	Unclear	High
**1128**	Wu	2023	China	Discriminative AI (Reinforcement learning)	Treatment recommendation	Optimal treatment of sepsis patients	5	None	MIMIC III	eICU-CRD	10.000-100.000	10.000 - 100.000	N/A	Unclear	Unclear	High	Unclear	High
**1129**	Xia	2019	China	Discriminative AI	Predicting mortality	Predicting intensive care unit mortality	3&4	None	MIMIC III	N/A	10.000-100.000	N/A	N/A	Low	Unclear	Low	Unclear	Unclear
**1130**	Xia	2020	China	Discriminative AI	Predicting prognosis	Predicting clinical outcome at discharge after rupture of anterior communicating artery aneurysm	5	None	Internal dataset	Internal dataset	100-1000	100-1.000	N/A	Low	Low	High	Unclear	High
**1131**	Xia	2022	China	Discriminative AI	Predicting complications	Predicting hypoxemia after extubation in the intensive care unit	3&4	TRIPOD/ TRIPOD+AI	MIMIC IV	N/A	10.000-100.000	N/A	N/A	Low	Unclear	Low	Unclear	Unclear
**1132**	Xian	2023	Canada	Discriminative AI	Predicting mortality; Predicting length of stay	Predicting mortality and length of stay of intensive care unit patients	3&4	None	Internal dataset	N/A	10.000-100.000	N/A	N/A	Unclear	Low	Low	Low	Unclear
**1133**	Xiao	2024	China	Discriminative AI	Predicting deterioration	Predicting new-onset function impairment in patients in the intensive care unit	5	TRIPOD/ TRIPOD+AI	Internal dataset	Internal dataset	1.000-10.000	100-1.000	N/A	Low	Low	Low	High	High
**1134**	Xie	2020	Singapore	Discriminative AI	Predicting mortality	Predicting mortality in intensive care unit patients	3&4	None	MIMIC III	N/A	10.000-100.000	N/A	N/A	Low	Low	Low	Low	Low
**1135**	Xie	2022	Singapore	Discriminative AI	Predicting mortality	Predicting mortality in intensive care unit patients	3&4	None	MIMIC III	N/A	10.000-100.000	N/A	N/A	Unclear	Low	Low	Unclear	Unclear
**1136**	Xie	2023	China	Discriminative AI	Predicting complications	Predict mortality for patients with diabetic ketoacidosis	5	None	Internal dataset, MIMIC IV, eICU-CRD	Internal dataset, eICU-CRD	1.000-10.000	1.000 - 10.000	N/A	Low	Unclear	Low	Unclear	Unclear
**1137**	Xie	2024	China	Discriminative AI	Predicting mortality	Predicting in-hospital mortality for patients with acute myocardial infarction	5	None	Internal dataset	MIMIC IV	10.000-100.000	1.000 - 10.000	N/A	Low	Low	Low	High	High
**1138**	Xinsai	2022	China	Discriminative AI	Predicting complications	Predicting postoperative acute kidney injury in patients with Type A	3&4	None	Internal dataset	N/A	1.000-10.000	N/A	N/A	Low	High	High	High	High

© 2025 Berkhout WEM et al. *JAMA Network Open.*

						or B acute aortic dissection												
**1139**	Xu	2018	United States	Discriminative AI	Predicting length of stay; Predicting deterioration	Predicting decompensation and length of stay in patients at the intensive care unit	3&4	None	MIMIC III	N/A	10.000-100.000	N/A	N/A	Unclear	Unclear	Unclear	Unclear	Unclear
**1140**	Xu	2022	China	Discriminative AI	Predicting mortality	Predicting early mortality in Acinetobacter baumannii complex-caused bloodstream infection	3&4	None	Internal dataset	N/A	100-1000	N/A	N/A	Low	Low	Low	High	High
**1141**	Xu	2022	China	Discriminative AI	Predicting mortality	Predicting mortality in rheumatic heart disease patients	3&4	None	MIMIC IV	N/A	100-1000	N/A	N/A	Low	Low	High	High	High
**1142**	Xu	2023	United States	Discriminative AI	Classifying sub-populations	Clustering sepsis patients	3&4	None	MIMIC IV	N/A	1.000-10.000	N/A	N/A	N/A	N/A	N/A	N/A	N/A
**1143**	Xu	2024	China	Discriminative AI	Predicting deterioration	Identify the tendency for hemodynamic deterioration	3&4	None	MIMIC IV	N/A	100-1000	N/A	N/A	Low	Unclear	Low	High	High
**1144**	Xu	2024	China	Discriminative AI	Predicting complications	Predicting the occurrence of pressure injury in diabetic patients at the intensive care unit	3&4	None	Internal dataset	N/A	100-1000	N/A	N/A	Low	Low	Low	Unclear	Unclear
**1145**	Xue	2019	United States	Discriminative AI	Predicting need for resource	Predicting readmission to the ICU	3&4	None	MIMIC II	N/A	1.000-10.000	N/A	N/A	Unclear	Low	Low	High	High
**1146**	Yadav	2021	India	Discriminative AI	Classifying sub-populations	Diagnosis of cardiac arrhythmia	3&4	None	MIMIC III	N/A	10.000-100.000	N/A	N/A	Low	Low	Low	High	High
**1147**	Yallabandi	2023	India	Discriminative AI	Predicting mortality	Predicting mortality in intensive care unit patients	3&4	None	Internal dataset	N/A	100-1000	N/A	N/A	Low	Low	Low	High	High
**1148**	Yamano	2024	Japan	Discriminative AI	Predicting complications	Predicting oligura in critically ill patients	3&4	None	Internal dataset	N/A	1.000-10.000	N/A	N/A	Unclear	Unclear	High	Low	High
**1149**	Yamga	2023	United States	Discriminative AI	Predicting mortality	Predicting mortality in patients with cardiogenic shock in the cardiac intensive care unit	5	None	eICU-CRD	MIMIC III	1.000-10.000	1.000 - 10.000	N/A	Low	Low	Low	High	High
**1150**	Yan	2019	China	Discriminative AI	Predicting mortality	Predicting mortality	3&4	None	Internal dataset	N/A	1.000-10.000	N/A	N/A	Low	Unclear	Low	Low	Unclear
**1151**	Yan	2021	United States	Discriminative AI	Predicting an event	Predicting brain function status of intensive care unit patients	5	None	Other type of dataset	Other type of dataset	1.000-10.000	100-1.000	N/A	Low	Unclear	Low	Unclear	Unclear
**1152**	Yan	2024	China	Discriminative AI	Predicting mortality	Predicting 28-day all-cause mortality of sepsis patients in the intensive care unit	3&4	None	MIMIC IV	N/A	1.000-10.000	N/A	N/A	Unclear	Low	Low	High	High
**1153**	Yang	2020	China	Discriminative AI	Predicting complications	Early detection of sepsis	3&4	None	Other type of dataset	N/A	10.000-100.000	N/A	N/A	Low	Low	Low	High	High
**1154**	Yang	2021	China	Discriminative AI	Predicting complications	Predicting traumatic brain injury-induced coagulopathy	5	None	MIMIC IV	eICU-CRD	100-1000	100-1.000	N/A	Low	Low	High	Unclear	High
**1155**	Yang	2021	China	Discriminative AI	Predicting mortality	Predicting survival of coronary care unit patients	3&4	None	MIMIC III	N/A	1.000-10.000	N/A	N/A	Low	Low	Low	High	High
**1156**	Yang	2021	South Korea	Discriminative AI	Predicting complications	To estimate and validate arterial blood pressure (ABP) using non-invasive biosignals, specifically ECG and photoplethysmogram (PPG), from a large dataset, comparing different AI models	5	None	Other type of dataset	MIMIC III	1.000-10.000	100-1.000	N/A	Low	Low	Low	Low	Low
**1157**	Yang	2021	China	Discriminative AI	Predicting mortality	Predicting mortality in intensive care unit patients	3&4	None	MIMIC III	N/A	No information	N/A	N/A	Low	Unclear	Low	Unclear	Unclear
**1158**	Yang	2022	China	Discriminative AI	Predicting mortality	Predicting mortality in patients with heart failure with diabetes at the intensive care unit	5	None	MIMIC IV	eICU-CRD	1.000-10.000	1.000 - 10.000	N/A	Low	Unclear	Unclear	High	High

© 2025 Berkhout WEM et al. *JAMA Network Open.*

**1159**	Yang	2022	China	Discriminative AI	Predicting complications	Prediction of disseminated intravascular coagulation	3&4	None	Internal dataset	N/A	1.000-10.000	N/A	N/A	Low	Low	Low	Low	Low
**1160**	Yang	2022	China	Discriminative AI	Predicting mortality	Predicting mortality in intensive care unit patients	3&4	None	MIMIC III	N/A	1.000-10.000	N/A	N/A	Low	Low	Low	Unclear	Unclear
**1161**	Yang	2022	Korea	Discriminative AI	Predicting mortality	Predicting short-term mortality in intensive care unit	5	None	Internal dataset	Internal dataset	1.000-10.000	1.000 - 10.000	N/A	Low	Unclear	Low	Unclear	Unclear
**1162**	Yang	2022	Taiwan	Discriminative AI	Classifying sub-populations	Predicting cluster membership of patients with prolonged mechanical ventilation using trajectories of rapid shallow breathing index (RSBI) based on baseline clinical variables.	3&4	TRIPOD/ TRIPOD+AI	Internal dataset	N/A	1.000-10.000	N/A	N/A	Low	Low	High	Unclear	High
**1163**	Yang	2023	China	Discriminative AI	Predicting mortality	Predicting 90-day mortality for intensive care trauma patients	3&4	TRIPOD/ TRIPOD+AI	MIMIC III	N/A	1.000-10.000	N/A	N/A	Low	Unclear	Low	High	High
**1164**	Yang	2023	United Kingdom	Discriminative AI (Reinforcement learning)	Predicting prognosis	Predicting patient discharge status	3&4	None	eICU-CRD	N/A	10.000-100.000	N/A	N/A	Low	Low	Low	High	High
**1165**	Yang	2023	China	Discriminative AI	Predicting mortality	Predicting mortality for patients with sepsis-associated acute kidney injury	3&4	None	MIMIC IV	N/A	1.000-10.000	N/A	N/A	Unclear	Unclear	Low	Unclear	Unclear
**1166**	Yang	2023	China	Discriminative AI	Predicting complications	Predict high-flow nasal cannula outcomes	3&4	None	Internal dataset	N/A	0-100	N/A	N/A	Low	Low	Low	Unclear	Unclear
**1167**	Yang	2024	China	Discriminative AI	Predicting complications	Predicting traumatic brain injury induced coagulopathy	5	RECORD	MIMIC IV	eICU-CRD	100-1000	100-1.000	N/A	Low	Unclear	High	Unclear	Unclear
**1168**	Yang	2024	China	Discriminative AI	Predicting complications	Predicting acute kidney injury in critically ill patients	5	None	MIMIC IV	Internal dataset, eICU-CRD	10.000-100.000	1.000 - 10.000	N/A	N/A	N/A	N/A	N/A	N/A
**1169**	Yang	2024	China	Discriminative AI	Predicting mortality	Predicting sepsis in-hospital mortality risk in intensive care unit	5	JMIR Guidelines for Developing and Reporting Machine Learning Predictive Models in Biomedical Research	MIMIC IV	eICU-CRD	10.000-100.000	10.000 - 100.000	N/A	Low	Low	Low	Low	Low
**1170**	Yao	2020	China	Discriminative AI	Predicting mortality	Predicting hospital mortality in patients with postoperative sepsis	3&4	TRIPOD/ TRIPOD+AI	MIMIC III	N/A	1.000-10.000	N/A	N/A	Low	Unclear	Low	High	High
**1171**	Ye	2022	United States	Discriminative AI	Predicting complications	Predicting early intracranial pressure evaluation for traumatic brain injury patients	3&4	None	Other type of dataset	N/A	No information	N/A	N/A	Low	Unclear	Low	High	High
**1172**	Ye	2022	United States	Discriminative AI	Predicting mortality	Predicting mortality in critically ill patients with diabetes	3&4	None	MIMIC III	N/A	1.000-10.000	N/A	N/A	Unclear	Low	High	Unclear	High
**1173**	Ye	2023	China	Discriminative AI	Predicting mortality	Predicting in-hospital mortality in chronic kidney disease patients with coronary artery disease	5	None	MIMIC IV	eICU-CRD	1.000-10.000	1.000 - 10.000	N/A	Low	Low	High	Unclear	Unclear
**1174**	Ye	2024	United States	Discriminative AI	Assessing videos and images	Distinguishing patients with ARDS or pneumonia from normal lungs	5	None	Internal dataset	Internal dataset	10.000-100.000	1.000 - 10.000	N/A	Low	Unclear	Low	High	High
**1175**	Yeh	2019	United States	Discriminative AI	Predicting complications	Predicting hyperchloremia	3&4	TRIPOD/ TRIPOD+AI	MIMIC III	N/A	10.000-100.000	N/A	N/A	Low	Unclear	Unclear	Low	Unclear
**1176**	Yeh	2020	United States	Discriminative AI	Predicting complications	Predicting hyperchloremia in intensive care unit patients	3&4	None	MIMIC III	N/A	10.000-100.000	N/A	N/A	Low	Unclear	High	Low	High
**1177**	Yen	2022	Taiwan	Discriminative AI	Determining physiological values	Parameter estimation model for photoplethysmography	3&4	None	MIMIC II	N/A	10.000-100.000	N/A	N/A	Unclear	Low	Low	Unclear	Unclear

© 2025 Berkhout WEM et al. *JAMA Network Open.*

**1178**	Yijing	2022	China	Discriminative AI	Predicting complications; Predicting an event	Predicting cardiac arrest in critically ill patients	3&4	None	MIMIC III	N/A	1.000-10.000	N/A	N/A	Low	Low	High	Low	High
**1179**	Yildirim	2024	Turkey	Discriminative AI	Predicting mortality	Predicting mortality for COVID-19 patients admitted to the intensive care unit	5	None	Internal dataset	Internal dataset	100-1000	100-1.000	N/A	Low	Unclear	Low	High	High
**1180**	Yimer	2023	Ethiopia	Discriminative AI	Classification of signals	Providing daily changes and results	3&4	None	MIMIC III	N/A	0-100	N/A	N/A	N/A	N/A	N/A	N/A	N/A
**1181**	Yin	2023	United States	Discriminative AI	Predicting mortality	Predicting mortality	3&4	None	eICU-CRD	N/A	10.000-100.000	N/A	N/A	Unclear	Unclear	Unclear	Unclear	Unclear
**1182**	Yong	2024	China	Discriminative AI	Predicting mortality	Predicting mortality for sepsis patients	3&4	None	MIMIC III	N/A	1.000-10.000	N/A	N/A	Low	Low	Low	High	High
**1183**	Yoon	2020	United States	Discriminative AI	Predicting complications	Predicting hypotension event among intensive care unit patients	3&4	TRIPOD/ TRIPOD+AI	MIMIC III	N/A	1.000-10.000	N/A	N/A	N/A	N/A	N/A	N/A	N/A
**1184**	Yoon	2024	Korea	Generative AI (Large language model)	Assessing clinical notes	Translating diagnosis codes to natural language text and extracting medication list from discharge summary	5	None	N/A	MIMIC III	N/A	10.000 - 100.000	N/A	Low	Unclear	Unclear	Unclear	Unclear
**1185**	Young	2021	Australia	Discriminative AI	Assessing clinical notes	Assessing words suggestive of behavioural disturbance in ICU progress notes	3&4	None	Internal dataset	N/A	1.000-10.000	N/A	N/A	N/A	N/A	N/A	N/A	N/A
**1186**	Young	2022	Australia	Discriminative AI	Predicting complications	Identify behaviour disturbance	3&4	None	Internal dataset	N/A	1.000-10.000	N/A	N/A	Low	Low	High	Unclear	High
**1187**	Young	2023	Australia	Discriminative AI	Assessing clinical notes	Screening for behavioural disturbance phenotypes	3&4	None	Internal dataset	N/A	1.000-10.000	N/A	N/A	Low	Low	High	Unclear	High
**1188**	Yu	2020	United States	Discriminative AI	Determining physiological values	Predicting laboratory test results	3&4	None	MIMIC III	N/A	10.000-100.000	N/A	N/A	N/A	N/A	N/A	N/A	N/A
**1189**	Yu	2021	China	Discriminative AI	Alarm reduction	Reducing false arrhythmia alarms in the intensive care unit	3&4	None	Other type of dataset	N/A	1.000-10.000	N/A	N/A	N/A	N/A	N/A	N/A	N/A
**1190**	Yu	2021	China	Discriminative AI	Predicting mortality	Predicting mortality of patients with craniotomy in the intensive care unit	3&4	None	Internal dataset	N/A	100-1000	N/A	N/A	N/A	N/A	N/A	N/A	N/A
**1191**	Yu	2022	China	Discriminative AI	Predicting mortality	Predicting long-term mortality in patients post-cardiac surgery	3&4	TRIPOD/ TRIPOD+AI	MIMIC III	N/A	1.000-10.000	N/A	N/A	Low	Low	High	High	High
**1192**	Yu	2022	China	Discriminative AI	Predicting mortality	Predicting mortality for ICU patients	3&4	None	Other type of dataset	N/A	1.000-10.000	N/A	N/A	Low	Low	High	Unclear	High
**1193**	Yu	2023	China	Discriminative AI	Diagnostic	Optimizing the diagnosis of pulmonary embolism in patients with acute exacerbation of chronic obstructive pulmonary disease	3&4	None	Internal dataset	N/A	100-1000	N/A	N/A	Low	Low	Unclear	Unclear	Unclear
**1194**	Yu	2023	China	Discriminative AI	Classifying sub-populations	Identifying cardiogenic shock phenotypes	3&4	None	MIMIC IV	N/A	100-1000	N/A	N/A	Low	Unclear	Low	High	High
**1195**	Yu	2023	China	Discriminative AI (Reinforcement learning)	Treatment recommendation	Evaluate the goodness of treatments during the learning process and the effectiveness of the final learned treatment policies	3&4	None	MIMIC III	N/A	10.000-100.000	N/A	N/A	Low	Low	High	Unclear	High
**1196**	Yu	2024	Norway	Discriminative AI	Assessing videos and images	Measuring mitral annular plane systolic excursion (MAPSE)	5	None	N/A	Other type of dataset	N/A	0-100	N/A	Low	Unclear	Low	High	High
**1197**	Yu	2024	China	Discriminative AI	Predicting mortality	Predicting mortality and complexity	3&4	None	Internal dataset	N/A	100-1000	N/A	N/A	Low	Low	Low	Unclear	Unclear
**1198**	Yuan	2021	Taiwan	Discriminative AI	Assessing videos and images	Assessing the placement of endotracheal tubes on Chest X-ray in the intensive care unit	3&4	None	Internal dataset	N/A	1.000-10.000	N/A	N/A	Unclear	Low	Low	Unclear	Unclear

© 2025 Berkhout WEM et al. *JAMA Network Open.*

**1199**	Yue	2022	China	Discriminative AI	Predicting complications	Predicting acute kidney injury in critically ill patients with sepsis	3&4	None	MIMIC III	N/A	1.000-10.000	N/A	N/A	Low	Unclear	High	Unclear	High
**1200**	Yun	2021	United States	Discriminative AI	Predicting mortality	Predicting in-hospital death of patients in the surgical intensive care unit	3&4	None	Internal dataset	N/A	1.000-10.000	N/A	N/A	Low	Low	High	Unclear	High
**1201**	Yun	2023	Korea	Discriminative AI	Assessing videos and images	Identifying change in pairs of chest radiographs during longitudinal follow-up	5	None	Internal dataset	Internal dataset	100.000+	100-1.000	N/A	Low	Unclear	Low	High	High
**1202**	Zabihi	2022	Canada	Discriminative AI	Determining physiological values	Cuff-less and non-invasive blood pressure estimation	3&4	None	No information	N/A	100.000+	N/A	N/A	Unclear	Low	Low	Low	Unclear
**1203**	Zahr	2024	United States	Discriminative AI	Predicting complications	Predicting acute kidney injury in patients with sickle cell disease	3&4	None	Internal dataset	N/A	1.000-10.000	N/A	N/A	Low	Low	Low	High	High
**1204**	Zappalà	2024	Italy	Discriminative AI	Predicting complications	Predicting acute kidney injury stage 3 in intensive care unit	5	None	MIMIC III, AmsterdamUMCdb	Other type of dataset, eICU-CRD	1.000-10.000	1.000 - 10.000	N/A	Low	Low	High	Unclear	High
**1205**	Zardkoohi	2022	Iran	Discriminative AI	Determining physiological values	Predicting blood pressure time series	3&4	None	Other type of dataset	N/A	0-100	N/A	N/A	N/A	N/A	N/A	N/A	N/A
**1206**	Zarrin	2024	United States	Discriminative AI	Predicting complications	Predicting cerebral vasospasm in subarachnoid hemorrhage patients	5	None	Internal dataset	Internal dataset	1.000-10.000	1.000 - 10.000	N/A	Unclear	Low	Low	High	High
**1207**	Zeng	2021	China	Discriminative AI	Predicting mortality	Predicting mortality for sepsis patients	5	TRIPOD/ TRIPOD+AI	eICU-CRD	eICU-CRD	10.000-100.000	10.000 - 100.000	N/A	Low	Unclear	Low	Unclear	Unclear
**1208**	Zeng	2022	China	Discriminative AI	Improving mechanical ventilation	Predicting extubation failure in invasive mechanical ventilation	3&4	None	MIMIC IV	N/A	1.000-10.000	N/A	N/A	Low	Low	Low	Low	Low
**1209**	Zeng	2023	China	Discriminative AI	Predicting mortality	Predicting mortality for ICU patients	5	None	MIMIC IV	eICU-CRD	10.000-100.000	100.000+	N/A	Unclear	Low	Low	Unclear	Unclear
**1210**	Zeng	2023	China	Discriminative AI	Predicting mortality	Predicting mortality in intensive care unit patients	3&4	None	MIMIC III	N/A	100.000+	N/A	N/A	Low	Low	Low	Low	Low
**1211**	Zeng	2024	Iran	Discriminative AI	Predicting mortality	Predicting mortality for acute tubular necrosis	3&4	None	MIMIC IV	N/A	1.000-10.000	N/A	N/A	Low	Low	Low	Unclear	Unclear
**1212**	Zhai	2022	China	Discriminative AI	Assessing videos and images	Calculating left ventricular outflow tract velocity time intregral from point of care ultrasound for critically ill patients	8	None	N/A	N/A	N/A	N/A	46	N/A	N/A	N/A	N/A	N/A
**1213**	Zhang	2020	China	Discriminative AI	Classifying sub-populations	Classify sepsis into different immune endotypes	5	None	Other type of dataset	Other type of dataset	100-1000	100-1.000	N/A	Low	Low	Low	Low	Low
**1214**	Zhang	2020	United States	Discriminative AI	Predicting mortality; Predicting readmissions; Predicting length of stay	Predicting in-hospital mortality, 30-day readmission and long length of stay	3&4	None	MIMIC III	N/A	10.000-100.000	N/A	N/A	Unclear	Low	Low	High	High
**1215**	Zhang	2021	China	Discriminative AI	Predicting complications	Predicting acute hypotensive episodes	3&4	None	MIMIC III	N/A	1.000-10.000	N/A	N/A	Low	Low	Low	Unclear	Unclear
**1216**	Zhang	2021	China	Discriminative AI	Predicting complications	Prediction of agitation in mechanically ventilated patients	5	None	Internal dataset	Internal dataset	1.000-10.000	100-1.000	N/A	Low	Low	Low	High	High
**1217**	Zhang	2022	United States	Discriminative AI (Reinforcement learning)	Treatment recommendation	Recommendations for managing hypotension in the intensive care unit	3&4	None	MIMIC III	N/A	10.000-100.000	N/A	N/A	Low	Low	Low	Unclear	Unclear
**1218**	Zhang	2022	United Kingdom	Generative AI (Large language model)	Predicting mortality; Predicting complications; Predicting length of stay	Predicting length of stay, in-hospital mortality and physiological decompensation	3&4	None	MIMIC III	N/A	10.000-100.000	N/A	N/A	Unclear	Unclear	Low	Unclear	Unclear
**1219**	Zhang	2022	China	Discriminative AI	Predicting complications	Prediction of sepsis-associated acute kidney injury	5	None	MIMIC IV	Internal dataset, eICU-CRD	10.000-100.000	10.000 - 100.000	N/A	Low	High	High	High	High
**1220**	Zhang	2022	China	Discriminative AI	Predicting complications	Predicting acute kidney injury in critical care patients with acute cerebrovascular disease	5	None	MIMIC III	Internal dataset	1.000-10.000	100-1.000	N/A	Unclear	Low	Low	Low	Unclear

© 2025 Berkhout WEM et al. *JAMA Network Open.*

**1221**	Zhang	2022	China	Discriminative AI	Predicting complications	Predicting postoperative shock in elderly patients	5	None	MIMIC IV	MIMIC III	1.000-10.000	100-1.000	N/A	N/A	N/A	N/A	N/A	N/A
**1222**	Zhang	2022	China	Discriminative AI	Predicting complications	Prediction of acute kidney injury after cardiac surgery	3&4	TRIPOD/ TRIPOD+AI	Internal dataset	N/A	1.000-10.000	N/A	N/A	Low	Low	Low	Low	Low
**1223**	Zhang	2022	China	Discriminative AI	Predicting mortality	Predicting 30-day mortality risk	3&4	None	MIMIC IV	N/A	1.000-10.000	N/A	N/A	Low	Low	Low	Low	Low
**1224**	Zhang	2023	China	Discriminative AI (Reinforcement learning)	Treatment recommendation	Sepsis treatment recommendations	3&4	None	MIMIC III	N/A	10.000-100.000	N/A	N/A	Low	Low	Low	Low	Low
**1225**	Zhang	2023	China	Discriminative AI	Predicting complications	Predicting sepsis-associated delirium in septic patients in the intensive care unit	5	None	MIMIC IV	eICU-CRD	10.000-100.000	1.000 - 10.000	N/A	Unclear	Low	Low	Unclear	Unclear
**1226**	Zhang	2023	China	Discriminative AI	Predicting complications	Predicting sepsis	5	None	MIMIC III	Internal dataset	100.000+	100-1.000	N/A	Low	Low	Low	High	High
**1227**	Zhang	2023	China	Discriminative AI	Predicting prognosis	Predicting in-hospital prognosis after traumatic brain injury	3&4	None	Internal dataset	N/A	100-1000	N/A	N/A	N/A	N/A	N/A	N/A	N/A
**1228**	Zhang	2023	China	Discriminative AI	Predicting complications	Predict blood stream infection	5	None	MIMIC III	Internal dataset	1.000-10.000	100-1.000	N/A	Low	Low	Low	High	High
**1229**	Zhang	2023	China	Discriminative AI	Predicting mortality	Predicting mortality for patients with hepatic encephalopathy	3&4	None	MIMIC IV	N/A	100-1000	N/A	N/A	N/A	N/A	N/A	N/A	N/A
**1230**	Zhang	2024	China	Discriminative AI (Reinforcement learning)	Provide dosage recommendations	Controlling drug dosage in sepsis treatment	3&4	None	MIMIC IV	N/A	1.000-10.000	N/A	N/A	N/A	N/A	N/A	N/A	N/A
**1231**	Zhang	2024	China	Discriminative AI	Predicting complications	Predicting delirium in sepsis patients in the intensive care unit	3&4	None	Internal dataset	N/A	100-1000	N/A	N/A	Low	Low	Low	Low	Low
**1232**	Zhang	2024	United States	Discriminative AI	Predicting length of stay	Predicting length of stay of 2nd admission to ICU	3&4	None	MIMIC IV	N/A	10.000-100.000	N/A	N/A	Low	Low	Low	High	High
**1233**	Zhang	2024	China	Discriminative AI	Classifying sub-populations	Classifying intensive care patients with blood cultures	5	None	MIMIC IV	MIMIC III	1.000-10.000	1.000 - 10.000	N/A	N/A	N/A	N/A	N/A	N/A
**1234**	Zhang	2024	China	Discriminative AI (Reinforcement learning)	Treatment recommendation	Guiding sepsis medication treatment	3&4	None	MIMIC III	N/A	10.000-100.000	N/A	N/A	High	Unclear	Low	High	High
**1235**	Zhang	2024	China	Discriminative AI	Predicting mortality	Predicting sepsis in-hospital mortality	3&4	None	Internal dataset, MIMIC IV, eICU-CRD	N/A	1.000-10.000	N/A	N/A	Low	Unclear	Unclear	Unclear	Unclear
**1236**	Zhao	2021	China	Discriminative AI	Predicting complications	Predicting the risk of sepsis-induced coagulopathy	5	TRIPOD/ TRIPOD+AI	MIMIC IV	eICU-CRD	10.000-100.000	10.000 - 100.000	N/A	High	Low	High	Unclear	High
**1237**	Zhao	2021	China	Discriminative AI	Predicting complications	Predicting of extubation failure	5	TRIPOD/ TRIPOD+AI	MIMIC IV	Internal dataset	10.000-100.000	100-1.000	N/A	Low	Unclear	Low	High	High
**1238**	Zhao	2022	China	Discriminative AI	Predicting mortality; Classifying sub-populations	Provide individualized care for patients with Low Cardiac Ouput Syndrome	3&4	None	Internal dataset	N/A	1.000-10.000	N/A	N/A	N/A	N/A	N/A	N/A	N/A
**1239**	Zhao	2022	China	Discriminative AI	Predicting health improvement	Predicting recovery of acute kidney injury	3&4	TRIPOD/ TRIPOD+AI	MIMIC IV	N/A	10.000-100.000	N/A	N/A	Unclear	Unclear	Low	Unclear	Unclear
**1240**	Zhao	2022	China	Discriminative AI	Predicting complications	Predicting circulatory failure in intensive care unit patients	3&4	None	Other type of dataset	N/A	10.000-100.000	N/A	N/A	N/A	N/A	N/A	N/A	N/A
**1241**	Zhao	2023	United States	Discriminative AI	Diagnostic	Providing a comorbidity network for improvement of diagnosing intensive care unit patients	3&4	None	MIMIC III	N/A	10.000-100.000	N/A	N/A	Low	Low	High	Low	High
**1242**	Zhao	2023	United States	Discriminative AI	Classifying sub-populations	Identifying cytokine signature clusters in patients with septic shock	3&4	None	Internal dataset	N/A	100-1000	N/A	N/A	N/A	N/A	N/A	N/A	N/A
**1243**	Zhao	2023	China	Discriminative AI	Predicting mortality	Predicting mortality for intensive care unit patients	3&4	None	Other type of dataset	N/A	1.000-10.000	N/A	N/A	Low	Low	Low	Low	Low
**1244**	Zhao	2023	China	Discriminative AI	Predicting complications	Predict upcoming urinary tract infection after intracerebral hemorrhage	3&4	None	Internal dataset	N/A	100-1000	N/A	N/A	Low	Low	Low	High	High
**1245**	Zhao	2024	China	Discriminative AI	Predicting mortality	Predicting ICU-acquired fungal infections	3&4	None	Internal dataset	N/A	1.000-10.000	N/A	N/A	Low	Unclear	Low	Unclear	Unclear

© 2025 Berkhout WEM et al. *JAMA Network Open.*

**1246**	Zheng	2021	United States	Discriminative AI (Reinforcement learning)	Improving mechanical ventilation	Identifying the optimal personalized oxygen flow rate	3&4	None	Internal dataset	N/A	1.000-10.000	N/A	N/A	Low	Low	Low	Low	Low
**1247**	Zheng	2023	China	Discriminative AI	Predicting complications	Predicting acute kidney injury in critically ill cirrhotic patients	5	TRIPOD/ TRIPOD+AI	eICU-CRD	MIMIC	1.000-10.000	100-1.000	N/A	Low	Low	High	High	High
**1248**	Zheng	2023	China	Discriminative AI	Predicting mortality	Predicting mortality for septic shock patients	5	None	MIMIC IV	eICU-CRD	10.000-100.000	1.000 - 10.000	N/A	N/A	N/A	N/A	N/A	N/A
**1249**	Zhi	2021	China	Discriminative AI	Predicting mortality	Predicting mortality for sepsis patients	5	None	MIMIC III	Internal dataset	1.000-10.000	100-1.000	N/A	Low	Low	Low	Unclear	Unclear
**1250**	Zhong	2021	China	Discriminative AI	Predicting mortality; Predicting complications	Predicting 30-days mortality, septic shock, thrombocytopenia and liver dysfunction after open-heart surgery	3&4	None	MIMIC III	N/A	1.000-10.000	N/A	N/A	Low	Low	Low	High	High
**1251**	Zhou	2022	China	Discriminative AI	Alarm reduction	False arrhythia alarm reduction in ICU	3&4	None	Other type of dataset	N/A	100-1000	N/A	N/A	Low	Low	Low	Low	Low
**1252**	Zhou	2022	China	Discriminative AI	Predicting complications	Predicting of acute kidney injury among patients with sepsis-associated acute respiratory distress syndrome	3&4	None	MIMIC III	N/A	1.000-10.000	N/A	N/A	Low	Low	High	Low	High
**1253**	Zhou	2022	Spain	Discriminative AI	Predicting mortality	Predicting the risk of death of patients with COVID-19 admitted to the ICU	3&4	None	Internal dataset	N/A	100-1000	N/A	N/A	Low	Low	High	High	High
**1254**	Zhou	2022	China	Discriminative AI	Determining physiological values	Predicting continuous blood pressure in intensive care unit patients	3&4	None	MIMIC II	N/A	10.000-100.000	N/A	N/A	N/A	N/A	N/A	N/A	N/A
**1255**	Zhou	2022	China	Discriminative AI	Predicting prognosis	Predicting prognosis of myocardial infarct patients	3&4	None	MIMIC III	N/A	1.000-10.000	N/A	N/A	Low	Low	Low	High	High
**1256**	Zhou	2023	China	Discriminative AI	Predicting complications	Predicting ARDS in patients with COVID-19	3&4	None	Internal dataset	N/A	100-1000	N/A	N/A	High	Low	High	High	High
**1257**	Zhou	2023	China	Discriminative AI	Predicting mortality	Predicting mortality in sepsis-associated acute kidney injury patients	5	TRIPOD/ TRIPOD+AI	MIMIC IV	Internal dataset	10.000-100.000	100-1.000	N/A	Unclear	Low	Low	Unclear	Unclear
**1258**	Zhou	2024	China	Discriminative AI	Predicting mortality	Predicting the risk of 28-day death in patients with sepsis-induced coagulopathy	5	None	MIMIC III	eICU-CRD, MIMIC-IV	1.000-10.000	1.000 - 10.000	N/A	Low	High	High	Unclear	High
**1259**	Zhu	2018	China	Discriminative AI	Predicting mortality	Predicting mortality in intensive care unit patients	3&4	None	Other type of dataset	N/A	1.000-10.000	N/A	N/A	Low	Low	Unclear	High	High
**1260**	Zhu	2021	China	Discriminative AI (Reinforcement learning)	Treatment recommendation	Treatment recommendation for sepsis patients	3&4	None	MIMIC III	N/A	10.000-100.000	N/A	N/A	N/A	N/A	N/A	N/A	N/A
**1261**	Zhu	2021	China	Discriminative AI	Predicting mortality	Predicting hospital mortality in mechanically ventilated ICU patients	3&4	TRIPOD/ TRIPOD+AI	MIMIC III	N/A	10.000-100.000	N/A	N/A	Low	Low	Low	High	High
**1262**	Zhu	2024	China	Discriminative AI	Predicting prognosis	Predicting traumatic brain injury severity and prognosis	3&4	TRIPOD/ TRIPOD+AI	Internal dataset	N/A	0-100	N/A	N/A	Unclear	Low	Low	Low	Unclear
**1263**	Zhuang	2023	China	Discriminative AI	Predicting mortality	Predicting mortality for ICU patients with sepsis	5	None	MIMIC III	Other type of dataset, MIMIC-IV, eICU-CRD	10.000-100.000	10.000 - 100.000	N/A	Unclear	Low	Unclear	High	High
**1264**	Zou	2023	China	Discriminative AI	Predicting mortality	Predicting mortality	3&4	None	eICU-CRD	N/A	10.000-100.000	N/A	N/A	Low	Low	Low	Unclear	Unclear
**1265**	Zou	2023	China	Discriminative AI	Predicting length of stay	Predicting length of stay range	3&4	None	MIMIC III	N/A	10.000-100.000	N/A	N/A	Unclear	Unclear	Low	High	High
**1266**	Zou	2023	China	Generative AI (Large language model)	Predicting mortality	Predicting the mortality of patients on mechanical ventilation	3&4	None	MIMIC III	N/A	10.000-100.000	N/A	N/A	Low	Low	Low	Unclear	Unclear
**1267**	Zwerwer	2024	Netherlands	Discriminative AI	Predicting need for resource	Predicting the need for an infection-related consultation	3&4	TRIPOD/ TRIPOD+AI	Internal dataset	N/A	1.000-10.000	N/A	N/A	Low	Low	High	High	High

AI = artificial intelligence, ICU = intensive care unit, LLM = large language model, TRIPOD = Transparent Reporting of a multivariable prediction model for Individual Prognosis Or Diagnosis, STROBE = STrengthening the Reporting of OBservational studies in Epidemiology, STARD = Standards for Reporting Diagnostic accuracy studies, RECORD = REporting of studies Conducted

© 2025 Berkhout WEM et al. *JAMA Network Open.*

using Observational Routinely-collected health Data, DECIDE-AI = Developmental and Exploratory Clinical Investigations of DEcision support systems driven by Artificial Intelligence, CONSORT = Consolidated Standards of Reporting Trials, SPIRIT = Standard Protocol Items: Recommendations for Interventional Trials, JMIR = Journal of Medical Internet Research, MINIMAR = MINimum Information for Medical AI Reporting, MDAR = Standards for Reporting Diagnostic Accuracy Studies and the Materials Design Analysis Reporting, MIMIC = Medical Information Mart for Intensive Care, eICU-CRD = eICU Collaborative Research Database.

© 2025 Berkhout WEM et al. *JAMA Network Open.*

eAppendix 6. Results

Figure S1. Over time, the percentage of studies that mention the use of reporting standards

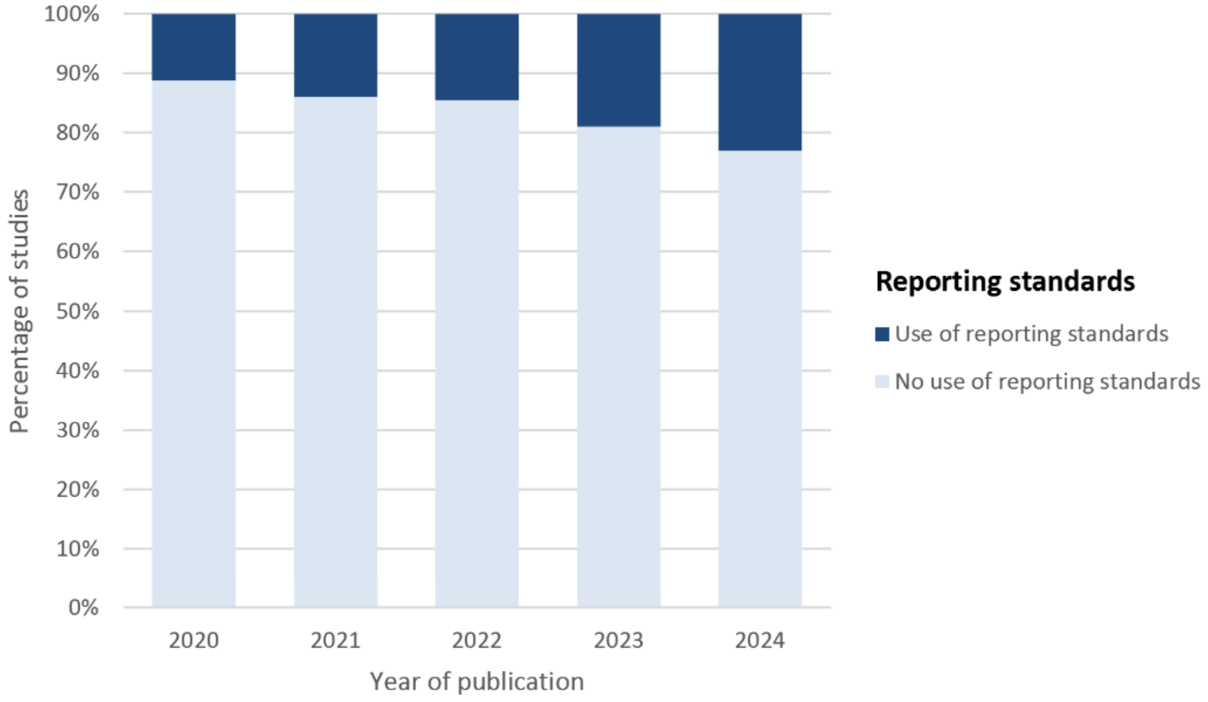

Figure S2. The distribution of the different reporting standards mentioned by the studies (n = 207)

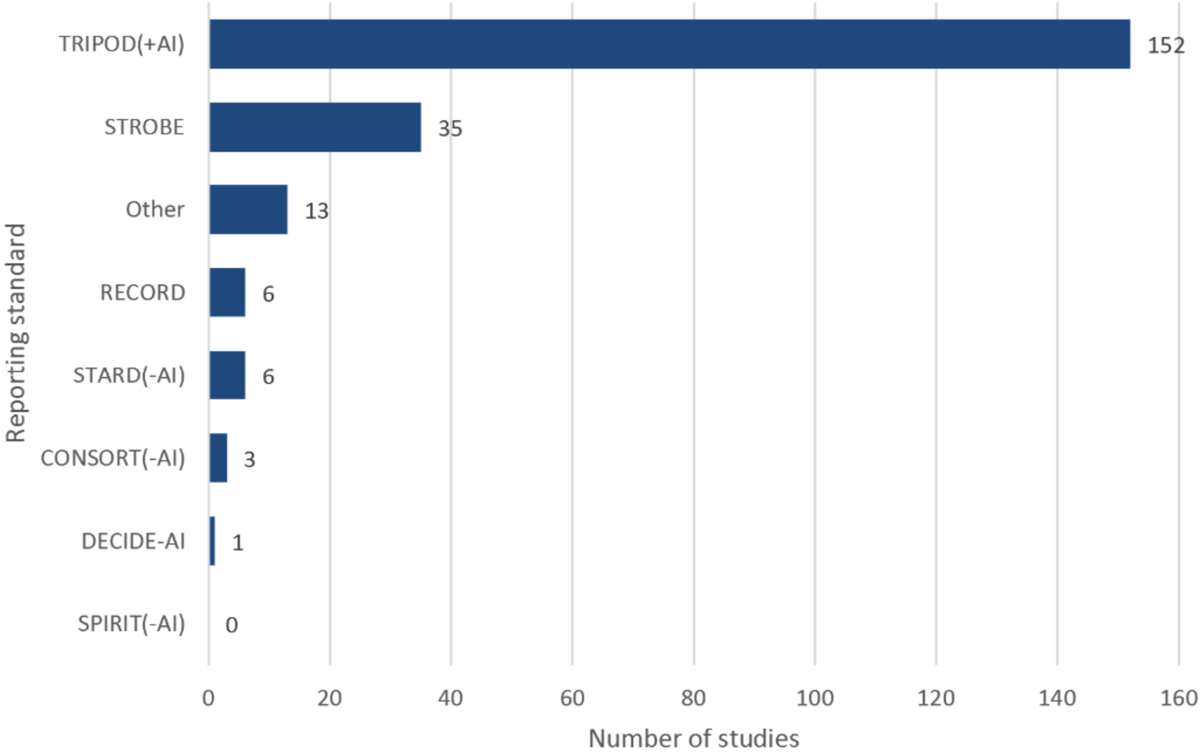

If studies reported the use of several reporting standards, each reporting standard is analyzed individually (n = 216). The ‘Other’ category included the Journal of Medical Internet Research (JMIR) Guidelines for Developing and Reporting Machine Learning Predictive Models in Biomedical Research, the MINimum Information for Medical AI Reporting (MINIMAR) standard, the Standards for Reporting Diagnostic Accuracy Studies and the Materials Design Analysis Reporting (MDAR) framework. AI = Artificial Intelligence, TRIPOD = Transparent Reporting of a multivariable prediction model for Individual Prognosis Or Diagnosis, STROBE = STrengthening the Reporting of OBservational studies in Epidemiology, STARD = Standards for Reporting Diagnostic accuracy studies, RECORD = REporting of studies Conducted using Observational Routinely-collected health Data, DECIDE-AI = Developmental and Exploratory Clinical Investigations of DEcision support systems driven by Artificial Intelligence, CONSORT = Consolidated Standards of Reporting Trials, SPIRIT = Standard Protocol Items: Recommendations for Interventional Trials.

Figure S3. The risk of bias for studies that cited the use of reporting standards (n = 188) compared to the studies that did not cite the use of reporting standards (n = 915)

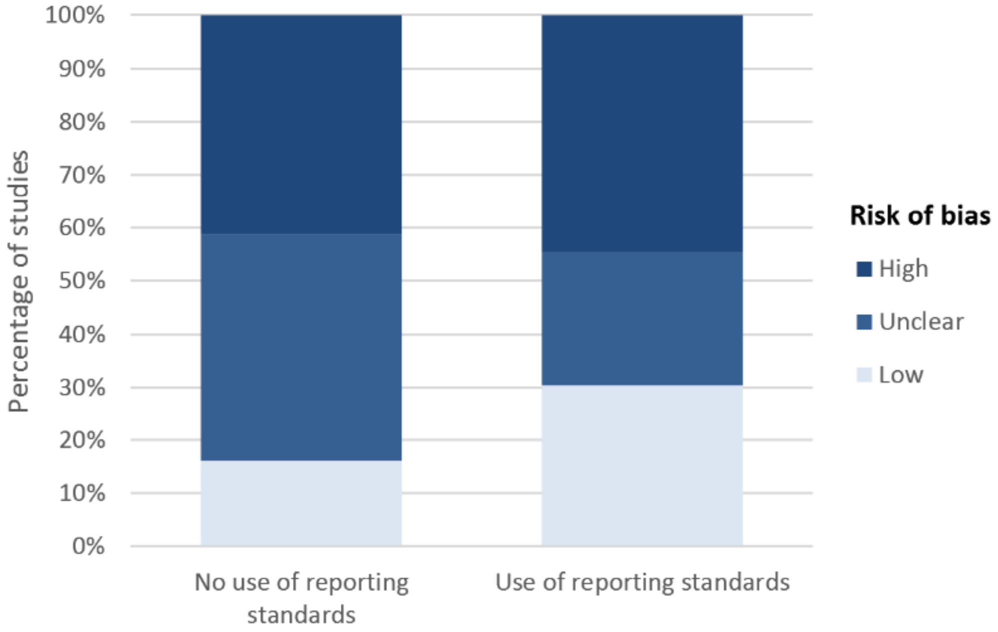

Figure S4. Type of dataset for model development

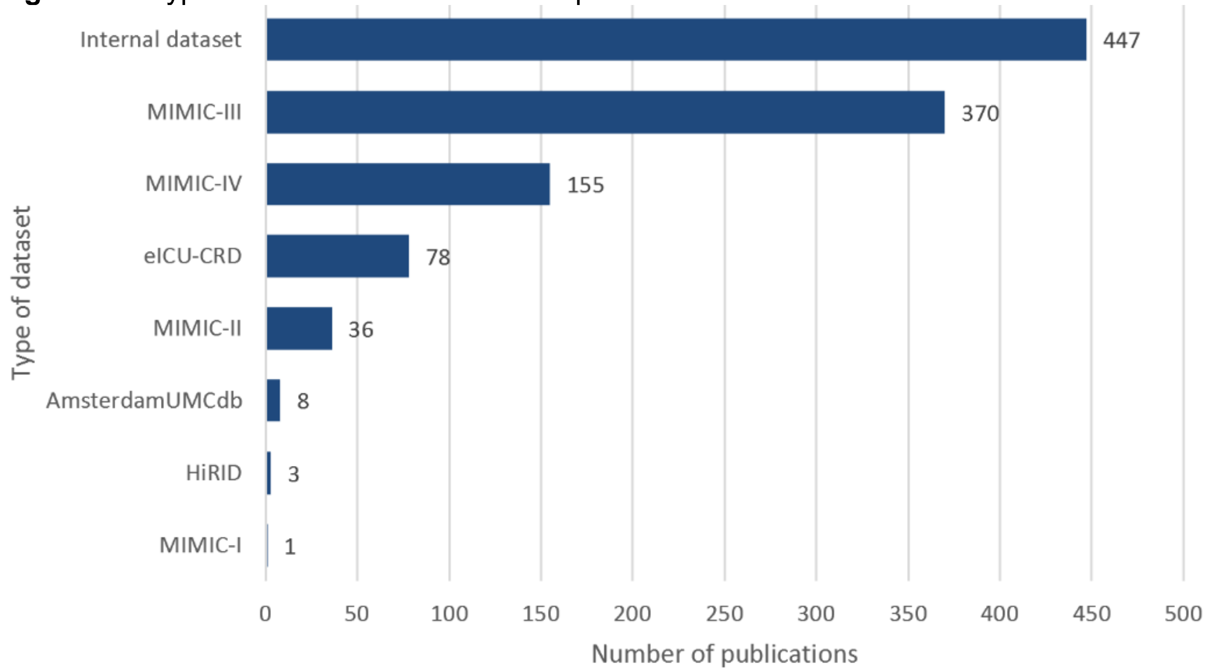

The overall count of studies describing model development is 1,215. Total number can be exceeded as one study can combine multiple datasets into a single dataset. MIMIC = Medical Information Mart for Intensive Care, eICU-CRD = eICU Collaborative Research Database.

Figure S5. Type of dataset for external validation

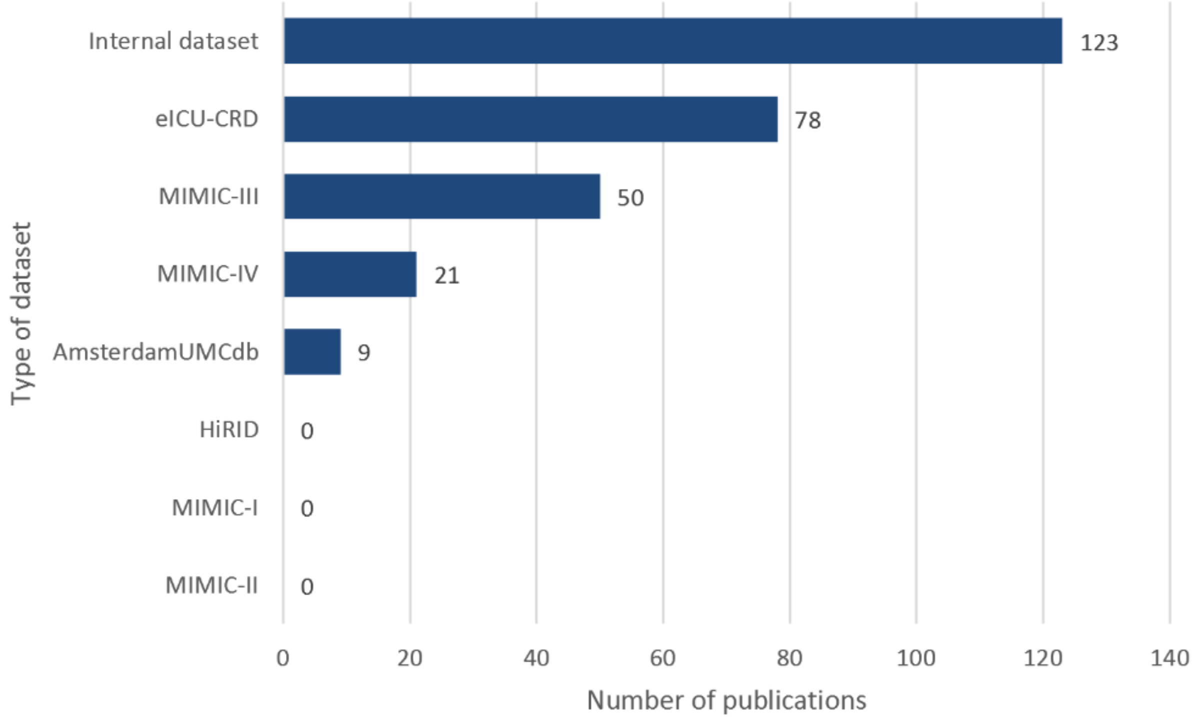

The overall count of studies describing external validation is 304. Total number can be exceeded as one study can combine multiple datasets into a single dataset. MIMIC = Medical Information Mart for Intensive Care, eICU-CRD = eICU Collaborative Research Database.

Figure S6. Number of studies per country for studies with a Technology Readiness Level (TRL) below or equal to 5

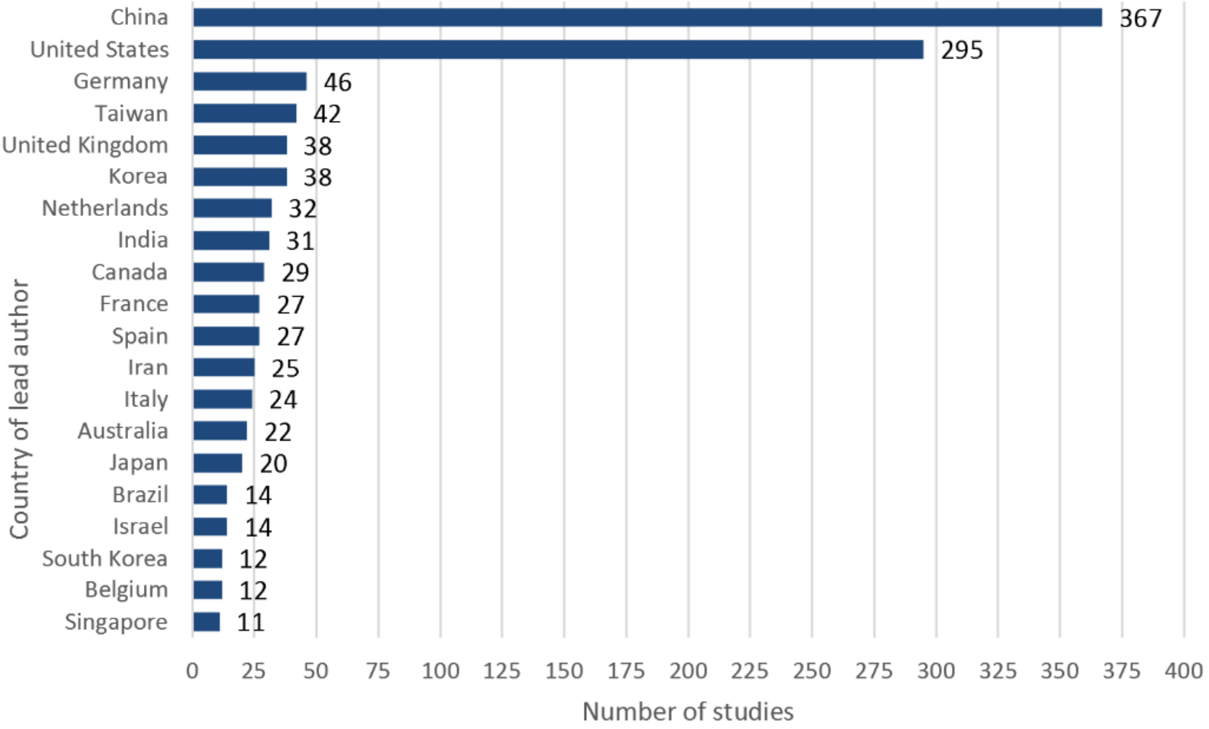

Studies with a TRL below or equal to 5 are focused on the development and validation of AI models. Exclusively the countries with more than 10 studies are included in the graph. Total number of studies with a TRL below or equal to 5 was 1,238.

Figure S7. Number of studies per country for studies with a Technology Readiness Level (TRL) above 5

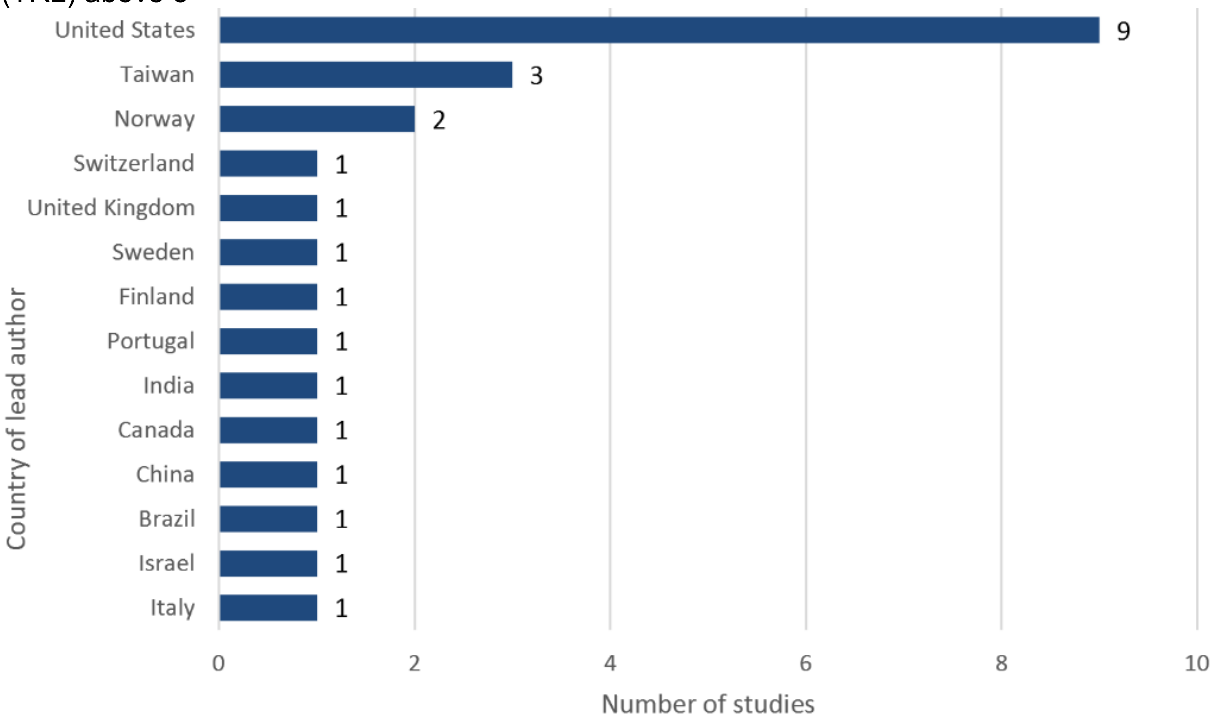

Studies with a TRL above 5 are focused on the integration of AI models into a clinical environment. Total number of studies with a TRL above 5 was 25.

eReferences.

1. van de Sande D, van Genderen ME, Huiskens J, Gommers D, van Bommel J. Moving from bytes to bedside: a systematic review on the use of artificial intelligence in the intensive care unit. *Intensive Care Med*. Jul 2021;47(7):750-760. doi:10.1007/s00134-021-06446-7
2. Sauer CM, Dam TA, Celi LA, et al. Systematic Review and Comparison of Publicly Available ICU Data Sets-A Decision Guide for Clinicians and Data Scientists. *Crit Care Med*. Jun 1 2022;50(6):e581-e588.
3. Schouten JS, Kalden M, van Twist E, et al. From bytes to bedside: a systematic review on the use and readiness of artificial intelligence in the neonatal and pediatric intensive care unit. *Intensive Care Med*. Nov 2024;50(11):1767-1777.
4. de Hond AAH, Shah VB, Kant IMJ, Van Calster B, Steyerberg EW, Hernandez-Boussard T. Perspectives on validation of clinical predictive algorithms. *NPJ Digit Med*. 2023/05/06 2023;6(1):86. doi:10.1038/s41746-023-00832-9
5. Abad ZSH, Lee J. Detecting Uncertainty of Mortality Prediction Using Confident Learning. *Annu Int Conf IEEE Eng Med Biol Soc*. 2021;2021:1719-1722. doi:10.1109/embc46164.2021.9629505
6. Abad ZSH, Maslove DM, Lee J. Predicting Discharge Destination of Critically Ill Patients Using Machine Learning. *IEEE J Biomed Health Inform*. 2021;25(3):827-837. doi:10.1109/jbhi.2020.2995836
7. Abbasi A, Li C, Dekle M, et al. Interpretable machine learning-based predictive modeling of patient outcomes following cardiac surgery. *J Thorac Cardiovasc Surg*. 2023;doi:10.1016/j.jtcvs.2023.11.034
8. Abdullahi T, Singh R, Eickhoff C. Learning to Make Rare and Complex Diagnoses With Generative AI Assistance: Qualitative Study of Popular Large Language Models. *JMIR Med Educ*. 2024;10:e51391. doi:10.2196/51391
9. Abououf M, Singh S, Mizouni R, Otrok H. Explainable AI for Event and Anomaly Detection and Classification in Healthcare Monitoring Systems. *IEEE Internet Things J*. 2024;11(2):3446-3457. doi:10.1109/jiot.2023.3296809
10. Abromavicius V, Plonis D, Tarasevicius D, Serackis A. Two-Stage Monitoring of Patients in Intensive Care Unit for Sepsis Prediction Using Non-Overfitted Machine Learning Models. *Electronics*. 2020;9(7)doi:10.3390/electronics9071133
11. Aden I, Child CHT, Reyes-Aldasoro CC. International Classification of Diseases Prediction from MIMIIC-III Clinical Text Using Pre-Trained ClinicalBERT and NLP Deep Learning Models Achieving State of the Art. *Big Data Cogn Comput*. 2024;8(5)doi:10.3390/bdcc8050047
12. Afshin-Pour B, Qiu M, Hosseini Vajargah S, et al. Discriminating Acute Respiratory Distress Syndrome from other forms of respiratory failure via iterative machine learning. *Intell Based Med*. 2023;7:100087. doi:10.1016/j.ibmed.2023.100087
13. Agmon S, Gillis P, Horvitz E, Radinsky K. Gender-sensitive word embeddings for healthcare. *J Am Med Inform Assoc*. 2022;29(3):415-423. doi:10.1093/jamia/ocab279
14. Ahmad FS, Ali L, Raza Ul M, et al. A hybrid machine learning framework to predict mortality in paralytic ileus patients using electronic health records (EHRs). *J Ambient Intell Humaniz Comput*. 2021;12(3):3283-3293. doi:10.1007/s12652-020-02456-3
15. Ahmed FS, Ali L, Joseph BA, Ikram A, Ul Mustafa R, Bukhari SAC. A statistically rigorous deep neural network approach to predict mortality in trauma patients admitted to the intensive care unit. *J Trauma Acute Care Surg*. 2020;89(4):736-742. doi:10.1097/TA.0000000000002888
16. Aikodon N, Ortega-Martorell S, Olier I. Predicting Decompensation Risk in Intensive Care Unit Patients Using Machine Learning. *Algorithms*. 2024;17(1)doi:10.3390/a17010006
17. Akerlund CAI, Holst A, Stocchetti N, et al. Clustering identifies endotypes of traumatic brain injury in an intensive care cohort: a CENTER-TBI study. *Crit Care*. 2022;26(1):228. doi:10.1186/s13054-022-04079-w
18. Akiki RK, Anand RS, Borrelli M, Sarkar IN, Liu PY, Chen ES. Predicting open wound mortality in the ICU using machine learning. *J Emerg Crit Care Med*. 2021;5:13. doi:10.21037/jeccm-20-154
19. Alabdulhafith M, Saleh H, Elmannai H, et al. A Clinical Decision Support System for Edge/Cloud ICU Readmission Model Based on Particle Swarm Optimization, Ensemble Machine Learning, and Explainable Artificial Intelligence. *IEEE Access*. 2023;11:100604-100621. doi:10.1109/access.2023.3312343
20. Ala-Kokko T, Erikson K, Koskenkari J, Laurila J, Kortelainen J. Monitoring of nighttime EEG slow-wave activity during dexmedetomidine infusion in patients with hyperactive ICU delirium: An observational pilot study. *Acta Anaesthesiol Scand*. 2022;66(10):1211-1218. doi:10.1111/aas.14131
21. Alam MZ, Masud MM, Rahman MS, Cheratta M, Nayeem MA. Feature-ranking-based ensemble classifiers for survivability prediction of intensive care unit patients using lab test data. *Inform Med Unlocked*. 2021;22doi:10.1016/j.imu.2020.100495

22. Al-Dailami A, Kuang H, Wang J. Predicting length of stay in ICU and mortality with temporal dilated separable convolution and context-aware feature fusion. *Comput Biol Med*. 2022;151(Pt A):106278. doi:10.1016/j.compbiomed.2022.106278
23. Alderden J, Kennerly SM, Wilson A, et al. Explainable Artificial Intelligence for Predicting Hospital-Acquired Pressure Injuries in COVID-19-Positive Critical Care Patients. *Comput Inform Nurs*. 2022;40(10):659-665. doi:10.1097/CIN.0000000000000943
24. Aldewereld ZT, Zhang LA, Urbano A, et al. Identification of Clinical Phenotypes in Septic Patients Presenting With Hypotension or Elevated Lactate. *Front Med (Lausanne)*. 2022;9:794423. doi:10.3389/fmed.2022.794423
25. Aldhoayan MD, Aljubran Y. Prediction of ICU Patients' Deterioration Using Machine Learning Techniques. *Cureus*. 2023;15(5):e38659. doi:10.7759/cureus.38659
26. Alfieri F, Ancona A, Tripepi G, et al. A deep-learning model to continuously predict severe acute kidney injury based on urine output changes in critically ill patients. *J Nephrol*. 2021;34(6):1875-1886. doi:10.1007/s40620-021-01046-6
27. Alfieri F, Ancona A, Tripepi G, et al. External validation of a deep-learning model to predict severe acute kidney injury based on urine output changes in critically ill patients. *J Nephrol*. 2022;35(8):2047-2056. doi:10.1007/s40620-022-01335-8
28. Alfieri F, Ancona A, Tripepi G, et al. Continuous and early prediction of future moderate and severe Acute Kidney Injury in critically ill patients: Development and multi-centric, multi-national external validation of a machine-learning model. *PLoS One*. 2023;18(7):e0287398. doi:10.1371/journal.pone.0287398
29. Alge OP, Gryak J, VanEpps JS, Najarian K. Sepsis Trajectory Prediction Using Privileged Information and Continuous Physiological Signals. *Diagnostics (Basel)*. 2024;14(3)doi:10.3390/diagnostics14030234
30. Alghatani K, Ammar N, Rezgui A, Shaban-Nejad A. Predicting Intensive Care Unit Length of Stay and Mortality Using Patient Vital Signs: Machine Learning Model Development and Validation. *JMIR Med Inform*. 2021;9(5):e21347. doi:10.2196/21347
31. Alghatani K, Ammar N, Rezgui A, Shaban-Nejad A. Precision Clinical Medicine Through Machine Learning: Using High and Low Quantile Ranges of Vital Signs for Risk Stratification of ICU Patients. *IEEE Access*. 2022;10:52418-52430. doi:10.1109/access.2022.3175304
32. Alharbi A, Alosaimi W, Sahal R, Saleh H. Real-Time System Prediction for Heart Rate Using Deep Learning and Stream Processing Platforms. *Complexity*. 2021;2021doi:10.1155/2021/5535734
33. Al-Hindawi A, Vizcaychipi M, Demiris Y. A dual-camera eye-tracking platform for rapid real-time diagnosis of acute delirium: a pilot study. *IEEE J Transl Eng Health Med*. 2024:1-1. doi:10.1109/jtehm.2024.3397737
34. Alizadeh B, Alibabaei A, Ahmadi S, Maroufi SF, Ghafouri-Fard S, Nateghinia S. Designing predictive models for appraisal of outcome of neurosurgery patients using machine learning-based techniques. *Interdiscip Neurosurg Adv Tech Case Manage*. 2023;31doi:10.1016/j.inat.2022.101658
35. Al-Mamun MA, Brothers T, Newsome AS. Development of Machine Learning Models to Validate a Medication Regimen Complexity Scoring Tool for Critically Ill Patients. *Ann Pharmacother*. 2021;55(4):421-429. doi:10.1177/1060028020959042
36. Alourani A, Tariq K, Tahir M, Sardaraz M. Patient Mortality Prediction and Analysis of Health Cloud Data Using a Deep Neural Network. *Appl Sci -Basel*. 2023;13(4)doi:10.3390/app13042391
37. Alsinglawi B, Alnajjar F, Mubin O, et al. Predicting Length of Stay for Cardiovascular Hospitalizations in the Intensive Care Unit: Machine Learning Approach. *Annu Int Conf IEEE Eng Med Biol Soc*. 2020;2020:5442-5445. doi:10.1109/embc44109.2020.9175889
38. Alsinglawi B, Alshari O, Alorjani M, et al. An explainable machine learning framework for lung cancer hospital length of stay prediction. *Sci Rep*. 2022;12(1):607. doi:10.1038/s41598-021-04608-7
39. Alves T, Laender A, Veloso A. Dynamic prediction of ICU mortality risk using domain adaptation. *2018 IEEE International …*. 2018;doi:
40. Amacher SA, Arpagaus A, Sahmer C, et al. Prediction of outcomes after cardiac arrest by a generative artificial intelligence model. *Resusc Plus*. 2024;18:100587. doi:10.1016/j.resplu.2024.100587
41. Amador T, Saturnino S, Veloso A, Ziviani N. Early identification of ICU patients at risk of complications: Regularization based on robustness and stability of explanations. *Artif Intell Med*. 2022;128:102283. doi:10.1016/j.artmed.2022.102283
42. Youssef Ali Amer A, Wouters F, Vranken J, et al. Vital Signs Prediction for COVID-19 Patients in ICU. *Sensors (Basel)*. 2021;21(23)doi:10.3390/s21238131

43. Amiri M, Raimondo F, Fisher PM, et al. Multimodal Prediction of 3- and 12-Month Outcomes in ICU Patients with Acute Disorders of Consciousness. *Neurocrit Care*. Apr 2023;40(2):718-733. doi:10.1007/s12028-023-01816-z
44. Amiri M, Fisher PM, Raimondo F, et al. Multimodal prediction of residual consciousness in the intensive care unit: the CONNECT-ME study. *Brain*. 2023;146(1):50-64. doi:10.1093/brain/awac335
45. Amrollahi F, Shashikumar SP, Razmi F, Nemati S. Contextual Embeddings from Clinical Notes Improves Prediction of Sepsis. *AMIA Annu Symp Proc*. 2020;2020:197-202. doi:
46. An R, Chang GM, Fan YY, Ji LL, Wang XH, Hong S. Machine learning-based patient classification system for adult patients in intensive care units: A cross-sectional study. *J Nurs Manag*. 2021;29(6):1752-1762. doi:10.1111/jonm.13284
47. Andersson P, Johnsson J, Bjornsson O, et al. Predicting neurological outcome after out-of-hospital cardiac arrest with cumulative information; development and internal validation of an artificial neural network algorithm. *Crit Care*. 2021;25(1):83. doi:10.1186/s13054-021-03505-9
48. Ang ETY, Nambiar M, Soh YS, Tan VYF. An Interpretable Intensive Care Unit Mortality Risk Calculator. *Annu Int Conf IEEE Eng Med Biol Soc*. 2021;2021:4152-4158. doi:10.1109/embc46164.2021.9631058
49. Anitha T, Gopu G, Devan PAM. Mechanical Ventilator Pressure and Volume Control Using Classifier Machine Learning Algorithm for Medical Care. *J Electr Eng Technol*. 2023;doi:10.1007/s42835-023-01744-0
50. Anitha T, Gopu G, Arun Mozhi Devan P, Assaad M. Machine learning algorithm for ventilator mode selection, pressure and volume control. *PLoS One*. 2024;19(3)doi:10.1371/journal.pone.0299653
51. Annapragada AV, Greenstein JL, Bose SN, Winters BD, Sarma SV, Winslow RL. SWIFT: A deep learning approach to prediction of hypoxemic events in critically-Ill patients using SpO2 waveform prediction. *PLoS Comput Biol*. 2021;17(12):e1009712. doi:10.1371/journal.pcbi.1009712
52. Aoki R, Tung F, Oliveira GL. Heterogeneous Multi-Task Learning With Expert Diversity. *IEEE/ACM Trans Comput Biol Bioinform*. 2022;19(6):3093-3102. doi:10.1109/tcbb.2022.3175456
53. Apalak M, Kiasaleh K. Improving Sepsis Prediction Performance Using Conditional Recurrent Adversarial Networks. *IEEE Access*. 2022;10:134466-134476. doi:10.1109/access.2022.3230324
54. Aref S. Utilizing machine learning to create a blood-based scoring system for sepsis detection. *Eur J Clin Exp Med*. 2023;21(4):830-834. doi:10.15584/ejcem.2023.4.27
55. Asgari P, Miri MM, Asgari F. The comparison of selected machine learning techniques and correlation matrix in ICU mortality risk prediction. *Inform Med Unlocked*. 2022;31doi:10.1016/j.imu.2022.100995
56. Ashrafi N, Liu Y, Xu X, Wang Y, Zhao Z, Pishgar M. Deep learning model utilization for mortality prediction in mechanically ventilated ICU patients. *Informatics in Medicine Unlocked*. 2024/01/01/ 2024;49:101562. doi:https://doi.org/10.1016/j.imu.2024.101562
57. Asrian G, Suri A, Rajapakse C. Machine learning-based mortality prediction in hip fracture patients using biomarkers. *J Orthop Res*. 2024;42(2):395-403. doi:10.1002/jor.25675
58. Asuroglu T, Ogul H. A deep learning approach for sepsis monitoring via severity score estimation. *Comput Methods Programs Biomed*. 2021;198:105816. doi:10.1016/j.cmpb.2020.105816
59. Athaya T, Choi S. An Efficient Fingertip Photoplethysmographic Signal Artifact Detection Method: A Machine Learning Approach. *J Sens*. 2021;2021doi:10.1155/2021/9925033
60. Athaya T, Choi S. Real-Time Cuffless Continuous Blood Pressure Estimation Using 1D Squeeze U-Net Model: A Progress toward mHealth. *Biosensors (Basel)*. 2022;12(8)doi:10.3390/bios12080655
61. Au-Yeung WM, Sevakula RK, Sahani AK, et al. Real-time machine learning-based intensive care unit alarm classification without prior knowledge of the underlying rhythm. *Eur heart j , Digit health*. 2021;2(3):437-445. doi:10.1093/ehjdh/ztab058
62. Ayad A, Hallawa A, Peine A, et al. Predicting Abnormalities in Laboratory Values of Patients in the Intensive Care Unit Using Different Deep Learning Models: Comparative Study. *JMIR Med Inform*. 2022;10(8):e37658. doi:10.2196/37658
63. Bacariza J, Gonzalez FA, Varudo R, et al. Smartphone-based automatic assessment of left ventricular ejection fraction with a silicon chip ultrasound probe: a prospective comparison study in critically ill patients. *Br J Anaesth*. 2023;130(6):e485-e487. doi:10.1016/j.bja.2023.02.032
64. Baedorf-Kassis EN, Glowala J, Póka KB, Wadehn F, Meyer J, Talmor D. Reverse triggering neural network and rules-based automated detection in acute respiratory distress syndrome. *J Crit Care*. 2023;75doi:10.1016/j.jcrc.2023.154256

65. Bahador N, Erikson K, Laurila J, Koskenkari J, Ala-Kokko T, Kortelainen J. A Correlation-Driven Mapping For Deep Learning application in detecting artifacts within the EEG. *J Neural Eng*. 2020;17(5):056018. doi:10.1088/1741-2552/abb5bd
66. Bai Y, Xia J, Huang X, Chen S, Zhan Q. Using machine learning for the early prediction of sepsis-associated ARDS in the ICU and identification of clinical phenotypes with differential responses to treatment. *Front Physiol*. 2022;13:1050849. doi:10.3389/fphys.2022.1050849
67. Baker S, Xiang W, Atkinson I. Continuous and automatic mortality risk prediction using vital signs in the intensive care unit: a hybrid neural network approach. *Sci Rep*. 2020;10(1):21282. doi:10.1038/s41598-020-78184-7
68. Bakkes T, Montree RJH, Mischi M, Mojoli F, Turco S. A machine learning method for automatic detection and classification of patient-ventilator asynchrony. *Annu Int Conf IEEE Eng Med Biol Soc*. 2020;2020:150-153. doi:10.1109/embc44109.2020.9175796
69. Bakkes T, van Diepen A, De Bie A, et al. Automated detection and classification of patient-ventilator asynchrony by means of machine learning and simulated data. *Comput Meth Programs Biomed*. 2023;230doi:10.1016/j.cmpb.2022.107333
70. Baniasadi A, Rezaeirad S, Zare H, Ghassemi MM. Two-Step Imputation and AdaBoost-Based Classification for Early Prediction of Sepsis on Imbalanced Clinical Data. *Crit Care Med*. 2021;49(1):e91-e97. doi:10.1097/ccm.0000000000004705
71. Bao C, Deng F, Zhao S. Machine-learning models for prediction of sepsis patients mortality. *Med Intensiva (Engl Ed)*. 2023;47(6):315-325. doi:10.1016/j.medine.2022.06.024
72. Barakat CS, Sharafutdinov K, Busch J, et al. Developing an Artificial Intelligence-Based Representation of a Virtual Patient Model for Real-Time Diagnosis of Acute Respiratory Distress Syndrome. *Diagnostics (Basel)*. 2023;13(12)doi:10.3390/diagnostics13122098
73. Baral S, Alsadoon A, Prasad PWC, Al Aloussi S, Alsadoon OH. A novel solution of using deep learning for early prediction cardiac arrest in Sepsis patient: enhanced bidirectional long short-term memory (LSTM). *Multimed Tools Appl*. 2021;80(21):32639-32664. doi:10.1007/s11042-021-11176-5
74. Barchitta M, Maugeri A, Favara G, et al. A machine learning approach to predict healthcare-associated infections at intensive care unit admission: findings from the SPIN-UTI project. *J Hosp Infect*. 2021;112:77-86. doi:10.1016/j.jhin.2021.02.025
75. Barchitta M, Maugeri A, Favara G, et al. Early Prediction of Seven-Day Mortality in Intensive Care Unit Using a Machine Learning Model: Results from the SPIN-UTI Project. *J Clin Med*. 2021;10(5)doi:10.3390/jcm10050992
76. Bardak B, Tan M. Improving clinical outcome predictions using convolution over medical entities with multimodal learning. *Artif Intell Med*. 2021;117:102112. doi:10.1016/j.artmed.2021.102112
77. Bashar SK, Hossain MB, Ding E, Walkey AJ, McManus DD, Chon KH. Atrial Fibrillation Detection During Sepsis: Study on MIMIC III ICU Data. *IEEE j biomed health inform*. 2020;24(11):3124-3135. doi:10.1109/jbhi.2020.2995139
78. Bashar SK, Han D, Zieneddin F, et al. Preliminary Results on Density Poincare Plot Based Atrial Fibrillation Detection from Premature Atrial/Ventricular Contractions. *Annu Int Conf IEEE Eng Med Biol Soc*. 2020;2020:2594-2597. doi:10.1109/embc44109.2020.9175216
79. Bashar SK, Ding EY, Walkey AJ, McManus DD, Chon KH. Atrial Fibrillation Prediction from Critically Ill Sepsis Patients. *Biosensors (Basel)*. 2021;11(8)doi:10.3390/bios11080269
80. Bashar SK, Hossain MB, Lazaro J, et al. Feasibility of atrial fibrillation detection from a novel wearable armband device. *Cardiovasc Digit Health J*. 2021;2(3):179-191. doi:10.1016/j.cvdhj.2021.05.004
81. Bashar SK, Han D, Zieneddin F, et al. Novel Density Poincare Plot Based Machine Learning Method to Detect Atrial Fibrillation From Premature Atrial/Ventricular Contractions. *IEEE Trans Biomed Eng*. 2021;68(2):448-460. doi:10.1109/tbme.2020.3004310
82. Bataille B, de Selle J, Moussot PE, Marty P, Silva S, Cocquet P. Machine learning methods to improve bedside fluid responsiveness prediction in severe sepsis or septic shock: an observational study. *Br J Anaesth*. 2021;126(4):826-834. doi:10.1016/j.bja.2020.11.039
83. Bednarski BP, Singh AD, Zhang W, Jones WM, Naeim A, Ramezani R. Temporal convolutional networks and data rebalancing for clinical length of stay and mortality prediction. *Sci Rep*. 2022;12(1):21247. doi:10.1038/s41598-022-25472-z
84. Begum MF, Narayan S. A pattern mixture model with long short-term memory network for acute kidney injury prediction. *J King Saud Univ -Comput Inf Sci*. 2023;35(4):172-182. doi:10.1016/j.jksuci.2023.03.007
85. Bendavid I, Statlender L, Shvartser L, et al. A novel machine learning model to predict respiratory failure and invasive mechanical ventilation in critically ill patients suffering from COVID-19. *Sci Rep*. 2022;12(1):10573. doi:10.1038/s41598-022-14758-x

86. Itai Bendavid I, Assi S, Sasson N, et al. The EyeControl-Med device, an alternative tool for communication in ventilated critically ill patients: A pilot study examining communication capabilities and delirium. *J Crit Care*. 2023;78doi:10.1016/j.jcrc.2023.154351
87. Benyo B, Palancz B, Szlavecz A, Szabo B, Kovacs K, Chase JG. Classification-based deep neural network vs mixture density network models for insulin sensitivity prediction problem. *Comput Methods Programs Biomed*. 2023;240:107633. doi:10.1016/j.cmpb.2023.107633
88. Berge GT, Granmo OC, Tveit TO, Munkvold BE, Ruthjersen AL, Sharma J. Machine learning-driven clinical decision support system for concept-based searching: a field trial in a Norwegian hospital. *BMC Med Inf Decis Mak*. 2023;23(1):5. doi:10.1186/s12911-023-02101-x
89. Bernabei JM, Owoputi O, Small SD, et al. A Full-Stack Application for Detecting Seizures and Reducing Data During Continuous Electroencephalogram Monitoring. *Crit care explor*. 2021;3(7):e0476. doi:10.1097/cce.0000000000000476
90. Bernard D, Msigwa C, Yun J. Toward IoT-Based Medical Edge Devices: PPG-Based Blood Pressure Estimation Application. *IEEE Internet Things J*. 2023;10(6):5240-5255. doi:10.1109/jiot.2022.3222477
91. Bhaskhar N, Ip W, Chen JH, Rubin DL. Clinical outcome prediction using observational supervision with electronic health records and audit logs. *J Biomed Inform*. 2023;147:104522. doi:10.1016/j.jbi.2023.104522
92. Bhattacharya S, Rajan V, Shrivastava H. ICU mortality prediction: a classification algorithm for imbalanced datasets. *… on Artificial Intelligence*. 2017;doi:
93. Bhattacharyay S, Milosevic I, Wilson L, et al. The leap to ordinal: Detailed functional prognosis after traumatic brain injury with a flexible modelling approach. *PLoS One*. 2022;17(7):e0270973. doi:10.1371/journal.pone.0270973
94. Bhattacharyay S, Caruso PF, Åkerlund C, et al. Mining the contribution of intensive care clinical course to outcome after traumatic brain injury. *NPJ Digit Med*. 2023;6(1)doi:10.1038/s41746-023-00895-8
95. Bhattacharyya A, Sheikhalishahi S, Torbic H, et al. Delirium prediction in the ICU: designing a screening tool for preventive interventions. *JAMIA open*. 2022;5(2):ooac048. doi:10.1093/jamiaopen/ooac048
96. Bi S, Chen S, Li J, Gu J. Machine learning-based prediction of in-hospital mortality for post cardiovascular surgery patients admitting to intensive care unit: a retrospective observational cohort study based on a large multi-center critical care database. *Comput Methods Programs Biomed*. 2022;226:107115. doi:10.1016/j.cmpb.2022.107115
97. Bignoumba N, Mellouli N, Ben Yahia S. A new efficient ALignment-driven Neural Network for Mortality Prediction from Irregular Multivariate Time Series data. *Expert Syst Appl*. 2024;238doi:10.1016/j.eswa.2023.122148
98. Bishara AM, Lituiev DS, Adelmann D, et al. Machine Learning Prediction of Liver Allograft Utilization From Deceased Organ Donors Using the National Donor Management Goals Registry. *Transplant Direct*. 2021;7(10):e771. doi:10.1097/txd.0000000000001212
99. Blaivas M, Adhikari S, Savitsky EA, Blaivas LN, Liu YT. Artificial intelligence versus expert: a comparison of rapid visual inferior vena cava collapsibility assessment between POCUS experts and a deep learning algorithm. *J Am Coll Emerg Physicians Open*. 2020;1(5):857-864. doi:10.1002/emp2.12206
100. Blaivas M, Blaivas L, Philips G, et al. Development of a Deep Learning Network to Classify Inferior Vena Cava Collapse to Predict Fluid Responsiveness. *J Ultrasound Med*. 2021;40(8):1495-1504. doi:10.1002/jum.15527
101. Bo Y, Xie J, Zhou J, Li S, Zhang Y, Zhou Z. Magnetic Resonance Imaging Features of Cerebral Infarction in Critical Patients Based on Convolutional Neural Network. *Contrast Media Mol Imaging*. 2021;2021:4095433. doi:10.1155/2021/4095433
102. Böck M, Malle J, Pasterk D, Kukina H, Hasani R. Superhuman performance on sepsis MIMIC-III data by distributional reinforcement learning. *PLoS One*. 2022;17(11):e0275358. doi:10.1371/journal.pone.0275358
103. Bodenes L, N'Guyen QT, Le Mao R, et al. Early heart rate variability evaluation enables to predict ICU patients' outcome. *Sci Rep*. 2022;12(1):2498. doi:10.1038/s41598-022-06301-9
104. Boie SD, Engelhardt LJ, Coenen N, et al. A Recurrent Neural Network Model for Predicting Activated Partial Thromboplastin Time After Treatment With Heparin: Retrospective Study. *JMIR Med Inform*. 2022;10(10):e39187. doi:10.2196/39187
105. Bollepalli SC, Sevakula RK, Au-Yeung WTM, et al. Real-Time Arrhythmia Detection Using Hybrid Convolutional Neural Networks. *J Am Heart Assoc*. 2021;10(23)doi:10.1161/jaha.121.023222

106. Bologheanu R, Kapral L, Laxar D, et al. Development of a Reinforcement Learning Algorithm to Optimize Corticosteroid Therapy in Critically Ill Patients with Sepsis. *J Clin Med*. 2023;12(4)doi:10.3390/jcm12041513
107. Bolton WJ, Rawson TM, Hern, et al. Machine learning and synthetic outcome estimation for individualised antimicrobial cessation. *Front digit health*. 2022;4:997219. doi:10.3389/fdgth.2022.997219
108. Bolton WJ, Wilson R, Gilchrist M, Georgiou P, Holmes A, Rawson TM. Personalising intravenous to oral antibiotic switch decision making through fair interpretable machine learning. *Nat Commun*. 2024;15(1):506. doi:10.1038/s41467-024-44740-2
109. Bossavi G, Yan RG, Irfan M. A Novel Convolutional Neural Network Deep Learning Implementation for Cuffless Heart Rate and Blood Pressure Estimation. *Appl Sci -Basel*. 2023;13(22)doi:10.3390/app132212403
110. Boussen S, Cordier PY, Malet A, et al. Triage and monitoring of COVID-19 patients in intensive care using unsupervised machine learning. *Comput Biol Med*. 2022;142:105192. doi:10.1016/j.compbiomed.2021.105192
111. Boussen S, Benard-Tertrais M, Ogea M, et al. Heart rate complexity helps mortality prediction in the intensive care unit: A pilot study using artificial intelligence. *Comput Biol Med*. 2024;169:107934. doi:10.1016/j.compbiomed.2024.107934
112. Boutin L, Morisson L, Riche F, et al. Radiomic analysis of abdominal organs during sepsis of digestive origin in a French intensive care unit. *Acute Crit Care*. 2023;38(3):343-352. doi:10.4266/acc.2023.00136
113. Brancato L, Calixto I, Abu-Hanna A, Vagliano I. Leveraging Multi-Word Concepts to Predict Acute Kidney Injury in Intensive Care. *Stud Health Technol Inform*. 2023;305:10-13. doi:10.3233/shti230410
114. Brokowski TJ, Chiang JN. Machine Learning for Antibiotic Stewardship in the Treatment of Stapholycoccus Bacterial Infections. *medRxiv*. 2022:2022.11.28.22282797. doi:10.1101/2022.11.28.22282797
115. Brossard C, Greze J, de Busschere JA, et al. Prediction of therapeutic intensity level from automatic multiclass segmentation of traumatic brain injury lesions on CT-scans. *Sci Rep*. 2023;13(1):20155. doi:10.1038/s41598-023-46945-9
116. Brown MS, Wong KP, Shrestha L, et al. Automated Endotracheal Tube Placement Check Using Semantically Embedded Deep Neural Networks. *Acad Radiol*. 2023;30(3):412-420. doi:10.1016/j.acra.2022.04.022
117. Bucklin AA, Ganglberger W, Quadri SA, et al. High prevalence of sleep-disordered breathing in the intensive care unit - a cross-sectional study. *Sleep Breath*. 2023;27(3):1013-1026. doi:10.1007/s11325-022-02698-9
118. Buell KG, Spicer AB, Casey JD, et al. Individualized Treatment Effects of Oxygen Targets in Mechanically Ventilated Critically Ill Adults. *JAMA*. 2024;331(14):1195-1204. doi:10.1001/jama.2024.2933
119. Bunney G, Murphy J, Colton K, et al. Predicting Early Seizures After Intracerebral Hemorrhage with Machine Learning. *Neurocrit Care*. 2022;37:322-327. doi:10.1007/s12028-022-01470-x
120. Busch F, Xu L, Sushko D, et al. Dual center validation of deep learning for automated multi-label segmentation of thoracic anatomy in bedside chest radiographs. *Comput Methods Programs Biomed*. 2023;234:107505. doi:10.1016/j.cmpb.2023.107505
121. Cai D, Xiao T, Zou A, et al. Predicting acute kidney injury risk in acute myocardial infarction patients: An artificial intelligence model using medical information mart for intensive care databases. *Front cardiovasc med*. 2022;9:964894. doi:10.3389/fcvm.2022.964894
122. Caicedo-Torres W, Gutierrez J. ISeeU2: Visually interpretable mortality prediction inside the ICU using deep learning and free-text medical notes. *Expert Syst Appl*. 2022;202doi:10.1016/j.eswa.2022.117190
123. Callcut RA, Xu Y, Moorman JR, et al. External validation of a novel signature of illness in continuous cardiorespiratory monitoring to detect early respiratory deterioration of ICU patients. *Physiol Meas*. 2021;42(9)doi:10.1088/1361-6579/ac2264
124. Campbell BR, Rooney AS, Krzyzaniak A, et al. Machine Learning Differentiates Extracorporeal Membrane Oxygenation Mortality Risk Profiles Among Trauma Patients. *Am Surg*. 2024:31348241256068. doi:10.1177/00031348241256068
125. Cao Y, Li Y, Wang M, et al. Interpretable machine learning for predicting risk of invasive fungal infection in critically ill patients in the intensive care unit: A retrospective cohort study based on MIMIC-IV database. *Shock*. Jun 1 2024;61(6):817-827. doi:10.1097/shk.0000000000002312

126. Carey S, McInerney C, Lawton T, et al. Fair Reinforcement Learning for Maternal Sepsis Treatment. *medRxiv*. 2022:2022.08.09.22278582. doi:10.1101/2022.08.09.22278582
127. Carra G, Güiza F, Depreitere B, et al. Prediction model for intracranial hypertension demonstrates robust performance during external validation on the CENTER-TBI dataset. *Intensive Care Med*. 2021;47(1):124-126. doi:10.1007/s00134-020-06247-4
128. Carra G, Güiza F, Piper I, et al. Development and External Validation of a Machine Learning Model for the Early Prediction of Doses of Harmful Intracranial Pressure in Patients with Severe Traumatic Brain Injury. *J Neurotrauma*. 2023;40(5):514-522. doi:10.1089/neu.2022.0251
129. Carrasco-Gomez M, Keijzer HM, Ruijter BJ, et al. EEG functional connectivity contributes to outcome prediction of postanoxic coma. *Clin Neurophysiol*. 2021;132(6):1312-1320. doi:10.1016/j.clinph.2021.02.011
130. Ribeiro Carvalho CR, Lamas CA, Chate RC, et al. Long-term respiratory follow-up of ICU hospitalized COVID-19 patients: Prospective cohort study. *PLoS One*. 2023;18(1):e0280567. doi:10.1371/journal.pone.0280567
131. Carvalho RMS, Oliveira D, Pesquita C. Knowledge Graph Embeddings for ICU readmission prediction. *BMC Med Inf Decis Mak*. 2023;23(1):12. doi:10.1186/s12911-022-02070-7
132. Celada-Bernal S, Pérez-Acosta G, Travieso-González CM, Blanco-López J, Santana-Cabrera L. Applying Neural Networks to Recover Values of Monitoring Parameters for COVID-19 Patients in the ICU. *Mathematics*. 2023;11(15)doi:10.3390/math11153332
133. Cena T, Cammarota G, Azzolina D, et al. Predictors of intubation and mortality in COVID-19 patients: a retrospective study. *J Anesth Analg Crit Care*. 2021;1(1):19. doi:10.1186/s44158-021-00016-5
134. Chakraborty A, Sadhukhan D, Pal S, Mitra M. Ppg-Based Automated Estimation of Blood Pressure Using Patient-Specific Neural Network Modeling. *J Mech Med Biol*. 2020;20(6)doi:10.1142/s0219519420500372
135. Chakraborty P, Tharini C. Non-invasive cuff free blood pressure and heart rate measurement from photoplethysmography (PPG) signal using machine learning. *Wirel Pers Commun*. 2024;134(4):2485-2497. doi:10.1007/s11277-024-11070-x
136. Chakraborty S, Kumar K, Tadepalli K, Pailla BR, Roy S. Unleashing the power of explainable AI: sepsis sentinel's clinical assistant for early sepsis identification. *Multimed Tools Appl*. 2023;doi:10.1007/s11042-023-17828-y
137. Chamanzar A, Elmer J, Shutter L, Hartings J, Grover P. Noninvasive and reliable automated detection of spreading depolarization in severe traumatic brain injury using scalp EEG. *Communications Med*. 2023;3(1)doi:10.1038/s43856-023-00344-3
138. Chan MC, Pai KC, Su SA, Wang MS, Wu CL, Chao WC. Explainable machine learning to predict long-term mortality in critically ill ventilated patients: a retrospective study in central Taiwan. *BMC Med Inf Decis Mak*. 2022;22(1):75. doi:10.1186/s12911-022-01817-6
139. Chandar V, Thangamani M. False Alarm Reduction in ICU Using Ensemble Classifier Approach. *Intell Autom Soft Comput*. 2022;34(1):165-181. doi:10.32604/iasc.2022.022339
140. Chang D. Risk prediction of critical vital signs for ICU patients using recurrent neural network. *2019 International …*. 2019;doi:
141. Chang HH, Chiang JH, Wang CS, et al. Predicting Mortality Using Machine Learning Algorithms in Patients Who Require Renal Replacement Therapy in the Critical Care Unit. *J Clin Med*. 2022;11(18)doi:10.3390/jcm11185289
142. Chang P, Li H, Quan SF, et al. A transformer-based diffusion probabilistic model for heart rate and blood pressure forecasting in Intensive Care Unit. *Comput Methods Programs Biomed*. 2024;246:108060. doi:10.1016/j.cmpb.2024.108060
143. Cheema BS, Walter J, Narang A, Thomas JD. Artificial Intelligence-Enabled POCUS in the COVID-19 ICU: A New Spin on Cardiac Ultrasound. *JACC Case Rep*. 2021;3(2):258-263. doi:10.1016/j.jaccas.2020.12.013
144. Chen WT, Long GD, Yao LN, Sheng QZ. AMRNN: attended multi-task recurrent neural networks for dynamic illness severity prediction. *World Wide Web*. 2020;23(5):2753-2770. doi:10.1007/s11280-019-00720-x
145. Chen H, Ma Y, Hong N, et al. Early warning of citric acid overdose and timely adjustment of regional citrate anticoagulation based on machine learning methods. *BMC Med Inf Decis Mak*. 2021;21:126. doi:10.1186/s12911-021-01489-8
146. Chen Z, Chen M, Sun X, et al. Analysis of the Impact of Medical Features and Risk Prediction of Acute Kidney Injury for Critical Patients Using Temporal Electronic Health Record Data With Attention-Based Neural Network. *Front Med (Lausanne)*. 2021;8:658665. doi:10.3389/fmed.2021.658665

147. Chen Y, Liu Z, Li X, et al. Risk factors for mortality due to COVID-19 in intensive care units: a single-center study. *Ann transl med*. 2021;9(4):276. doi:10.21037/atm-20-4877
148. Chen WT, Huang HL, Ko PS, Su W, Kao CC, Su SL. A Simple Algorithm Using Ventilator Parameters to Predict Successfully Rapid Weaning Program in Cardiac Intensive Care Unit Patients. *J Pers Med*. 2022;12(3)doi:10.3390/jpm12030501
149. Chen D, Lin K, Deng Z, Li D, Deng Q. Attention-based convolutional long short-term memory neural network for detection of patient-ventilator asynchrony from mechanical ventilation. *Biomed Signal Process Control*. 2022;78doi:10.1016/j.bspc.2022.103923
150. Chen YP, Huang CH, Lo YH, Chen YY, Lai FP. Combining attention with spectrum to handle missing values on time series data without imputation. *Inf Sci*. 2022;609:1271-1287. doi:10.1016/j.ins.2022.07.124
151. Chen Z, Zeng L, Liu G, et al. Construction of Autophagy-Related Gene Classifier for Early Diagnosis, Prognosis and Predicting Immune Microenvironment Features in Sepsis by Machine Learning Algorithms. *J Inflamm Res*. 2022;15:6165-6186. doi:10.2147/jir.S386714
152. Chen Q, Tang B, Song J, et al. Dynamic Bayesian network for predicting physiological changes, organ dysfunctions and mortality risk in critical trauma patients. *BMC Med Inf Decis Mak*. 2022;22(1):119. doi:10.1186/s12911-022-01803-y
153. Chen YW, Li YJ, Deng P, et al. Learning to predict in-hospital mortality risk in the intensive care unit with attention-based temporal convolution network. *BMC Anesthesiol*. 2022;22(1):119. doi:10.1186/s12871-022-01625-5
154. Chen B, Javadi G, Hamilton A, et al. Quantifying deep neural network uncertainty for atrial fibrillation detection with limited labels. *Sci Rep*. 2022;12(1):20140. doi:10.1038/s41598-022-24574-y
155. Chen M, Hernández A. Towards an Explainable Model for Sepsis Detection Based on Sensitivity Analysis. *Irbm*. 2022;43(1):75-86. doi:10.1016/j.irbm.2021.05.006
156. Chen Q, Li R, Lin C, et al. Transferability and interpretability of the sepsis prediction models in the intensive care unit. *BMC Med Inf Decis Mak*. 2022;22(1):343. doi:10.1186/s12911-022-02090-3
157. Chen J, Qi TD, Vu J, Wen Y. A deep learning approach for inpatient length of stay and mortality prediction. *J Biomed Inform*. 2023;147:104526. doi:10.1016/j.jbi.2023.104526
158. Chen B, Maslove DM, Curran JD, et al. A deep learning model for the classification of atrial fibrillation in critically ill patients. *Intensive care med exp*. 2023;11(1):2. doi:10.1186/s40635-022-00490-3
159. Chen D, Wang R, Jiang Y, et al. Application of artificial neural network in daily prediction of bleeding in ICU patients treated with anti-thrombotic therapy. *BMC Med Inf Decis Mak*. 2023;23(1):171. doi:10.1186/s12911-023-02274-5
160. Chen J, Lowin M, Kellner D, et al. Designing Expert-Augmented Clinical Decision Support Systems to Predict Mortality Risk in ICUs. *Kunstl Intell*. 2023;doi:10.1007/s13218-023-00808-7
161. Chen C, Chen B, Yang J, et al. Development and validation of a practical machine learning model to predict sepsis after liver transplantation. *Ann Med*. 2023;55(1):624-633. doi:10.1080/07853890.2023.2179104
162. Chen Z, Li T, Guo S, Zeng D, Wang K. Machine learning-based in-hospital mortality risk prediction tool for intensive care unit patients with heart failure. *Front cardiovasc med*. 2023;10:1119699. doi:10.3389/fcvm.2023.1119699
163. Chen X, Cheng Q. Acute Complication Prediction and Diagnosis Model CLSTM-BPR: A Fusion Method of Time Series Deep Learning and Bayesian Personalized Ranking. *Tsinghua Sci Technol*. 2024;29(5):1509-1523. doi:10.26599/tst.2023.9010103
164. Chen GH, Li L, Zuo R, Coston A, Weiss JC. Neural topic models with survival supervision: Jointly predicting time-to-event outcomes and learning how clinical features relate. *Artif Intell Med*. 2024;154:102898. doi:10.1016/j.artmed.2024.102898
165. Chen Y, Wang L, Wang GR, et al. Spatio-temporal features for fast early warning of unplanned self-extubation in ICU. *Eng Appl Artif Intell*. 2024;127doi:10.1016/j.engappai.2023.107294
166. Cheng LF, Prasad N, Engelhardt BE. An Optimal Policy for Patient Laboratory Tests in Intensive Care Units. *Pac Symp Biocomput*. 2019;24:320-331. doi:
167. Cheng J, Xu Y, Song R, Liu Y, Li C, Chen X. Prediction of arterial blood pressure waveforms from photoplethysmogram signals via fully convolutional neural networks. *Comput Biol Med*. 2021;138doi:10.1016/j.compbiomed.2021.104877
168. Cheng Y, Chen C, Yang J, et al. Using Machine Learning Algorithms to Predict Hospital Acquired Thrombocytopenia after Operation in the Intensive Care Unit: A Retrospective Cohort Study. *Diagnostics (Basel)*. 2021;11(9)doi:10.3390/diagnostics11091614

169. Cheng J, Sollee J, Hsieh C, et al. COVID-19 mortality prediction in the intensive care unit with deep learning based on longitudinal chest X-rays and clinical data. *Eur Radiol*. 2022;32(7):4446-4456. doi:10.1007/s00330-022-08588-8
170. Cheng Y, Yang J, Wu Q, et al. Machine learning for the prediction of acute kidney injury in patients with acute pancreatitis admitted to the intensive care unit. *Chin Med J*. 2022;135(23):2886-2887. doi:10.1097/cm9.0000000000002531
171. Cheng KH, Tan MC, Chang YJ, et al. The Feasibility of a Machine Learning Approach in Predicting Successful Ventilator Mode Shifting for Adult Patients in the Medical Intensive Care Unit. *Medicina (Kaunas)*. 2022;58(3)doi:10.3390/medicina58030360
172. Cheng YW, Kuo PC, Chen SH, et al. Early prediction of mortality at sepsis diagnosis time in critically ill patients by using interpretable machine learning. *J Clin Monit Comput*. 2024/04/01 2023;38(2):271-279. doi:10.1007/s10877-023-01108-z
173. Cheng N, Bai R, Li L, et al. The influence of biological rhythms on the initial onset of status epilepticus in critically ill inpatients and the study of its predictive Model. *Chronobiol Int*. 2024:1-13. doi:10.1080/07420528.2024.2351490
174. Cherifa M, Interian Y, Blet A, Resche-Rigon M, Pirracchio R. The Physiological Deep Learner: First application of multitask deep learning to predict hypotension in critically ill patients. *Artif Intell Med*. 2021;118:102118. doi:10.1016/j.artmed.2021.102118
175. Chia AHT, Khoo MS, Lim AZ, et al. Explainable machine learning prediction of ICU mortality. *Inform Med Unlocked*. 2021;25doi:10.1016/j.imu.2021.100674
176. Chimbunde E, Sigwadhi LN, Tamuzi JL, et al. Machine learning algorithms for predicting determinants of COVID-19 mortality in South Africa. *Front Artif Intell*. 2023;6doi:10.3389/frai.2023.1171256
177. Chiu CC, Wu CM, Chien TN, Kao LJ, Li C, Jiang HL. Applying an Improved Stacking Ensemble Model to Predict the Mortality of ICU Patients with Heart Failure. *J Clin Med*. 2022;11(21)doi:10.3390/jcm11216460
178. Chiu CC, Wu CM, Chien TN, Kao LJ, Qiu JT. Predicting the Mortality of ICU Patients by Topic Model with Machine-Learning Techniques. *Healthcare (Basel)*. 2022;10(6)doi:10.3390/healthcare10061087
179. Chiu IM, Cheng JY, Chen TY, et al. Using Deep Transfer Learning to Detect Hyperkalemia From Ambulatory Electrocardiogram Monitors in Intensive Care Units: Personalized Medicine Approach. *J Med Internet Res*. 2022;24(12):e41163. doi:10.2196/41163
180. Chiu CC, Wu CM, Chien TN, Kao LJ, Li C, Chu CM. Integrating Structured and Unstructured EHR Data for Predicting Mortality by Machine Learning and Latent Dirichlet Allocation Method. *Int J Environ Res Public Health*. 2023;20(5)doi:10.3390/ijerph20054340
181. Chiu IM, Cheng CY, Chang PK, Li CJ, Cheng FJ, Lin CR. Utilization of Personalized Machine-Learning to Screen for Dysglycemia from Ambulatory ECG, toward Noninvasive Blood Glucose Monitoring. *Biosensors (Basel)*. 2022;13(1)doi:10.3390/bios13010023
182. Cho J, Shin H, Choi A. Calibration-free blood pressure estimation based on a convolutional neural network. *Psychophysiology*. 2023:e14480. doi:10.1111/psyp.14480
183. Choi BK, Kim MS, Kim SH. Risk prediction models for the development of oral-mucosal pressure injuries in intubated patients in intensive care units: A prospective observational study. *J Tissue Viability*. 2020;29(4):252-257. doi:10.1016/j.jtv.2020.06.002
184. Choi MH, Kim D, Choi EJ, et al. Mortality prediction of patients in intensive care units using machine learning algorithms based on electronic health records. *Sci Rep*. 2022;12(1):7180. doi:10.1038/s41598-022-11226-4
185. Choi H, Lee JY, Sul Y, et al. Comparing machine learning and logistic regression for acute kidney injury prediction in trauma patients: A retrospective observational study at a single tertiary medical center. *Medicine (Baltimore)*. 2023;102(33):e34847. doi:10.1097/md.0000000000034847
186. Choi H, Kang HJ, Ahn I, et al. Machine learning models to predict the warfarin discharge dosage using clinical information of inpatients from South Korea. *Sci Rep*. 2023;13(1):22461. doi:10.1038/s41598-023-49831-6
187. Choi JW, Yang M, Kim JW, Shin YM, Shin YG, Park S. Prognostic prediction of sepsis patient using transformer with skip connected token for tabular data. *Artif Intell Med*. 2024;149doi:10.1016/j.artmed.2024.102804
188. Chou SY, Bamodu OA, Chiu WT, Hong CT, Chan L, Chung CC. Artificial neural network-boosted Cardiac Arrest Survival Post-Resuscitation In-hospital (CASPRI) score accurately predicts outcome in cardiac arrest patients treated with targeted temperature management. *Sci Rep*. 2022;12(1):7254. doi:10.1038/s41598-022-11201-z

189. Chou RH, Hsu BW, Yu CL, et al. Machine-learning models are superior to severity scoring systems for the prediction of the mortality of critically ill patients in a tertiary medical center. *J Chin Med Assoc*. 2024;87(4):369-376. doi:10.1097/jcma.0000000000001066
190. Chu Y, Tang K, Hsu YC, et al. Non-invasive arterial blood pressure measurement and SpO2 estimation using PPG signal: a deep learning framework. *BMC Med Inf Decis Mak*. 2023;23(1):131. doi:10.1186/s12911-023-02215-2
191. Churpek MM, Gupta S, Spicer AB, et al. Machine Learning Prediction of Death in Critically Ill Patients With Coronavirus Disease 2019. *Crit care explor*. 2021;3(8):e0515. doi:10.1097/cce.0000000000000515
192. Clare K, Stein A, Damodara N, et al. Safety and efficacy of a novel robotic transcranial doppler system in subarachnoid hemorrhage. *Sci Rep*. 2022;12(1):2266. doi:10.1038/s41598-021-04751-1
193. Clark MG, Mueller DA, Dudaryk R, Li G, Freundlich RE. Patient and Operative Factors Predict Risk of Discretionary Prolonged Postoperative Mechanical Ventilation in a Broad Surgical Cohort. *Anesth Analg*. 2023;136(3):524-531. doi:10.1213/ane.0000000000006205
194. Cleri NA, Saadon JR, Zheng X, et al. Predicting traumatic brain injury outcomes using a posterior dominant rhythm. *J Neurosurg*. 2023;139(6):1523-1533. doi:10.3171/2023.4.Jns23569
195. Cohen S, Dagan N, Cohen-Inger N, Ofer D, Rokach L. ICU Survival Prediction Incorporating Test-Time Augmentation to Improve the Accuracy of Ensemble-Based Models. *IEEE Access*. 2021;9:91584-91592. doi:10.1109/access.2021.3091622
196. Cohen SN, Foster J, Foster P, et al. Subtle variation in sepsis-III definitions markedly influences predictive performance within and across methods. *Sci Rep*. 2024;14(1):1920. doi:10.1038/s41598-024-51989-6
197. Coombes CE, Coombes KR, Fareed N. A novel model to label delirium in an intensive care unit from clinician actions. *BMC Med Inf Decis Mak*. 2021;21(1):97. doi:10.1186/s12911-021-01461-6
198. Coombes CE, Coombes KR, Fareed N. Sequences of Events from the Electronic Medical Record and the Onset of Infection. *Chem Biodiversity*. 2022;19(11)doi:10.1002/cbdv.202200657
199. Crespo-Diaz R, Wolfson J, Yannopoulos D, Bartos JA. Machine Learning Identifies Higher Survival Profile In Extracorporeal Cardiopulmonary Resuscitation. *Crit Care Med*. Jul 1 2024;52(7):1065-1076. doi:10.1097/ccm.0000000000006261
200. Cuadrado D, Valls A, Riaño D. Predicting Intensive Care Unit Patients' Discharge Date with a Hybrid Machine Learning Model That Combines Length of Stay and Days to Discharge. *Mathematics*. 2023;11(23)doi:10.3390/math11234773
201. Cui W, Ge S, Shi Y, et al. Death after discharge: prognostic model of 1-year mortality in traumatic brain injury patients undergoing decompressive craniectomy. *Chin Neurosurg J*. 2021;7(1)doi:10.1186/s41016-021-00242-4
202. Cui Y, Zhou Y, Liu C, Mao Z, Zhou F. Interpretable machine learning models for predicting the incidence of antibiotic- associated diarrhea in elderly ICU patients. *BMC Geriatr*. 2024;24(1):458. doi:10.1186/s12877-024-05028-8
203. Cysneiros A, Galvao T, Domingues N, Jorge P, Bento L, Martin-Loeches I. ARDS Mortality Prediction Model Using Evolving Clinical Data and Chest Radiograph Analysis. *Biomedicines*. Feb 16 2024;12(2)doi:10.3390/biomedicines12020439
204. Daenen K, Tong-Minh K, Liesenfeld O, et al. A Transcriptomic Severity Classifier IMX-SEV-3b to Predict Mortality in Intensive Care Unit Patients with COVID-19: A Prospective Observational Pilot Study. *J Clin Med*. 2023;12(19)doi:10.3390/jcm12196197
205. Dai H, Hwang HG, Tseng VS. PoEMS: Policy Network-Based Early Warning Monitoring System for Sepsis in Intensive Care Units. *IEEE j biomed health inform*. 2023;27(7):3610-3621. doi:10.1109/jbhi.2023.3272486
206. Dai PY, Wu YC, Sheu RK, et al. An automated ICU agitation monitoring system for video streaming using deep learning classification. *BMC Med Inf Decis Mak*. 2024;24(1):77. doi:10.1186/s12911-024-02479-2
207. Dala Ali AHH, Harun SN, Othman N, et al. Determinants of Inadequate Empiric Antimicrobial Therapy in ICU Sepsis Patients in Al-Madinah Al-Munawwarah, Saudi Arabia: A Comparison of Artificial Neural Network and Regression Analysis. *Antibiotics*. 2023;12doi:10.3390/antibiotics12081305
208. Dam TA, Roggeveen LF, van Diggelen F, et al. Predicting responders to prone positioning in mechanically ventilated patients with COVID-19 using machine learning. *Ann Intensive Care*. 2022;12(1):99. doi:10.1186/s13613-022-01070-0
209. Danilatou V, Nikolakakis S, Antonakaki D, et al. Outcome Prediction in Critically-Ill Patients with Venous Thromboembolism and/or Cancer Using Machine Learning Algorithms: External

Validation and Comparison with Scoring Systems. *Int J Mol Sci*. 2022;23(13)doi:10.3390/ijms23137132
210. Danilovich I, Moshkin V, Reimche A, Tevelevich M, Mikhaylovskiy N. Video monitoring over anti-decubitus protocol execution with a deep neural network to prevent pressure ulcer. *Annu Int Conf IEEE Eng Med Biol Soc*. 2021;2021:1384-1387. doi:10.1109/embc46164.2021.9630830
211. Darabi S, Kachuee M, Fazeli S, Sarrafzadeh M. TAPER: Time-Aware Patient EHR Representation. *IEEE j biomed health inform*. 2020;24(11):3268-3275. doi:10.1109/jbhi.2020.2984931
212. Das PP, Wiese L, Mast M, et al. An attention-based bidirectional LSTM-CNN architecture for the early prediction of sepsis. *Int J, Data Sci Anal*. 2024;doi:10.1007/s41060-024-00568-z
213. Dave C, Wu D, Tschirhart J, et al. Prospective Real-Time Validation of a Lung Ultrasound Deep Learning Model in the ICU. *Crit Care Med*. 2023;51(2):301-309. doi:10.1097/ccm.0000000000005759
214. de Haro C, Santos-Pulpon V, Telias I, et al. Flow starvation during square-flow assisted ventilation detected by supervised deep learning techniques. *Crit Care*. 2024;28(1):75. doi:10.1186/s13054-024-04845-y
215. de Hond AAH, Kant IMJ, Fornasa M, et al. Predicting Readmission or Death After Discharge From the ICU: External Validation and Retraining of a Machine Learning Model. *Crit Care Med*. 2023;51(2):291-300. doi:10.1097/ccm.0000000000005758
216. de Jonge M, Wubben N, van Kaam CR, et al. Optimizing an existing prediction model for quality of life one-year post-intensive care unit: An exploratory analysis. *Acta Anaesthesiol Scand*. 2022;66(10):1228-1236. doi:10.1111/aas.14138
217. de Oliveira Gomes BF, da Silva TMB, Dutra GP, et al. Late Mortality After Myocardial Injury in Critical Care Non-Cardiac Surgery Patients Using Machine Learning Analysis. *Am J Cardiol*. 2023;204:70-76. doi:10.1016/j.amjcard.2023.07.044
218. De Silva K, Mathews N, Teede H, et al. Clinical notes as prognostic markers of mortality associated with diabetes mellitus following critical care: A retrospective cohort analysis using machine learning and unstructured big data. *Comput Biol Med*. 2021;132:104305. doi:10.1016/j.compbiomed.2021.104305
219. Deasy J, Ercole A, Liò P. Impact of novel aggregation methods for flexible, time-sensitive EHR prediction without variable selection or cleaning. *arXiv preprint arXiv:1909 08981*. 2019;doi:
220. Deasy J, Lio P, Ercole A. Dynamic survival prediction in intensive care units from heterogeneous time series without the need for variable selection or curation. *Sci Rep*. 2020;10(1):22129. doi:10.1038/s41598-020-79142-z
221. Delgado R, Nunez-Gonzalez JD, Yebenes JC, Lavado A. Survival in the Intensive Care Unit: A prognosis model based on Bayesian classifiers. *Artif Intell Med*. 2021;115:102054. doi:10.1016/j.artmed.2021.102054
222. den Hengst F, Otten M, Elbers P, van Harmelen F, François-Lavet V, Hoogendoorn M. Guideline-informed reinforcement learning for mechanical ventilation in critical care. *Artif Intell Med*. 2024;147doi:10.1016/j.artmed.2023.102742
223. Deng Y, Liu S, Wang Z, Wang Y, Jiang Y, Liu B. Explainable time-series deep learning models for the prediction of mortality, prolonged length of stay and 30-day readmission in intensive care patients. *Front Med (Lausanne)*. 2022;9:933037. doi:10.3389/fmed.2022.933037
224. Deng T, Hamdan H, Yaakob R, Kasmiran KA. Personalized Federated Learning for In-Hospital Mortality Prediction of Multi-Center ICU. *IEEE Access*. 2023;11:11652-11663. doi:10.1109/access.2023.3241488
225. Deng F, Cao Y, Zhao S. Upper gastrointestinal haemorrhage patients' survival: A causal inference and prediction study. *Eur J Clin Invest*. 2024:e14180. doi:10.1111/eci.14180
226. Dhamala J, Azuh E, Al-Dujaili A, Rubin J, O'Reilly UM. Multivariate Time-Series Similarity Assessment via Unsupervised Representation Learning and Stratified Locality Sensitive Hashing: Application to Early Acute Hypotensive Episode Detection. *IEEE Sens Lett*. 2019;3(1)doi:10.1109/lsens.2018.2877920
227. Ding N, Guo C, Li C, Zhou Y, Chai X. An Artificial Neural Networks Model for Early Predicting In-Hospital Mortality in Acute Pancreatitis in MIMIC-III. *Biomed Res Int*. 2021;2021:6638919. doi:10.1155/2021/6638919
228. Ding M, Luo Y. Unsupervised phenotyping of sepsis using nonnegative matrix factorization of temporal trends from a multivariate panel of physiological measurements. *BMC Med Inf Decis Mak*. 2021;21:95. doi:10.1186/s12911-021-01460-7
229. Ding C, Xiao R, Do DH, et al. Log-Spectral Matching GAN: PPG-Based Atrial Fibrillation Detection can be Enhanced by GAN-Based Data Augmentation With Integration of Spectral Loss. *IEEE J Biomedical Health Informat*. 2023;27(3):1331-1341. doi:10.1109/jbhi.2023.3234557

230. Dong L, Liu P, Qi Z, Lin J, Duan M. Development and validation of a machine-learning model for predicting the risk of death in sepsis patients with acute kidney injury. *Heliyon*. 2024;10(9):e29985. doi:10.1016/j.heliyon.2024.e29985
231. Du H, Siah KTH, Ru-Yan VZ, et al. Prediction of in-hospital mortality of Clostriodiodes difficile infection using critical care database: a big data-driven, machine learning approach. *BMJ Open Gastroenterol*. 2021;8(1)doi:10.1136/bmjgast-2021-000761
232. Du H, Pan Z, Ngiam KY, Wang F, Shum P, Feng M. Self-Correcting Recurrent Neural Network for Acute Kidney Injury Prediction in Critical Care. *Health data sci*. 2021;2021:9808426. doi:10.34133/2021/9808426
233. Duanmu H, Ren T, Li H, et al. Deep learning of longitudinal chest X-ray and clinical variables predicts duration on ventilator and mortality in COVID-19 patients. *Biomed Eng Online*. 2022;21(1)doi:10.1186/s12938-022-01045-z
234. Dung-Hung C, Cong T, Zeyu J, Yu-Shan OY, Yung-Yan L. External validation of a machine learning model to predict hemodynamic instability in intensive care unit. *Crit Care*. 2022;26(1):215. doi:10.1186/s13054-022-04088-9
235. Dupont T, Kentish-Barnes N, Pochard F, Duchesnay E, Azoulay E. Prediction of post-traumatic stress disorder in family members of ICU patients: a machine learning approach. *Intensive Care Med*. 2024;50(1):114-124. doi:10.1007/s00134-023-07288-1
236. Edinburgh T, Smielewski P, Czosnyka M, Cabeleira M, Eglen SJ, Ercole A. DeepClean: Self-Supervised Artefact Rejection for Intensive Care Waveform Data Using Deep Generative Learning. *Acta Neurochir Suppl*. 2021;131:235-241. doi:10.1007/978-3-030-59436-7_45
237. Eghbali N, Alhanai T, Ghassemi MM. Patient-Specific Sedation Management via Deep Reinforcement Learning. *Front digit health*. 2021;3:608893. doi:10.3389/fdgth.2021.608893
238. Eickelberg G, Sanchez-Pinto LN, Luo Y. Predictive modeling of bacterial infections and antibiotic therapy needs in critically ill adults. *J Biomed Inform*. 2020;109:103540. doi:10.1016/j.jbi.2020.103540
239. Eickelberg G, Sanchez-Pinto LN, Kline AS, Luo Y. Transportability of bacterial infection prediction models for critically ill patients. *J Am Med Inform Assoc*. 2023;31(1):98-108. doi:10.1093/jamia/ocad174
240. Eini-Porat B, Eytan D, Shalit U. Aiming for Relevance. *AMIA Summits Transl Sci Proc*. 2024;2024:145-154. doi:
241. Elaanba A, Ridouani M, Hassouni L. A Stacked Generalization Chest-X-Ray-Based Framework for Mispositioned Medical Tubes and Catheters Detection. *Biomed Signal Process Control*. 2023;79doi:10.1016/j.bspc.2022.104111
242. El-Ganainy NO, Balasingham I, Halvorsen PS, Rossel, A L. A New Real Time Clinical Decision Support System Using Machine Learning for Critical Care Units. *IEEE Access*. 2020;8:185676-185687. doi:10.1109/access.2020.3030031
243. El-Hajj C, Kyriacou PA. Cuffless blood pressure estimation from PPG signals and its derivatives using deep learning models. *Biomed Signal Process Control*. 2021;70doi:10.1016/j.bspc.2021.102984
244. El-Hajj C, Kyriacou PA. Deep learning models for cuffless blood pressure monitoring from PPG signals using attention mechanism. *Biomed Signal Process Control*. 2021;65doi:10.1016/j.bspc.2020.102301
245. Elhazmi A, Al-Omari A, Sallam H, et al. Machine learning decision tree algorithm role for predicting mortality in critically ill adult COVID-19 patients admitted to the ICU. *J Infect Public Health*. 2022;15(7):826-834. doi:10.1016/j.jiph.2022.06.008
246. El-Manzalawy Y, Abbas M, Hoaglund I, et al. OASIS +: leveraging machine learning to improve the prognostic accuracy of OASIS severity score for predicting in-hospital mortality. *BMC Med Inf Decis Mak*. 2021;21(1):156. doi:10.1186/s12911-021-01517-7
247. Elmer J, Kurz MC, Coppler PJ, et al. Time to Awakening and Self-Fulfilling Prophecies after Cardiac Arrest. *Crit Care Med*. 2023;51(4):503-512. doi:10.1097/ccm.0000000000005790
248. El-Rashidy N, El-Sappagh S, Abuhmed T, Abdelrazek S, El-Bakry HM. Intensive Care Unit Mortality Prediction: An Improved Patient-Specific Stacking Ensemble Model. *IEEE Access*. 2020;8:133541-133564. doi:10.1109/access.2020.3010556
249. El-Rashidy N, Abuhmed T, Alarabi L, et al. Sepsis prediction in intensive care unit based on genetic feature optimization and stacked deep ensemble learning. *Neural Comput Appl*. 2022;34(5):3603-3632. doi:10.1007/s00521-021-06631-1
250. El-Rashidy N, ElSayed NE, El-Ghamry A, Talaat FM. Utilizing fog computing and explainable deep learning techniques for gestational diabetes prediction. *Neural Comput Appl*. 2023;35(10):7423-7442. doi:10.1007/s00521-022-08007-5

251. Eltaybani S. Capabilities and limitations of large language models in critical care nursing research: Examples from the big three. *Nurs Crit Care*. 2023;28(6):838-853. doi:10.1111/nicc.12974
252. Emdad FB, Tian S, y E, Hanna K, He Z. Towards Interpretable Multimodal Predictive Models for Early Mortality Prediction of Hemorrhagic Stroke Patients. *AMIA Summits Transl Sci Proc*. 2023;2023:128-137. doi:
253. Eskandari MA, Moridani MK, S M. Detection of sepsis using biomarkers based on machine learning. *Bratisl Lek Listy*. 2023;124(3):239-250. doi:10.4149/bll_2023_037
254. Essay PT, Mosier JM, Nayebi A, Fisher JM, Subbian V. Predicting Failure of Noninvasive Respiratory Support Using Deep Recurrent Learning. *Respir Care*. 2023;68(4):488-496. doi:10.4187/respcare.10382
255. Fabregat A, Magret M, Ferre JA, et al. A Machine Learning decision-making tool for extubation in Intensive Care Unit patients. *Comput Methods Programs Biomed*. 2021;200:105869. doi:10.1016/j.cmpb.2020.105869
256. Fachet M, Mushunuri RV, Bergmann CB, Marzi I, Hoeschen C, Relja B. Utilizing predictive machine-learning modelling unveils feature-based risk assessment system for hyperinflammatory patterns and infectious outcomes in polytrauma. *Front Immunol*. 2023;14:1281674. doi:10.3389/fimmu.2023.1281674
257. Faghihpirayesh R, Ruf S, Rocca M, et al. Automatic Detection of EEG Epileptiform Abnormalities in Traumatic Brain Injury using Deep Learning. *Annu Int Conf IEEE Eng Med Biol Soc*. 2021;2021:302-305. doi:10.1109/embc46164.2021.9630242
258. Fahmi F, Apriyulida F, Nasution IK, Sawaluddin. Automatic Detection of Brain Tumor on Computed Tomography Images for Patients in the Intensive Care Unit. *J Healthc Eng*. 2020;2020:2483285. doi:10.1155/2020/2483285
259. Falter M, Godderis D, Scherrenberg M, et al. Using natural language processing for automated classification of disease and to identify misclassified ICD codes in cardiac disease. *Eur Heart J -Digit Health*. 2024;doi:10.1093/ehjdh/ztae008
260. Fan XM, Wang HL, Zhao Y, Li Y, Tsui KL. An Adaptive Weight Learning-Based Multitask Deep Network for Continuous Blood Pressure Estimation Using Electrocardiogram Signals. *Sensors*. 2021;21(5)doi:10.3390/s21051595
261. Fan G, Yang S, Liu H, et al. Machine Learning-based Prediction of Prolonged Intensive Care Unit Stay for Critical Patients with Spinal Cord Injury. *Spine (Phila Pa 1976)*. 2022;47(9):E390-E398. doi:10.1097/brs.0000000000004267
262. Fan Z, Jiang J, Xiao C, et al. Construction and validation of prognostic models in critically Ill patients with sepsis-associated acute kidney injury: interpretable machine learning approach. *J Transl Med*. 2023;21(1):406. doi:10.1186/s12967-023-04205-4
263. Fan G, Liu H, Yang S, et al. Early prognostication of critical patients with spinal cord injury: A machine learning study with 1485 cases. *Spine (Phila Pa 1976)*. Jun 1 2023;49(11):754-762. doi:10.1097/brs.0000000000004861
264. Fan T, Wang J, Li L, Kang J, Wang W, Zhang C. Predicting the risk factors of diabetic ketoacidosis-associated acute kidney injury: A machine learning approach using XGBoost. *Front public health*. 2023;11:1087297. doi:10.3389/fpubh.2023.1087297
265. Faris Ali N, Atef M. An efficient hybrid LSTM-ANN joint classification-regression model for PPG based blood pressure monitoring. *Biomed Signal Process Control*. 2023;84doi:10.1016/j.bspc.2023.104782
266. Farzaneh N, Ansari S, Lee E, Ward KR, Sjoding MW. Collaborative strategies for deploying artificial intelligence to complement physician diagnoses of acute respiratory distress syndrome. *NPJ Digit Med*. 2023;6(1)doi:10.1038/s41746-023-00797-9
267. Fathi M, Moghaddam NM, Jahromi SN. A prognostic model for 1-month mortality in the postoperative intensive care unit. *Surg Today*. 2022;52(5):795-803. doi:10.1007/s00595-021-02391-6
268. Fathi M, Moghaddam NM, Balaye Jame SZ, Darvishi M, Mortazavi M. The association of Glasgow Coma Scale score with characteristics of patients admitted to the intensive care unit. *Inform Med Unlocked*. 2022;29doi:10.1016/j.imu.2022.100904
269. Faucoz O, St, arovski D, et al. Moving beyond the lines: lung ultrasound pixel-wise computer-assisted analysis for critically ill patients. *Crit Care*. 2023;27(1)doi:10.1186/s13054-022-04219-2
270. Feng X, Pan S, Yan M, et al. Dynamic prediction of late noninvasive ventilation failure in intensive care unit using a time adaptive machine model. *Comput Methods Programs Biomed*. 2021;208:106290. doi:10.1016/j.cmpb.2021.106290
271. Feng X, Wang D, Pan Q, et al. Reinforcement Learning Model for Managing Noninvasive Ventilation Switching Policy. *IEEE J Biomed Health Inform*. 2023;doi:10.1109/jbhi.2023.3274568

272. Feng Z, Prosperi M, Guo Y, Bian J. Variational Temporal Deconfounder for Individualized Treatment Effect Estimation with Longitudinal Observational Data. *Res Sq*. Feb 6 2023;doi:10.21203/rs.3.rs-2536079/v1
273. Feng X, Zhu S, Shen Y, et al. Multi-organ spatiotemporal information aware model for sepsis mortality prediction. *Artif Intell Med*. 2024;147doi:10.1016/j.artmed.2023.102746
274. Feretzakis G, Sakagianni A, Loupelis E, et al. Using Machine Learning to Predict Antimicrobial Resistance of Acinetobacter Baumannii, Klebsiella Pneumoniae and Pseudomonas Aeruginosa Strains. *Stud Health Technol Inform*. 2021;281:43-47. doi:10.3233/shti210117
275. Festor P, Jia Y, Gordon AC, Faisal AA, Habli I, Komorowski M. Assuring the safety of AI-based clinical decision support systems: a case study of the AI Clinician for sepsis treatment. *BMJ Health Care Inform*. 2022;29(1)doi:10.1136/bmjhci-2022-100549
276. Fitzgerald O, Perez-Concha O, Gallego B, et al. Incorporating real-world evidence into the development of patient blood glucose prediction algorithms for the ICU. *J Am Med Inform Assoc*. 2021;28(8):1642-1650. doi:10.1093/jamia/ocab060
277. Fitzgerald O, Perez-Concha O, Gallego-Luxan B, Metke-Jimenez A, Rudd L, Jorm L. Continuous time recurrent neural networks: Overview and benchmarking at forecasting blood glucose in the intensive care unit. *J Biomed Inform*. 2023;146:104498. doi:10.1016/j.jbi.2023.104498
278. Fleuren LM, Dam TA, Tonutti M, et al. Predictors for extubation failure in COVID-19 patients using a machine learning approach. *Crit Care*. 2021;25(1):448. doi:10.1186/s13054-021-03864-3
279. Fleuren LM, Tonutti M, de Bruin DP, et al. Risk factors for adverse outcomes during mechanical ventilation of 1152 COVID-19 patients: a multicenter machine learning study with highly granular data from the Dutch Data Warehouse. *Intensive care med exp*. 2021;9(1):32. doi:10.1186/s40635-021-00397-5
280. Fong N, Feng J, Hubbard A, Dang LE, Pirracchio R. IntraCranial pressure prediction AlgoRithm using machinE learning (I-CARE): Training and Validation Study. *Crit care explor*. 2024;6(1):e1024. doi:10.1097/cce.0000000000001024
281. Fonseca J, Liu X, Oliveira HP, Pereira T. Mortality prediction using medical time series on TBI patients. *Comput Methods Programs Biomed*. 2023;242:107806. doi:10.1016/j.cmpb.2023.107806
282. Castela Forte J, Yeshmagambetova G, van der Grinten ML, et al. Identifying and characterizing high-risk clusters in a heterogeneous ICU population with deep embedded clustering. *Sci Rep*. 2021;11(1):12109. doi:10.1038/s41598-021-91297-x
283. Franca ARM, Rocha E, Bastos LSL, et al. Development and validation of a machine learning model to predict the use of renal replacement therapy in 14,374 patients with COVID-19. *J Crit Care*. 2024;80:154480. doi:10.1016/j.jcrc.2023.154480
284. Franca ARM, Cantarino JN, Salluh JIF, Bastos L. Generalizing the application of machine learning predictive models across different populations: does a model to predict the use of renal replacement therapy in critically ill COVID-19 patients apply to general intensive care unit patients? *Crit Care Sci*. 2024;36:e20240285en. doi:10.62675/2965-2774.20240285-en
285. Fransen J, Lundin J, Freden F, Huss F. A proof-of-concept study on mortality prediction with machine learning algorithms using burn intensive care data. *Scars Burn Heal*. 2022;8:20595131211066585. doi:10.1177/20595131211066585
286. Fritsch SJ, Riedel M, Marx G, Bickenbach J, Schuppert A. Development of a machine learning model for prediction of the duration of unassisted spontaneous breathing in patients during prolonged weaning from mechanical ventilation. *J Crit Care*. 2024;doi:10.1016/j.jcrc.2024.154795
287. Fu LH, Knaplund C, Cato K, et al. Utilizing timestamps of longitudinal electronic health record data to classify clinical deterioration events. *J Am Med Inform Assoc*. 2021;28(9):1955-1963. doi:10.1093/jamia/ocab111
288. Fu P, Zhang Y, Zhang J, Hu J, Sun Y. Prediction of Intracranial Infection in Patients under External Ventricular Drainage and Neurological Intensive Care: A Multicenter Retrospective Cohort Study. *J Clin Med*. 2022;11(14)doi:10.3390/jcm11143973
289. Fu W, Liu X, Guan L, et al. Prognostic analysis of high-flow nasal cannula therapy and non-invasive ventilation in mild to moderate hypoxemia patients and construction of a machine learning model for 48-h intubation prediction-a retrospective analysis of the MIMIC database. *Front Med (Lausanne)*. 2024;11:1213169. doi:10.3389/fmed.2024.1213169
290. Fuadah YN, Lim KM. Classification of Blood Pressure Levels Based on Photoplethysmogram and Electrocardiogram Signals with a Concatenated Convolutional Neural Network. *Diagn*. 2022;12(11)doi:10.3390/diagnostics12112886
291. Fuest KE, Ulm B, Daum N, et al. Clustering of critically ill patients using an individualized learning approach enables dose optimization of mobilization in the ICU. *Crit Care*. 2023;27(1):1. doi:10.1186/s13054-022-04291-8

292. Fujarski M, Porschen C, Plagwitz L, et al. Prediction of Acute Kidney Injury in the Intensive Care Unit: Preliminary Findings in a European Open Access Database. *Stud Health Technol Inform*. 2022;294:139-140. doi:10.3233/shti220419
293. Futoma J, Masood MA, Doshi-Velez F. Identifying Distinct, Effective Treatments for Acute Hypotension with SODA-RL: Safely Optimized Diverse Accurate Reinforcement Learning. *AMIA Summits Transl Sci Proc*. 2020;2020:181-190. doi:
294. Gakuba C, Le Barbey C, Sar A, et al. Evaluation of ChatGPT in Predicting 6-Month Outcomes After Traumatic Brain Injury. *Crit Care Med*. Jun 1 2024;52(6):942-950. doi:10.1097/ccm.0000000000006236
295. Gandin I, Scagnetto A, Romani S, G. B. Interpretability of time-series deep learning models: A study in cardiovascular patients admitted to Intensive care unit. *J Biomed Inform*. 2021;121:103876. doi:10.1016/j.jbi.2021.103876
296. Ganglberger W, Krishnamurthy PV, Quadri SA, et al. Sleep staging in the ICU with heart rate variability and breathing signals. An exploratory cross-sectional study using deep neural networks. *Front Netw Physiol*. 2023;3:1120390. doi:10.3389/fnetp.2023.1120390
297. Ganzert S, Guttmann J, Kersting K, Kuhlen R. Analysis of respiratory pressure–volume curves in intensive care medicine using inductive machine learning. *Artificial intelligence in ….* 2002;
298. Gao J, Mar PL, Chen G. More Generalizable Models For Sepsis Detection Under Covariate Shift. *AMIA Summits Transl Sci Proc*. 2021;2021:220-228. doi:
299. Gao W, Wang J, Zhou L, et al. Prediction of acute kidney injury in ICU with gradient boosting decision tree algorithms. *Comput Biol Med*. 2021;140:105097. doi:10.1016/j.compbiomed.2021.105097
300. Gao Q, Wang D, Sun P, Luan X, Wang W. Sentiment Analysis Based on the Nursing Notes on In-Hospital 28-Day Mortality of Sepsis Patients Utilizing the MIMIC-III Database. *Comput Math Methods Med*. 2021;2021:3440778. doi:10.1155/2021/3440778
301. Gao CA, Markov NS, Stoeger T, et al. A machine learning approach identifies unresolving secondary pneumonia as a contributor to mortality in patients with severe pneumonia, including COVID-19. *medRxiv*. 2022:2022.09.23.22280118. doi:10.1101/2022.09.23.22280118
302. Gao Y, Miller T, Xu D, Dligach D, Churpek MM, Afshar M. Summarizing Patients' Problems from Hospital Progress Notes Using Pre-trained Sequence-to-Sequence Models. *Proc - Int Conf Comput Ling*. 2022;2022:2979-2991. doi:
303. Gao Y, Wang C, Dong W, et al. An Explainable Machine Learning Model to Predict Acute Kidney Injury After Cardiac Surgery: A Retrospective Cohort Study. *Clin Epidemiol*. 2023;15:1145-1157. doi:10.2147/clep.S404580
304. Gao J, Zhu Y, Wang W, et al. A comprehensive benchmark for COVID-19 predictive modeling using electronic health records in intensive care. *Patterns (N Y)*. 2024;5(4):100951. doi:10.1016/j.patter.2024.100951
305. Gao Z, Liu X, Kang Y, et al. Improving the Prognostic Evaluation Precision of Hospital Outcomes for Heart Failure Using Admission Notes and Clinical Tabular Data: Multimodal Deep Learning Model. *J Med Internet Res*. 2024;26:e54363. doi:10.2196/54363
306. Gao T, Nong Z, Luo Y, et al. Machine learning-based prediction of in-hospital mortality for critically ill patients with sepsis-associated acute kidney injury. *Ren Fail*. 2024;46(1):2316267. doi:10.1080/0886022x.2024.2316267
307. Gao J, Lu Y, Domingo I, Alaei K, Pishgar M. Predicting Sepsis Mortality Using Machine Learning Methods. *medRxiv*. 2024:2024.03.14.24304184. doi:10.1101/2024.03.14.24304184
308. Ge C, Deng F, Chen W, et al. Machine learning for early prediction of sepsis-associated acute brain injury. *Front Med (Lausanne)*. 2022;9:962027. doi:10.3389/fmed.2022.962027
309. Ge W, Huh JW, Park YR, et al. Using deep learning with attention mechanism for identification of novel temporal data patterns for prediction of ICU mortality. *Inform Med Unlocked*. 2022;29doi:10.1016/j.imu.2022.100875
310. George N, Moseley E, Eber R, et al. Deep learning to predict long-term mortality in patients requiring 7 days of mechanical ventilation. *PLoS One*. 2021;16(6):e0253443. doi:10.1371/journal.pone.0253443
311. Getzen E, Ruan Y, Ungar L, Long Q. Mining for Health: A Comparison of Word Embedding Methods for Analysis of EHRs Data. *medRxiv*. 2022:2022.03.05.22271961. doi:10.1101/2022.03.05.22271961
312. Ghanvatkar S, Rajan V. Evaluating Explanations from AI Algorithms for Clinical Decision-Making: A Social Science-based Approach. *IEEE J Biomed Health Inform*. Jul 2024;28(7):4269-4280. doi:10.1109/jbhi.2024.3393719

313. Ghassemi M, Naumann T, Joshi R. Topic models for mortality modeling in intensive care units. *ICML machine learning for clinical data analysis workshop, June*. 2012;
314. Ghassemi M, Naumann T, Doshi-Velez F. Unfolding physiological state: Mortality modelling in intensive care units. *Proceedings of the 20th …*. 2014;doi:10.1145/2623330.2623742
315. Ghassemi M, Pimentel M, Naumann T. A multivariate timeseries modeling approach to severity of illness assessment and forecasting in ICU with sparse, heterogeneous clinical data. *… on artificial intelligence*. 2015;doi:
316. Ghassemi M, Wu M, Hughes MC, Szolovits P, Doshi-Velez F. Predicting intervention onset in the ICU with switching state space models. *AMIA Jt Summits Transl Sci Proc*. 2017;2017:82-91.
317. Ghavidel A, Ghousi R, Atashi A. An ensemble data mining approach to discover medical patterns and provide a system to predict the mortality in the ICU of cardiac surgery based on stacking machine learning method. *Comput Methods Biomech Biomed Eng Imaging and Visualization*. 2023;11(4):1316-1326. doi:10.1080/21681163.2022.2063189
318. Ghazi MM, Sorensen L, Ourselin S, Nielsen M. CARRNN: A Continuous Autoregressive Recurrent Neural Network for Deep Representation Learning From Sporadic Temporal Data. *IEEE Trans Neural Netw Learn Syst*. 2022;doi:10.1109/tnnls.2022.3177366
319. Ghias N, Haq SU, Arshad H, et al. Using Machine Learning Algorithms to predict sepsis and its stages in ICU patients. *medRxiv*. 2022:2022.03.15.22271655. doi:10.1101/2022.03.15.22271655
320. Gholamzadeh M, Abtahi H, Safdari R. Comparison of different machine learning algorithms to classify patients suspected of having sepsis infection in the intensive care unit. *Inform Med Unlocked*. 2023;38doi:10.1016/j.imu.2023.101236
321. Ghorbani R, Ghousi R, Makui A, Atashi A. A New Hybrid Predictive Model to Predict the Early Mortality Risk in Intensive Care Units on a Highly Imbalanced Dataset. *IEEE Access*. 2020;8:141066-141079. doi:10.1109/access.2020.3013320
322. Ghosh E, Eshelman L, Lanius S, et al. Estimation of Baseline Serum Creatinine with Machine Learning. *Am J Nephrol*. 2021;52(9):753-762. doi:10.1159/000518902
323. Ghosheh GO, Thwaites CL, Zhu T. Synthesizing Electronic Health Records for Predictive Models in Low-Middle-Income Countries (LMICs). *Biomedicines*. 2023;11(6)doi:10.3390/biomedicines11061749
324. Giampanis S, Mahajan A, Goldstein T, Norgeot B. MAgEC: Using Non-Homogeneous Ensemble Consensus for Predicting Drivers in Unexpected Mechanical Ventilation. *AMIA Annu Symp Proc*. 2021;2021:238-247. doi:
325. Giang C, Calvert J, Rahmani K, et al. Predicting ventilator-associated pneumonia with machine learning. *Medicine (Baltimore)*. 2021;100(23):e26246. doi:10.1097/md.0000000000026246
326. Giri J, Al-Lohedan HA, Mohammad F, et al. A Comparative Study on Predication of Appropriate Mechanical Ventilation Mode through Machine Learning Approach. *Bioengineering (Basel)*. 2023;10(4)doi:10.3390/bioengineering10040418
327. Goh KH, Wang L, Yeow AYK, et al. Artificial intelligence in sepsis early prediction and diagnosis using unstructured data in healthcare. *Nat Commun*. 2021;12(1):711. doi:10.1038/s41467-021-20910-4
328. Gomes B, Pilz M, Reich C, et al. Machine learning-based risk prediction of intrahospital clinical outcomes in patients undergoing TAVI. *Clin Res Cardiol*. 2021;110(3):343-356. doi:10.1007/s00392-020-01691-0
329. Goncalves JMC, Portela F, Santos MF, et al. Real-Time Predictive Analytics for Sepsis Level and Therapeutic Plans in Intensive Care Medicine. *Int J Healthc Inf Syst Inf*. 2014;9(3):36-54. doi:10.4018/ijhisi.2014070103
330. Gong K, Lee HK, Yu K, Xie X, Li J. A prediction and interpretation framework of acute kidney injury in critical care. *J Biomed Inform*. 2021;113:103653. doi:10.1016/j.jbi.2020.103653
331. Gong W, Cao LX, Zhu YF, Zuo F, He X, Zhou HQ. Federated Inverse Reinforcement Learning for Smart ICUs With Differential Privacy. *IEEE Internet Things J*. 2023;10(21):19117-19124. doi:10.1109/jiot.2023.3281347
332. Gong KD, Lu R, Bergamaschi TS, et al. Predicting Intensive Care Delirium with Machine Learning: Model Development and External Validation. *Anesthesiology*. 2023;138(3):299-311. doi:10.1097/aln.0000000000004478
333. Gong LM, Gong SB, Wu XQ, et al. Predicting Intensive Care Unit Readmission among Patients after Liver Transplantation Using Machine Learning. *Fractals-Complex Geom Patterns Scaling Nat Soc*. 2023;31(6)doi:10.1142/s0218348x23401345
334. Gonzalez-Novoa JA, Busto L, Rodriguez-Andina JJ, et al. Using Explainable Machine Learning to Improve Intensive Care Unit Alarm Systems. *Sensors (Basel)*. 2021;21(21)doi:10.3390/s21217125

335. Gonzalez-Novoa JA, Busto L, Campanioni S, et al. Two-Step Approach for Occupancy Estimation in Intensive Care Units Based on Bayesian Optimization Techniques. *Sensors (Basel)*. 2023;23(3)doi:10.3390/s23031162
336. Gonzalez-Novoa JA, Campanioni S, Busto L, et al. Improving Intensive Care Unit Early Readmission Prediction Using Optimized and Explainable Machine Learning. *Int J Environ Res Public Health*. 2023;20(4)doi:10.3390/ijerph20043455
337. Gordon A, Banerjee I, Block J, et al. Natural language processing of head computed tomography reports to identify intracranial mass effect. *Acad Emerg Med*. 2021;28:S29. doi:10.1111/acem.14249
338. Gordon AJ, Banerjee I, Block J, et al. Natural language processing of head CT reports to identify intracranial mass effect: CTIME algorithm. *Am J Emerg Med*. 2022;51:388-392. doi:10.1016/j.ajem.2021.11.001
339. Gortzis LG, Sakellaropoulos F, Ilias I, Stamoulis K, Lyberopoulos P, Dimopoulou I. Investigating the prognostic accuracy of standardized data mining algorithms in intensive care unit. *J Comput Methods Sci Eng*. 2008;8(4):253-259. doi:
340. Gosset C, Foguenne J, Simul M, et al. Machine learning identification of specific changes in myeloid cell phenotype during bloodstream infections. *Sci Rep*. 2021;11(1):20288. doi:10.1038/s41598-021-99628-8
341. Gourdeau D, Potvin O, Biem JH, et al. Deep learning of chest X-rays can predict mechanical ventilation outcome in ICU-admitted COVID-19 patients. *Sci Rep*. 2022;12(1):6193. doi:10.1038/s41598-022-10136-9
342. Gourdeau D, Potvin O, Archambault P, et al. Tracking and predicting COVID-19 radiological trajectory on chest X-rays using deep learning. *Sci Rep*. 2022;12(1):5616. doi:10.1038/s41598-022-09356-w
343. Grapin K, De Bauchene R, Bonnet B, et al. Severe Acute Respiratory Syndrome Coronavirus 2 Pneumonia in Critically Ill Patients: A Cluster Analysis According to Baseline Characteristics, Biological Features, and Chest CT Scan on Admission. *Crit Care Med*. 2024;52(2):e38-e46. doi:10.1097/ccm.0000000000006105
344. Greco M, Angelotti G, Caruso PF, et al. Outcome prediction during an ICU surge using a purely data-driven approach: A supervised machine learning case-study in critically ill patients from COVID-19 Lombardy outbreak. *Int J Med Inf*. 2022;164:104807. doi:10.1016/j.ijmedinf.2022.104807
345. Grolleau F, Petit F, Gaudry S, et al. Personalizing renal replacement therapy initiation in the intensive care unit: a reinforcement learning-based strategy with external validation on the AKIKI randomized controlled trials. *J Am Med Inform Assoc*. 2024;31(5):1074-1083. doi:10.1093/jamia/ocae004
346. Gu S, Lee EW, Zhang W, Simpson RL, Hertzberg VS, Ho JC. Evaluating Natural Language Processing Packages for Predicting Hospital-Acquired Pressure Injuries From Clinical Notes. *CIN: Computers, Informatics, Nursing*. 2023;42doi:10.1097/cin.0000000000001053
347. Gu LY, Hu HW, Wu SA, et al. Machine learning predictors of risk of death within 7 days in patients with non-traumatic subarachnoid hemorrhage in the intensive care unit: A multicenter retrospective study. *Heliyon*. 2024;10(1)doi:10.1016/j.heliyon.2023.e23943
348. Guan C, Ma F, Chang S, Zhang J. Interpretable machine learning models for predicting venous thromboembolism in the intensive care unit: an analysis based on data from 207 centers. *Crit Care*. 2023;27(1):406. doi:10.1186/s13054-023-04683-4
349. Gulamali F, Jayaraman P, Sawant AS, et al. Derivation, External Validation and Clinical Implications of a deep learning approach for intracranial pressure estimation using non-cranial waveform measurements. Preprint. *medRxiv*. 2024;doi:10.1101/2024.01.30.24301974
350. Guo CH, Liu MC, Lu ML. A Dynamic Ensemble Learning Algorithm based on K-means for ICU mortality prediction. *Appl Soft Comput*. 2021;103doi:10.1016/j.asoc.2021.107166
351. Guo F, Zhu X, Wu Z, Zhu L, Wu J, Zhang F. Clinical applications of machine learning in the survival prediction and classification of sepsis: coagulation and heparin usage matter. *J Transl Med*. 2022;20(1):265. doi:10.1186/s12967-022-03469-6
352. Guo LL, Pfohl SR, Fries J, et al. Evaluation of domain generalization and adaptation on improving model robustness to temporal dataset shift in clinical medicine. *Sci Rep*. 2022;12(1):2726. doi:10.1038/s41598-022-06484-1
353. Guo H, Li J, Liu H, He J. Learning dynamic treatment strategies for coronary heart diseases by artificial intelligence: real-world data-driven study. *BMC Med Inf Decis Mak*. 2022;22(1):39. doi:10.1186/s12911-022-01774-0
354. Guo J, Cheng H, Wang Z, Qiao M, Li J, Lyu J. Factor analysis based on SHapley Additive exPlanations for sepsis-associated encephalopathy in ICU mortality prediction using XGBoost — a

retrospective study based on two large database. *Front Neurol*. 2023;14doi:10.3389/fneur.2023.1290117
355. Guo C, Pan J, Tian S, Gao Y. Using machine learning algorithms to predict 28-day mortality in critically ill elderly patients with colorectal cancer. *J Int Med Res*. 2023;51(11):3000605231198725. doi:10.1177/03000605231198725
356. Guo YR, Leng YX, Gao CJ. Blood Urea Nitrogen-to-Albumin Ratio May Predict Mortality in Patients with Traumatic Brain Injury from the MIMIC Database: A Retrospective Study. *Bioengineering-Basel*. 2024;11(1)doi:10.3390/bioengineering11010049
357. Gupta A, Lash MT, Nachimuthu SK. Optimal sepsis patient treatment using human-in-the-loop artificial intelligence. *Expert Syst Appl*. 2021;169doi:10.1016/j.eswa.2020.114476
358. Gupta S, Singh A, Sharma A, Tripathy RK. Higher Order Derivative-Based Integrated Model for Cuff-Less Blood Pressure Estimation and Stratification Using PPG Signals. *IEEE Sens J*. 2022;22(22):22030-22039. doi:10.1109/jsen.2022.3211993
359. Habibzadeh A, Khademolhosseini S, Kouhpayeh A, et al. Machine learning-based models to predict the need for neurosurgical intervention after moderate traumatic brain injury. *Health Sci Rep*. 2023;6(11):e1666. doi:10.1002/hsr2.1666
360. Hadler RA, Dexter F, Epstein RH. Logistic Regression and Machine Learning Models for Predicting Whether Intensive Care Patients Who Are Alert and Without Delirium Remain As Such for at Least Two More Days. *Cureus*. 2023;15(2):e34913. doi:10.7759/cureus.34913
361. Hagan R, Gillan CJ, Spence I, McAuley D, Shyamsundar M. Comparing regression and neural network techniques for personalized predictive analytics to promote lung protective ventilation in Intensive Care Units. *Comput Biol Med*. 2020;126:104030. doi:10.1016/j.compbiomed.2020.104030
362. Hamar A, Mohammed D, Varadi A, et al. COVID-19 mortality prediction in Hungarian ICU settings implementing random forest algorithm. *Sci Rep*. 2024;14(1):11941. doi:10.1038/s41598-024-62791-9
363. Hameed MAB, Alamgir Z. Improving mortality prediction in Acute Pancreatitis by machine learning and data augmentation. *Comput Biol Med*. 2022;150doi:10.1016/j.compbiomed.2022.106077
364. Hamzi K, Gall E, Roubille F, et al. Phenotypic clustering of patients hospitalized in intensive cardiac care units: Insights from the ADDICT-ICCU study. *Arch Cardiovasc Dis*. Jun-Jul 2024;117(6-7):392-401. doi:S1875-2136(24)00211-0 [pii]
10.1016/j.acvd.2024.03.004
365. Han S, Zhang RF, Shi L, et al. Classifying social determinants of health from unstructured electronic health records using deep learning-based natural language processing. *J Biomed Inform*. 2022;127:103984. doi:10.1016/j.jbi.2021.103984
366. Han F, Zhang Z, Zhang H, Nakaya J, Kudo K, Ogasawara K. Extraction and Quantification of Words Representing Degrees of Diseases: Combining the Fuzzy C-Means Method and Gaussian Membership. *JMIR Form Res*. 2022;6(11):e38677. doi:10.2196/38677
367. Han T, Xiong F, Sun B, Zhong L, Han Z, Lei M. Development and validation of an artificial intelligence mobile application for predicting 30-day mortality in critically ill patients with orthopaedic trauma. *Int J Med Inf*. 2024;184:105383. doi:10.1016/j.ijmedinf.2024.105383
368. Handel C, Frerichs I, Weiler N, Bergh B. Prediction and simulation of PEEP setting effects with machine learning models. *Med Intensiva (Engl Ed)*. Apr 2024;48(4):191-199. doi:S2173-5727(23)00250-3 [pii]
10.1016/j.medine.2023.09.005
369. Hanin A, Demeret S, Lambrecq V, et al. Clinico-biological markers for the prognosis of status epilepticus in adults. *J Neurol*. 2022;269(11):5868-5882. doi:10.1007/s00415-022-11199-4
370. Nateghi Haredasht F, Viaene L, Pottel H, De Corte W, Vens C. Predicting outcomes of acute kidney injury in critically ill patients using machine learning. *Sci Rep*. 2023;13(1):9864. doi:10.1038/s41598-023-36782-1
371. Harerimana G, Kim JW, Jang B. A deep attention model to forecast the Length Of Stay and the in-hospital mortality right on admission from ICD codes and demographic data. *J Biomed Informatics*. 2021;118doi:10.1016/j.jbi.2021.103778
372. Harerimana G, Kim JW, Jang B. A Multi-Headed Transformer Approach for Predicting the Patient's Clinical Time-Series Variables From Charted Vital Signs. *IEEE Access*. 2022;10:105993-106004. doi:10.1109/access.2022.3211334
373. Hasegawa D, Yamakawa K, Nishida K, Okada N, Murao S, Nishida O. Comparative analysis of three machine-learning techniques and conventional techniques for predicting sepsis-induced coagulopathy progression. *J Clin Med*. 2020;9(7):1-10. doi:10.3390/jcm9072113
374. Hashir M, Sawhney R. Towards unstructured mortality prediction with free-text clinical notes. *J Biomed Inform*. 2020;108doi:10.1016/j.jbi.2020.103489

375. Hassanzadeh R, Farhadian M, Rafieemehr H. Hospital mortality prediction in traumatic injuries patients: comparing different SMOTE-based machine learning algorithms. *BMC Med Res Methodol*. 2023;23(1):101. doi:10.1186/s12874-023-01920-w
376. He Z, Du L, Zhang P, Zhao R, Chen X, Fang Z. Early Sepsis Prediction Using Ensemble Learning With Deep Features and Artificial Features Extracted From Clinical Electronic Health Records. *Crit Care Med*. 2020;48(12):e1337-e1342. doi:10.1097/ccm.0000000000004644
377. He J, Lin J, Duan M. Application of Machine Learning to Predict Acute Kidney Disease in Patients With Sepsis Associated Acute Kidney Injury. *Front Med (Lausanne)*. 2021;8:792974. doi:10.3389/fmed.2021.792974
378. Heffernan AJ, Judge S, Petrie SM, et al. Association Between Urine Output and Mortality in Critically Ill Patients: A Machine Learning Approach. *Crit Care Med*. 2022;50(3):e263-e271. doi:10.1097/ccm.0000000000005310
379. Hegselmann S, Ertmer C, Volkert T, Gottschalk A, Dugas M, Varghese J. Development and validation of an interpretable 3 day intensive care unit readmission prediction model using explainable boosting machines. *Front Med (Lausanne)*. 2022;9:960296. doi:10.3389/fmed.2022.960296
380. Heimark S, Botker-Rasmussen KG, Stepanov A, et al. Accuracy of non-invasive cuffless blood pressure in the intensive care unit: Promises and challenges. *Front Med (Lausanne)*. 2023;10:1154041. doi:10.3389/fmed.2023.1154041
381. Hempel L, Sadeghi S, Kirsten T. Prediction of Intensive Care Unit Length of Stay in the MIMIC-IV Dataset. *Appl Sci -Basel*. 2023;13(12)doi:10.3390/app13126930
382. Hern, ez-Carnerero A, Sanchez-Marre M, et al. Dimensionality reduction and ensemble of LSTMs for antimicrobial resistance prediction. *Artif Intell Med*. 2023;138:102508. doi:10.1016/j.artmed.2023.102508
383. Herzog AL, von Jouanne-Diedrich HK, Wanner C, et al. COVID-19 and the kidney: A retrospective analysis of 37 critically ill patients using machine learning. *PLoS One*. 2021;16(5):e0251932. doi:10.1371/journal.pone.0251932
384. Hill BL, Rakocz N, Rudas A, et al. Imputation of the continuous arterial line blood pressure waveform from non-invasive measurements using deep learning. *Sci Rep*. 2021;11(1):15755. doi:10.1038/s41598-021-94913-y
385. Hirano Y, Shinmoto K, Okada Y, et al. Machine Learning Approach to Predict Positive Screening of Methicillin-Resistant Staphylococcus aureus During Mechanical Ventilation Using Synthetic Dataset From MIMIC-IV Database. *Front Med (Lausanne)*. 2021;8:694520. doi:10.3389/fmed.2021.694520
386. Hirzallah MI, Bose S, Hu J, Maltz JS. Automation of ultrasonographic optic nerve sheath diameter measurement using convolutional neural networks. *J Neuroimaging*. 2023;33(6):898-903. doi:10.1111/jon.13163
387. Ho JC, Sotoodeh M, Zhang W, Simpson RL, Hertzberg VS. An AdaBoost-based algorithm to detect hospital-acquired pressure injury in the presence of conflicting annotations. *Comput Biol Med*. 2024;168:107754. doi:10.1016/j.compbiomed.2023.107754
388. Holder AL, Shashikumar SP, Wardi G, Buchman TG, Nemati S. A Locally Optimized Data-Driven Tool to Predict Sepsis-Associated Vasopressor Use in the ICU. *Crit Care Med*. 2021;49(12):e1196-e1205. doi:10.1097/ccm.0000000000005175
389. Holmgren G, Andersson P, Jakobsson A. Artificial neural networks improve and simplify intensive care mortality prognostication: a national cohort study of 217,289 first-time intensive care unit admissions. *Journal of Intensive Care*. 2019;
390. Hong C, Sun Z, Hao Y, Dong Z, Gu Z, Huang Z. Identifying Patients With Heart Failure Who Are Susceptible to De Novo Acute Kidney Injury: Machine Learning Approach. *JMIR Med Inform*. 2022;10(10):e37484. doi:10.2196/37484
391. Hong L, Feng T, Qiu R, et al. A novel interpretative tool for early prediction of low cardiac output syndrome after valve surgery: online machine learning models. *Ann Med*. 2023;55(2):2293244. doi:10.1080/07853890.2023.2293244
392. Horton WB, Barros AJ, Andris RT, Clark MT, Moorman JR. Pathophysiologic Signature of Impending ICU Hypoglycemia in Bedside Monitoring and Electronic Health Record Data: Model Development and External Validation. *Crit Care Med*. 2022;50(3):e221-e230. doi:10.1097/ccm.0000000000005171
393. Hossein Abad ZS, Kline A, Lee J. Evaluation of Machine Learning-based Patient Outcome Prediction Using Patient-specific Difficulty and Discrimination Indices. *Annu Int Conf IEEE Eng Med Biol Soc*. 2020;2020:5446-5449. doi:10.1109/embc44109.2020.9176622

394. Hou N, Li M, He L, et al. Predicting 30-days mortality for MIMIC-III patients with sepsis-3: a machine learning approach using XGboost. *J Transl Med*. 2020;18(1):462. doi:10.1186/s12967-020-02620-5
395. Hripcsak G, Albers DJ. Evaluating Prediction of Continuous Clinical Values: A Glucose Case Study. *Methods Inf Med*. 2022;61:E35-E44. doi:10.1055/s-0042-1743170
396. Hsu J, Kim H, Gong K, Azad TD, Stevens RD. Hyperacute Prediction of Targeted Temperature Management Outcome After Cardiac Arrest. *medRxiv*. 2023:2023.06.12.23291152. doi:10.1101/2023.06.12.23291152
397. Hu C, Tan Q, Zhang Q, et al. Application of interpretable machine learning for early prediction of prognosis in acute kidney injury. *Comput Struct Biotechnol J*. 2022;20:2861-2870. doi:10.1016/j.csbj.2022.06.003
398. Hu C, Li Y, Wang F, Peng Z. Application of Machine Learning for Clinical Subphenotype Identification in Sepsis. *Infect dis ther*. 2022;11(5):1949-1964. doi:10.1007/s40121-022-00684-y
399. Hu K, Deng XL, Han L, Xiang S, Xiong B, Pinhu L. Development and validation of a predictive model for feeding intolerance in intensive care unit patients with sepsis. *Saudi J Gastroenterol*. 2022;28(1):32-38. doi:10.4103/sjg.sjg_286_21
400. Hu J, Kang XH, Xu FF, Huang KZ, Du B, Weng L. Dynamic prediction of life-threatening events for patients in intensive care unit. *BMC Med Inf Decis Mak*. 2022;22(1):276. doi:10.1186/s12911-022-02026-x
401. Hu C, Li L, Li Y, Wang F, Hu B, Peng Z. Explainable Machine-Learning Model for Prediction of In-Hospital Mortality in Septic Patients Requiring Intensive Care Unit Readmission. *Infect dis ther*. 2022;11(4):1695-1713. doi:10.1007/s40121-022-00671-3
402. Hu C, Li L, Huang W, et al. Interpretable Machine Learning for Early Prediction of Prognosis in Sepsis: A Discovery and Validation Study. *Infect dis ther*. 2022;11(3):1117-1132. doi:10.1007/s40121-022-00628-6
403. Hu J, Fei Y, Li WQ. Predicting the mortality risk of acute respiratory distress syndrome: radial basis function artificial neural network model versus logistic regression model. *J Clin Monit Comput*. 2022;36(3):839-848. doi:10.1007/s10877-021-00716-x
404. Hu W, Jin T, Pan Z, et al. An interpretable ensemble learning model facilitates early risk stratification of ischemic stroke in intensive care unit: Development and external validation of ICU-ISPM. *Comput Biol Med*. 2023;166doi:10.1016/j.compbiomed.2023.107577
405. Hu X, Yin S, Zhang X, et al. Blood pressure stratification using photoplethysmography and light gradient boosting machine. *Front Physiol*. 2023;14:1072273. doi:10.3389/fphys.2023.1072273
406. Hu W, Chen H, Wang H, et al. Identifying high-risk phenotypes and associated harms of delayed time-to-antibiotics in patients with ICU onset sepsis: A retrospective cohort study. *J Crit Care*. 2023;74doi:10.1016/j.jcrc.2022.154221
407. Hu Y, Lui A, Goldstein M, et al. Development and external validation of a dynamic risk score for early prediction of cardiogenic shock in cardiac intensive care units using machine learning. *Eur Heart J Acute Cardiovasc Care*. Jun 30 2024;13(6):472-480. doi:7633877 [pii]
zuae037 [pii]
10.1093/ehjacc/zuae037
408. Hu C, Gao C, Li T, Liu C, Peng Z. Explainable artificial intelligence model for mortality risk prediction in the intensive care unit: a derivation and validation study. *Postgrad Med J*. Mar 18 2024;100(1182):219-227. doi:7578621 [pii]
10.1093/postmj/qgad144
409. Huang H, Liu Y, Wu M, Gao Y, Yu X. Development and validation of a risk stratification model for predicting the mortality of acute kidney injury in critical care patients. *Ann transl med*. 2021;9(4):323. doi:10.21037/atm-20-5723
410. Huang CT, Chang RC, Tsai YL, et al. Entropy-Based Time Window Features Extraction for Machine Learning to Predict Acute Kidney Injury in ICU. *Appl Sci -Basel*. 2021;11(14)doi:10.3390/app11146364
411. Huang B, Liang D, Zou R, et al. Mortality prediction for patients with acute respiratory distress syndrome based on machine learning: a population-based study. *Ann transl med*. 2021;9(9):794. doi:10.21037/atm-20-6624
412. Huang K, Gray TF, Romero-Brufau S, Tulsky JA, Lindvall C. Using nursing notes to improve clinical outcome prediction in intensive care patients: A retrospective cohort study. *J Am Med Inform Assoc*. 2021;28(8):1660-1666. doi:10.1093/jamia/ocab051
413. Huang X, Li B, Huang T, et al. External validation based on transfer learning for diagnosing atelectasis using portable chest X-rays. *Front Med (Lausanne)*. 2022;9:920040. doi:10.3389/fmed.2022.920040

414. Huang Y, Zheng Z, Ma M, et al. Improving the Performance of Outcome Prediction for Inpatients With Acute Myocardial Infarction Based on Embedding Representation Learned From Electronic Medical Records: Development and Validation Study. *J Med Internet Res*. 2022;24(8)doi:10.2196/37486
415. Huang X, Shan S, Khan YA, Salem S, Mohamed A, Attia EA. Risk assessment of ICU patients through deep learning technique: A big data approach. *J glob health*. 2022;12:04044. doi:10.7189/jogh.12.04044
416. Huang J, Jin W, Duan X, et al. Twenty-eight-day in-hospital mortality prediction for elderly patients with ischemic stroke in the intensive care unit: Interpretable machine learning models. *Front public health*. 2022;10:1086339. doi:10.3389/fpubh.2022.1086339
417. Huang MH, Chen CY, Horng MH, et al. Validation of a Deep Learning-based Automatic Detection Algorithm for Measurement of Endotracheal Tube-to-Carina Distance on Chest Radiographs. *Anesthesiology*. 2022;137(6):704-715. doi:10.1097/aln.0000000000004378
418. Huang YZ, Chen YM, Lin CC, Chiu HY, Chang YC. A nursing note-aware deep neural network for predicting mortality risk after hospital discharge. *Int J Nurs Stud*. 2024;156:104797. doi:10.1016/j.ijnurstu.2024.104797
419. Yu M, Huang Z, Zhu Y, Zhou P, Zhu J. Attention-based residual improved U-Net model for continuous blood pressure monitoring by using photoplethysmography signal. *Biomed Signal Process Control*. 2022;75doi:10.1016/j.bspc.2022.103581
420. Huang KY, Hsu YL, Chen HC, et al. Developing a machine-learning model for real-time prediction of successful extubation in mechanically ventilated patients using time-series ventilator-derived parameters. *Front Med (Lausanne)*. 2023;10:1167445. doi:10.3389/fmed.2023.1167445
421. Huang CY, Guiza F, De Vlieger G, et al. Development and validation of clinical prediction models for acute kidney injury recovery at hospital discharge in critically ill adults. *J Clin Monit Comput*. 2023;37(1):113-125. doi:10.1007/s10877-022-00865-7
422. Huang CY, Guiza F, Wouters P, et al. Development and validation of the creatinine clearance predictor machine learning models in critically ill adults. *Crit Care*. 2023;27(1):272. doi:10.1186/s13054-023-04553-z
423. Huang CT, Wang TJ, Kuo LK, et al. Federated machine learning for predicting acute kidney injury in critically ill patients: a multicenter study in Taiwan. *Health Inf Sci Syst*. 2023;11(1)doi:10.1007/s13755-023-00248-5
424. Huang S, Teng Y, Du J, Zhou X, Duan F, Feng C. Internal and external validation of machine learning-assisted prediction models for mechanical ventilation-associated severe acute kidney injury. *Aust Crit Care*. 2023;36(4):604-612. doi:10.1016/j.aucc.2022.06.001
425. Huang J, Chen H, Deng J, et al. Interpretable machine learning for predicting 28-day all-cause in-hospital mortality for hypertensive ischemic or hemorrhagic stroke patients in the ICU: a multi-center retrospective cohort study with internal and external cross-validation. *Front Neurol*. 2023;14:1185447. doi:10.3389/fneur.2023.1185447
426. Huang T, Le D, Yuan L, Xu S, Peng X. Machine learning for prediction of in-hospital mortality in lung cancer patients admitted to intensive care unit. *PLoS One*. 2023;18(1):e0280606. doi:10.1371/journal.pone.0280606
427. Hui V, Litton E, Edibam C, et al. Using machine learning to predict bleeding after cardiac surgery. *Eur J Cardiothorac Surg*. Dec 1 2023;64(6)doi:7260514 [pii]
10.1093/ejcts/ezad297
428. Hung PS, Lin PR, Hsu HH, Huang YC, Wu SH, Kor CT. Explainable Machine Learning-Based Risk Prediction Model for In-Hospital Mortality after Continuous Renal Replacement Therapy Initiation. *Diagnostics (Basel)*. 2022;12(6)doi:10.3390/diagnostics12061496
429. Hunter RB, Jiang S, Nishisaki A, et al. Supervised Machine Learning Applied to Automate Flash and Prolonged Capillary Refill Detection by Pulse Oximetry. *Front Physiol*. 2020;11:564589. doi:10.3389/fphys.2020.564589
430. Huo S, Nelde A, Meisel C, et al. A supervised, externally validated machine learning model for artifact and drainage detection in high-resolution intracranial pressure monitoring data. *J Neurosurg*. 2024:1-9. doi:10.3171/2023.12.Jns231670
431. Hur C, Wi J, Kim Y. Facilitating the development of deep learning models with visual analytics for electronic health records. *Int J Environ Res Public Health*. 2020;17(22):1-14. doi:10.3390/ijerph17228303
432. Hur S, Ko RE, Yoo J, Ha J, Cha WC, Chung CR. A Machine Learning-Based Algorithm for the Prediction of Intensive Care Unit Delirium (PRIDE): Retrospective Study. *JMIR Med Inform*. 2021;9(7):e23401. doi:10.2196/23401

433. Hur S, Min JY, Yoo J, et al. Development and Validation of Unplanned Extubation Prediction Models Using Intensive Care Unit Data: Retrospective, Comparative, Machine Learning Study. *J Med Internet Res*. 2021;23(8):e23508. doi:10.2196/23508
434. Hüser M, Lyu X, Faltys M, et al. A comprehensive ML-based Respiratory Monitoring System for Physiological Monitoring & Resource Planning in the ICU. *medRxiv*. 2024:2024.01.23.24301516. doi:10.1101/2024.01.23.24301516
435. Hutanu A, Molnar AA, Pal K, Gabor MR, Szederjesi J, Dobreanu M. Decision tree analysis as predictor tool for in-hospital mortality in critical SARS-CoV-2 infected patients. *Rev Romana Med Lab*. 2023;31(2):91-106. doi:10.2478/rrlm-2023-0015
436. Hwang CS, Kim YH, Hyun JK, et al. Evaluation of the Photoplethysmogram-Based Deep Learning Model for Continuous Respiratory Rate Estimation in Surgical Intensive Care Unit. *Bioengineering (Basel)*. Oct 19 2023;10(10)doi:bioengineering10101222 [pii]
bioengineering-10-01222 [pii]
10.3390/bioengineering10101222
437. Hyun S, Kaewprag P, Cooper C, Hixon B, Moffatt-Bruce S. Exploration of critical care data by using unsupervised machine learning. *Comput Meth Programs Biomed*. 2020;194doi:10.1016/j.cmpb.2020.105507
438. Ismukhamedova A, Uvaliyeva I, Belginova S. Integrating machine learning in electronic health passport based on WHO study and healthcare resources. *Inform Med Unlocked*. 2024;44doi:10.1016/j.imu.2023.101428
439. Itzhak N, Pessach IM, Moskovitch R. Prediction of acute hypertensive episodes in critically ill patients. *Artif Intell Med*. 2023;139:102525. doi:10.1016/j.artmed.2023.102525
440. Iwase S, Nakada TA, Shimada T, et al. Prediction algorithm for ICU mortality and length of stay using machine learning. *Sci Rep*. 2022;12(1):12912. doi:10.1038/s41598-022-17091-5
441. Iwashyna TJ, Ma C, Wang XQ, Seelye S, Zhu J, Waljee AK. Variation in model performance by data cleanliness and classification methods in the prediction of 30-day ICU mortality, a US nationwide retrospective cohort and simulation study. *BMJ Open*. 2020;10(12):e041421. doi:10.1136/bmjopen-2020-041421
442. Jagesar AR, Otten M, Dam TA, et al. Comparative performance of intensive care mortality prediction models based on manually curated versus automatically extracted electronic health record data. *Int J Med Inf*. 2024;188:105477. doi:10.1016/j.ijmedinf.2024.105477
443. Jajcay N, Bezak B, Segev A, et al. Data processing pipeline for cardiogenic shock prediction using machine learning. *Front cardiovasc med*. 2023;10:1132680. doi:10.3389/fcvm.2023.1132680
444. Jamshidi E, Asgary A, Tavakoli N, et al. Using Machine Learning to Predict Mortality for COVID-19 Patients on Day 0 in the ICU. *Front digit health*. 2021;3:681608. doi:10.3389/fdgth.2021.681608
445. Jang JH, Kim TY, Lim HS, Yoon D. Unsupervised feature learning for electrocardiogram data using the convolutional variational autoencoder. *PLoS One*. 2021;16(12)doi:10.1371/journal.pone.0260612
446. Jaotombo F, Adorni L, Ghattas B, Boyer L. Finding the best trade-off between performance and interpretability in predicting hospital length of stay using structured and unstructured data. *PLoS One*. 2023;18(11):e0289795. doi:10.1371/journal.pone.0289795
447. Jentzer JC, Kashou AH, Attia ZI, et al. Left ventricular systolic dysfunction identification using artificial intelligence-augmented electrocardiogram in cardiac intensive care unit patients. *Int J Cardiol*. 2021;326:114-123. doi:10.1016/j.ijcard.2020.10.074
448. Jentzer JC, Soussi S, Lawler PR, Kennedy JN, Kashani KB. Validation of cardiogenic shock phenotypes in a mixed cardiac intensive care unit population. *Catheter Cardiovasc Interv*. 2022;99(4):1006-1014. doi:10.1002/ccd.30103
449. Jeon K, Lee N, Jeong S, Park MJ, Song W. Immature granulocyte percentage for prediction of sepsis in severe burn patients: a machine leaning-based approach. *BMC Infect Dis*. 2021;21(1):1258. doi:10.1186/s12879-021-06971-2
450. Jeon ET, Lee HJ, Park TY, et al. Machine learning-based prediction of in-ICU mortality in pneumonia patients. *Sci Rep*. 2023;13(1):11527. doi:10.1038/s41598-023-38765-8
451. Jia Y, Kaul C, Lawton T, Murray-Smith R, Habli I. Prediction of weaning from mechanical ventilation using Convolutional Neural Networks. *Artif Intell Med*. 2021;117:102087. doi:10.1016/j.artmed.2021.102087
452. Jiang DZ, Tu G, Jin DH, et al. A hybrid intelligent model for acute hypotensive episode prediction with large-scale data. *Inf Sci*. 2021;546:787-802. doi:10.1016/j.ins.2020.08.033

453. Jiang Z, Bo L, Xu Z, et al. An explainable machine learning algorithm for risk factor analysis of in-hospital mortality in sepsis survivors with ICU readmission. *Comput Methods Programs Biomed*. 2021;204:106040. doi:10.1016/j.cmpb.2021.106040
454. Jiang X, Wang Y, Zhou M, et al. Application of an infrared thermography-based model to detect pressure injuries: a prospective cohort study*. *Br J Dermatol*. 2022;187(4):571-579. doi:10.1111/bjd.21665
455. Jiang X, Dai WF, Cai YR. Comparison of machine learning algorithms to SAPS II in predicting in-hospital mortality of fractures of the pelvis and acetabulum: analyzes based on MIMIC-III database. *All Life*. 2022;15(1):1000-1012. doi:10.1080/26895293.2022.2125448
456. Jiang S, Wang R, Zhang H. Integrated Learning Model-Based Assessment of Enteral Nutrition Support in Neurosurgical Intensive Care Patients. *Biomed Res Int*. 2022;2022:4061043. doi:10.1155/2022/4061043
457. Jiang X, Wang Y, Pan Y, Zhang W. Prediction Models for Sepsis-Associated Thrombocytopenia Risk in Intensive Care Units Based on a Machine Learning Algorithm. *Front Med (Lausanne)*. 2022;9:837382. doi:10.3389/fmed.2022.837382
458. Jiang X, Hu Y, Guo S, Du C, Cheng X. Prediction of persistent acute kidney injury in postoperative intensive care unit patients using integrated machine learning: a retrospective cohort study. *Sci Rep*. 2022;12(1):17134. doi:10.1038/s41598-022-21428-5
459. Jiang J, Yu X, Wang B, Ma L, Guan Y. DECAF: An interpretable deep cascading framework for ICU mortality prediction. *Artif Intell Med*. 2023;138:102437. doi:10.1016/j.artmed.2022.102437
460. Jiang M, Pan CQ, Li J, Xu LG, Li CL. Explainable machine learning model for predicting furosemide responsiveness in patients with oliguric acute kidney injury. *Ren Fail*. 2023;45(1):2151468. doi:10.1080/0886022x.2022.2151468
461. Jiang XD, Zhang WM, Pan YT, Cheng XP. Identification of subphenotypes in critically ill thrombocytopenic patients with different responses to therapeutic interventions: a retrospective study. *Front Med*. 2023;10doi:10.3389/fmed.2023.1166896
462. Jiang Z, Bo L, Wang L, et al. Interpretable machine-learning model for real-time, clustered risk factor analysis of sepsis and septic death in critical care. *Comput Methods Programs Biomed*. 2023;241:107772. doi:10.1016/j.cmpb.2023.107772
463. Jiang Z, Liu L, Du L, et al. Machine learning for the early prediction of acute respiratory distress syndrome (ARDS) in patients with sepsis in the ICU based on clinical data. *Heliyon*. 2024;10(6):e28143. doi:10.1016/j.heliyon.2024.e28143
464. Jiang S, Gai X, Treggiari MM, et al. Soft phenotyping for sepsis via EHR time-aware soft clustering. *J Biomed Informatics*. 2024;152doi:10.1016/j.jbi.2024.104615
465. Jing J, d'Angremont E, Ebrahim S, et al. Rapid annotation of seizures and interictal-ictal-injury continuum EEG patterns. *J Neurosci Methods*. 2021;347:108956. doi:10.1016/j.jneumeth.2020.108956
466. Jing Q, Leung CHC, Wu AR. Cell-Free DNA as Biomarker for Sepsis by Integration of Microbial and Host Information. *Clin Chem*. 2022;68(9):1184-1195. doi:10.1093/clinchem/hvac097
467. Job SM, Tao XH, Li L, et al. Optimal Treatment Strategies for Critical Patients with Deep Reinforcement Learning. *ACM Trans Intell Syst Technol*. 2024;15(2)doi:10.1145/3643856
468. Johansson PI, Henriksen HH, Karvelsson ST, et al. LASSO regression shows histidine and sphingosine 1 phosphate are linked to both sepsis mortality and endothelial damage. *Eur J Med Res*. 2024;29(1)doi:10.1186/s40001-023-01612-7
469. Johnson AEW, Mark RG. Real-time mortality prediction in the Intensive Care Unit. *AMIA Annu Symp Proc*. 2017;2017:994-1003.
470. Johnson N, Parbhoo S, Ross AS, Doshi-Velez F. Learning Predictive and Interpretable Timeseries Summaries from ICU Data. *AMIA Annu Symp Proc*. 2021;2021:581-590. doi:
471. Johnsson J, Bjornsson O, Andersson P, et al. Artificial neural networks improve early outcome prediction and risk classification in out-of-hospital cardiac arrest patients admitted to intensive care. *Crit Care*. 2020;24(1):474. doi:10.1186/s13054-020-03103-1
472. Jonas S, Muller M, Rossetti AO, et al. Diagnostic and prognostic EEG analysis of critically ill patients: A deep learning study. *Neuroimage (Amst)*. 2022;36:103167. doi:10.1016/j.nicl.2022.103167
473. Jung C, Mam, ipoor B, et al. Disease-Course Adapting Machine Learning Prognostication Models in Elderly Patients Critically Ill With COVID-19: Multicenter Cohort Study With External Validation. *JMIR Med Inform*. 2022;10(3):e32949. doi:10.2196/32949
474. Jung MK, Ahn D, Park CM, et al. Prediction of Serious Intracranial Hypertension from Low-Resolution Neuromonitoring in Traumatic Brain Injury: An Explainable Machine Learning Approach. *IEEE J Biomedical Health Informat*. 2023;27(4):1903-1913. doi:10.1109/jbhi.2023.3240460

475. Kaewprag P, Newton C, Vermillion B, Hyun S, Huang K, Machiraju R. Predictive Modeling for Pressure Ulcers from Intensive Care Unit Electronic Health Records. *AMIA Summits Transl Sci Proc*. 2015;2015:82-86. doi:
476. Kamaleswaran R, Lian J, Lin DL, et al. Predicting Volume Responsiveness Among Sepsis Patients Using Clinical Data and Continuous Physiological Waveforms. *AMIA Annu Symp Proc*. 2020;2020:619-628. doi:
477. Kamaleswaran R, Sataphaty SK, Mas VR, Eason JD, Maluf DG. Artificial Intelligence May Predict Early Sepsis After Liver Transplantation. *Front Physiol*. 2021;12:692667. doi:10.3389/fphys.2021.692667
478. Kamboj N, Metcalfe K, Chu CH, Conway A. Predicting Blood Pressure After Nitroglycerin Infusion Dose Titration in Critical Care Units: A Multicenter Retrospective Study. *Comput Inform Nurs*. 2023;doi:10.1097/cin.0000000000001086
479. Kamel Rahimi A, Ghadimi M, van der Vegt AH, et al. Machine learning clinical prediction models for acute kidney injury: the impact of baseline creatinine on prediction efficacy. *BMC Med Inf Decis Mak*. 2023;23(1):207. doi:10.1186/s12911-023-02306-0
480. Kamio T, Ikegami M, Machida Y, Uemura T, Chino N, Iwagami M. Machine learning-based prognostic modeling of patients with acute heart failure receiving furosemide in intensive care units. *Digit Health*. 2023;9:20552076231194933. doi:10.1177/20552076231194933
481. Kang MW, Kim DK, Oh KH, Joo KW, Kim YS, Han SS. Machine learning algorithm to predict mortality in patients undergoing continuous renal replacement therapy. *Journal of the American Society of Nephrology : JASN*. 2019;30:445. doi:
482. Kang S, Park C, Lee J, Yoon D. Machine Learning Model for the Prediction of Hemorrhage in Intensive Care Units. *Healthc inform res*. 2022;28(4):364-375. doi:10.4258/hir.2022.28.4.364
483. Kang Y, Jia X, Wang K, et al. A Clinically Practical and Interpretable Deep Model for ICU Mortality Prediction with External Validation. *AMIA Annu Symp Proc*. 2020;2020:629-637. doi:
484. Kara S, Akers JY, Chang PD. Identification and Localization of Endotracheal Tube on Chest Radiographs Using a Cascaded Convolutional Neural Network Approach. *J Digit Imaging*. 2021;34(4):898-904. doi:10.1007/s10278-021-00463-0
485. Karabacak M, Margetis K. Precision medicine for traumatic cervical spinal cord injuries: accessible and interpretable machine learning models to predict individualized in-hospital outcomes. *Spine J*. 2023;23(12):1750-1763. doi:10.1016/j.spinee.2023.08.009
486. Karabacak M, Margetis K. Prognosis at your fingertips: a machine learning-based web application for outcome prediction in acute traumatic epidural hematoma. *J Neurotrauma*. 2023;doi:10.1089/neu.2023.0122
487. Karabacak M, Jagtiani P, Margetis K. The Predictive Abilities of Machine Learning Algorithms in Patients with Thoracolumbar Spinal Cord Injuries. *World Neurosurg*. 2024;182:e67-e90. doi:10.1016/j.wneu.2023.11.043
488. Karboub K, Tabaa M. A Machine Learning Based Discharge Prediction of Cardiovascular Diseases Patients in Intensive Care Units. *Healthcare (Basel)*. 2022;10(6)doi:10.3390/healthcare10060966
489. Karri R, Chen YP, Burrell AJC, et al. Machine learning predicts the short-term requirement for invasive ventilation among Australian critically ill COVID-19 patients. *PLoS One*. 2022;17(10):e0276509. doi:10.1371/journal.pone.0276509
490. Kashou AH, Noseworthy PA, Lopez-Jimenez F, et al. The effect of cardiac rhythm on artificial intelligence-enabled ECG evaluation of left ventricular ejection fraction prediction in cardiac intensive care unit patients. *Int J Cardiol*. 2021;339:54-55. doi:10.1016/j.ijcard.2021.07.001
491. Katuwal GJ, Chen R. Machine learning model interpretability for precision medicine. *arXiv preprint arXiv:1610 09045*. 2016;doi:
492. Katz S, Suijker J, Hardt C, et al. Decision support system and outcome prediction in a cohort of patients with necrotizing soft-tissue infections. *Int J Med Inf*. 2022;167:104878. doi:10.1016/j.ijmedinf.2022.104878
493. Kaya U, Yılmaz A, Aşar S. Sepsis Prediction by Using a Hybrid Metaheuristic Algorithm: A Novel Approach for Optimizing Deep Neural Networks. *Diagn*. 2023;13(12)doi:10.3390/diagnostics13122023
494. Ke X, Zhang F, Huang G, Wang A. Interpretable Machine Learning to Optimize Early In-Hospital Mortality Prediction for Elderly Patients with Sepsis: A Discovery Study. *Comput Math Methods Med*. 2022;2022:4820464. doi:10.1155/2022/4820464
495. Keats K, Deng S, Chen X, et al. Unsupervised machine learning analysis to identify patterns of ICU medication use for fluid overload prediction. *medRxiv*. Mar 22 2024;doi:2024.03.21.24304663 [pii] 10.1101/2024.03.21.24304663

496. Kennedy CJ, Chiu C, Chapman AC, et al. Negativity and Positivity in the ICU: Exploratory Development of Automated Sentiment Capture in the Electronic Health Record. *Crit care explor*. 2023;5(10):e0960. doi:10.1097/cce.0000000000000960
497. Kessler S, Schroeder D, Korlakov S, et al. Predicting readmission to the cardiovascular intensive care unit using recurrent neural networks. *Digit Health*. 2023;9:20552076221149529. doi:10.1177/20552076221149529
498. Khadanga S, Aggarwal K, Joty S. Using clinical notes with time series data for icu management. *arXiv preprint arXiv …*. 2019;doi:
499. Khader F, Kather JN, Muller-Franzes G, et al. Medical transformer for multimodal survival prediction in intensive care: integration of imaging and non-imaging data. *Sci Rep*. 2023;13(1):10666. doi:10.1038/s41598-023-37835-1
500. Khader F, Muller-Franzes G, Wang T, et al. Multimodal Deep Learning for Integrating Chest Radiographs and Clinical Parameters: A Case for Transformers. *Radiology*. 2023;309(1):e230806. doi:10.1148/radiol.230806
501. Khodadadi A, Ghanbari Bousejin N, Molaei S, Kumar Chauhan V, Zhu T, Clifton DA. Improving Diagnostics with Deep Forest Applied to Electronic Health Records. *Sensors (Basel)*. Jul 21 2023;23(14)doi:s23146571 [pii]
sensors-23-06571 [pii]
10.3390/s23146571
502. Khope SR, Elias S. A Novel Integrated Learning Scheme for Predictive Diagnosis of Critical Care Patient. *CMC-Comput Mat Contin*. 2022;73(2):2333-2350. doi:10.32604/cmc.2022.029423
503. Khope SR, Elias S. Critical Correlation of Predictors for an Efficient Risk Prediction Framework of ICU Patient Using Correlation and Transformation of MIMIC-III Dataset. *Data Sci Eng*. 2022;7(1):71-86. doi:10.1007/s41019-022-00176-6
504. Kikutani K, Ohshimo S, Sadamori T, et al. Quantification of respiratory sounds by a continuous monitoring system can be used to predict complications after extubation: a pilot study. *J Clin Monit Comput*. 2023;37(1):237-248. doi:10.1007/s10877-022-00884-4
505. Kim JH, Kwon YS, Baek MS. Machine Learning Models to Predict 30-Day Mortality in Mechanically Ventilated Patients. *J Clin Med*. 2021;10(10)doi:10.3390/jcm10102172
506. Kim JH, Hua M, Whittington RA, et al. A machine learning approach to identifying delirium from electronic health records. *JAMIA open*. 2022;5(2):ooac042. doi:10.1093/jamiaopen/ooac042
507. Kim HB, Nguyen HT, Jin Q, et al. Computational signatures for post-cardiac arrest trajectory prediction: Importance of early physiological time series. *Anaesth Crit Care Pain Med*. 2022;41(1):101015. doi:10.1016/j.accpm.2021.101015
508. Kim MK, Oh J, Kim JJ, Park JY. Development and Validation of Simplified Delirium Prediction Model in Intensive Care Unit. *Front Psychiatr*. 2022;13:886186. doi:10.3389/fpsyt.2022.886186
509. Kim JK, Ahn W, Park S, Lee SH, Kim L. Early Prediction of Sepsis Onset Using Neural Architecture Search Based on Genetic Algorithms. *Int J Environ Res Public Health*. 2022;19(4)doi:10.3390/ijerph19042349
510. Kim T, Tae Y, Yeo HJ, et al. Development and Validation of Deep-Learning-Based Sepsis and Septic Shock Early Prediction System (DeepSEPS) Using Real-World ICU Data. *J Clin Med*. 2023;12(22)doi:10.3390/jcm12227156
511. Kim YK, Koo JH, Lee SJ, Song HS, Lee M. Explainable Artificial Intelligence Warning Model Using an Ensemble Approach for In-Hospital Cardiac Arrest Prediction: Retrospective Cohort Study. *J Med Internet Res*. 2023;25:e48244. doi:10.2196/48244
512. Kim GH, Kim JW, Kim KH, et al. FT-GAT: Graph neural network for predicting spontaneous breathing trial success in patients with mechanical ventilation. *Comput Methods Programs Biomed*. 2023;240:107673. doi:10.1016/j.cmpb.2023.107673
513. Kim J, Kim YK, Kim H, et al. Machine Learning Algorithms Predict Successful Weaning From Mechanical Ventilation Before Intubation: Retrospective Analysis From the Medical Information Mart for Intensive Care IV Database. *JMIR Form Res*. 2023;7:e44763. doi:10.2196/44763
514. Kim D, Choi HS, Lee D, et al. A Deep Learning-Based Approach for Prediction of Vancomycin Treatment Monitoring: Retrospective Study Among Patients With Critical Illness. *JMIR Form Res*. 2024;8:e45202. doi:10.2196/45202
515. Kim M, Kim TH, Kim D, et al. In-Advance Prediction of Pressure Ulcers via Deep-Learning-Based Robust Missing Value Imputation on Real-Time Intensive Care Variables. *J Clin Med*. 2024;13(1)doi:10.3390/jcm13010036
516. King AJ, Angus DC, Cooper GF, et al. A voice-based digital assistant for intelligent prompting of evidence-based practices during ICU rounds. *J Biomed Inform*. 2023;146:104483. doi:10.1016/j.jbi.2023.104483

517. King AJ, Tang L, Davis BS, et al. Machine learning-based prediction of low-value care for hospitalized patients. *Intell Based Med*. 2023;8doi:10.1016/j.ibmed.2023.100115
518. Kline A, Kline T, Shakeri Hossein Abad Z, Lee J. Using Item Response Theory for Explainable Machine Learning in Predicting Mortality in the Intensive Care Unit: Case-Based Approach. *J Med Internet Res*. 2020;22(9):e20268. doi:10.2196/20268
519. Knox DB, Lanspa MJ, Wilson E, et al. Initial Derivation of a Predictive Model for Left Ventricular Longitudinal Strain (LS) in Early Sepsis. *J Intensive Care Med*. 2022;37(8):1049-1054. doi:10.1177/08850666211053796
520. Ko RE, Cho J, Shin MK, et al. Machine Learning-Based Mortality Prediction Model for Critically Ill Cancer Patients Admitted to the Intensive Care Unit (CanICU). *Cancers (Basel)*. 2023;15(3)doi:10.3390/cancers15030569
521. Kobayashi N, Shiga T, Ikumi S, Watanabe K, Murakami H, Yamauchi M. Semi-automated tracking of pain in critical care patients using artificial intelligence: a retrospective observational study. *Sci Rep*. 2021;11(1):5229. doi:10.1038/s41598-021-84714-8
522. Kobayashi N, Watanabe K, Murakami H, Yamauchi M. Continuous visualization and validation of pain in critically ill patients using artificial intelligence: a retrospective observational study. *Sci Rep*. 2023;13(1):17479. doi:10.1038/s41598-023-44970-2
523. Kohn R, Weissman GE, Wang W, et al. Prediction of In-hospital Mortality Among Intensive Care Unit Patients Using Modified Daily Laboratory-based Acute Physiology Score, Version 2. *Med Care*. 2023;61(8):562-569. doi:10.1097/mlr.0000000000001878
524. Kolisnyk M, Kazazian K, Rego K, et al. Predicting neurologic recovery after severe acute brain injury using resting-state networks. *J Neurol*. 2023;270(12):6071-6080. doi:10.1007/s00415-023-11941-6
525. Kong G, Lin K, Hu Y. Using machine learning methods to predict in-hospital mortality of sepsis patients in the ICU. *BMC Med Inf Decis Mak*. 2020;20(1):251. doi:10.1186/s12911-020-01271-2
526. Kramer AA, LaFonte M, El Husseini I, et al. Prospective evaluation of a machine learning-based clinical decision support system (ViSIG) in reducing adverse outcomes for adult critically ill patients. *Inform Med Unlocked*. 2024;44doi:10.1016/j.imu.2023.101433
527. Krieg SM, Schwendner M, Kram L, et al. Transcranial Transmission Ultrasound for Reliable Noninvasive Exclusion of Intracranial Hypertension in Traumatic Brain Injury Patients: A Proof of Concept Study. *J Neurotrauma*. Oct 2024;41(19-20):2298-2306. doi:10.1089/neu.2024.0027
528. Krishnan GS. Evaluating the quality of word representation models for unstructured clinical text based ICU mortality prediction. *Proceedings of the 20th International Conference on …*. 2019;doi:10.1145/3288599.3297118
529. Krishnan P, Rad MG, Agarwal P, et al. HIRA: Heart Rate Interval based Rapid Alert score to characterize autonomic dysfunction among patients with sepsis-related acute respiratory failure (ARF). *Physiol Meas*. Oct 13 2023;44(10)doi:pmeaacf5c7 [pii]
10.1088/1361-6579/acf5c7
530. Kudo D, Goto T, Uchimido R, et al. Coagulation phenotypes in sepsis and effects of recombinant human thrombomodulin: an analysis of three multicentre observational studies. *Crit Care*. 2021;25(1)doi:10.1186/s13054-021-03541-5
531. Kugler S, Hahnefeld L, Kloka JA, et al. Short-term predictor for COVID-19 severity from a longitudinal multi-omics study for practical application in intensive care units. *Talanta*. 2024;268:125295. doi:10.1016/j.talanta.2023.125295
532. Kurtz P, Peres IT, Soares M, Salluh JIF, Bozza FA. Hospital Length of Stay and 30-Day Mortality Prediction in Stroke: A Machine Learning Analysis of 17,000 ICU Admissions in Brazil. *Neurocrit Care*. 2022;37:313-321. doi:10.1007/s12028-022-01486-3
533. Kwak GH, Ling L, Hui P. Predicting the Need For Vasopressors in the Intensive Care Unit Using an Attention Based Deep Learning Model. *Shock*. 2021;56(1):73-79. doi:10.1097/shk.0000000000001692
534. La Cava W, Williams H, Fu W, Vitale S, Srivatsan D, Moore JH. Evaluating recommender systems for AI-driven biomedical informatics. *Bioinformatics*. 2021;37(2):250-256. doi:10.1093/bioinformatics/btaa698
535. Lal A, Li G, Cubro E, et al. Development and Verification of a Digital Twin Patient Model to Predict Specific Treatment Response During the First 24 Hours of Sepsis. *Crit care explor*. 2020;2(11):e0249. doi:10.1097/cce.0000000000000249
536. Lancia G, Varkila MRJ, Cremer OL, Spitoni C. Two-step interpretable modeling of ICU-AIs. *Artif Intell Med*. 2024;151:102862. doi:10.1016/j.artmed.2024.102862

537. Lau S, Shum HP, Chan CCY, et al. Prediction of hospital mortality among critically ill patients in a single centre in Asia: comparison of artificial neural networks and logistic regression-based model. *Hong Kong Med J*. 2024;30(2):130-138. doi:10.12809/hkmj2210235
538. Le S, Pellegrini E, Green-Saxena A, et al. Supervised machine learning for the early prediction of acute respiratory distress syndrome (ARDS). *J Crit Care*. 2020;60:96-102. doi:10.1016/j.jcrc.2020.07.019
539. Le S, Allen A, Calvert J, et al. Convolutional Neural Network Model for Intensive Care Unit Acute Kidney Injury Prediction. *KI Rep*. 2021;6(5):1289-1298. doi:10.1016/j.ekir.2021.02.031
540. Lee GH, Shin SY. Federated Learning on Clinical Benchmark Data: Performance Assessment. *J Med Internet Res*. 2020;22(10):e20891. doi:10.2196/20891
541. Lee BT, Kwon OY, Park H, Cho KJ, Kwon JM, Lee Y. Graph Convolutional Networks-Based Noisy Data Imputation in Electronic Health Record. *Crit Care Med*. 2020;48(11):e1106-e1111. doi:10.1097/ccm.0000000000004583
542. Lee S, Chang D, Song MH, Kim JY. Explainable Machine Learning Based a Packed Red Blood Cell Transfusion Prediction and Evaluation for Major Internal Medical Condition. *J Inf Process Syst*. 2022;18(3):302-310. doi:10.3745/jips.04.0243
543. Lee SW, Kung HC, Huang JF, et al. The Clinical Application of Machine Learning-Based Models for Early Prediction of Hemorrhage in Trauma Intensive Care Units. *J Pers Med*. 2022;12(11)doi:10.3390/jpm12111901
544. Lee H, Yang HL, Ryu HG, et al. Real-time machine learning model to predict in-hospital cardiac arrest using heart rate variability in ICU. *NPJ Digit Med*. 2023;6(1)doi:10.1038/s41746-023-00960-2
545. Lee H, Song MJ, Cho YJ, et al. Supervised machine learning model to predict mortality in patients undergoing venovenous extracorporeal membrane oxygenation from a nationwide multicentre registry. *BMJ Open Respir Res*. Dec 28 2023;10(1)doi:10/1/e002025 [pii]
bmjresp-2023-002025 [pii]
10.1136/bmjresp-2023-002025
546. Lee TH, Kim S, Lee J, Jun CH. HarmoSATE: Harmonized embedding-based self-attentive encoder to improve accuracy of privacy-preserving federated predictive analysis. *Inf Sci*. 2024;662doi:10.1016/j.ins.2024.120265
547. Lei M, Han Z, Wang S, et al. Biological signatures and prediction of an immunosuppressive status-persistent critical illness-among orthopedic trauma patients using machine learning techniques. *Front Immunol*. 2022;13:979877. doi:10.3389/fimmu.2022.979877
548. Lei M, Han Z, Wang S, et al. A machine learning-based prediction model for in-hospital mortality among critically ill patients with hip fracture: An internal and external validated study. *Injury*. 2023;54(2):636-644. doi:10.1016/j.injury.2022.11.031
549. Leitner J, Chiang PH, Dey S. Personalized Blood Pressure Estimation Using Photoplethysmography: A Transfer Learning Approach. *IEEE J Biomedical Health Informat*. 2022;26(1):218-228. doi:10.1109/jbhi.2021.3085526
550. Lemmon J, Guo LL, Posada J, et al. Evaluation of Feature Selection Methods for Preserving Machine Learning Performance in the Presence of Temporal Dataset Shift in Clinical Medicine. *Methods Inf Med*. 2023;62(1):60-70. doi:10.1055/s-0043-1762904
551. Lengerich BJ, Nunnally ME, Aphinyanaphongs Y, Ellington C, Caruana R. Automated interpretable discovery of heterogeneous treatment effectiveness: A COVID-19 case study. *J Biomed Inform*. 2022;130:104086. doi:10.1016/j.jbi.2022.104086
552. Lenivtceva I, Panfilov D, Kopanitsa G, Kozlov B. Aortic Risks Prediction Models after Cardiac Surgeries Using Integrated Data. *J Pers Med*. 2022;12(4)doi:10.3390/jpm12040637
553. Levi R, Carli F, Arevalo AR, et al. Artificial intelligence-based prediction of transfusion in the intensive care unit in patients with gastrointestinal bleeding. *BMJ Health Care Inform*. 2021;28(1)doi:10.1136/bmjhci-2020-100245
554. Li Y, Yao L, Mao C, Srivastava A, Jiang X, Luo Y. Early Prediction of Acute Kidney Injury in Critical Care Setting Using Clinical Notes. *Proceedings (IEEE Int Conf Bioinformatics Biomed)*. 2018;2018:683-686. doi:10.1109/bibm.2018.8621574
555. Li Y, Nair P, Lu XH, et al. Inferring multimodal latent topics from electronic health records. *Nat Commun*. 2020;11(1):2536. doi:10.1038/s41467-020-16378-3
556. Li YH, Harfiya LN, Purw, ari K, Lin YD. Real-Time Cuffless Continuous Blood Pressure Estimation Using Deep Learning Model. *Sensors (Basel)*. 2020;20(19)doi:10.3390/s20195606
557. Li Q, Li LL, Zhong J, Huang LF. Real-time sepsis severity prediction on knowledge graph deep learning networks for the intensive care unit. *J Vis Commun Image Represent*. 2020;72doi:10.1016/j.jvcir.2020.102901

558. Li D, Gao J, Hong N, et al. A Clinical Prediction Model to Predict Heparin Treatment Outcomes and Provide Dosage Recommendations: Development and Validation Study. *J Med Internet Res*. 2021;23(5):e27118. doi:10.2196/27118
559. Li K, Shi Q, Liu S, Xie Y, Liu J. Predicting in-hospital mortality in ICU patients with sepsis using gradient boosting decision tree. *Medicine (Baltimore)*. 2021;100(19):e25813. doi:10.1097/md.0000000000025813
560. Li M, Zhuang Q, Zhao S, et al. Development and deployment of interpretable machine-learning model for predicting in-hospital mortality in elderly patients with acute kidney disease. *Ren Fail*. 2022;44(1):1886-1896. doi:10.1080/0886022x.2022.2142139
561. Li LT, Huang T, Bernstam EV, Jiang X. External Validation of a Laboratory Prediction Algorithm for the Reduction of Unnecessary Labs in the Critical Care Setting. *Am J Med*. 2022;135(6):769-774. doi:10.1016/j.amjmed.2021.12.020
562. Li L, Tu B, Xiong Y, et al. Machine Learning-Based Model for Predicting Prolonged Mechanical Ventilation in Patients with Congestive Heart Failure. *Cardiovasc Drugs Ther*. 2022;doi:10.1007/s10557-022-07399-9
563. Li J, Liu S, Hu Y, Zhu L, Mao Y, Liu J. Predicting Mortality in Intensive Care Unit Patients With Heart Failure Using an Interpretable Machine Learning Model: Retrospective Cohort Study. *J Med Internet Res*. 2022;24(8):e38082. doi:10.2196/38082
564. Li B, Cao J. Classification of coma/brain-death EEG dataset based on one-dimensional convolutional neural network. *Cogn Neurodynamics*. 2023;doi:10.1007/s11571-023-09942-2
565. Li S, Dou R, Song X, et al. Developing an Interpretable Machine Learning Model to Predict in-Hospital Mortality in Sepsis Patients: A Retrospective Temporal Validation Study. *J Clin Med*. 2023;12(3)doi:10.3390/jcm12030915
566. Li Q, Lv H, Chen Y, Shen J, Shi J, Zhou C. Development and Validation of a Machine Learning Predictive Model for Cardiac Surgery-Associated Acute Kidney Injury. *J Clin Med*. 2023;12(3)doi:10.3390/jcm12031166
567. Li L, Ding L, Zhang Z, et al. Development and Validation of Machine Learning-Based Models to Predict In-Hospital Mortality in Life-Threatening Ventricular Arrhythmias: Retrospective Cohort Study. *J Med Internet Res*. 2023;25:e47664. doi:10.2196/47664
568. Li DA, Ma HT, Li WJ, et al. KTI-RNN: Recognition of Heart Failure from Clinical Notes. *Tsinghua Sci Technol*. 2023;28(1):117-130. doi:10.26599/tst.2021.9010093
569. Li X, Wu R, Zhao W, et al. Machine learning algorithm to predict mortality in critically ill patients with sepsis-associated acute kidney injury. *Sci Rep*. 2023;13(1):5223. doi:10.1038/s41598-023-32160-z
570. Li X, Zhu Y, Zhao W, et al. Machine learning algorithm to predict the in-hospital mortality in critically ill patients with chronic kidney disease. *Ren Fail*. 2023;45(1):2212790. doi:10.1080/0886022x.2023.2212790
571. Li J, Xi F, Yu W, Sun C, Wang X. Real-Time Prediction of Sepsis in Critical Trauma Patients: Machine Learning-Based Modeling Study. *JMIR Form Res*. 2023;7:e42452. doi:10.2196/42452
572. Li Q, Li J, Chen J, et al. A machine learning-based prediction model for postoperative delirium in cardiac valve surgery using electronic health records. *BMC Cardiovasc Disord*. 2024;24(1):56. doi:10.1186/s12872-024-03723-3
573. Li C, Liu Y, Dong R, Zhang T, Song Y, Zhang Q. Deep learning radiomics on shear wave elastography and b-mode ultrasound videos of diaphragm for weaning outcome prediction. *Med Eng Phys*. 2024;123:104090. doi:10.1016/j.medengphy.2023.104090
574. Li X, Wang P, Zhu Y, Zhao W, Pan H, Wang D. Interpretable machine learning model for predicting acute kidney injury in critically ill patients. *BMC Med Inf Decis Mak*. 2024;24(1):148. doi:10.1186/s12911-024-02537-9
575. Li X, Wang Z, Zhao W, et al. Machine learning algorithm for predict the in-hospital mortality in critically ill patients with congestive heart failure combined with chronic kidney disease. *Ren Fail*. 2024;46(1):2315298. doi:10.1080/0886022x.2024.2315298
576. Li M, Han S, Liang F, et al. Machine Learning for Predicting Risk and Prognosis of Acute Kidney Disease in Critically Ill Elderly Patients During Hospitalization: Internet-Based and Interpretable Model Study. *J Med Internet Res*. 2024;26:e51354. doi:10.2196/51354
577. Li R, Xu Z, Xu J, et al. Predicting intubation for intensive care units patients: A deep learning approach to improve patient management. *Int J Med Inf*. 2024;186:105425. doi:10.1016/j.ijmedinf.2024.105425
578. Li X, Xu X, Xie F, et al. A Time-Phased Machine Learning Model for Real-Time Prediction of Sepsis in Critical Care. *Crit Care Med*. 2020;48(10):e884-e888. doi:10.1097/ccm.0000000000004494

579. Liang Q, Zhao Q, Xu X, Zhou Y, Huang M. Early prediction of carbapenem-resistant Gram-negative bacterial carriage in intensive care units using machine learning. *J Glob Antimicrob Resist*. 2022;29:225-231. doi:10.1016/j.jgar.2022.03.019
580. Liang Y, Zhu C, Tian C, et al. Early prediction of ventilator-associated pneumonia in critical care patients: a machine learning model. *BMC polm med*. 2022;22(1):250. doi:10.1186/s12890-022-02031-w
581. Liang N, Wang C, Duan J, Xie X, Wang Y. Efficacy prediction of noninvasive ventilation failure based on the stacking ensemble algorithm and autoencoder. *BMC Med Inf Decis Mak*. 2022;22(1):27. doi:10.1186/s12911-022-01767-z
582. Liang Q, Ding S, Chen J, et al. Prediction of carbapenem-resistant gram-negative bacterial bloodstream infection in intensive care unit based on machine learning. *BMC Med Inf Decis Mak*. 2024;24(1):123. doi:10.1186/s12911-024-02504-4
583. Liang Q, Xu X, Ding S, Wu J, Huang M. Prediction of successful weaning from renal replacement therapy in critically ill patients based on machine learning. *Ren Fail*. 2024;46(1):2319329. doi:10.1080/0886022x.2024.2319329
584. Liao KM, Ko SC, Liu CF, et al. Development of an Interactive AI System for the Optimal Timing Prediction of Successful Weaning from Mechanical Ventilation for Patients in Respiratory Care Centers. *Diagn*. 2022;12(4)doi:10.3390/diagnostics12040975
585. Liao W, Voldman J. A Multidatabase ExTRaction PipEline (METRE) for facile cross validation in critical care research. *J Biomed Inform*. 2023;141:104356. doi:10.1016/j.jbi.2023.104356
586. Lichtner G, Balzer F, Haufe S, et al. Predicting lethal courses in critically ill COVID-19 patients using a machine learning model trained on patients with non-COVID-19 viral pneumonia. *Sci Rep*. 2021;11(1):13205. doi:10.1038/s41598-021-92475-7
587. Liliopoulos SG, Dejaco A, Paseiro-Garcia L, Dimakopoulos VS, Gkouzionis IA. Development and Validation of the VIOSync Sepsis Prediction Index: A Novel Machine Learning Model for Sepsis Prediction in ICU Patients. 2024;doi:10.1101/2024.02.22.24303211
588. Lilly CM, Kirk D, Pessach IM, et al. Application of Machine Learning Models to Biomedical and Information System Signals From Critically Ill Adults. *Chest*. May 2024;165(5):1139-1148. doi:S0012-3692(23)05682-9 [pii]
10.1016/j.chest.2023.10.036
589. Lim L, Gim U, Cho K, Yoo D, Ryu HG, Lee HC. Real-time machine learning model to predict short-term mortality in critically ill patients: development and international validation. *Crit Care*. 2024;28(1):76. doi:10.1186/s13054-024-04866-7
590. Lin MY, Li CC, Lin PH, et al. Explainable Machine Learning to Predict Successful Weaning Among Patients Requiring Prolonged Mechanical Ventilation: A Retrospective Cohort Study in Central Taiwan. *Front Med*. 2021;8doi:10.3389/fmed.2021.663739
591. Lin T, Zhang X, Gong J, et al. A dosing strategy model of deep deterministic policy gradient algorithm for sepsis patients. *BMC Med Inf Decis Mak*. 2023;23(1):81. doi:10.1186/s12911-023-02175-7
592. Lin CH, Liu CL. Prediction of Blood Glucose Concentration Based on OptiScanner and XGBoost in ICU. *IEEE Access*. 2023;11:116524-116533. doi:10.1109/access.2023.3325430
593. Lin S, Yang M, Liu C, Wang Z, Long X. A pretrain-finetune approach for improving model generalizability in outcome prediction of acute respiratory distress syndrome patients. *Int J Med Inf*. 2024;186:105397. doi:10.1016/j.ijmedinf.2024.105397
594. Lin J, Yang J, Yin M, et al. Development and Validation of Multimodal Models to Predict the 30-Day Mortality of ICU Patients Based on Clinical Parameters and Chest X-Rays. *J Imaging Inform Med*. Aug 2024;37(4):1312-1322. doi:10.1007/s10278-024-01066-1 [pii]
1066 [pii]
10.1007/s10278-024-01066-1
595. Lin L, Ding L, Fu Z, Zhang L. Machine learning-based models for prediction of the risk of stroke in coronary artery disease patients receiving coronary revascularization. *PLoS One*. 2024;19(2):e0296402. doi:10.1371/journal.pone.0296402
596. Lin S, Lu W, Wang T, et al. Predictive model of acute kidney injury in critically ill patients with acute pancreatitis: a machine learning approach using the MIMIC-IV database. *Ren Fail*. 2024;46(1):2303395. doi:10.1080/0886022x.2024.2303395
597. Lin YH, Chang TC, Liu CF, Lai CC, Chen CM, Chou W. The intervention of artificial intelligence to improve the weaning outcomes of patients with mechanical ventilation: Practical applications in the medical intensive care unit and the COVID-19 intensive care unit: A retrospective study. *Medicine (Baltimore)*. 2024;103(12):e37500. doi:10.1097/md.0000000000037500

598. Ling J, Liu H, Yu D, Wang Z, Fang M. Three subtypes of postoperative ARDS that showing different outcomes and responses to mechanical ventilation and fluid management: A machine learning and latent profile analysis. *Heart Lung*. 2023;62:135-144. doi:10.1016/j.hrtlng.2023.07.007
599. Lintu MK, Micheal DR, Kamath A. Mortality prediction on unsupervised and semi-supervised clusters of medical intensive care unit patients based on MIMIC-II database. *Inform Med Unlocked*. 2023;39doi:10.1016/j.imu.2023.101264
600. Liu D, Miller T, Sayeed R, Mandl KD. FADL: Federated-autonomous deep learning for distributed electronic health record. *arXiv preprint arXiv:1811 11400*. 2018;doi:
601. Liu M, Guo C, Cui S. A Deep Learning Post-Discharge Mortality Prediction Method Considering Diagnosis Information for ICU Patients. 2021;doi:10.21203/rs.3.rs-1027700/v1
602. Liu W, Tao G, Zhang Y, et al. A Simple Weaning Model Based on Interpretable Machine Learning Algorithm for Patients With Sepsis: A Research of MIMIC-IV and eICU Databases. *Front Med (Lausanne)*. 2021;8:814566. doi:10.3389/fmed.2021.814566
603. Liu Z, Khoj, i A, et al. HeMA: A hierarchically enriched machine learning approach for managing false alarms in real time: A sepsis prediction case study. *Comput Biol Med*. 2021;131:104255. doi:10.1016/j.compbiomed.2021.104255
604. Liu C, Liu X, Mao Z, et al. Interpretable Machine Learning Model for Early Prediction of Mortality in ICU Patients with Rhabdomyolysis. *Med Sci Sports Exerc*. 2021;53(9):1826-1834. doi:10.1249/mss.0000000000002674
605. Liu J, Wu J, Liu S, Li M, Hu K, Li K. Predicting mortality of patients with acute kidney injury in the ICU using XGBoost model. *PLoS One*. 2021;16(2):e0246306. doi:10.1371/journal.pone.0246306
606. Liu X, Liu T, Zhang Z, et al. TOP-Net Prediction Model Using Bidirectional Long Short-term Memory and Medical-Grade Wearable Multisensor System for Tachycardia Onset: Algorithm Development Study. *JMIR Med Inform*. 2021;9(4):e18803. doi:10.2196/18803
607. Liu D, Zheng M, Sepulveda NA. Using Artificial Neural Network Condensation to Facilitate Adaptation of Machine Learning in Medical Settings by Reducing Computational Burden: Model Design and Evaluation Study. *JMIR Form Res*. 2021;5(12):e20767. doi:10.2196/20767
608. Liu G, Xu J, Wang C, et al. A machine learning method for predicting the probability of MODS using only non-invasive parameters. *Comput Methods Programs Biomed*. 2022;227:107236. doi:10.1016/j.cmpb.2022.107236
609. Liu Z, Yang Y, Song H, Luo J. A prediction model with measured sentiment scores for the risk of in-hospital mortality in acute pancreatitis: a retrospective cohort study. *Ann transl med*. 2022;10(12):676. doi:10.21037/atm-22-1613
610. Liu Y, Gao K, Deng H, et al. A time-incorporated SOFA score-based machine learning model for predicting mortality in critically ill patients: A multicenter, real-world study. *Int J Med Inf*. 2022;163:104776. doi:10.1016/j.ijmedinf.2022.104776
611. Liu CF, Hung CM, Ko SC, et al. An artificial intelligence system to predict the optimal timing for mechanical ventilation weaning for intensive care unit patients: A two-stage prediction approach. *Front Med (Lausanne)*. 2022;9:935366. doi:10.3389/fmed.2022.935366
612. Liu M, Guo C, Guo S. An explainable knowledge distillation method with XGBoost for ICU mortality prediction. *Comput Biol Med*. 2023;152:106466. doi:10.1016/j.compbiomed.2022.106466
613. Liu W, Ma W, Bai N, et al. Identification of key predictors of hospital mortality in critically ill patients with embolic stroke using machine learning. *Biosci Rep*. 2022;42(9)doi:10.1042/bsr20220995
614. Liu WT, Liu XQ, Jiang TT, et al. Using a machine learning model to predict the development of acute kidney injury in patients with heart failure. *Front cardiovasc med*. 2022;9:911987. doi:10.3389/fcvm.2022.911987
615. Liu X, DuMontier C, Hu P, et al. Clinically Interpretable Machine Learning Models for Early Prediction of Mortality in Older Patients with Multiple Organ Dysfunction Syndrome: An International Multicenter Retrospective Study. *J Gerontol A Biol Sci Med Sci*. 2023;78(4):718-726. doi:10.1093/gerona/glac107
616. Liu F, Yao J, Liu C, Shou S. Construction and validation of machine learning models for sepsis prediction in patients with acute pancreatitis. *BMC Surg*. 2023;23(1):267. doi:10.1186/s12893-023-02151-y
617. Liu C, Yao Z, Liu P, et al. Early prediction of MODS interventions in the intensive care unit using machine learning. *J Big Data*. 2023;10(1):55. doi:10.1186/s40537-023-00719-2
618. Liu RQ, Hunold KM, Caterino JM, Zhang P. Estimating treatment effects for time-to-treatment antibiotic stewardship in sepsis. *Nat Mach Intell*. 2023;5(4):421-431. doi:10.1038/s42256-023-00638-0
619. Liu HH, Wang YT, Yang MH, Lin WSK, Oyang YJ. Exploiting Machine Learning Technologies to Study the Compound Effects of Serum Creatinine and Electrolytes on the Risk of Acute Kidney Injury in Intensive Care Units. *Diagn*. 2023;13(15)doi:10.3390/diagnostics13152551

620. Liu X, Hu P, Yeung W, et al. Illness severity assessment of older adults in critical illness using machine learning (ELDER-ICU): an international multicentre study with subgroup bias evaluation. *Lancet Digit Health*. 2023;5(10):e657-e667. doi:10.1016/s2589-7500(23)00128-0
621. Liu C, Elmer J, Arefan D, Pease M, Wu S. Interpretable machine learning model for imaging-based outcome prediction after cardiac arrest. *Resuscitation*. 2023;191doi:10.1016/j.resuscitation.2023.109894
622. Liu S, Schlesinger JJ, McCoy AB, et al. New onset delirium prediction using machine learning and long short-Term memory (LSTM) in electronic health record. *J Am Med Informatics Assoc*. 2023;30(1):120-131. doi:10.1093/jamia/ocac210
623. Liu Z, Hu Y, Wu X, Mertes G, Yang Y, Clifton DA. Patient Clustering for Vital Organ Failure Using ICD Code with Graph Attention. *IEEE Trans Biomed Eng*. 2023;doi:10.1109/tbme.2023.3243311
624. Liu P, Li S, Zheng T, et al. Subphenotyping heterogeneous patients with chronic critical illness to guide individualised fluid balance treatment using machine learning: a retrospective cohort study. *EClinicalMedicine*. 2023;59:101970. doi:10.1016/j.eclinm.2023.101970
625. Liu S, Huang Z, Zhu J, Liu B, Zhou P. Continuous blood pressure monitoring using photoplethysmography and electrocardiogram signals by random forest feature selection and GWO-GBRT prediction model. *Biomed Signal Process Control*. 2024;88doi:10.1016/j.bspc.2023.105354
626. Liu Z, Wu X, Yang Y, Clifton DA. DuKA: A Dual-Keyless-Attention Model for Multi-Modality EHR Data Fusion and Organ Failure Prediction. *IEEE Trans Biomed Eng*. 2024;71(4):1247-1256. doi:10.1109/tbme.2023.3331305
627. Liu X, Niu H, Peng J. Enhancing predictions with a stacking ensemble model for ICU mortality risk in patients with sepsis-associated encephalopathy. *J Int Med Res*. 2024;52(3):3000605241239013. doi:10.1177/03000605241239013
628. Liu X, Niu H, Peng J. Improving predictions: Enhancing in-hospital mortality forecast for ICU patients with sepsis-induced coagulopathy using a stacking ensemble model. *Medicine (Baltimore)*. 2024;103(14):e37634. doi:10.1097/md.0000000000037634
629. Liu JP, Wu XQ, Xie YM, Tang ZH, Xie YF, Gong SB. Small samples-oriented intrinsically explainable machine learning using Variational Bayesian Logistic Regression: An intensive care unit readmission prediction case for liver transplantation patients. *Expert Syst Appl*. 2024;235doi:10.1016/j.eswa.2023.121138
630. Liu J, Xie Y, Shu X, et al. Value function assessment to different RL algorithms for heparin treatment policy of patients with sepsis in ICU. *Artif Intell Med*. 2024;147:102726. doi:10.1016/j.artmed.2023.102726
631. Lombardi S, Partanen P, Francia P, et al. Classifying sepsis from photoplethysmography. *Health Inf Sci Syst*. 2022;10(1):30. doi:10.1007/s13755-022-00199-3
632. Long W, Wang X. BPNet: A multi-modal fusion neural network for blood pressure estimation using ECG and PPG. *Biomed Signal Process Control*. 2023;86doi:10.1016/j.bspc.2023.105287
633. Loo NL, Chiew YS, Tan CP, Mat-Nor MB, Ralib AM. A machine learning approach to assess magnitude of asynchrony breathing. *Biomed Signal Process Control*. 2021;66doi:10.1016/j.bspc.2021.102505
634. Lorenzoni G, Sella N, Boscolo A, et al. COVID-19 ICU mortality prediction: a machine learning approach using SuperLearner algorithm. *J Anesth Analg Crit Care*. 2021;1(1):3. doi:10.1186/s44158-021-00002-x
635. Loutati R, Perel N, Marmor D, et al. Artificial intelligence based prediction model of in-hospital mortality among females with acute coronary syndrome: for the Jerusalem Platelets Thrombosis and Intervention in Cardiology (JUPITER-12) Study Group. *Front cardiovasc med*. 2024;11:1333252. doi:10.3389/fcvm.2024.1333252
636. Lozano Gomez H, Rodriguez Garcia A, Rodriguez Esteban MA, et al. Design of a new mortality indicator in acute coronary syndrome on admission to the Intensive Care Unit. *Med Intensiva (Engl Ed)*. 2023;doi:10.1016/j.medine.2023.03.008
637. Lu Z, Zhang J, Hong J, et al. Development of a Nomogram to Predict 28-Day Mortality of Patients With Sepsis-Induced Coagulopathy: An Analysis of the MIMIC-III Database. *Front Med (Lausanne)*. 2021;8:661710. doi:10.3389/fmed.2021.661710
638. Lu M, Shahn Z, Sow D, Doshi-Velez F, Lehman LH. Is Deep Reinforcement Learning Ready for Practical Applications in Healthcare? A Sensitivity Analysis of Duel-DDQN for Hemodynamic Management in Sepsis Patients. *AMIA Annu Symp Proc*. 2020;2020:773-782. doi:
639. Lu X, Kang H, Zhou D, Li Q. Prediction and risk assessment of sepsis-associated encephalopathy in ICU based on interpretable machine learning. *Sci Rep*. 2022;12(1):22621. doi:10.1038/s41598-022-27134-6

640. Lu Y, Chen Q, Zhang H, et al. Machine Learning Models of Postoperative Atrial Fibrillation Prediction After Cardiac Surgery. *J Cardiothorac Vasc Anesth*. 2023;37(3):360-366. doi:10.1053/j.jvca.2022.11.025
641. Lu X, Chen Y, Zhang G, Zeng X, Lai L, Qu C. Application of interpretable machine learning algorithms to predict acute kidney injury in patients with cerebral infarction in ICU. *J Stroke Cerebrovasc Dis*. 2024;33(7):107729. doi:10.1016/j.jstrokecerebrovasdis.2024.107729
642. Lucini FR, Fiest KM, Stelfox HT, Lee J. Delirium prediction in the intensive care unit: a temporal approach. *Annu Int Conf IEEE Eng Med Biol Soc*. 2020;2020:5527-5530. doi:10.1109/embc44109.2020.9176042
643. Lucini FR, Stelfox HT, Lee J. Deep Learning-Based Recurrent Delirium Prediction in Critically Ill Patients. *Crit Care Med*. 2023;51(4):492-502. doi:10.1097/ccm.0000000000005789
644. Luethi N, Wermelinger SD, Haynes AG, et al. Development of an electronic Poor Outcome Screening (ePOS) Score to identify critically ill patients with potential palliative care needs. *J Crit Care*. 2022;69doi:10.1016/j.jcrc.2022.154007
645. Luo Y, Xin Y, Joshi R, Celi L, Szolovits P. Predicting ICU mortality risk by grouping temporal trends from a multivariate panel of physiologic measurements. *… on Artificial Intelligence*. 2016;doi:
646. Luo L, Kou R, Feng Y, Xiang J, Zhu W. Cost-Effective Machine Learning Based Clinical Pre-Test Probability Strategy for DVT Diagnosis in Neurological Intensive Care Unit. *Clin Appl Thromb Hemost*. 2021;27:10760296211008650. doi:10.1177/10760296211008650
647. Luo Y, Wang Z, Wang C. Improvement of APACHE II score system for disease severity based on XGBoost algorithm. *BMC Med Inf Decis Mak*. 2021;21(1):237. doi:10.1186/s12911-021-01591-x
648. Luo XQ, Yan P, Zhang NY, et al. Machine learning for early discrimination between transient and persistent acute kidney injury in critically ill patients with sepsis. *Sci Rep*. 2021;11(1):20269. doi:10.1038/s41598-021-99840-6
649. Luo C, Zhu Y, Zhu Z, Li R, Chen G, Wang Z. A machine learning-based risk stratification tool for in-hospital mortality of intensive care unit patients with heart failure. *J Transl Med*. 2022;20(1):136. doi:10.1186/s12967-022-03340-8
650. Luo XQ, Yan P, Duan SB, et al. Development and Validation of Machine Learning Models for Real-Time Mortality Prediction in Critically Ill Patients With Sepsis-Associated Acute Kidney Injury. *Front Med (Lausanne)*. 2022;9:853102. doi:10.3389/fmed.2022.853102
651. Luo Y, Ye W, Sun Y, Bao H, Liu H. Development and Comparative Analysis of an Early Prediction Model for Acute Kidney Injury within 72-Hours Post-ICU Admission Using Evidence from the MIMIC-III Database. *Discov medicin*. 2023;35(177):623-631. doi:10.24976/Discov.Med.202335177.61
652. Luo JC, Wang H, Tong SQ, et al. Interpreting Infrared Thermography with Deep Learning to Assess the Mortality Risk of Critically Ill Patients at Risk of Hypoperfusion. *Rev Cardiovasc Med*. 2023;24(1)doi:10.31083/j.rcm2401007
653. Lyra S, Mayer L, Ou LY, et al. A Deep Learning-Based Camera Approach for Vital Sign Monitoring Using Thermography Images for ICU Patients. *Sensors*. 2021;21(4)doi:10.3390/s21041495
654. Lyu X, Fan B, Hüser M, et al. An Empirical Study on KDIGO-Defined Acute Kidney Injury Prediction in the Intensive Care Unit. *medRxiv*. 2024:2024.02.01.24302063. doi:10.1101/2024.02.01.24302063
655. Ma P, Liu J, Shen F, et al. Individualized resuscitation strategy for septic shock formalized by finite mixture modeling and dynamic treatment regimen. *Crit Care*. 2021;25(1):243. doi:10.1186/s13054-021-03682-7
656. Ma CY, Sun GR, Yang XW, Yang S. A clinically applicable prediction model for the risk of in-hospital mortality in solid cancer patients admitted to intensive care units with sepsis. *J Cancer Res Clin Oncol*. 2023;doi:10.1007/s00432-023-04661-x
657. Ma X, Wang M, Lin S, Zhang Y, Ouyang W, Liu X. Knowledge and data-driven prediction of organ failure in critical care patients. *Health Inf Sci Syst*. 2023;11(1):7. doi:10.1007/s13755-023-00210-5
658. Ma M, Sun P, Li Y, Huo W. Predicting the risk of mortality in ICU patients based on dynamic graph attention network of patient similarity. *Math Biosci Eng*. 2023;20(8):15326-15344. doi:10.3934/mbe.2023685
659. Maddali MV, Churpek M, Pham T, et al. Validation and utility of ARDS subphenotypes identified by machine-learning models using clinical data: an observational, multicohort, retrospective analysis. *Lancet Respir Med*. 2022;10(4):367-377. doi:10.1016/s2213-2600(21)00461-6
660. Madden MG, McNicholas BA, Laffey JG. Assessing the usefulness of a large language model to query and summarize unstructured medical notes in intensive care. *Intensive Care Med*. 2023;49(8):1018-1020. doi:10.1007/s00134-023-07128-2

661. Magunia H, Lederer S, Verbuecheln R, et al. Machine learning identifies ICU outcome predictors in a multicenter COVID-19 cohort. *Crit Care*. 2021;25(1):295. doi:10.1186/s13054-021-03720-4
662. Mahardika T NQ, Fuadah YN, Jeong DU, Lim KM. PPG Signals-Based Blood-Pressure Estimation Using Grid Search in Hyperparameter Optimization of CNN–LSTM. *Diagn*. 2023;13(15)doi:10.3390/diagnostics13152566
663. Mahbub M, Srinivasan S, Danciu I, et al. Unstructured clinical notes within the 24 hours since admission predict short, mid & long-term mortality in adult ICU patients. *PLoS One*. 2022;17(1):e0262182. doi:10.1371/journal.pone.0262182
664. Mahendra M, Luo Y, Mills H, Schenk G, Butte AJ, Dudley RA. Impact of Different Approaches to Preparing Notes for Analysis With Natural Language Processing on the Performance of Prediction Models in Intensive Care. *Crit care explor*. 2021;3(6):e0450. doi:10.1097/cce.0000000000000450
665. Maheshwarappa HM, Mishra S, Kulkarni AV, Gunaseelan V, Kanchi M. Use of Handheld Ultrasound Device with Artificial Intelligence for Evaluation of Cardiorespiratory System in COVID-19. *Indian J Crit Care Med*. 2021;25(5):524-527. doi:10.5005/jp-journals-10071-23803
666. Mahmud TI, Imran SA, Shahnaz C. Res-SE-ConvNet: A Deep Neural Network for Hypoxemia Severity Prediction for Hospital In-Patients Using Photoplethysmograph Signal. *IEEE J Transl Eng Health Med*. 2022;10doi:10.1109/jtehm.2022.3217428
667. Mahmud S, Ibtehaz N, Kh, et al. NABNet: A Nested Attention-guided BiConvLSTM network for a robust prediction of Blood Pressure components from reconstructed Arterial Blood Pressure waveforms using PPG and ECG signals. *Biomed Signal Process Control*. 2023;79doi:10.1016/j.bspc.2022.104247
668. Maiello L, Ball L, Micali M, et al. Automatic Lung Segmentation and Quantification of Aeration in Computed Tomography of the Chest Using 3D Transfer Learning. *Front Physiol*. 2021;12:725865. doi:10.3389/fphys.2021.725865
669. Majhi B, Kashyap A. DEVELOPMENT AND ASSESSMENT OF DIFFERENT BALANCING TECHNIQUES AND DEEP LEARNING BASED EARLY MORTALITY PREDICTION MODELS FOR ICU IMBALANCE DATA. *Int J Pharm Sci Res*. 2023;14(8):4170-4192. doi:10.13040/ijpsr.0975-8232.14(8).4170-92
670. Majhi B, Kashyap A. Wavelet based ensemble models for early mortality prediction using imbalance ICU big data. *Smart Health*. 2023;28doi:10.1016/j.smhl.2023.100374
671. Malayeri AB, Khodabakhshi MB. Concatenated convolutional neural network model for cuffless blood pressure estimation using fuzzy recurrence properties of photoplethysmogram signals. *Sci Rep*. 2022;12(1)doi:10.1038/s41598-022-10244-6
672. Mamandipoor B, Frutos-Vivar F, Penuelas O, et al. Machine learning predicts mortality based on analysis of ventilation parameters of critically ill patients: multi-centre validation. *BMC Med Inf Decis Mak*. 2021;21(1):152. doi:10.1186/s12911-021-01506-w
673. Mamandipoor B, Yeung W, Agha-Mir-Salim L, Stone DJ, Osmani V, Celi LA. Prediction of blood lactate values in critically ill patients: a retrospective multi-center cohort study. *J Clin Monit Comput*. 2022;36(4):1087-1097. doi:10.1007/s10877-021-00739-4
674. Mandel C, Stich K, Autexier S, et al. Using Gated Recurrent Unit Networks for the Prediction of Hemodynamic and Pulmonary Decompensation. *Annu Int Conf IEEE Eng Med Biol Soc*. 2022;2022:4584-4589. doi:10.1109/embc48229.2022.9871500
675. Manni F, Bukharev A, Jain A, Moorthy S, Rahman A, Bucur A. Neural gradient boosting in federated learning for hemodynamic instability prediction: towards a distributed and scalable deep learning-based solution. *AMIA Annu Symp Proc*. 2022;2022:729-738. doi:
676. Mansour A, Fuhrman JD, Ammar FE, et al. Machine Learning for Early Detection of Hypoxic-Ischemic Brain Injury After Cardiac Arrest. *Neurocrit Care*. 2022;36(3):974-982. doi:10.1007/s12028-021-01405-y
677. Mansouri A, Noei M, Abadeh MS. A hybrid machine learning approach for early mortality prediction of ICU patients. *Prog Artif Intell*. 2022;11(4):333-347. doi:10.1007/s13748-022-00288-0
678. Mantena S, Arevalo AR, Maley JH, et al. Predicting hypoglycemia in critically Ill patients using machine learning and electronic health records. *J Clin Monit Comput*. 2022;36(5):1297-1303. doi:10.1007/s10877-021-00760-7
679. Mao LK, Huang MH, Lai CH, Sun YN, Chen CY. Detecting Endotracheal Tube and Carina on Portable Supine Chest Radiographs Using One-Stage Detector with a Coarse-to-Fine Attention. *Diagnostics*. 2022;12(8)doi:10.3390/diagnostics12081913
680. Mao B, Zhang R, Pan Y, et al. Machine learning for the prediction of in-hospital mortality in patients with spontaneous intracerebral hemorrhage. *medRxiv*. 2023:2023.08.15.23294147. doi:10.1101/2023.08.15.23294147

681. Maray I, Rodriguez-Ferreras A, Alvarez-Asteinza C, et al. Linezolid induced thrombocytopenia in critically ill patients: Risk factors and development of a machine learning-based prediction model. *J Infect Chemother*. 2022;28(9):1249-1254. doi:10.1016/j.jiac.2022.05.004
682. Martinez N, Bertran M, Sapiro G. Minimax Pareto Fairness: A Multi Objective Perspective. *Proc Mach Learn Res*. 2020;119:6755-6764. doi:
683. Martinez G, Garduno A, Mahmud-Al-Rafat A, et al. An artificial neural network classification method employing longitudinally monitored immune biomarkers to predict the clinical outcome of critically ill COVID-19 patients. *PeerJ*. 2022;10doi:10.7717/peerj.14487
684. Martinez J, Nowroozilarki Z, Jafari R, Mortazavi BJ. Data-Driven Guided Attention for Analysis of Physiological Waveforms with Deep Learning. *IEEE J Biomedical Health Informat*. 2022;26(11):5482-5493. doi:10.1109/jbhi.2022.3199199
685. Martinez GS, Ostadgavahi AT, Al-Rafat AM, et al. Model-interpreted outcomes of artificial neural networks classifying immune biomarkers associated with severe infections in ICU. *Front Immunol*. 2023;14:1137850. doi:10.3389/fimmu.2023.1137850
686. Martinez-Aguero S, Soguero-Ruiz C, Alonso-Moral JM, Mora-Jimenez I, Alvarez-Rodriguez J, Marques AG. Interpretable clinical time-series modeling with intelligent feature selection for early prediction of antimicrobial multidrug resistance. *Futur Gener Comp Syst*. 2022;133:68-83. doi:10.1016/j.future.2022.02.021
687. Mashraqi A, Halawani H, Alelyani T, et al. Prediction Model of Adverse Effects on Liver Functions of COVID-19 ICU Patients. *J Healthc Eng*. 2022;2022:4584965. doi:10.1155/2022/4584965
688. Mataczynski C, Kazimierska A, Uryga A, Burzynska M, Rusiecki A, Kasprowicz M. End-to-End Automatic Morphological Classification of Intracranial Pressure Pulse Waveforms Using Deep Learning. *IEEE J Biomed Health Inform*. 2022;26(2):494-504. doi:10.1109/jbhi.2021.3088629
689. Mathis MR, Engoren MC, Williams AM, et al. Prediction of Postoperative Deterioration in Cardiac Surgery Patients Using Electronic Health Record and Physiologic Waveform Data. *Anesthesiology*. 2022;137(5):586-601. doi:10.1097/aln.0000000000004345
690. Mattia GM, Sarton B, Villain E, et al. Multimodal MRI-Based Whole-Brain Assessment in Patients In Anoxoischemic Coma by Using 3D Convolutional Neural Networks. *Neurocrit Care*. 2022;37:303-312. doi:10.1007/s12028-022-01525-z
691. Maviglia R, Michi T, Passaro D, et al. Machine Learning and Antibiotic Management. *Antibiotics (Basel)*. 2022;11(3)doi:10.3390/antibiotics11030304
692. Mbous YPV, Brothers T, Al-Mamun MA. Medication Regimen Complexity Index Score at Admission as a Predictor of Inpatient Outcomes: A Machine Learning Approach. *Int J Environ Res Public Health*. 2023;20(4)doi:10.3390/ijerph20043760
693. Mears J, Kaleem S, Panchamia R, et al. Leveraging the Capabilities of AI: Novice Neurology-Trained Operators Performing Cardiac POCUS in Patients with Acute Brain Injury. *Neurocrit Care*. Oct 2024;41(2):523-532. doi:10.1007/s12028-024-01953-z [pii]
10.1007/s12028-024-01953-z
694. Mejía-Mejía E, May JM, Kyriacou PA, Elgendi M. Classification of blood pressure in critically ill patients using photoplethysmography and machine learning. *Comput Methods Programs Biomed*. 2021;208doi:10.1016/j.cmpb.2021.106222
695. Menguy J, De Longeaux K, Bodenes L, Hourmant B, L'Her E. Defining predictors for successful mechanical ventilation weaning, using a data-mining process and artificial intelligence. *Sci Rep*. 2023;13(1):20483. doi:10.1038/s41598-023-47452-7
696. Mercier JA, Ferguson TW, Tangri N. A Machine Learning Model to Predict Diuretic Resistance. *Kidney360*. 2023;4(1):15-22. doi:10.34067/kid.0005562022
697. Miao J, Zuo C, Cao H, et al. Predicting ICU readmission risks in intracerebral hemorrhage patients: Insights from machine learning models using MIMIC databases. *J Neurol Sci*. 2024;456:122849. doi:10.1016/j.jns.2023.122849
698. Michalak AJ, Mendiratta A, Eliseyev A, et al. Frontotemporal EEG to guide sedation in COVID-19 related acute respiratory distress syndrome. *Clin Neurophysiol*. 2021;132(3):730-736. doi:10.1016/j.clinph.2021.01.003
699. Mirtchouk M, Srikishan B, Kleinberg S. Hierarchical Information Criterion for Variable Abstraction. *Proc Mach Learn Res*. 2021;149:440-460. doi:
700. Mirzakhani F, Sadoughi F, Hatami M, Amirabadizadeh A. Which model is superior in predicting ICU survival: artificial intelligence versus conventional approaches. *BMC Med Inf Decis Mak*. 2022;22(1):167. doi:10.1186/s12911-022-01903-9
701. Mitra A, Ahsan H, Li W, et al. Risk Factors Associated With Nonfatal Opioid Overdose Leading to Intensive Care Unit Admission: A Cross-sectional Study. *JMIR Med Inform*. 2021;9(11):e32851. doi:10.2196/32851

702. Mo J, Ling S, Yang M, Qin H. Risk of Acute Respiratory Distress Syndrome in Community-Acquired Pneumonia Patients: Use of an Artificial Neural Network Model. *Emerg Med Int*. 2023;2023:2631779. doi:10.1155/2023/2631779
703. Moghadam MC, Masoumi E, Bagherzadeh N, Ramsingh D, Kain ZN. Supervised Machine-Learning Algorithms in Real-time Prediction of Hypotensive Events. *Annu Int Conf IEEE Eng Med Biol Soc*. 2020;2020:5468-5471. doi:10.1109/embc44109.2020.9175451
704. Moghadam MC, Masoumi E, Kendale S, Bagherzadeh N. Predicting hypotension in the ICU using noninvasive physiological signals. *Comput Biol Med*. 2021;129:104120. doi:10.1016/j.compbiomed.2020.104120
705. Moghadam MC, Masoumi E, Kendale S, Bagherzadeh N. Predicting hypotensive events in the ICU settings using patient's short-term physiological history and contextual data. *Comput Methods Programs Biomed Update*. 2023;3doi:10.1016/j.cmpbup.2023.100100
706. Mohammed A, Van Wyk F, Chinthala LK, et al. Temporal Differential Expression of Physiomarkers Predicts Sepsis in Critically Ill Adults. *Shock*. 2021;56(1):58-64. doi:10.1097/shk.0000000000001670
707. Mohedano-Munoz MA, Soguero-Ruiz C, Mora-Jimenez I, Rubio-Sanchez M, Alvarez-Rodriguez J, Sanchez A. A streaming data visualization framework for supporting decision-making in the Intensive Care Unit. *Expert Syst Appl*. 2023;227doi:10.1016/j.eswa.2023.120252
708. Mollura M, Romano S, Mantoan G, Lehman LW, Barbieri R. Prediction of Septic Shock Onset in ICU by Instantaneous Monitoring of Vital Signs. *Annu Int Conf IEEE Eng Med Biol Soc*. 2020;2020:2768-2771. doi:10.1109/embc44109.2020.9176276
709. Mollura M, Lehman LH, Mark RG, Barbieri R. A novel artificial intelligence based intensive care unit monitoring system: using physiological waveforms to identify sepsis. *Philos Transact Ser A Math Phys Eng Sci*. 2021;379(2212):20200252. doi:10.1098/rsta.2020.0252
710. Mollura M, Drudi C, Lehman LW, Barbieri R. A Reinforcement Learning Application for Optimal Fluid and Vasopressor Interventions in Septic ICU Patients. *Annu Int Conf IEEE Eng Med Biol Soc*. 2022;2022:321-324. doi:10.1109/embc48229.2022.9871055
711. Mollura M, Chicco D, Paglialonga A, Barbieri R. Identifying prognostic factors for survival in intensive care unit patients with SIRS or sepsis by machine learning analysis on electronic health records. *PLOS Digit Health*. 2024;3(3):e0000459. doi:10.1371/journal.pdig.0000459
712. Momo LNW, Moorosi N, Nsoesie EO, Rademakers F, De Moor B. Length of stay prediction for hospital management using domain adaptation. *Eng Appl Artif Intell*. 2024;133doi:10.1016/j.engappai.2024.108088
713. Montazeri M, Ahmadinejad M, Bahaadinbeigy K, Ahmadian L. A Rule Based Intelligent Software to Predict Length of Stay and the Mortality Rate in Trauma Patients in the Intensive Care Unit. *Iran J Public Health*. 2023;52(1):175-183. doi:10.18502/ijph.v52i1.11680
714. Montomoli J, Romeo L, Moccia S, et al. Machine learning using the extreme gradient boosting (XGBoost) algorithm predicts 5-day delta of SOFA score at ICU admission in COVID-19 patients. *J Intensive Med*. 2021;1(2):110-116. doi:10.1016/j.jointm.2021.09.002
715. Mora-Jimenez I, Tarancon-Rey J, Alvarez-Rodriguez J, Soguero-Ruiz C. Artificial Intelligence to Get Insights of Multi-Drug Resistance Risk Factors during the First 48 Hours from ICU Admission. *Antibiotics (Basel)*. 2021;10(3)doi:10.3390/antibiotics10030239
716. Morales FL, Xu F, Lee HA, et al. Open-source computational pipeline automatically flags instances of acute respiratory distress syndrome from electronic health records. *medRxiv*. Mar 1 2025;doi:2024.05.21.24307715 [pii]
10.1101/2024.05.21.24307715
717. Morales Chacon LM, Galan Garcia L, Cruz H, et al. Clinical Phenotypes and Mortality Biomarkers: A Study Focused on COVID-19 Patients with Neurological Diseases in Intensive Care Units. *Behav Sci (Basel)*. 2022;12(7)doi:10.3390/bs12070234
718. Moridani MK, Setarehdan SK, Nasrabadi AM, Hajinasrollah E. New algorithm of mortality risk prediction for cardiovascular patients admitted in intensive care unit. *Int J Clin Exp Med*. 2015;8(6):8916-8926. doi:
719. Morik K, Brockhausen P, Joachims T. Combining statistical learning with a knowledge-based approach: a case study in intensive care monitoring. *Proceedings of the Sixteenth International Conference on Machine Learning*. 1999;
720. Morisson L, Duceau B, Do Rego H, et al. A new machine learning algorithm to predict veno-arterial ECMO implantation after post-cardiotomy low cardiac output syndrome. *Anaesth Crit Care Pain Med*. 2023;42(1)doi:10.1016/j.accpm.2022.101172
721. Moro A, Janjua HM, Rogers MP, et al. Survival Tree Provides Individualized Estimates of Survival After Lung Transplant. *J Surg Res*. 2024;299:195-204. doi:10.1016/j.jss.2024.04.017

722. Moser A, Reinikainen M, Jakob SM, et al. Mortality prediction in intensive care units including premorbid functional status improved performance and internal validity. *J Clin Epidemiol*. 2022;142:230-241. doi:10.1016/j.jclinepi.2021.11.028
723. Mroueh M, Alshamaa D, Mourad-Chehade F, Abdallah F. A Decision-Making System with Reject Option for Atrial Fibrillation Prediction Without ECG Signals. *Irbm*. 2022;43(6):573-584. doi:10.1016/j.irbm.2022.04.008
724. Mugisha C, Paik I. Comparison of Neural Language Modeling Pipelines for Outcome Prediction From Unstructured Medical Text Notes. *IEEE Access*. 2022;10:16489-16498. doi:10.1109/access.2022.3148279
725. Mulkey MA, Huang H, Albanese T, Kim S, Yang B. Supervised deep learning with vision transformer predicts delirium using limited lead EEG. *Sci Rep*. 2023;13(1):7890. doi:10.1038/s41598-023-35004-y
726. Murray B, Zhang T, Most A, et al. Augmenting mortality prediction with medication data and machine learning models. *medRxiv*. 2024:2024.04.16.24305420. doi:10.1101/2024.04.16.24305420
727. Musalia M, Laha S, Cazalilla-Chica J, et al. A user evaluation of speech/phrase recognition software in critically ill patients: a DECIDE-AI feasibility study. *Crit Care*. 2023;27(1):277. doi:10.1186/s13054-023-04420-x
728. Mutlu B, Yeşilyurt ME, Shahbazi N, Güzel MS, Sezer EA. Early prediction of Sepsis: A comparative assessment on patients' covariates. *Biomed Signal Process Control*. 2024;95doi:10.1016/j.bspc.2024.106400
729. Nair SS, Guo A, Boen J, et al. A Real-Time Deep Learning Approach for Inferring Intracranial Pressure from Routinely Measured Extracranial Waveforms in the Intensive Care Unit. *medRxiv*. 2023:2023.05.16.23289747. doi:10.1101/2023.05.16.23289747
730. Nair SS, Guo A, Boen J, et al. A deep learning approach for generating intracranial pressure waveforms from extracranial signals routinely measured in the intensive care unit. *Comput Biol Med*. 2024;177:108677. doi:10.1016/j.compbiomed.2024.108677
731. Nakanishi T, Tsuji T, Tamura T, Fujiwara K, Sobue K. Development and Validation of a Prediction Model for Acute Hypotensive Events in Intensive Care Unit Patients. *J Clin Med*. May 9 2024;13(10)doi:jcm13102786 [pii]
jcm-13-02786 [pii]
10.3390/jcm13102786
732. Nallabasannagari AR, Reddiboina M, Seltzer R. All data inclusive, deep learning models to predict critical events in the medical information mart for intensive care iii database (mimic iii). *arXiv preprint arXiv ….* 2020;doi:
733. Naqvi SG, Shahid K, Shahid A. The Role of Artificial Neural Networks in Machine Learning. *Neuroquantology*. 2022;20(13):3239-3251. doi:10.14704/nq.2022.20.13.NQ88398
734. Narula G, Haeberlin M, Balsiger J, Strassle C, Imbach LL, Keller E. Detection of EEG burst-suppression in neurocritical care patients using an unsupervised machine learning algorithm. *Clin Neurophysiol*. 2021;132(10):2485-2492. doi:10.1016/j.clinph.2021.07.018
735. Nayebi A, Tipirneni S, Reddy CK, Foreman B, Subbian V. WindowSHAP: An Efficient Framework for Explaining Time-series Classifiers based on Shapley Values. *J Biomed Inform*. 2023:104438. doi:10.1016/j.jbi.2023.104438
736. Nazir S, Pateau V, Bert J, et al. Surface imaging for real-time patient respiratory function assessment in intensive care. *Med Phys*. 2021;48(1):142-155. doi:10.1002/mp.14557
737. Nemeth C, Amos-Binks A, Burris C, et al. Decision Support for Tactical Combat Casualty Care Using Machine Learning to Detect Shock. *Mil Med*. 2021;186:273-280. doi:10.1093/milmed/usaa275
738. Nesaragi N, Patidar S. Early Prediction of Sepsis From Clinical Data Using Ratio and Power-Based Features. *Crit Care Med*. 2020;48(12):e1343-e1349. doi:10.1097/ccm.0000000000004691
739. Nesaragi N, Patidar S, Thangaraj V. A correlation matrix-based tensor decomposition method for early prediction of sepsis from clinical data. *Biocybern Biomed Eng*. 2021;41(3):1013-1024. doi:10.1016/j.bbe.2021.06.009
740. Nesaragi N, Patidar S, Aggarwal V. Tensor learning of pointwise mutual information from EHR data for early prediction of sepsis. *Comput Biol Med*. 2021;134:104430. doi:10.1016/j.compbiomed.2021.104430
741. Nestor B, McDermott MBA, Boag W. Feature robustness in non-stationary health records: caveats to deployable model performance in common clinical machine learning tasks. *Machine Learning ….* 2019;doi:
742. Neyra JA, Ortiz-Soriano V, Liu LJ, et al. Prediction of Mortality and Major Adverse Kidney Events in Critically Ill Patients With Acute Kidney Injury. *Am J Kidney Dis*. 2023;81(1):36-47. doi:10.1053/j.ajkd.2022.06.004

743. Ng QA, Chiew YS, Wang X, et al. Network Data Acquisition and Monitoring System for Intensive Care Mechanical Ventilation Treatment. *IEEE Access*. 2021;9:91859-91873. doi:10.1109/access.2021.3092194
744. Nguyen-Duc T, Tay A, Chen D, et al. Estimation of Clinical Workload and Patient Activity Using Deep Learning and Optical Flow. *IEEE Sens Lett*. 2022;6(7)doi:10.1109/lsens.2022.3181600
745. Nie X, Cai Y, Liu J, et al. Mortality Prediction in Cerebral Hemorrhage Patients Using Machine Learning Algorithms in Intensive Care Units. *Front Neurol*. 2020;11:610531. doi:10.3389/fneur.2020.610531
746. Nie XK, Zhao X. Forecasting medical state transition using machine learning methods. *Sci Rep*. 2022;12(1)doi:10.1038/s41598-022-24408-x
747. Nie W, Yu Y, Zhang C, Song D, Zhao L, Bai Y. Temporal-Spatial Correlation Attention Network for Clinical Data Analysis in Intensive Care Unit. *IEEE Trans Biomed Eng*. 2024;71(2):583-595. doi:10.1109/tbme.2023.3309956
748. Ning YL, Sun C, Xu XH, et al. Tendency of dynamic vasoactive and inotropic medications data as a robust predictor of mortality in patients with septic shock: An analysis of the MIMIC-IV database. *Front cardiovasc med*. 2023;10:1126888. doi:10.3389/fcvm.2023.1126888
749. Nistal-Nuno B. Developing machine learning models for prediction of mortality in the medical intensive care unit. *Comput Methods Programs Biomed*. 2022;216:106663. doi:10.1016/j.cmpb.2022.106663
750. Nistal-Nuno B. Outcome prediction for critical care patients with respiratory neoplasms using a multilayer perceptron neural network. *Einstein*. 2023;21:eAO0071. doi:10.31744/einstein_journal/2023AO0071
751. Nistal-Nuno B. Machine learning applied to a Cardiac Surgery Recovery Unit and to a Coronary Care Unit for mortality prediction. *J Clin Monit Comput*. 2022;36(3):751-763. doi:10.1007/s10877-021-00703-2
752. Niu K, Pei S, Peng XP, Zeng JN, Zhang K. Intensive Care Unit readmission prediction with correlation enhanced multi-task learning. *Comput Electr Eng*. 2023;110doi:10.1016/j.compeleceng.2023.108780
753. Nori N, Kashima H, Yamashita K, Kunisawa S. Learning implicit tasks for patient-specific risk modeling in ICU. *… on Artificial Intelligence*. 2017;doi:
754. Nourelahi M, Dadboud F, Khalili H, Niakan A, Parsaei H. A machine learning model for predicting favorable outcome in severe traumatic brain injury patients after 6 months. *Acute Crit Care*. 2022;37(1):45-52. doi:10.4266/acc.2021.00486
755. Nowak S, Schneider H, Layer YC, et al. Development of image-based decision support systems utilizing information extracted from radiological free-text report databases with text-based transformers. *Eur Radiol*. May 2024;34(5):2895-2904. doi:10.1007/s00330-023-10373-0 [pii]
10373 [pii]
10.1007/s00330-023-10373-0
756. Nowakowska K, Sakellarios A, Kaźmierski J, Fotiadis DI, Pezoulas VC. AI-Enhanced Predictive Modeling for Identifying Depression and Delirium in Cardiovascular Patients Scheduled for Cardiac Surgery. *Diagn*. 2024;14(1)doi:10.3390/diagnostics14010067
757. Nistal-Nuno B. A neural network for prediction of risk of nosocomial infection at intensive care units: a didactic preliminary model. *Einstein*. 2020;18:eAO5480. doi:10.31744/einstein_journal/2020AO5480
758. Nistal-Nuno B. Artificial intelligence forecasting mortality at an intensive care unit and comparison to a logistic regression system. *Einstein*. 2021;19:eAO6283. doi:10.31744/einstein_journal/2021AO6283
759. Nuryani N, Pambudi Utomo T, Wiyono N, Sutomo AD, Ling S. Cuffless Hypertension Detection using Swarm Support Vector Machine Utilizing Photoplethysmogram and Electrocardiogram. *J biomed phys eng*. 2023;13(5):477-488. doi:10.31661/jbpe.v0i0.2206-1504
760. Oliver M, Renou A, Allou N, Moscatelli L, Ferdynus C, Allyn J. Image augmentation and automated measurement of endotracheal-tube-to-carina distance on chest radiographs in intensive care unit using a deep learning model with external validation. *Crit Care*. 2023;27(1):40. doi:10.1186/s13054-023-04320-0
761. Orangi-Fard N, Akhbardeh A, Sagreiya H. Predictive Model for ICU Readmission Based on Discharge Summaries Using Machine Learning and Natural Language Processing. *Informatics-Basel*. 2022;9(1)doi:10.3390/informatics9010010
762. Ortega-Martorell S, Olier I, Johnston BW, Welters ID. Sepsis-induced coagulopathy is associated with new episodes of atrial fibrillation in patients admitted to critical care in sinus rhythm. *Front Med (Lausanne)*. 2023;10:1230854. doi:10.3389/fmed.2023.1230854

763. Osathitporn P, Sawadwuthikul G, Thuwajit P, et al. RRWaveNet: A Compact End-to-End Multiscale Residual CNN for Robust PPG Respiratory Rate Estimation. *IEEE Internet Things J*. 2023;10(18):15943-15952. doi:10.1109/jiot.2023.3265980
764. Otaguro T, Tanaka H, Igarashi Y, et al. Machine Learning for Prediction of Successful Extubation of Mechanical Ventilated Patients in an Intensive Care Unit: A Retrospective Observational Study. *J Nippon Med Sch*. 2021;88(5):408-417. doi:10.1272/jnms.JNMS.2021_88-508
765. Ben Othman S, Decaudin B, Odou P, Rousseliere C, Cousein E, Hammadi S. Pharmaceutical Decision Support System Using Machine Learning to Analyze and Limit Drug-Related Problems in Hospitals. *Stud Health Technol Inform*. 2024;310:1593-1597. doi:10.3233/shti231332
766. Ouyang Y, Cheng M, He B, et al. Interpretable machine learning models for predicting in-hospital death in patients in the intensive care unit with cerebral infarction. *Comput Methods Programs Biomed*. 2023;231:107431. doi:10.1016/j.cmpb.2023.107431
767. Ovcharenko E, Kutikhin A, Gruzdeva O, et al. Cardiovascular and Renal Comorbidities Included into Neural Networks Predict the Outcome in COVID-19 Patients Admitted to an Intensive Care Unit: Three-Center, Cross-Validation, Age- and Sex-Matched Study. *J Cardiovasc Dev Dis*. 2023;10(2)doi:10.3390/jcdd10020039
768. Padrao EMH, Bustos B, Mahesh A, Fonseca GHH, Taniguchi LU. Phenotypes of sickle cell intensive care admissions: an unsupervised machine learning approach in a single-center retrospective cohort. *Ann Hematol*. 2022;101(9):1951-1957. doi:10.1007/s00277-022-04918-4
769. Pai KC, Wang MS, Chen YF, et al. An Artificial Intelligence Approach to Bloodstream Infections Prediction. *J Clin Med*. 2021;10(13)doi:10.3390/jcm10132901
770. Pai KC, Su SA, Chan MC, Wu CL, Chao WC. Explainable machine learning approach to predict extubation in critically ill ventilated patients: a retrospective study in central Taiwan. *BMC Anesthesiol*. 2022;22(1):351. doi:10.1186/s12871-022-01888-y
771. Pais V, Rao S, Muniyal B, Yun S. FedICU: a federated learning model for reducing the medication prescription errors in intensive care units. *Cogent Eng*. 2024;11(1)doi:10.1080/23311916.2023.2301150
772. Pal R, Patel S, Bhatnagar A, et al. ShockModes: A Multimodal Model for Prognosticating Intensive Care Outcomes from Physician Notes and Vitals. *medRxiv*. 2022:2022.12.16.22283559. doi:10.1101/2022.12.16.22283559
773. Palmowski L, Nowak H, Witowski A, et al. Assessing SOFA score trajectories in sepsis using machine learning: A pragmatic approach to improve the accuracy of mortality prediction. *PLoS One*. 2024;19(3):e0300739. doi:10.1371/journal.pone.0300739
774. Pan P, Li Y, Xiao Y, et al. Prognostic Assessment of COVID-19 in the Intensive Care Unit by Machine Learning Methods: Model Development and Validation. *J Med Internet Res*. 2020;22(11):e23128. doi:10.2196/23128
775. Pan Q, Zhang L, Jia M, et al. An interpretable 1D convolutional neural network for detecting patient-ventilator asynchrony in mechanical ventilation. *Comput Methods Programs Biomed*. 2021;204doi:10.1016/j.cmpb.2021.106057
776. Pan Q, Zhang H, Jiang M, Ning G, Fang L, Ge H. Comprehensive breathing variability indices enhance the prediction of extubation failure in patients on mechanical ventilation. *Comput Biol Med*. 2023;153:106459. doi:10.1016/j.compbiomed.2022.106459
777. Pan X, Xie J, Zhang L, et al. Evaluate prognostic accuracy of SOFA component score for mortality among adults with sepsis by machine learning method. *BMC Infect Dis*. 2023;23(1):76. doi:10.1186/s12879-023-08045-x
778. Pan P, Liu Y, Xie F, et al. Significance of platelets in the early warning of new-onset AKI in the ICU by using supervise learning: a retrospective analysis. *Ren Fail*. 2023;45(1):2194433. doi:10.1080/0886022x.2023.2194433
779. Pan WS, Xu ZX, Rajendran S, Wang F. An adaptive federated learning framework for clinical risk prediction with electronic health records from multiple hospitals. *Patterns*. 2024;5(1)doi:10.1016/j.patter.2023.100898
780. Pan P, Wang Y, Liu C, et al. Revisiting the potential value of vital signs in the real-time prediction of mortality risk in intensive care unit patients. *J Big Data*. 2024;11(1)doi:10.1186/s40537-024-00896-8
781. Pang K, Li L, Ouyang W, Liu X, Tang Y. Establishment of ICU Mortality Risk Prediction Models with Machine Learning Algorithm Using MIMIC-IV Database. *Diagnostics (Basel)*. 2022;12(5)doi:10.3390/diagnostics12051068
782. Pankaj, Kumar A, Komaragiri R, Kumar M. A novel CS-NET architecture based on the unification of CNN, SVM and super-resolution spectrogram to monitor and classify blood pressure

using photoplethysmography. *Comput Methods Programs Biomed*. 2023;240doi:10.1016/j.cmpb.2023.107716
783. Paragliola G, Ribino P, Ullah Z. A Federated Learning Approach to Support the Decision-Making Process for ICU Patients in a European Telemedicine Network. *J Sens Actuat Netw*. 2023;12(6)doi:10.3390/jsan12060078
784. Parente JD, Chase JG, Moeller K, Shaw GM. High Inter-Patient Variability in Sepsis Evolution: A Hidden Markov Model Analysis. *Comput Methods Programs Biomed*. 2021;201doi:10.1016/j.cmpb.2021.105956
785. Park JE, Kim TY, Jung YJ, et al. Biosignal-Based Digital Biomarkers for Prediction of Ventilator Weaning Success. *Int J Environ Res Public Health*. 2021;18(17)doi:10.3390/ijerph18179229
786. Park E, Lee K, Han T, Nam HS. Agreement and Reliability Analysis of Machine Learning Scaling and Wireless Monitoring in the Assessment of Acute Proximal Weakness by Experts and Non-Experts: A Feasibility Study. *J Pers Med*. 2022;12(1)doi:10.3390/jpm12010020
787. Park JH, Cho Y, Shin D, Choi SS. Prediction of Mortality after Burn Surgery in Critically Ill Burn Patients Using Machine Learning Models. *J Pers Med*. 2022;12(8)doi:10.3390/jpm12081293
788. Park JE, Kim DY, Park JH, et al. A machine learning model for predicting weaning success using only ventilator data during spontaneous breathing trials. *Respirology (Carlton, Vic)*. 2023;28:324-325. doi:10.1111/resp.14433
789. Park JE, Kim DY, Park JW, et al. Development of a Machine Learning Model for Predicting Weaning Outcomes Based Solely on Continuous Ventilator Parameters during Spontaneous Breathing Trials. *Bioengineering (Basel)*. Oct 5 2023;10(10)doi:bioengineering10101163 [pii]
bioengineering-10-01163 [pii]
10.3390/bioengineering10101163
790. Parker F, Brodsky MB, Akst LM, Ali H. Machine Learning in Laryngoscopy Analysis: A Proof of Concept Observational Study for the Identification of Post-Extubation Ulcerations and Granulomas. *Ann Otol Rhinol Laryngol*. 2021;130(3):286-291. doi:10.1177/0003489420950364
791. Patel BV, Haar S, slip R, et al. Natural history, trajectory, and management of mechanically ventilated COVID-19 patients in the United Kingdom. *Intensive Care Med*. 2021;47(5):549-565. doi:10.1007/s00134-021-06389-z
792. Na Pattalung T, Ingviya T, Chaichulee S. Feature Explanations in Recurrent Neural Networks for Predicting Risk of Mortality in Intensive Care Patients. *J Pers Med*. 2021;11(9)doi:10.3390/jpm11090934
793. Pattharanitima P, Vaid A, Jaladanki SK, et al. Comparison of Approaches for Prediction of Renal Replacement Therapy-Free Survival in Patients with Acute Kidney Injury. *Blood Purif*. 2021;50(4):621-627. doi:10.1159/000513700
794. Pattharanitima P, Thongprayoon C, Petnak T, et al. Machine Learning Consensus Clustering Approach for Patients with Lactic Acidosis in Intensive Care Units. *J Pers Med*. 2021;11(11)doi:10.3390/jpm11111132
795. Pattharanitima P, Thongprayoon C, Kaewput W, et al. Machine Learning Prediction Models for Mortality in Intensive Care Unit Patients with Lactic Acidosis. *J Clin Med*. 2021;10(21)doi:10.3390/jcm10215021
796. Paul J, Annamalai M, Ming W, Al Badawi A, Veeravalli B, Aung KMM. Privacy-Preserving Collective Learning With Homomorphic Encryption. *IEEE Access*. 2021;9:132084-132096. doi:10.1109/access.2021.3114581
797. Pease M, Arefan D, Barber J, et al. Outcome Prediction in Patients with Severe Traumatic Brain Injury Using Deep Learning from Head CT Scans. *Radiology*. 2022;304(2):385-394. doi:10.1148/radiol.212181
798. Peine A, Hallawa A, Bickenbach J, et al. Development and validation of a reinforcement learning algorithm to dynamically optimize mechanical ventilation in critical care. *NPJ Digit Med*. 2021;4(1)doi:10.1038/s41746-021-00388-6
799. Penarrubia L, Verstraete A, Orkisz M, et al. Precision of CT-derived alveolar recruitment assessed by human observers and a machine learning algorithm in moderate and severe ARDS. *Intensive Care Med Exp*. 2023;11(1)doi:10.1186/s40635-023-00495-6
800. Peng X, Ding Y, Wihl D, Gottesman O. Improving sepsis treatment strategies by combining deep and kernel-based reinforcement learning. *AMIA Annu Symp Proc*. 2018;2018:887-896.
801. Peng X, Li L, Wang X, Zhang H. A Machine Learning-Based Prediction Model for Acute Kidney Injury in Patients With Congestive Heart Failure. *Front cardiovasc med*. 2022;9:842873. doi:10.3389/fcvm.2022.842873
802. Peng S, Huang J, Liu X, et al. Interpretable machine learning for 28-day all-cause in-hospital mortality prediction in critically ill patients with heart failure combined with hypertension: A

retrospective cohort study based on medical information mart for intensive care database-IV an. *Front cardiovasc med*. 2022;9:994359. doi:10.3389/fcvm.2022.994359
803. Peng L, Peng C, Yang F, et al. Machine learning approach for the prediction of 30-day mortality in patients with sepsis-associated encephalopathy. *BMC Med Res Methodol*. 2022;22(1):183. doi:10.1186/s12874-022-01664-z
804. Peng C, Yang F, Li L, et al. A Machine Learning Approach for the Prediction of Severe Acute Kidney Injury Following Traumatic Brain Injury. *Neurocrit Care*. 2023;38(2):335-344. doi:10.1007/s12028-022-01606-z
805. Pennati F, Aliverti A, Pozzi T, et al. Machine learning predicts lung recruitment in acute respiratory distress syndrome using single lung CT scan. *Ann Intensive Care*. 2023;13(1):60. doi:10.1186/s13613-023-01154-5
806. Penny-Dimri JC, Bergmeir C, Reid CM, Williams-Spence J, Cochrane AD, Smith JA. Paying attention to cardiac surgical risk: An interpretable machine learning approach using an uncertainty-aware attentive neural network. *PLoS One*. 2023;18(8)doi:10.1371/journal.pone.0289930
807. Peres IT, Hamacher S, Cyrino Oliveira FL, Bozza FA, Salluh JIF. Data-driven methodology to predict the ICU length of stay: A multicentre study of 99,492 admissions in 109 Brazilian units. *Anaesth Crit Care Pain Med*. 2022;41(6):101142. doi:10.1016/j.accpm.2022.101142
808. Peres IT, Ferrari GF, Quintairos A, Bastos LSL, Salluh JIF. Validation of a new data-driven SLOSR ICU efficiency measure compared to the traditional SRU. *Intensive Care Med*. 2023;49(12):1546-1548. doi:10.1007/s00134-023-07255-w
809. Peronnet E, Terraz G, Cerrato E, et al. Use of Immune Profiling Panel to assess the immune response of septic patients for prediction of worsening as a composite endpoint. *Sci Rep*. 2024;14(1):11305. doi:10.1038/s41598-024-62202-z
810. Persson I, Ostling A, Arlbr, t M, Soderberg J, Becedas D. A Machine Learning Sepsis Prediction Algorithm for Intended Intensive Care Unit Use (NAVOY Sepsis): Proof-of-Concept Study. *JMIR Form Res*. 2021;5(9):e28000. doi:10.2196/28000
811. Persson I, Grünwald A, Morvan L, Becedas D, Arlbr, t M. A Machine Learning Algorithm Predicting Acute Kidney Injury in Intensive Care Unit Patients (NAVOY Acute Kidney Injury): Proof-of-Concept Study. *JMIR Form Res*. 2023;7doi:10.2196/45979
812. Persson I, Macura A, Becedas D, Sjovall F. Early prediction of sepsis in intensive care patients using the machine learning algorithm NAVOY R Sepsis, a prospective randomized clinical validation study. *J Crit Care*. 2024;80:154400. doi:10.1016/j.jcrc.2023.154400
813. Pessoa SMB, Oliveira BSS, Santos WGD, et al. Prediction of septic and hypovolemic shock in intensive care unit patients using machine learning. *Rev bras ter intensiva*. 2022;34(4):477-483. doi:10.5935/0103-507X.20220280-pt
814. Petrov D, Mir, a SP, et al. Prediction of intracranial pressure crises after severe traumatic brain injury using machine learning algorithms. *J Neurosurg*. 2023;139(2):528-535. doi:10.3171/2022.12.Jns221860
815. Pettinati MJ, Chen G, Rajput KS, Selvaraj N. Practical Machine Learning-Based Sepsis Prediction. *Annu Int Conf IEEE Eng Med Biol Soc*. 2020;2020:4986-4991. doi:10.1109/embc44109.2020.9176323
816. Pham SDT, Keijzer HM, Ruijter BJ, et al. Outcome Prediction of Postanoxic Coma: A Comparison of Automated Electroencephalography Analysis Methods. *Neurocrit Care*. 2022;37:248-258. doi:10.1007/s12028-022-01449-8
817. Phetrittikun R, Suvirat K, Horsiritham K, Ingviya T, Chaichulee S. Prediction of Acid-Base and Potassium Imbalances in Intensive Care Patients Using Machine Learning Techniques. *Diagnostics (Basel)*. 2023;13(6)doi:10.3390/diagnostics13061171
818. Piedrafita A, Siwy J, Klein J, et al. A universal predictive and mechanistic urinary peptide signature in acute kidney injury. *Crit Care*. 2022;26(1)doi:10.1186/s13054-022-04193-9
819. Pinevich Y, Amos-Binks A, Burris CS, et al. Validation of a Machine Learning Model for Early Shock Detection. *Mil Med*. 2022;187(1):82-88. doi:10.1093/milmed/usab220
820. Bollen Pinto B, Ribas Ripoll V, Subias-Beltran P, et al. Application of an Exploratory Knowledge-Discovery Pipeline Based on Machine Learning to Multi-Scale OMICS Data to Characterise Myocardial Injury in a Cohort of Patients with Septic Shock: An Observational Study. *J Clin Med*. 2021;10(19)doi:10.3390/jcm10194354
821. Pirracchio R. Mortality Prediction in the ICU Based on MIMIC-II Results from the Super ICU Learner Algorithm (SICULA) Project. *Springer*. 2016:295-313. doi:10.1007/978-3-319-43742-2
822. Pirracchio R, Hubbard A, Sprung CL, Chevret S, Annane D. Assessment of Machine Learning to Estimate the Individual Treatment Effect of Corticosteroids in Septic Shock. *JAMA netw open*. 2020;3(12):e2029050. doi:10.1001/jamanetworkopen.2020.29050

823. Pishgar M, Theis J, Del Rios M, Ardati A, Anahideh H, Darabi H. Prediction of unplanned 30-day readmission for ICU patients with heart failure. *BMC Med Inf Decis Mak*. 2022;22(1):117. doi:10.1186/s12911-022-01857-y
824. Ponce D, de Goes CR, de Andrade LGM. Proposal of a new equation for estimating resting energy expenditure of acute kidney injury patients on dialysis: a machine learning approach. *Nutr Metab*. 2020;17(1)doi:10.1186/s12986-020-00519-y
825. Ponthongmak W, Thammasudjarit R, McKay GJ, Attia J, Theera-Ampornpunt N, Thakkinstian A. Development and external validation of automated ICD-10 coding from discharge summaries using deep learning approaches. *Inform Med Unlocked*. 2023;38doi:10.1016/j.imu.2023.101227
826. Popkes AL, Overweg H, Ercole A, Li Y. Interpretable outcome prediction with sparse Bayesian neural networks in intensive care. *arXiv*. 2019;doi:10.48550/arXiv.1905.02599
827. Potter KM, Kennedy JN, Onyemekwu C, et al. Data-derived subtypes of delirium during critical illness. *EBioMedicine*. 2024;100:104942. doi:10.1016/j.ebiom.2023.104942
828. Poulsen MN, Freda PJ, Troiani V, Davoudi A, Mowery DL. Classifying Characteristics of Opioid Use Disorder From Hospital Discharge Summaries Using Natural Language Processing. *Front public health*. 2022;10:850619. doi:10.3389/fpubh.2022.850619
829. Prasad N, yam A, Chivers C, et al. Guiding Efficient, Effective, and Patient-Oriented Electrolyte Replacement in Critical Care: An Artificial Intelligence Reinforcement Learning Approach. *J Pers Med*. 2022;12(5)doi:10.3390/jpm12050661
830. Prasad N, Cheng LF, Chivers C, Draugelis M. A reinforcement learning approach to weaning of mechanical ventilation in intensive care units. *arXiv preprint arXiv …*. 2017;doi:
831. Qi J, Lei J, Li N, et al. Machine learning models to predict in-hospital mortality in septic patients with diabetes. *Front Endocrinol (Lausanne)*. 2022;13:1034251. doi:10.3389/fendo.2022.1034251
832. Qian Q, Wu J, Wang J, Sun H, Yang L. Prediction Models for AKI in ICU: A Comparative Study. *Int J Gen Med*. 2021;14:623-632. doi:10.2147/ijgm.S289671
833. Qiu Y, Ding S, Yao NG, Gu DX, Li XJ. HFS-LightGBM: A machine learning model based on hybrid feature selection for classifying ICU patient readmissions. *Expert Syst*. 2021;38(3)doi:10.1111/exsy.12658
834. Qiu XH, Tan XY, Li Q, Chen ST, Ru YJ, Jin YC. A latent batch-constrained deep reinforcement learning approach for precision dosing clinical decision support. *Knowledge-Based Syst*. 2022;237doi:10.1016/j.knosys.2021.107689
835. Qiu X, Tan X, Wang C, Chen S, Du B, Huang J. A long short-temory relation network for real-time prediction of patient-specific ventilator parameters. *Math Biosci Eng*. 2023;20(8):14756-14776. doi:10.3934/mbe.2023660
836. Quesado I, Duarte J, Silva A, Manuel M, Quintas C. Data Mining Models for Automatic Problem Identification in Intensive Medicine. *Procedia Comput Sci*. 2022;210:218-223. doi:10.1016/j.procs.2022.10.140
837. Tran Quoc V, Nguyen Thi Ngoc D, Nguyen Hoang T, et al. Predicting Antibiotic Resistance in ICUs Patients by Applying Machine Learning in Vietnam. *Infect drug resist*. 2023;16:5535-5546. doi:10.2147/idr.S415885
838. Radhakrishnan S, Nair SG, Isaac J. Multilayer perceptron neural network model development for mechanical ventilator parameters prediction by real time system learning. *Biomedical signal processing control*. 2022;71:103170. doi:10.1016/j.bspc.2021.103170
839. Rafiei A, Ghiasi Rad M, Sikora A, Kamaleswaran R. Improving mixed-integer temporal modeling by generating synthetic data using conditional generative adversarial networks: A case study of fluid overload prediction in the intensive care unit. *Comput Biol Med*. 2024;168:107749. doi:10.1016/j.compbiomed.2023.107749
840. Raghu A, Komorowski M, Ahmed I, Celi L. Deep reinforcement learning for sepsis treatment. *arXiv preprint arXiv …*. 2017;doi:
841. Raghu A, Komorowski M, Singh S. Model-based reinforcement learning for sepsis treatment. *arXiv preprint arXiv:1811 09602*. 2018;doi:
842. Rahman A, Chang Y, Dong J, et al. Early prediction of hemodynamic interventions in the intensive care unit using machine learning. *Crit Care*. 2021;25(1):388. doi:10.1186/s13054-021-03808-x
843. Raj R, Wennervirta JM, Tjerkaski J, et al. Dynamic prediction of mortality after traumatic brain injury using a machine learning algorithm. *NPJ Digit Med*. 2022;5(1):96. doi:10.1038/s41746-022-00652-3

844. Rajendran S, Xu Z, Pan W, Ghosh A, Wang F. Data heterogeneity in federated learning with Electronic Health Records: Case studies of risk prediction for acute kidney injury and sepsis diseases in critical care. *PLOS Digit Health*. 2023;2(3):e0000117. doi:10.1371/journal.pdig.0000117
845. Rajendran S, Xu Z, Pan W, et al. Corticosteroids for infectious critical illness: A multicenter target trial emulation stratified by predicted organ dysfunction trajectory. *medRxiv*. Mar 8 2024;doi:2024.03.07.24303926 [pii]
10.1101/2024.03.07.24303926
846. Ramakrishnaiah Y, Macesic N, Webb GI, Peleg AY, Tyagi S. EHR-ML: A generalisable pipeline for reproducible clinical outcomes using electronic health records. *medRxiv*. 2024:2024.03.02.24302664. doi:10.1101/2024.03.02.24302664
847. Ramos G, Gjini E, Coelho L, Silveira M. Unsupervised learning approach for predicting sepsis onset in ICU patients. *Annu Int Conf IEEE Eng Med Biol Soc*. 2021;2021:1916-1919. doi:10.1109/embc46164.2021.9629559
848. Rangan ES, Pathinarupothi RK, An, S KJ, Snyder MP. Performance effectiveness of vital parameter combinations for early warning of sepsis-an exhaustive study using machine learning. *JAMIA open*. 2022;5(4):ooac080. doi:10.1093/jamiaopen/ooac080
849. Rank N, Pfahringer B, Kempfert J, et al. Deep-learning-based real-time prediction of acute kidney injury outperforms human predictive performance. *NPJ Digit Med*. 2020;3(1)doi:10.1038/s41746-020-00346-8
850. Rannon E, Goldschmidt E, Bernstein D, Wasserman A, Coster D, Shamir R. Predicting appropriateness of antibiotic treatment among ICU patients with hospital acquired infection. *medRxiv*. 2023:2023.08.15.23294109. doi:10.1101/2023.08.15.23294109
851. Raphaeli O, Statlender L, Hajaj C, et al. Using Machine-Learning to Assess the Prognostic Value of Early Enteral Feeding Intolerance in Critically Ill Patients: A Retrospective Study. *Nutrients*. 2023;15(12)doi:10.3390/nu15122705
852. Rastegar AS, GholamHosseini AH, Lowe AA, Linden BM. Continuous Blood Pressure Estimation From Non-Invasive Measurements Using Support Vector Regression. *Annu Int Conf IEEE Eng Med Biol Soc*. 2021;2021:1487-1490. doi:10.1109/embc46164.2021.9629685
853. Rauseo M, Perrini M, Gallo C, et al. Machine learning and predictive models: 2 years of Sars-CoV-2 pandemic in a single-center retrospective analysis. *J Anesth Analg Crit Care*. 2022;2(1):42. doi:10.1186/s44158-022-00071-6
854. Rayan Z, Alfonse M, Salem ABM. Predicting Sepsis in the Intensive Care Unit (ICU) through Vital Signs using Support Vector Machine (SVM). *Open Bioinformatics J*. 2021;14(1):108-113. doi:10.2174/18750362021140100108
855. Reamaroon N, Sjoding MW, Gryak J, Athey BD, Najarian K, Derksen H. Automated detection of acute respiratory distress syndrome from chest X-Rays using Directionality Measure and deep learning features. *Comput Biol Med*. 2021;134:104463. doi:10.1016/j.compbiomed.2021.104463
856. Rehm GB, Woo SH, Chen XL, et al. Leveraging IoTs and Machine Learning for Patient Diagnosis and Ventilation Management in the Intensive Care Unit. *IEEE Pervasive Comput*. 2020;19(3):68-78. doi:10.1109/mprv.2020.2986767
857. Rehm GB, Cortes-Puch I, Kuhn BT, et al. Use of Machine Learning to Screen for Acute Respiratory Distress Syndrome Using Raw Ventilator Waveform Data. *Crit care explor*. 2021;3(1):e0313. doi:10.1097/cce.0000000000000313
858. Ren S, Zupetic JA, Tabary M, et al. Machine learning based algorithms to impute PaO2 from SpO2 values and development of an online calculator. *Sci Rep*. 2022;12(1):8235. doi:10.1038/s41598-022-12419-7
859. Ren N, Zhao X, Zhang X. Mortality prediction in ICU Using a Stacked Ensemble Model. *Comput Math Methods Med*. 2022;2022:3938492. doi:10.1155/2022/3938492
860. Ren S, Niu J, Cai M, Shi Y, Wang T, Luo Z. Sputum deposition classification for mechanically ventilated patients using LSTM method based on airflow signals. *Heliyon*. 2022;8(12):e11929. doi:10.1016/j.heliyon.2022.e11929
861. Ren S, Wang XH, Hao LM, et al. 1D-CNNs model for classification of sputum deposition degree in mechanical ventilated patients based on airflow signals. *Expert Syst Appl*. 2024;237doi:10.1016/j.eswa.2023.121621
862. Ren W, Zou K, Huang S, et al. Prediction of in-hospital Mortality of Intensive Care Unit Patients with Acute Pancreatitis Based on an Explainable Machine Learning Algorithm. *J Clin Gastroenterol*. Jul 1 2024;58(6):619-626. doi:00004836-990000000-00212 [pii]
10.1097/MCG.0000000000001910
863. Rezaei MR, Fard RS, Pourjafari E, et al. Reverse survival model (RSM): a pipeline for explaining predictions of deep survival models. *Appl Intell*. 2023;doi:10.1007/s10489-023-04577-6

864. Rhodes G, Davidian M, Lu WB. Dynamic Prediction of Residual Life with Longitudinal Covariates Using Long Short-Term Memory Networks. *Ann Appl Stat*. 2023;17(3):2039-2058. doi:10.1214/22-aoas1706
865. Rhodes G, Davidian M, Lu W. Estimation of optimal treatment regimes with electronic medical record data using the residual life value estimator. *Biostatistics*. Oct 1 2024;25(4):933-946. doi:7604037 [pii]
kxae002 [pii]
10.1093/biostatistics/kxae002
866. Rigo-Bonnin R, Gumucio-Sanguino VD, Perez F, et al. Individual outcome prediction models for patients with COVID-19 based on their first day of admission to the intensive care unit. *Clin Biochem*. 2022;100:13-21. doi:10.1016/j.clinbiochem.2021.11.001
867. Rodriguez A, Ruiz-Botella M, Martin-Loeches I, et al. Deploying unsupervised clustering analysis to derive clinical phenotypes and risk factors associated with mortality risk in 2022 critically ill patients with COVID-19 in Spain. *Crit Care*. 2021;25(1):63. doi:10.1186/s13054-021-03487-8
868. Rodriguez A, Gomez J, Franquet A, et al. Applicability of an unsupervised cluster model developed on first wave COVID-19 patients in second/third wave critically ill patients. *Med Intensiva (Engl Ed)*. Jun 2024;48(6):326-340. doi:S2173-5727(24)00042-0 [pii]
10.1016/j.medine.2024.02.006
869. Rodriguez-Obregon DE, Mejia-Rodriguez AR, Cendejas-Zaragoza L, et al. Semi-supervised COVID-19 volumetric pulmonary lesion estimation on CT images using probabilistic active contour and CNN segmentation. *Biomed Signal Process Control*. 2023;85doi:10.1016/j.bspc.2023.104905
870. Roggeveen L, El Hassouni A, Ahrendt J, et al. Transatlantic transferability of a new reinforcement learning model for optimizing haemodynamic treatment for critically ill patients with sepsis. *Artif Intell Med*. 2021;112:102003. doi:10.1016/j.artmed.2020.102003
871. Roggeveen LF, Hassouni AE, de Grooth HJ, Girbes ARJ, Hoogendoorn M, Elbers PWG. Reinforcement learning for intensive care medicine: actionable clinical insights from novel approaches to reward shaping and off-policy model evaluation. *Intensive care med exp*. 2024;12(1):32. doi:10.1186/s40635-024-00614-x
872. Romanov A, Shahar Y. Automated Process Mining and Learning of Therapeutic Actions in the Intensive Care Unit. *Stud Health Technol Inform*. 2024;310:825-829. doi:10.3233/shti231080
873. Romanowski B, Ben Abacha A, Fan Y. Extracting social determinants of health from clinical note text with classification and sequence-to-sequence approaches. *J Am Med Inform Assoc*. 2023;doi:10.1093/jamia/ocad071
874. Rong F, Xiang H, Qian L, Xue Y, Ji K, Yin R. Machine Learning for Prediction of Outcomes in Cardiogenic Shock. *Front cardiovasc med*. 2022;9:849688. doi:10.3389/fcvm.2022.849688
875. Roquencourt C, Lamy E, Bardin E, Devillier P, Grassin-Delyle S. A benchmark study of data normalisation methods for PTR-TOF-MS exhaled breath metabolomics. *J Breath Res*. Nov 10 2023;18(1)doi:10.1088/1752-7163/ad08ce
876. Rosenfeld N, Last M. Using ECG signals for hypotensive episode prediction in trauma patients. *Comput Methods Programs Biomed*. 2022;223doi:10.1016/j.cmpb.2022.106955
877. Roy S, Mincu D, Loreaux E, et al. Multitask prediction of organ dysfunction in the intensive care unit using sequential subnetwork routing. *J Am Med Inform Assoc*. 2021;28(9):1936-1946. doi:10.1093/jamia/ocab101
878. Ruan H, Li SS, Zhang Q, Ran X. Elevated MMP-8 levels, inversely associated with BMI, predict mortality in mechanically ventilated patients: an observational multicenter study. *Crit Care*. 2023;27(1)doi:10.1186/s13054-023-04579-3
879. Rubinos C, Kwon SB, Megjhani M, et al. Predicting Shunt Dependency from the Effect of Cerebrospinal Fluid Drainage on Ventricular Size. *Neurocrit Care*. 2022;37(3):670-677. doi:10.1007/s12028-022-01538-8
880. Ruyssinck J, van der Herten J, Houthooft R, et al. Random Survival Forests for Predicting the Bed Occupancy in the Intensive Care Unit. *Comput Math Method Med*. 2016;2016doi:10.1155/2016/7087053
881. Ryan L, Lam C, Mataraso S, et al. Mortality prediction model for the triage of COVID-19, pneumonia, and mechanically ventilated ICU patients: A retrospective study. *Ann Med Surg (Lond)*. 2020;59:207-216. doi:10.1016/j.amsu.2020.09.044
882. Ryan CT, Zeng Z, Chatterjee S, et al. Machine learning for dynamic and early prediction of acute kidney injury after cardiac surgery. *J Thorac Cardiovasc Surg*. 2022;doi:10.1016/j.jtcvs.2022.09.045

883. Sabeti E, Drews J, Reamaroon N, et al. Learning Using Partially Available Privileged Information and Label Uncertainty: Application in Detection of Acute Respiratory Distress Syndrome. *IEEE j biomed health inform*. 2021;25(3):784-796. doi:10.1109/jbhi.2020.3008601
884. Safaei N, Safaei B, Seyedekrami S, et al. E-CatBoost: An efficient machine learning framework for predicting ICU mortality using the eICU Collaborative Research Database. *PLoS One*. 2022;17(5):e0262895. doi:10.1371/journal.pone.0262895
885. Sakagianni A, Koufopoulou C, Kalles D, Loupelis E, Verykios VS, Feretzakis G. Automated ML Techniques for Predicting COVID-19 Mortality in the ICU. *Stud Health Technol Inform*. 2023;305:517-520. doi:10.3233/shti230547
886. Sakagianni A, Koufopoulou C, Verykios V, Loupelis E, Kalles D, Feretzakis G. Prediction of COVID-19 Mortality in the Intensive Care Unit Using Machine Learning. *Stud Health Technol Inform*. 2023;302:536-540. doi:10.3233/shti230200
887. Sakhaee E, Amirahmadi A, Mahdiani M, et al. Developing a novel prediction model in opioid overdose using machine learning; a pilot analytical study. *Health Sci Rep*. 2022;5(5):e767. doi:10.1002/hsr2.767
888. Saleh H, Younis EMG, Sahal R, Ali AA. Predicting Systolic Blood Pressure in Real-Time Using Streaming Data and Deep Learning. *Mobile Netw Appl*. 2021;26(1):326-335. doi:10.1007/s11036-020-01645-w
889. Salimi M, Bastani P, Nasiri M, Karajizadeh M, Ravangard R. Predicting Readmission of Cardiovascular Patients Admitted to the CCU using Data Mining Techniques. *Open Cardiovasc Med J*. 2023;17doi:10.2174/18741924-v17-e230627-2022-21
890. Salman S, Payrovnaziri SN, Liu X, Rengifo-Moreno P, He Z. DeepConsensus: Consensus-based Interpretable Deep Neural Networks with Application to Mortality Prediction. *Proc Int Jt Conf Neural Netw*. 2020;2020doi:10.1109/ijcnn48605.2020.9206678
891. Samadani A, Wang T, van Zon K, Celi LA. VAP risk index: Early prediction and hospital phenotyping of ventilator-associated pneumonia using machine learning. *Artif Intell Med*. 2023;146:102715. doi:10.1016/j.artmed.2023.102715
892. Samadi ME, Kiefer S, Fritsch SJ, Bickenbach J, Schuppert A. A training strategy for hybrid models to break the curse of dimensionality. *PLoS One*. 2022;17(9):e0274569. doi:10.1371/journal.pone.0274569
893. Samadi ME, Guzman-Maldonado J, Nikulina K, et al. A hybrid modeling framework for generalizable and interpretable predictions of ICU mortality across multiple hospitals. *Sci Rep*. 2024;14(1):5725. doi:10.1038/s41598-024-55577-6
894. Samy SS, Karthick S, Ghosal M, Singh S, Sudarsan JS, Nithiyanantham S. Adoption of machine learning algorithm for predicting the length of stay of patients (construction workers) during COVID pandemic. *Int j inf*. 2023:1-9. doi:10.1007/s41870-023-01296-6
895. Saner FH, Saner YM, Abufarhaneh E, Broering DC, Raptis DA. Comparative Analysis of Artificial Intelligence (AI) Languages in Predicting Sequential Organ Failure Assessment (SOFA) Scores. *Cureus*. 2024;16(5):e59662. doi:10.7759/cureus.59662
896. Sanfilippo F, La Via L, Dezio V, et al. Inferior vena cava distensibility from subcostal and trans-hepatic imaging using both M-mode or artificial intelligence: a prospective study on mechanically ventilated patients. *Intensive care med exp*. 2023;11(1):40. doi:10.1186/s40635-023-00529-z
897. Sarkar S, Ghosh A. Schrodinger spectrum based continuous cuff-less blood pressure estimation using clinically relevant features from PPG signal and its second derivative. *Comput Biol Med*. 2023;166:107558. doi:10.1016/j.compbiomed.2023.107558
898. Sarraf E, Sadr AV, Abedi V, Bonavia AS. Integrating Social Determinants of Health with SOFA Scoring to Enhance Mortality Prediction in Septic Patients: A Multidimensional Prognostic Model. *medRxiv*. 2024:2024.03.13.24304233. doi:10.1101/2024.03.13.24304233
899. Sato N, Uchino E, Kojima R, Hiragi S, Yanagita M, Okuno Y. Prediction and visualization of acute kidney injury in intensive care unit using one-dimensional convolutional neural networks based on routinely collected data. *Comput Methods Programs Biomed*. 2021;206:106129. doi:10.1016/j.cmpb.2021.106129
900. Savage T, Wang JH, Shieh L. A Large Language Model Screening Tool to Target Patients for Best Practice Alerts: Development and Validation. *JMIR Med Inf*. 2023;11doi:10.2196/49886
901. Savareh BA, Alibabaei A, Ahmady S, Mokhtari M, Hajiesmaeili M, Nateghinia S. Predicting patient length of stay in a neurosurgical intensive care unit of a large teaching hospital. *J Cell Mol Anesth*. 2021;6(2):132-140. doi:10.22037/jcma.v6i2.32395
902. Sayed M, Riano D, Villar J. Novel criteria to classify ARDS severity using a machine learning approach. *Crit Care*. 2021;25(1):150. doi:10.1186/s13054-021-03566-w

903. Sayed M, Riano D, Villar J. Predicting Duration of Mechanical Ventilation in Acute Respiratory Distress Syndrome Using Supervised Machine Learning. *J Clin Med*. 2021;10(17)doi:10.3390/jcm10173824
904. Scales NB, Herry CL, van Beinum A, et al. Predicting Time to Death After Withdrawal of Life-Sustaining Measures Using Vital Sign Variability: Derivation and Validation. *Crit care explor*. 2022;4(4):e0675. doi:10.1097/cce.0000000000000675
905. Scheibner A, Betthauser KD, Bewley AF, et al. Machine learning to predict vasopressin responsiveness in patients with septic shock. *Pharmacotherapy*. 2022;42(6):460-471. doi:10.1002/phar.2683
906. Schmidt M, Guidet B, Demoule A, et al. Predicting 90-day survival of patients with COVID-19: Survival of Severely Ill COVID (SOSIC) scores. *Ann Intensive Care*. 2021;11(1):170. doi:10.1186/s13613-021-00956-9
907. Schmidt SV, Drysch M, Reinkemeier F, et al. Improvement of Predictive Scores in Burn Medicine through Different Machine Learning Approaches. *Healthcare (Basel)*. Aug 31 2023;11(17)doi:healthcare11172437 [pii]
healthcare-11-02437 [pii]
10.3390/healthcare11172437
908. Schulz D, Rasch S, Heilmaier M, et al. A deep learning model enables accurate prediction and quantification of pulmonary edema from chest X-rays. *Crit Care*. 2023;27(1):201. doi:10.1186/s13054-023-04426-5
909. Schvetz M, Fuchs L, Novack V, Moskovitch R. Outcomes prediction in longitudinal data: Study designs evaluation, use case in ICU acquired sepsis. *J Biomed Inform*. 2021;117:103734. doi:10.1016/j.jbi.2021.103734
910. Schwager E, Lanius S, Ghosh E, et al. Including urinary output to define AKI enhances the performance of machine learning models to predict AKI at admission. *J Crit Care*. 2021;62:283-288. doi:10.1016/j.jcrc.2021.01.003
911. Schwager E, Ghosh E, Eshelman L, Pasupathy KS, Barreto EF, Kashani K. Accurate and interpretable prediction of ICU-acquired AKI. *J Crit Care*. 2023;75:154278. doi:10.1016/j.jcrc.2023.154278
912. Schwager E, Liu X, Nabian M, et al. Machine learning prediction of the total duration of invasive and non-invasive ventilation During ICU Stay. *PLOS Digit Health*. 2023;2(9):e0000289. doi:10.1371/journal.pdig.0000289
913. Schwager E, Nabian M, Liu X, et al. Machine learning modelling for predicting the utilization of invasive and non-invasive ventilation throughout the ICU duration. *Healthc Technol Lett*. 2024;doi:10.1049/htl2.12081
914. Schweingruber N, Mader MMD, Wiehe A, et al. A recurrent machine learning model predicts intracranial hypertension in neurointensive care patients. *Brain*. 2022;145(8):2910-2919. doi:10.1093/brain/awab453
915. Schweingruber N, Bremer J, Wiehe A, et al. Early prediction of ventricular peritoneal shunt dependency in aneurysmal subarachnoid haemorrhage patients by recurrent neural network-based machine learning using routine intensive care unit data. *J Clin Monit Comput*. Oct 2024;38(5):1175-1186. doi:10.1007/s10877-024-01151-4 [pii]
1151 [pii]
10.1007/s10877-024-01151-4
916. Searle T, Ibrahim Z, Teo J, Dobson R. Estimating redundancy in clinical text. *J Biomed Inform*. 2021;124:103938. doi:10.1016/j.jbi.2021.103938
917. Selcuk M, Koc O, Kestel AS. The prediction power of machine learning on estimating the sepsis mortality in the intensive care unit. *Inform Med Unlocked*. 2022;28doi:10.1016/j.imu.2022.100861
918. Selvaskandan H, Gaultney T, Heath D, Linfoot S, Xu G. Can artificial intelligence predict the need for acute renal replacement therapy among inpatients with acute kidney injury? *Future Healthc J*. 2023;10:S19-S20. doi:10.7861/fhj.10-3-s19
919. Semeraro F, Monsieurs KG. Synthesizing and communicating scientific evidence into treatment recommendations using artificial intelligence: The “case” of temperature control after cardiac arrest. *Resuscitation*. 2024;197doi:10.1016/j.resuscitation.2024.110145
920. Sena J, Bandyopadhyay S, Mostafiz MT, et al. Diurnal Pain Classification in Critically Ill Patients using Machine Learning on Accelerometry and Analgesic Data. *IEEE Int Conf Bioinform Biomed Workshops*. 2023;2023:2207-2212. doi:10.1109/bibm58861.2023.10385764
921. Sena J, Mostafiz MT, Zhang J, et al. Wearable sensors in patient acuity assessment in critical care. *Front Neurol*. 2024;15:1386728. doi:10.3389/fneur.2024.1386728

922. Serafim LP, Simonetto DA, Choi DH, et al. DERIVATION OF A MORTALITY PREDICTION MODEL IN CRITICAL CARE PATIENTS WITH CIRRHOSIS AND SEPSIS. *Shock*. 2024;61(3):382-386. doi:10.1097/shk.0000000000002323
923. Servia L, Montserrat N, Badia M, et al. Machine learning techniques for mortality prediction in critical traumatic patients: anatomic and physiologic variables from the RETRAUCI study. *BMC Med Res Methodol*. 2020;20(1):262. doi:10.1186/s12874-020-01151-3
924. Seymour SE, Rava RA, Swetz DJ, et al. Predicting Hematoma Expansion after Spontaneous Intracranial Hemorrhage Through a Radiomics Based Model. *Proc SPIE Int Soc Opt Eng*. 2022;12033doi:10.1117/12.2611847
925. Shaikh F, Kenny JE, Awan O, et al. Measuring the accuracy of cardiac output using POCUS: the introduction of artificial intelligence into routine care. *Ultrasound J*. 2022;14(1):47. doi:10.1186/s13089-022-00301-6
926. Shankar V, Yousefi E, Manashty A, Blair D, Teegapuram D. Clinical-GAN: Trajectory Forecasting of Clinical Events using Transformer and Generative Adversarial Networks. *Artif Intell Med*. 2023;138doi:10.1016/j.artmed.2023.102507
927. Shapiro M, Shahar Y. Treatment Prediction in the ICU Setting Using a Partitioned, Sequential Deep Time Series Analysis. *Stud Health Technol Inform*. 2022;295:360-361. doi:10.3233/shti220738
928. Sharafoddini A, Dubin JA, Lee J. Identifying subpopulations of septic patients: A temporal data-driven approach. *Comput Biol Med*. 2021;130doi:10.1016/j.compbiomed.2020.104182
929. Sharma P, Shamout FE, Abrol V, Clifton DA. Data Pre-Processing Using Neural Processes for Modeling Personalized Vital-Sign Time-Series Data. *IEEE J Biomed Health Inform*. 2022;26(4):1528-1537. doi:10.1109/jbhi.2021.3107518
930. Sharma DK, Lakhotia P, Sain P, Brahmachari S. Early prediction and monitoring of sepsis using sequential long short term memory model. *Expert Syst*. 2022;39(3)doi:10.1111/exsy.12798
931. Shashikumar SP, Josef CS, Sharma A, Nemati S. DeepAISE - An interpretable and recurrent neural survival model for early prediction of sepsis. *Artif Intell Med*. 2021;113:102036. doi:10.1016/j.artmed.2021.102036
932. Shashikumar SP, Wardi G, Paul P, et al. Development and Prospective Validation of a Deep Learning Algorithm for Predicting Need for Mechanical Ventilation. *Chest*. 2021;159(6):2264-2273. doi:10.1016/j.chest.2020.12.009
933. Shawwa K, Ghosh E, Lanius S, Schwager E, Eshelman L, Kashani KB. Predicting acute kidney injury in critically ill patients using comorbid conditions utilizing machine learning. *Clin Kidney J*. 2021;14(5):1428-1435. doi:10.1093/ckj/sfaa145
934. She S, Shen Y, Luo K, Zhang X, Luo C. Prediction of Acute Kidney Injury in Intracerebral Hemorrhage Patients Using Machine Learning. *Neuropsychiatr Dis Treat*. 2023;19:2765-2773. doi:10.2147/ndt.S439549
935. She Y, Zhou L, Li Y. Interpretable machine learning models for predicting 90-day death in patients in the intensive care unit with epilepsy. *Seizure*. 2024;114:23-32. doi:10.1016/j.seizure.2023.11.017
936. Sheetrit E, Brief M, Elisha O. Predicting unplanned readmissions in the intensive care unit: a multimodality evaluation. *Sci Rep*. 2023;13(1):15426. doi:10.1038/s41598-023-42372-y
937. Sheikh MS, Thongprayoon C, Suppadungsuk S, et al. Evaluating ChatGPT's Accuracy in Responding to Patient Education Questions on Acute Kidney Injury and Continuous Renal Replacement Therapy. *Blood Purif*. 2024;doi:10.1159/000539065
938. Sheikh MS, Thongprayoon C, Qureshi F, et al. Personalized Medicine Transformed: ChatGPT's Contribution to Continuous Renal Replacement Therapy Alarm Management in Intensive Care Units. *J Pers Med*. Feb 22 2024;14(3)doi:jpm14030233 [pii]
jpm-14-00233 [pii]
10.3390/jpm14030233
939. Sheikhalishahi S, Bhattacharyya A, Celi LA, Osmani V. An interpretable deep learning model for time-series electronic health records: Case study of delirium prediction in critical care. *Artif Intell Med*. 2023;144:102659. doi:10.1016/j.artmed.2023.102659
940. Sheikhalishahi S, Kaspar M, Zaghdoudi S, et al. Predicting Successful Weaning from Mechanical Ventilation by Reduction in Positive End-expiratory Pressure Level Using Machine Learning. *PLOS Digit Health*. 2024;3(3):e0000478. doi:10.1371/journal.pdig.0000478
941. Shi DJ, Zheng HZ. A Mortality Risk Assessment Approach on ICU Patients Clinical Medication Events Using Deep Learning. *CMES-Comp Model Eng Sci*. 2021;128(1):161-181. doi:10.32604/cmes.2021.014917

942. Shi S. A novel hybrid deep learning architecture for predicting acute kidney injury using patient record data and ultrasound kidney images. *Appl Artif Intell*. 2021;35(15):1329-1345. doi:10.1080/08839514.2021.1976908
943. Shi ZK, Wang S, Yue L, et al. Deep dynamic imputation of clinical time series for mortality prediction. *Inf Sci*. 2021;579:607-622. doi:10.1016/j.ins.2021.08.016
944. Shi K, Ho V, Song JJ, Bechler K, J HC. Predicting Unplanned 7-day Intensive Care Unit Readmissions with Machine Learning Models for Improved Discharge Risk Assessment. *AMIA Annu Symp Proc*. 2022;2022:446-455. doi:
945. Shi ZK, Wang S, Yue L, et al. Dual-core mutual learning between scoring systems and clinical features for ICU mortality prediction. *Inf Sci*. 2023;637doi:10.1016/j.ins.2023.118984
946. Shi H, Shen Y, Li L. Early prediction of acute kidney injury in patients with gastrointestinal bleeding admitted to the intensive care unit based on extreme gradient boosting. *Front Med (Lausanne)*. 2023;10:1221602. doi:10.3389/fmed.2023.1221602
947. Shi J, Han H, Chen S, Liu W, Li Y. Machine learning for prediction of acute kidney injury in patients diagnosed with sepsis in critical care. *PLoS One*. 2024;19(4):e0301014. doi:10.1371/journal.pone.0301014
948. Shickel B, Davoudi A, Ozrazgat-Baslanti T, Ruppert M, Bihorac A, Rashidi P. Deep Multi-Modal Transfer Learning for Augmented Patient Acuity Assessment in the Intelligent ICU. *Front Digit Health*. 2021;3doi:10.3389/fdgth.2021.640685
949. Shickel B, Silva B, Ozrazgat-Baslanti T, et al. Multi-dimensional patient acuity estimation with longitudinal EHR tokenization and flexible transformer networks. *Front digit health*. 2022;4:1029191. doi:10.3389/fdgth.2022.1029191
950. Shin Y, Tae Y, Lee Y. Improving Mortality Prediction in ICU by Learning Long-Tailed Population of Patients Through Graph-Domain Aggregation. *IEEE Access*. 2022;10:84405-84416. doi:10.1109/access.2022.3197297
951. Shin Y, Cho KJ, Chang M, et al. The development and validation of a novel deep-learning algorithm to predict in-hospital cardiac arrest in ED-ICU (emergency department-based intensive care units): a single center retrospective cohort study. *Signa Vitae*. 2024;20(4):83-98. doi:10.22514/sv.2024.045
952. Shoeibi F, Najafiaghdam E, Ebrahimi A. Nonlinear features of photoplethysmography signals for Non-invasive blood pressure estimation. *Biomed Signal Process Control*. 2023;85doi:10.1016/j.bspc.2023.105067
953. Shung D, Huang J, Castro E, et al. Neural network predicts need for red blood cell transfusion for patients with acute gastrointestinal bleeding admitted to the intensive care unit. *Sci Rep*. 2021;11(1):8827. doi:10.1038/s41598-021-88226-3
954. Si Y, Wang J, Xu H, Roberts K. Enhancing clinical concept extraction with contextual embeddings. *J Am Med Inform Assoc*. 2019;26(11):1297-1304. doi:10.1093/jamia/ocz096
955. Sikora A, Zhao B, Kong Y, Murray B, Shen Y. Machine learning based prediction of prolonged duration of mechanical ventilation incorporating medication data. *medRxiv*. 2023;doi:10.1101/2023.09.18.23295724
956. Sikora A, Zhang T, Murphy DJ, et al. Machine learning vs. traditional regression analysis for fluid overload prediction in the ICU. *Sci Rep*. 2023;13(1):19654. doi:10.1038/s41598-023-46735-3
957. Sikora A, Rafiei A, Rad MG, et al. Pharmacophenotype identification of intensive care unit medications using unsupervised cluster analysis of the ICURx common data model. *Crit Care*. 2023;27(1):167. doi:10.1186/s13054-023-04437-2
958. Silva JF, Matos S. Modelling patient trajectories using multimodal information. *J Biomed Inform*. 2022;134:104195. doi:10.1016/j.jbi.2022.104195
959. Silva DO, de Souza PN, de Araujo Sousa ML, et al. Impact on the ability of healthcare professionals to correctly identify patient-ventilator asynchronies of the simultaneous visualization of estimated muscle pressure curves on the ventilator display: a randomized study (Pmus study). *Crit Care*. 2023;27(1):128. doi:10.1186/s13054-023-04414-9
960. Caires Silveira E, Mattos Pretti S, Santos BA, Santos Correa CF, Madureira Silva L, Freire de Melo F. Prediction of hospital mortality in intensive care unit patients from clinical and laboratory data: A machine learning approach. *World j crit care med*. 2022;11(5):317-329. doi:10.5492/wjccm.v11.i5.317
961. Sin P, Hokynkova A, Marie N, Andrea P, Krc R, Podrouzek J. Machine Learning-Based Pressure Ulcer Prediction in Modular Critical Care Data. *Diagnostics (Basel)*. 2022;12(4)doi:10.3390/diagnostics12040850
962. Singh J, Sato M, Ohkuma T. On Missingness Features in Machine Learning Models for Critical Care: Observational Study. *JMIR Med Inform*. 2021;9(12):e25022. doi:10.2196/25022

963. Singh YV, Singh P, Khan S, Singh RS. A Machine Learning Model for Early Prediction and Detection of Sepsis in Intensive Care Unit Patients. *J Healthc Eng*. 2022;2022:9263391. doi:10.1155/2022/9263391
964. Singhal L, Garg Y, Yang P, et al. eARDS: A multi-center validation of an interpretable machine learning algorithm of early onset Acute Respiratory Distress Syndrome (ARDS) among critically ill adults with COVID-19. *PLoS One*. 2021;16(9):e0257056. doi:10.1371/journal.pone.0257056
965. Siu BMK, Kwak GH, Ling L, Hui P. Predicting the need for intubation in the first 24 h after critical care admission using machine learning approaches. *Sci Rep*. 2020;10(1):20931. doi:10.1038/s41598-020-77893-3
966. Siuba MT, Bulgarelli L, Duggal A, et al. Differential Effect of Positive End-Expiratory Pressure Strategies in Patients With ARDS: A Bayesian Analysis of Clinical Subphenotypes. *Chest*. Oct 2024;166(4):754-764. doi:S0012-3692(24)00630-5 [pii]
10.1016/j.chest.2024.04.011
967. Sjoding MW, Taylor D, Motyka J, et al. Deep learning to detect acute respiratory distress syndrome on chest radiographs: a retrospective study with external validation. *Lancet Digit Health*. 2021;3(6):e340-e348. doi:10.1016/s2589-7500(21)00056-x
968. Smit JM, Krijthe JH, Endeman H, et al. Dynamic prediction of mortality in COVID-19 patients in the intensive care unit: A retrospective multi-center cohort study. *Intell Based Med*. 2022;6:100071. doi:10.1016/j.ibmed.2022.100071
969. Soares DM, de Souza Mendes R, Rocco Suassuna JH. Microchip Versus Piezoelectric Point of Care Ultrasonography for Pulmonary and Vena Cava Evaluation in Patients With Acute Kidney Injury. *Kidney Intl Rep*. 2024;9(2):395-400. doi:10.1016/j.ekir.2023.11.019
970. Soliman MM, Marshall C, Kimball JP, et al. Parsimonious waveform-derived features consisting of pulse arrival time and heart rate variability predicts the onset of septic shock. *Biomed Signal Process Control*. 2024;92doi:10.1016/j.bspc.2024.105974
971. Sottile PD, Smith B, Moss M, Albers DJ. The Development, Optimization, and Validation of Four Different Machine Learning Algorithms to Identify Ventilator Dyssynchrony. *medRxiv*. Nov 29 2023;doi:10.1101/2023.11.28.23299134
972. Soundoulounaki S, Sylligardos E, Akoumianaki E, et al. Neural Network-Enabled Identification of Weak Inspiratory Efforts during Pressure Support Ventilation Using Ventilator Waveforms. *J Pers Med*. 2023;13(2)doi:10.3390/jpm13020347
973. Srimedha BC, Raj RN, Mayya V. A Comprehensive Machine Learning Based Pipeline for an Accurate Early Prediction of Sepsis in ICU. *IEEE Access*. 2022;10:105120-105132. doi:10.1109/access.2022.3210575
974. Srivastava S, Rajan V. ExpertNet: A Deep Learning Approach to Combined Risk Modeling and Subtyping in Intensive Care Units. *IEEE j biomed health inform*. 2023;27(10):5076-5086. doi:10.1109/jbhi.2023.3295751
975. Steinmeyer C, Wiese L. Sampling methods and feature selection for mortality prediction with neural networks. *J Biomed Inform*. 2020;111doi:10.1016/j.jbi.2020.103580
976. Stenwig E, Salvi G, Rossi PS, Skjaervold NK. Comparative analysis of explainable machine learning prediction models for hospital mortality. *BMC Med Res Methodol*. 2022;22(1):53. doi:10.1186/s12874-022-01540-w
977. Stenwig E, Salvi G, Salvo Rossi P, Skjaervold NK. Comparison of correctly and incorrectly classified patients for in-hospital mortality prediction in the intensive care unit. *BMC Med Res Methodol*. 2023;23(1):102. doi:10.1186/s12874-023-01921-9
978. Stephens AF, Seman M, Diehl A, et al. ECMO PAL: using deep neural networks for survival prediction in venoarterial extracorporeal membrane oxygenation. *Intensive Care Med*. 2023;49(9):1090-1099. doi:10.1007/s00134-023-07157-x
979. Strickler EAT, Thomas J, Thomas JP, Benjamin B, Shamsuddin R. Exploring a global interpretation mechanism for deep learning networks when predicting sepsis. *Sci Rep*. 2023;13(1):3067. doi:10.1038/s41598-023-30091-3
980. Strodthoff N, Strodthoff C, Becher T, Weiler N, Frerichs I. Inferring Respiratory and Circulatory Parameters from Electrical Impedance Tomography With Deep Recurrent Models. *IEEE j biomed health inform*. 2021;25(8):3105-3111. doi:10.1109/jbhi.2021.3059016
981. Su L, Zhang Z, Zheng F, et al. Five novel clinical phenotypes for critically ill patients with mechanical ventilation in intensive care units: a retrospective and multi database study. *Respir Res*. 2020;21(1):325. doi:10.1186/s12931-020-01588-6
982. Su L, Xu Z, Chang F, et al. Early Prediction of Mortality, Severity, and Length of Stay in the Intensive Care Unit of Sepsis Patients Based on Sepsis 3.0 by Machine Learning Models. *Front Med (Lausanne)*. 2021;8:664966. doi:10.3389/fmed.2021.664966

983. Su Y, Guo C, Zhou S, Li C, Ding N. Early predicting 30-day mortality in sepsis in MIMIC-III by an artificial neural networks model. *Eur J Med Res*. 2022;27(1):294. doi:10.1186/s40001-022-00925-3
984. Su L, Li Y, Liu S, et al. Establishment and Implementation of Potential Fluid Therapy Balance Strategies for ICU Sepsis Patients Based on Reinforcement Learning. *Front Med (Lausanne)*. 2022;9:766447. doi:10.3389/fmed.2022.766447
985. Su B, Li H. Development and validation of models for risk of death in patients with systemic lupus erythematosus admitted to the intensive care unit: a retrospective study. *Clin Rheumatol*. 2023;42(11):2987-2999. doi:10.1007/s10067-023-06701-w
986. Su M, Hu S, Xiong H, Kassis EB, Lehman LH. Counterfactual Sepsis Outcome Prediction Under Dynamic and Time-Varying Treatment Regimes. *AMIA Summits Transl Sci Proc*. 2024;2024:285-294. doi:
987. Su L, Liu S, Yang Y, et al. Positive fluid balance and poor outcomes after initial intensive care unit admission in sepsis resuscitation: a retrospective study. *Arch Med Sci*. 2024;20(2):464-475. doi:10.5114/aoms/172160
988. Sun C, Hong S, Song M, Shang J, Li H. Personalized vital signs control based on continuous action-space reinforcement learning with supervised experience. *Biomed Signal Process Control*. 2021;69doi:10.1016/j.bspc.2021.102847
989. Sun Y, Rashedi N, Vaze V, et al. Predicting Future Occurrence of Acute Hypotensive Episodes Using Noninvasive and Invasive Features. *Mil Med*. 2021;186:445-451. doi:10.1093/milmed/usaa418
990. Sun X, Zhou L, Chang S, Liu Z. Using CNN and HHT to Predict Blood Pressure Level Based on Photoplethysmography and Its Derivatives. *Biosensors (Basel)*. 2021;11(4)doi:10.3390/bios11040120
991. Sun Z, Lu X, Duan H, Li H. Deep Dynamic Patient Similarity Analysis: Model Development and Validation in ICU. *Comput Methods Programs Biomed*. 2022;225:107033. doi:10.1016/j.cmpb.2022.107033
992. Sun R, Wang X, Jiang H, et al. Prediction of 30-day mortality in heart failure patients with hypoxic hepatitis: Development and external validation of an interpretable machine learning model. *Front cardiovasc med*. 2022;9:1035675. doi:10.3389/fcvm.2022.1035675
993. Sun C, Li H, Song M, Cai D, Zhang B, Hong S. Continuous diagnosis and prognosis by controlling the update process of deep neural networks. *Patterns (N Y)*. 2023;4(2):100687. doi:10.1016/j.patter.2023.100687
994. Sun T, Yue X, Zhang G, et al. AKIMLpred: An interpretable machine learning model for predicting acute kidney injury within seven days in critically ill patients based on a prospective cohort study. *Clin Chim Acta*. 2024;559:119705. doi:10.1016/j.cca.2024.119705
995. Sun MX, Yang XB, Niu JH, Gu YF, Wang CT, Zhang WS. A cross-modal clinical prediction system for intensive care unit patient outcome. *Knowledge-Based Syst*. 2024;283doi:10.1016/j.knosys.2023.111160
996. Sung CW, Shieh JS, Chang WT, et al. Machine Learning Analysis of Heart Rate Variability for the Detection of Seizures in Comatose Cardiac Arrest Survivors. *IEEE Access*. 2020;8:160515-160525. doi:10.1109/access.2020.3020742
997. Sung M, Hahn S, Han CH, et al. Event Prediction Model Considering Time and Input Error Using Electronic Medical Records in the Intensive Care Unit: Retrospective Study. *JMIR Med Inform*. 2021;9(11):e26426. doi:10.2196/26426
998. Sushil M, Butte AJ, Schuit E, van Smeden M, Leeuwenberg AM. Cross-institution natural language processing for reliable clinical association studies: a methodological exploration. *J Clin Epidemiol*. 2024;167:111258. doi:10.1016/j.jclinepi.2024.111258
999. Susianti H, Asmoro AA, Sujarwoto, et al. Acute Kidney Injury Prediction Model Using Cystatin-C, Beta-2 Microglobulin, and Neutrophil Gelatinase-Associated Lipocalin Biomarker in Sepsis Patients. *Int J Nephrol Renovascular Dis*. 2024;17:105-112. doi:10.2147/ijnrd.S450901
1000. Tajgardoon M, Cooper GF, King AJ, et al. Modeling physician variability to prioritize relevant medical record information. *JAMIA open*. 2020;3(4):602-610. doi:10.1093/jamiaopen/ooaa058
1001. Takahashi M, Ogura K, Goto T, Hayakawa M. Electrocardiogram monitoring as a predictor of neurological and survival outcomes in patients with out-of-hospital cardiac arrest: a single-center retrospective observational study. *Front Neurol*. 2023;14:1210491. doi:10.3389/fneur.2023.1210491
1002. Takkavatakarn K, Oh W, Chan L, et al. Machine learning derived serum creatinine trajectories in acute kidney injury in critically ill patients with sepsis. *Crit Care*. 2024;28(1):156. doi:10.1186/s13054-024-04935-x

1003. Tamboli D, Chen J, Jotheeswaran KP, Yu D, Aggarwal V. Reinforced Sequential Decision-Making for Sepsis Treatment: The PosNegDM Framework With Mortality Classifier and Transformer. *IEEE J Biomedical Health Informat*. 2024;28(5):3114-3122. doi:10.1109/jbhi.2024.3377214
1004. Tan Y, Huang J, Zhuang J, et al. Identifying acute kidney injury subphenotypes using an outcome-driven deep-learning approach. *J Biomed Inform*. 2023;143:104393. doi:10.1016/j.jbi.2023.104393
1005. Tan Y, Young M, Girish A, et al. Predicting respiratory decompensation in mechanically ventilated adult ICU patients. *Front Physiol*. 2023;14:1125991. doi:10.3389/fphys.2023.1125991
1006. Tan Y, Dede M, Mohanty V, et al. Forecasting acute kidney injury and resource utilization in ICU patients using longitudinal, multimodal models. *J Biomed Inform*. 2024;154:104648. doi:10.1016/j.jbi.2024.104648
1007. Tanaka K, Nakada TA, Takahashi N, et al. Superiority of Supervised Machine Learning on Reading Chest X-Rays in Intensive Care Units. *Front Med (Lausanne)*. 2021;8:676277. doi:10.3389/fmed.2021.676277
1008. Tang S, Davarmanesh P, Song Y, Koutra D, Sjoding MW, Wiens J. Democratizing EHR analyses with FIDDLE: a flexible data-driven preprocessing pipeline for structured clinical data. *J Am Med Inform Assoc*. 2020;27(12):1921-1934. doi:10.1093/jamia/ocaa139
1009. Tang WJ, Ma JQ, Waljee AK, Zhu J. Semi-supervised joint learning for longitudinal clinical events classification using neural network models. *Stat*. 2020;9(1)doi:10.1002/sta4.305
1010. Tang QF, Chen ZC, Guo YK, et al. Robust Reconstruction of Electrocardiogram Using Photoplethysmography: A Subject-Based Model. *Front Physiol*. 2022;13doi:10.3389/fphys.2022.859763
1011. Tang CHM, Seah JCY, Ahmad HK, et al. Analysis of Line and Tube Detection Performance of a Chest X-ray Deep Learning Model to Evaluate Hidden Stratification. *Diagnostics (Basel)*. Jul 9 2023;13(14)doi:diagnostics13142317 [pii]
diagnostics-13-02317 [pii]
10.3390/diagnostics13142317
1012. Tang Y, Zhang Y, Li J. A time series driven model for early sepsis prediction based on transformer module. *BMC Med Res Methodol*. 2024;24(1):23. doi:10.1186/s12874-023-02138-6
1013. Tang D, Ma C, Xu Y. Interpretable machine learning model for early prediction of delirium in elderly patients following intensive care unit admission: a derivation and validation study. *Front Med (Lausanne)*. 2024;11:1399848. doi:10.3389/fmed.2024.1399848
1014. Tang J, Huang J, He X, et al. The prediction of in-hospital mortality in elderly patients with sepsis-associated acute kidney injury utilizing machine learning models. *Heliyon*. 2024;10(4):e26570. doi:10.1016/j.heliyon.2024.e26570
1015. Tao T, Lu S, Hu N, et al. Prognosis of comatose patients with reduced EEG montage by combining quantitative EEG features in various domains. *Front Neurosci*. 2023;17doi:10.3389/fnins.2023.1302318
1016. Tardiveau C, Monneret G, Lukaszewicz AC, et al. A 9-mRNA signature measured from whole blood by a prototype PCR panel predicts 28-day mortality upon admission of critically ill COVID-19 patients. *Front Immunol*. 2022;13:1022750. doi:10.3389/fimmu.2022.1022750
1017. Tariq A, Su L, Patel B, Banerjee I. Prediction of Transfusion among In-patient Population using Temporal Pattern based Clinical Similarity Graphs. *AMIA Annu Symp Proc*. 2023;2023:679-688. doi:
1018. Tasken AA, Yu J, Berg EAR, et al. Automatic Detection and Tracking of Anatomical Landmarks in Transesophageal Echocardiography for Quantification of Left Ventricular Function. *Ultrasound Med Biol*. 2024;50(6):797-804. doi:10.1016/j.ultrasmedbio.2024.01.017
1019. Tasnim N, Al Mamun S, Islam MS, Kaiser MS, Mahmud M. Explainable Mortality Prediction Model for Congestive Heart Failure with Nature-Based Feature Selection Method. *Appl Sci -Basel*. 2023;13(10)doi:10.3390/app13106138
1020. Tavakolian A, Rezaee A, Hajati F, Uddin S. Hospital Readmission and Length-of-Stay Prediction Using an Optimized Hybrid Deep Model. *Future Internet*. 2023;15(9)doi:10.3390/fi15090304
1021. Tehrany PM, Zabihi MR, Ghorbani Vajargah P, et al. Risk predictions of hospital-acquired pressure injury in the intensive care unit based on a machine learning algorithm. *Int Wound J*. 2023;doi:10.1111/iwj.14275
1022. Tewarie PKB, Beernink TMJ, Eertman-Meyer CJ, et al. Early EEG monitoring predicts clinical outcome in patients with moderate to severe traumatic brain injury. *Neuroimage (Amst)*. 2023;37:103350. doi:10.1016/j.nicl.2023.103350
1023. Thoral PJ, Fornasa M, de Bruin DP, et al. Explainable Machine Learning on AmsterdamUMCdb for ICU Discharge Decision Support: Uniting Intensivists and Data Scientists. *Crit care explor*. 2021;3(9):e0529. doi:10.1097/cce.0000000000000529

1024. Thorsen-Meyer HC, Nielsen AB, Nielsen AP, et al. Dynamic and explainable machine learning prediction of mortality in patients in the intensive care unit: a retrospective study of high-frequency data in electronic patient records. *Lancet Digit Health*. 2020;2(4):e179-e191.
1025. Thorsen-Meyer HC, Placido D, Kaas-Hansen BS, et al. Discrete-time survival analysis in the critically ill: a deep learning approach using heterogeneous data. *NPJ Digit Med*. 2022;5(1):142. doi:10.1038/s41746-022-00679-6
1026. Tian J, Zhou Y, Liu H, Qu Z, Zhang L, Liu L. Quantitative EEG parameters can improve the predictive value of the non-traumatic neurological ICU patient prognosis through the machine learning method. *Front Neurol*. 2022;13:897734. doi:10.3389/fneur.2022.897734
1027. Tian Y, Liang Y, Chen Y, Bian H. Analysis of delirium prediction in the ICU based on the hybrid SGDCS-ANFIS approach. *Med Biol Eng Comput*. 2023;61(3):673-683. doi:10.1007/s11517-022-02741-7
1028. Tian J, Cui R, Song H, Zhao Y, Zhou T. Prediction of acute kidney injury in patients with liver cirrhosis using machine learning models: evidence from the MIMIC-III and MIMIC-IV. *Int Urol Nephrol*. 2023;doi:10.1007/s11255-023-03646-6
1029. Wu TS, Chen SY, Tian YM, Wu P. A Feature Optimized Deep Learning Model for Clinical Data Mining. *Chin J Electron*. 2020;29(3):476-481. doi:10.1049/cje.2020.03.004
1030. Toh EMS, Yan B, Lim IC, et al. The role of intracranial pressure variability as a predictor of intracranial hypertension and mortality in critically ill patients. *J Neurosurg*. 2023:1-8. doi:10.3171/2023.4.Jns23123
1031. Tol JTM, Terwindt LE, Rellum SR, et al. Performance of a Machine Learning Algorithm to Predict Hypotension in Spontaneously Breathing Non-Ventilated Post-Anesthesia and ICU Patients. *J Pers Med*. Feb 15 2024;14(2)doi:jpm14020210 [pii]
jpm-14-00210 [pii]
10.3390/jpm14020210
1032. Toledo LV, Bhering LL, Ercole FF. Artificial intelligence to predict bed bath time in Intensive Care Units. *Rev Bras Enferm*. 2024;77(1):e20230201. doi:10.1590/0034-7167-2023-0201
1033. Tootooni MS, Barreto EF, Wutthisirisart P, Kashani KB, Pasupathy KS. Determining steady-state trough range in vancomycin drug dosing using machine learning. *J Crit Care*. 2024;82:154784. doi:10.1016/j.jcrc.2024.154784
1034. Tran A, Topp R, Tarshizi E, Shao A. Predicting the Onset of Sepsis Using Vital Signs Data: A Machine Learning Approach. *Clin Nurs Res*. 2023:10547738231183207. doi:10.1177/10547738231183207
1035. Triana AJ, Vyas R, Shah AS, Tiwari V. Predicting Length of Stay of Coronary Artery Bypass Grafting Patients Using Machine Learning. *J Surg Res*. 2021;264:68-75. doi:10.1016/j.jss.2021.02.003
1036. Tsai LW, Yuan KC, Hou SK, et al. Determining Carina and Clavicular Distance-Dependent Positioning of Endotracheal Tube in Critically Ill Patients: An Artificial Intelligence-Based Approach. *Biology-Basel*. 2022;11(4)doi:10.3390/biology11040490
1037. Tsai WC, Tsai YC, Kuo KC, et al. Natural language processing and network analysis in patients withdrawing from life-sustaining treatments: a retrospective cohort study. *BMC Palliat Care*. 2022;21(1):225. doi:10.1186/s12904-022-01119-8
1038. Tsiklidis EJ, Sinno T, Diamond SL. Predicting risk for trauma patients using static and dynamic information from the MIMIC III database. *PLoS One*. 2022;17(1):e0262523. doi:10.1371/journal.pone.0262523
1039. Tu KC, Tau ENT, Chen NC, et al. Machine Learning Algorithm Predicts Mortality Risk in Intensive Care Unit for Patients with Traumatic Brain Injury. *Diagnostics (Basel)*. Sep 21 2023;13(18)doi:diagnostics13183016 [pii]
diagnostics-13-03016 [pii]
10.3390/diagnostics13183016
1040. Urner M, Mitsakakis N, Vorona S, et al. Identifying subjects at risk for diaphragm atrophy during mechanical ventilation using routinely available clinical data. *Respir Care*. 2021;66(4):551-558. doi:10.4187/respcare.08223
1041. Vagliano I, Lvova O, Schut MC. Interpretable and Continuous Prediction of Acute Kidney Injury in the Intensive Care. *Stud Health Technol Inform*. 2021;281:103-107. doi:10.3233/shti210129
1042. Vagliano I, Schut MC, Abu-Hanna A, et al. Assess and validate predictive performance of models for in-hospital mortality in COVID-19 patients: A retrospective cohort study in the Netherlands comparing the value of registry data with high-granular electronic health records. *Int J Med Inf*. 2022;167:104863. doi:10.1016/j.ijmedinf.2022.104863

1043. Vagliano I, Brinkman S, Abu-Hanna A, et al. Can we reliably automate clinical prognostic modelling? A retrospective cohort study for ICU triage prediction of in-hospital mortality of COVID-19 patients in the Netherlands. *Int J Med Inf*. 2022;160:104688. doi:10.1016/j.ijmedinf.2022.104688
1044. Vagliano I, Byrne Salsas C, Wunn T, Schut MC. External Validation and Transportability of Models to Predict Acute Kidney Injury in the Intensive Care Unit. *Stud Health Technol Inform*. 2022;295:148-151. doi:10.3233/shti220683
1045. Vagliano I, Hsu WH, Schut MC. Machine Learning, Clinical Notes and Knowledge Graphs for Early Prediction of Acute Kidney Injury in the Intensive Care. *Stud Health Technol Inform*. 2022;289:329-332. doi:10.3233/shti210926
1046. Vagliano I, Kingma MY, Dongelmans DA, de Lange DW, de Keizer NF, Schut MC. Automated identification of patient subgroups: A case-study on mortality of COVID-19 patients admitted to the ICU. *Comput Biol Med*. 2023;163:107146. doi:10.1016/j.compbiomed.2023.107146
1047. Vaid A, Chan L, Chaudhary K, et al. Predictive approaches for acute dialysis requirement and death in COVID-19. *Clin J Am Soc Nephrol*. 2021;16(8):1158-1168. doi:10.2215/cjn.17311120
1048. Valderrama CE, Niven DJ, Stelfox HT, Lee J. Predicting Abnormal Laboratory Blood Test Results in the Intensive Care Unit Using Novel Features Based on Information Theory and Historical Conditional Probability: Observational Study. *JMIR Med Inform*. 2022;10(6):e35250. doi:10.2196/35250
1049. van de Kamp LV, Reinders J, Hunnekens B, Oomen T, Wouw NV. Automatic patient-ventilator asynchrony detection framework using objective asynchrony definitions. *IFAC J Syst Control*. 2024;27doi:10.1016/j.ifacsc.2023.100236
1050. van de Sande D, van Genderen ME, Rosman B, et al. Predicting thromboembolic complications in COVID-19 ICU patients using machine learning. *J clin trans res*. 2020;6(4):179-186. doi:
1051. van der Ven WH, Terwindt LE, Risvanoglu N, et al. Performance of a machine-learning algorithm to predict hypotension in mechanically ventilated patients with COVID-19 admitted to the intensive care unit: a cohort study. *J Clin Monit Comput*. 2022;36(5):1397-1405. doi:10.1007/s10877-021-00778-x
1052. Varudo R, Gonzalez FA, Leote J, et al. Machine learning for the real-time assessment of left ventricular ejection fraction in critically ill patients: a bedside evaluation by novices and experts in echocardiography. *Crit Care*. 2022;26(1):386. doi:10.1186/s13054-022-04269-6
1053. Vázquez B, Fuentes-Pineda G, García F, Borrayo G, Prohías J. Risk markers by sex for in-hospital mortality in patients with acute coronary syndrome: A machine learning approach. *Inform Med Unlocked*. 2021;27doi:10.1016/j.imu.2021.100791
1054. Venturini M, Van Keilegom I, De Corte W, Vens C. Predicting time-to-intubation after critical care admission using machine learning and cured fraction information. *Artif Intell Med*. 2024;150:102817. doi:10.1016/j.artmed.2024.102817
1055. Verhaeghe J, Dhaese SAM, De Corte T, et al. Development and evaluation of uncertainty quantifying machine learning models to predict piperacillin plasma concentrations in critically ill patients. *BMC Med Inf Decis Mak*. 2022;22(1):224. doi:10.1186/s12911-022-01970-y
1056. Verhaeghe J, De Corte T, Sauer CM, et al. Generalizable calibrated machine learning models for real-time atrial fibrillation risk prediction in ICU patients. *Int J Med Inf*. 2023;175:105086. doi:10.1016/j.ijmedinf.2023.105086
1057. Verma R, Maheshwari S, Shukla A. Feature engineering combined with 1-D convolutional neural network for improved mortality prediction. *Bio-Algorithms Med-Syst*. 2020;16(4)doi:10.1515/bams-2020-0056
1058. Vigia E, Ramalhete L, Ribeiro R, et al. Pancreas Rejection in the Artificial Intelligence Era: New Tool for Signal Patients at Risk. *J Pers Med*. Jun 29 2023;13(7)doi:jpm13071071 [pii]
jpm-13-01071 [pii]
10.3390/jpm13071071
1059. Vigneron C, Laousy O, Chassagnon G, et al. Assessment of Functional and Nutritional Status and Skeletal Muscle Mass for the Prognosis of Critically Ill Solid Cancer Patients. *Cancers (Basel)*. 2022;14(23)doi:10.3390/cancers14235870
1060. Villar J, Gonzalez-Martin JM, Hern, et al. Predicting ICU Mortality in Acute Respiratory Distress Syndrome Patients Using Machine Learning: The Predicting Outcome and STratifiCation of severity in ARDS (POSTCARDS) Study. *Crit Care Med*. 2023;51(12):1638-1649. doi:10.1097/ccm.0000000000006030
1061. Villar J, González-Martín JM, Fernández C, et al. Predicting the Length of Mechanical Ventilation in Acute Respiratory Disease Syndrome Using Machine Learning: The PIONEER Study. *J Clin Med*. 2024;13(6)doi:10.3390/jcm13061811

1062. Vornhagen J, Rao K, Bachman MA. Gut community structure as a risk factor for infection in Klebsiella -colonized patients. *medRxiv*. 2023;doi:10.1101/2023.04.18.23288742
1063. Wang H, Bi Y. Building Deep Learning Models to Predict Mortality in ICU Patients. *arXiv preprint arXiv:2012 07585*. 2020;doi:
1064. Wang T, Tian Y, Qiu RG. Long short-term memory recurrent neural networks for multiple diseases risk prediction by leveraging longitudinal medical records. *IEEE J Biomedical Health Informat*. 2020;24(8):2337-2346. doi:10.1109/jbhi.2019.2962366
1065. Wang B, Ding S, Liu X, Li X, Li G. Predictive classification of ICU readmission using weight decay random forest. *Futur Gener Comp Syst*. 2021;124:351-360. doi:10.1016/j.future.2021.06.011
1066. Wang Y, Wei Y, Yang H, Li J, Zhou Y, Wu Q. Utilizing imbalanced electronic health records to predict acute kidney injury by ensemble learning and time series model. *BMC Med Inf Decis Mak*. 2020;20(1):238. doi:10.1186/s12911-020-01245-4
1067. Wang D, Li J, Sun Y, et al. A Machine Learning Model for Accurate Prediction of Sepsis in ICU Patients. *Front public health*. 2021;9:754348. doi:10.3389/fpubh.2021.754348
1068. Wang T, Chen Y, Du H, Liu Y, Zhang L, Meng M. Monitoring of Neuroendocrine Changes in Acute Stage of Severe Craniocerebral Injury by Transcranial Doppler Ultrasound Image Features Based on Artificial Intelligence Algorithm. *Comp Math Methods Med*. 2021;2021doi:10.1155/2021/3584034
1069. Wang H, Bao Q, Cao D, Dong S, Wu L. Characteristics of Computed Tomography Images for Patients with Acute Liver Injury Caused by Sepsis under Deep Learning Algorithm. *Contrast Media Mol Imaging*. 2022;2022:9322196. doi:10.1155/2022/9322196
1070. Wang H, Li Y, Naidech A, Luo Y. Comparison between machine learning methods for mortality prediction for sepsis patients with different social determinants. *BMC Med Inf Decis Mak*. 2022;22:156. doi:10.1186/s12911-022-01871-0
1071. Wang K, Yan LZ, Li WZ, et al. Comparison of Four Machine Learning Techniques for Prediction of Intensive Care Unit Length of Stay in Heart Transplantation Patients. *Front cardiovasc med*. 2022;9:863642. doi:10.3389/fcvm.2022.863642
1072. Wang Z, Lan Y, Xu Z, Gu Y, Li J. Comparison of Mortality Predictive Models of Sepsis Patients Based on Machine Learning. *Chin Med Sci J*. 2022;37(3):201-210. doi:10.24920/004102
1073. Wang Z, Zhang L, Huang T, et al. Developing an explainable machine learning model to predict the mechanical ventilation duration of patients with ARDS in intensive care units. *Heart Lung*. 2023;58:74-81. doi:10.1016/j.hrtlng.2022.11.005
1074. Wang Y, Li C, Yuan M, et al. Development of a complete blood count with differential—based prediction model for in-hospital mortality among patients with acute myocardial infarction in the coronary care unit. *Front Cardiovasc Med*. 2022;9doi:10.3389/fcvm.2022.1001356
1075. Wang H, Zhao QY, Luo JC, et al. Early prediction of noninvasive ventilation failure after extubation: development and validation of a machine-learning model. *BMC polm med*. 2022;22(1):304. doi:10.1186/s12890-022-02096-7
1076. Wang P, Xu J, Wang C, Zhang G, Wang H. Method of non-invasive parameters for predicting the probability of early in-hospital death of patients in intensive care unit. *Biomed Signal Process Control*. 2022;73doi:10.1016/j.bspc.2021.103405
1077. Wang Z, Yao B. Multi-Branching Temporal Convolutional Network for Sepsis Prediction. *IEEE J Biomedical Health Informat*. 2022;26(2):876-887. doi:10.1109/jbhi.2021.3092835
1078. Wang X, Zhu T, Xia M, et al. Predicting the Prognosis of Patients in the Coronary Care Unit: A Novel Multi-Category Machine Learning Model Using XGBoost. *Front Cardiovasc Med*. 2022;9doi:10.3389/fcvm.2022.764629
1079. Wang Y, Deng Y, Tan Y, Zhou M, Jiang Y, Liu B. A comparison of random survival forest and Cox regression for prediction of mortality in patients with hemorrhagic stroke. *BMC Med Inf Decis Mak*. 2023;23(1):215. doi:10.1186/s12911-023-02293-2
1080. Wang S, Li J, Wang Q, et al. A data-driven medical knowledge discovery framework to predict the length of ICU stay for patients undergoing craniotomy based on electronic medical records. *Math Biosci Eng*. 2023;20(1):837-858. doi:10.3934/mbe.2023038
1081. Wang Y, Wang G, Zhao Y, et al. A deep learning model for predicting multidrug-resistant organism infection in critically ill patients. *J Intensive Care*. 2023;11(1)doi:10.1186/s40560-023-00695-y
1082. Wang Y, Stroh JN, Hripcsak G, et al. A methodology of phenotyping ICU patients from EHR data: High-fidelity, personalized, and interpretable phenotypes estimation. *J Biomed Inform*. 2023;148:104547. doi:10.1016/j.jbi.2023.104547

1083. Wang Z, Zhang L, Chao Y, Xu M, Geng X, Hu X. Development of a Machine Learning Model for Predicting 28-Day Mortality of Septic Patients with Atrial Fibrillation. *Shock*. 2023;59(3):400-408. doi:10.1097/shk.0000000000002078
1084. Wang ML, Kuo YT, Kuo LC, et al. Early prediction of delirium upon intensive care unit admission: Model development, validation, and deployment. *J Clin Anesth*. 2023;88:111121. doi:10.1016/j.jclinane.2023.111121
1085. Wang L, Zhang Y, Yao R, et al. Identification of distinct clinical phenotypes of cardiogenic shock using machine learning consensus clustering approach. *BMC Cardiovasc Disord*. 2023;23(1):426. doi:10.1186/s12872-023-03380-y
1086. Wang H, Wang C, Xu J, Yuan J, Liu G, Zhang G. Invasive mechanical ventilation probability estimation using machine learning methods based on non-invasive parameters. *Biomed Signal Process Control*. 2023;79doi:10.1016/j.bspc.2022.104193
1087. Wang YX, Li XL, Zhang LH, et al. Machine learning algorithms assist early evaluation of enteral nutrition in ICU patients. *Front nutr*. 2023;10:1060398. doi:10.3389/fnut.2023.1060398
1088. Wang R, Cai L, Liu Y, Zhang J, Ou X, Xu J. Machine learning algorithms for prediction of ventilator associated pneumonia in traumatic brain injury patients from the MIMIC-III database. *Heart Lung*. 2023;62:225-232. doi:10.1016/j.hrtlng.2023.08.002
1089. Wang G, Xu J, Lin X, et al. Machine learning-based models for predicting mortality and acute kidney injury in critical pulmonary embolism. *BMC Cardiovasc Disord*. 2023;23(1):385. doi:10.1186/s12872-023-03363-z
1090. Wang B, Li Y, Tian Y, Ju C, Xu X, Pei S. Novel pneumonia score based on a machine learning model for predicting mortality in pneumonia patients on admission to the intensive care unit. *Respir Med*. 2023;217:107363. doi:10.1016/j.rmed.2023.107363
1091. Wang R, Cai L, Zhang J, He M, Xu J. Prediction of Acute Respiratory Distress Syndrome in Traumatic Brain Injury Patients Based on Machine Learning Algorithms. *Medicina (Kaunas)*. 2023;59(1)doi:10.3390/medicina59010171
1092. Wang R, Zeng X, Long Y, et al. Prediction of Mortality in Geriatric Traumatic Brain Injury Patients Using Machine Learning Algorithms. *Brain Sci*. 2023;13(1)doi:10.3390/brainsci13010094
1093. Wang YC, Lin WY, Tseng YJ, et al. Risk Stratification for Herpes Simplex Virus Pneumonia Using Elastic Net Penalized Cox Proportional Hazard Algorithm with Enhanced Explainability. *J Clin Med*. 2023;12(13)doi:10.3390/jcm12134489
1094. Wang Z, Samsten I, Kougia V, Papapetrou P. Style-transfer counterfactual explanations: An application to mortality prevention of ICU patients. *Artif Intell Med*. 2023;135:102457. doi:10.1016/j.artmed.2022.102457
1095. Wang L, Duan SB, Yan P, Luo XQ, Zhang NY. Utilization of interpretable machine learning model to forecast the risk of major adverse kidney events in elderly patients in critical care. *Ren Fail*. 2023;45(1):2215329. doi:10.1080/0886022x.2023.2215329
1096. Wang MN, Ma JJ, Zhao X, Xing X. Automated Physiological Status Detection and Disease Evaluation of Critically Ill Patients via Image Processing Technologies. *Trait Signal*. 2024;41(1):153-163. doi:10.18280/ts.410112
1097. Wang QY, Sun JN, Liu XF, et al. Comparison of risk prediction models for the progression of pelvic inflammatory disease patients to sepsis: Cox regression model and machine learning model. *Heliyon*. 2024;10(1)doi:10.1016/j.heliyon.2023.e23148
1098. Wang Z, Chao Y, Xu M, Zhao W, Hu X. Machine learning prediction of the failure of high-flow nasal oxygen therapy in patients with acute respiratory failure. *Sci Rep*. 2024;14(1):1825. doi:10.1038/s41598-024-52061-z
1099. Wang L, Long DY. Significant risk factors for intensive care unit-acquired weakness: A processing strategy based on repeated machine learning. *World j clin cases*. 2024;12(7):1235-1242. doi:10.12998/wjcc.v12.i7.1235
1100. Wanyan TY, Honarvar H, Azad A, Ding Y, Glicksberg BS. Deep Learning with Heterogeneous Graph Embeddings for Mortality Prediction from Electronic Health Records. *Data Intell*. 2021;3(3):329-339. doi:10.1162/dint_a_00097
1101. Wanyan T, Vaid A, De Freitas JK, et al. Relational Learning Improves Prediction of Mortality in COVID-19 in the Intensive Care Unit. *IEEE trans big data*. 2021;7(1):38-44. doi:10.1109/tbdata.2020.3048644
1102. Wei C, Zhang L, Feng Y, Ma A, Kang Y. Machine learning model for predicting acute kidney injury progression in critically ill patients. *BMC Med Inf Decis Mak*. 2022;22(1):17. doi:10.1186/s12911-021-01740-2

1103. Wei S, Zhang Y, Dong H, et al. Machine learning-based prediction model of acute kidney injury in patients with acute respiratory distress syndrome. *BMC polm med*. 2023;23(1):370. doi:10.1186/s12890-023-02663-6
1104. Weisman Raymond M, Shahar Y. Provision of Decision Support Through Continuous Prediction of Recurring Clinical Actions. *Stud Health Technol Inform*. 2023;305:200-203. doi:10.3233/shti230462
1105. Wen C, Zhang X, Li Y, et al. An interpretable machine learning model for predicting 28-day mortality in patients with sepsis-associated liver injury. *PLoS One*. 2024;19(5):e0303469. doi:10.1371/journal.pone.0303469
1106. Weng WH, Gao M, He Z, Yan S, Szolovits P. Representation and reinforcement learning for personalized glycemic control in septic patients. *arXiv preprint arXiv …*. 2017;doi:
1107. Wernly B, Mam, ipoor B, Baldia P, Jung C, Osmani V. Machine learning predicts mortality in septic patients using only routinely available ABG variables: a multi-centre evaluation. *Int J Med Inf*. 2021;145:104312. doi:10.1016/j.ijmedinf.2020.104312
1108. Whitlock KC, ala M, Bishop KL, Moll V, Sharp JJ, Krishnan S. Lower AM-PAC 6-Clicks Basic Mobility Score Predicts Discharge to a Postacute Care Facility Among Patients in Cardiac Intensive Care Units. *Phys Ther*. 2022;102(1)doi:10.1093/ptj/pzab252
1109. Wieringa A, Ewoldt TMJ, Gangapersad RN, et al. Predicting Beta-Lactam Target Non-Attainment in ICU Patients at Treatment Initiation: Development and External Validation of Three Novel (Machine Learning) Models. *Antibiotics*. 2023;12(12)doi:10.3390/antibiotics12121674
1110. Woillard JB, Salmon G, onniere C, et al. A Machine Learning Approach to Estimate the Glomerular Filtration Rate in Intensive Care Unit Patients Based on Plasma Iohexol Concentrations and Covariates. *Clin Pharmacokinet*. 2021;60(2):223-233. doi:10.1007/s40262-020-00927-6
1111. Wollborn J, Hassenzahl LO, Reker D, et al. Diagnosing capillary leak in critically ill patients: development of an innovative scoring instrument for non-invasive detection. *Ann Intensive Care*. 2021;11(1):175. doi:10.1186/s13613-021-00965-8
1112. Wollborn J, Zhang Z, Gaa J, et al. Angiopoietin-2 is associated with capillary leak and predicts complications after cardiac surgery. *Ann Intensive Care*. 2023;13(1)doi:10.1186/s13613-023-01165-2
1113. Wong KP, Homer SY, Wei SH, et al. Integration and evaluation of chest X-ray artificial intelligence in clinical practice. *J Med Imaging (Bellingham)*. 2023;10(5):051805. doi:10.1117/1.Jmi.10.5.051805
1114. Wu W, Zhou Z. A Comprehensive Way to Access Hospital Death Prediction Model for Acute Mesenteric Ischemia: A Combination of Traditional Statistics and Machine Learning. *Int J Gen Med*. 2021;14:591-602. doi:10.2147/ijgm.S300492
1115. Wu J, Lin Y, Li P, Hu Y, Zhang L, Kong G. Predicting Prolonged Length of ICU Stay through Machine Learning. *Diagnostics (Basel)*. 2021;11(12)doi:10.3390/diagnostics11122242
1116. Wu Y, Huang S, Chang X. Understanding the complexity of sepsis mortality prediction via rule discovery and analysis: a pilot study. *BMC Med Inf Decis Mak*. 2021;21(1):334. doi:10.1186/s12911-021-01690-9
1117. Wu CL, Liu SF, Yu TL, et al. Deep Learning-Based Pain Classifier Based on the Facial Expression in Critically Ill Patients. *Front Med*. 2022;9doi:10.3389/fmed.2022.851690
1118. Wu J, Liu C, Xie L, et al. Early prediction of moderate-to-severe condition of inhalation-induced acute respiratory distress syndrome via interpretable machine learning. *BMC polm med*. 2022;22(1):193. doi:10.1186/s12890-022-01963-7
1119. Wu R, Smith A, Brown T, et al. Deterioration Index in Critically Injured Patients: A Feasibility Analysis. *J Surg Res*. 2023;281:45-51. doi:10.1016/j.jss.2022.08.019
1120. Wu W, Wang Y, Tang J, Yu M, Yuan J, Zhang G. Developing and evaluating a machine-learning-based algorithm to predict the incidence and severity of ARDS with continuous non-invasive parameters from ordinary monitors and ventilators. *Comput Methods Programs Biomed*. 2023;230:107328. doi:10.1016/j.cmpb.2022.107328
1121. Wu M, Jiang X, Du K, Xu Y, Zhang W. Ensemble machine learning algorithm for predicting acute kidney injury in patients admitted to the neurointensive care unit following brain surgery. *Sci Rep*. 2023;13(1):6705. doi:10.1038/s41598-023-33930-5
1122. Wu T, Wei Y, Wu J, Yi B, Li H. Logistic regression technique is comparable to complex machine learning algorithms in predicting cognitive impairment related to post intensive care syndrome. *Sci Rep*. 2023;13(1):2485. doi:10.1038/s41598-023-28421-6
1123. Wu L, Li Y, Zhang X, et al. Prediction differences and implications of acute kidney injury with and without urine output criteria in adult critically ill patients. *Nephrol Dial Transplant*. 2023;doi:10.1093/ndt/gfad065

1124. Wu CW, Pham BT, Wang JC, Wu YK, Kuo CY, Hsu YC. The COVIDTW study: Clinical predictors of COVID-19 mortality and a novel AI prognostic model using chest X-ray. *J Formos Med Assoc*. 2023;122(3):267-275. doi:10.1016/j.jfma.2022.09.014
1125. Wu Q, Ye F, Gu Q, et al. A customised down-sampling machine learning approach for sepsis prediction. *Int J Med Inf*. 2024;184:105365. doi:10.1016/j.ijmedinf.2024.105365
1126. Wu YA, Rocha BM, Kaimakamis E, et al. A deep learning method for predicting the COVID-19 ICU patient outcome fusing X-rays, respiratory sounds, and ICU parameters. *Expert Syst Appl*. 2024;235doi:10.1016/j.eswa.2023.121089
1127. Wu JC, Liao NC, Yang TH, et al. Deep-Learning-Based Automated Anomaly Detection of EEGs in Intensive Care Units. *Bioengineering (Basel)*. Apr 25 2024;11(5)doi:bioengineering11050421 [pii]
bioengineering-11-00421 [pii]
10.3390/bioengineering11050421
1128. Wu XD, Li RC, He Z, Yu TZ, Cheng CQ. A value-based deep reinforcement learning model with human expertise in optimal treatment of sepsis. *NPJ Digit Med*. 2023;6(1)doi:10.1038/s41746-023-00755-5
1129. Xia J, Pan S, Zhu M, Cai G, Yan M, Su Q. A long short-term memory ensemble approach for improving the outcome prediction in intensive care unit. *Comput Math Methods Med*. 2019;2019:8152713. doi:10.1155/2019/8152713
1130. Xia N, Chen J, Zhan C, et al. Prediction of Clinical Outcome at Discharge After Rupture of Anterior Communicating Artery Aneurysm Using the Random Forest Technique. *Front Neurol*. 2020;11doi:10.3389/fneur.2020.538052
1131. Xia M, Jin C, Cao S, et al. Development and validation of a machine-learning model for prediction of hypoxemia after extubation in intensive care units. *Ann transl med*. 2022;10(10):577. doi:10.21037/atm-22-2118
1132. Xian CQ, de Souza CPE, Rodrigues FF. Health outcome predictive modelling in intensive care units. *Oper Res Health Care*. 2023;39doi:10.1016/j.orhc.2023.100409
1133. Xiao Z, Zeng L, Chen S, Wu J, Huang H. Development and validation of early prediction models for new-onset functional impairment in patients after being transferred from the ICU. *Sci Rep*. 2024;14(1):11902. doi:10.1038/s41598-024-62447-8
1134. Xie F, Chakraborty B, Ong MEH, Goldstein BA, Liu N. AutoScore: A Machine Learning-Based Automatic Clinical Score Generator and Its Application to Mortality Prediction Using Electronic Health Records. *JMIR Med Inform*. 2020;8(10):e21798. doi:10.2196/21798
1135. Xie F, Ning Y, Yuan H, et al. AutoScore-Survival: Developing interpretable machine learning-based time-to-event scores with right-censored survival data. *J Biomed Inform*. 2022;125:103959. doi:10.1016/j.jbi.2021.103959
1136. Xie W, Li Y, Meng X, Zhao M. Machine learning prediction models and nomogram to predict the risk of in-hospital death for severe DKA: A clinical study based on MIMIC-IV, eICU databases, and a college hospital ICU. *Int J Med Inf*. 2023;174:105049. doi:10.1016/j.ijmedinf.2023.105049
1137. Xie P, Wang H, Xiao J, et al. Development and Validation of an Explainable Deep Learning Model to Predict In-Hospital Mortality for Patients With Acute Myocardial Infarction: Algorithm Development and Validation Study. *J Med Internet Res*. 2024;26:e49848. doi:10.2196/49848
1138. Xinsai L, Zhengye W, Xuan H, et al. Prediction model of acute kidney injury after different types of acute aortic dissection based on machine learning. *Front Cardiovasc Med*. 2022;9doi:10.3389/fcvm.2022.984772
1139. Xu Y, Biswal S, Deshpande SR, Maher KO. Raim: Recurrent attentive and intensive model of multimodal patient monitoring data. *Proceedings of the 24th …*. 2018;doi:10.1145/3219819.3220051
1140. Xu J, Chen X, Zheng X. Acinetobacter baumannii complex-caused bloodstream infection in ICU during a 12-year period: Predicting fulminant sepsis by interpretable machine learning. *Front Microbiol*. 2022;13:1037735. doi:10.3389/fmicb.2022.1037735
1141. Xu Y, Han D, Huang T, et al. Predicting ICU Mortality in Rheumatic Heart Disease: Comparison of XGBoost and Logistic Regression. *Front cardiovasc med*. 2022;9:847206. doi:10.3389/fcvm.2022.847206
1142. Xu S, Cai J, Doomi A, Shi J. A prognostic assessment predicated by blood culture-based bacteria clustering from real-world evidence: Novel strategies and perspectives on prevention and management of sepsis. *Front mol biosci*. 2023;10:1160146. doi:10.3389/fmolb.2023.1160146
1143. Xu J, Hu Z, Miao J, et al. Machine Learning for Predicting Hemodynamic Deterioration of Patients with Intermediate-Risk Pulmonary Embolism in Intensive Care Unit. *Shock*. 2024;61(1):68-75. doi:10.1097/shk.0000000000002261

1144. Xu J, Chen T, Fang X, Xia L, Pan X. Prediction model of pressure injury occurrence in diabetic patients during ICU hospitalization--XGBoost machine learning model can be interpreted based on SHAP. *Intensive Crit Care Nurs*. 2024;83:103715. doi:10.1016/j.iccn.2024.103715
1145. Xue Y, Klabjan D, Luo Y. Predicting ICU readmission using grouped physiological and medication trends. *Artif Intell Med*. 2019;95:27-37. doi:10.1016/j.artmed.2018.08.004
1146. Yadav SS, Jadhav SM. Detection of common risk factors for diagnosis of cardiac arrhythmia using machine learning algorithm. *Expert Syst Appl*. 2021;163doi:10.1016/j.eswa.2020.113807
1147. Yallabandi G, Jeganathan J, Mayya V, Kamath SS. ICU Patients' Pattern Recognition and Correlation Identification of Vital Parameters Using Optimized Machine Learning Models. *Int J Electr Comput Eng Syst*. 2023;14(9):1003-1013. doi:
1148. Yamao Y, Oami T, Yamabe J, Takahashi N, Nakada TA. Machine-learning model for predicting oliguria in critically ill patients. *Sci Rep*. 2024;14(1):1054. doi:10.1038/s41598-024-51476-y
1149. Yamga E, Mantena S, Rosen D, et al. Optimized Risk Score to Predict Mortality in Patients With Cardiogenic Shock in the Cardiac Intensive Care Unit. *J Am Heart Assoc*. 2023;12(13):e029232. doi:10.1161/jaha.122.029232
1150. Yan P, Huang S, Li Y, et al. A New Risk Model Based on the Machine Learning Approach for Prediction of Mortality in the Respiratory Intensive Care Unit. *Curr Pharm Biotechnol*. 2023;24(13):1673-1681. doi:10.2174/1389201024666230220103755
1151. Yan C, Gao C, Zhang Z, et al. Predicting brain function status changes in critically ill patients via Machine learning. *J Am Med Inform Assoc*. 2021;28(11):2412-2422. doi:10.1093/jamia/ocab166
1152. Yan F, Chen X, Quan X, Wang L, Wei X, Zhu J. Association between the stress hyperglycemia ratio and 28-day all-cause mortality in critically ill patients with sepsis: a retrospective cohort study and predictive model establishment based on machine learning. *Cardiovasc Diabetol*. 2024;23(1)doi:10.1186/s12933-024-02265-4
1153. Yang M, Liu C, Wang X, et al. An Explainable Artificial Intelligence Predictor for Early Detection of Sepsis. *Crit Care Med*. 2020;48(11):e1091-e1096. doi:10.1097/ccm.0000000000004550
1154. Yang F, Peng C, Peng L, Wang J, Li Y, Li W. A Machine Learning Approach for the Prediction of Traumatic Brain Injury Induced Coagulopathy. *Front Med (Lausanne)*. 2021;8:792689. doi:10.3389/fmed.2021.792689
1155. Yang R, Huang T, Wang Z, et al. Deep-Learning-Based Survival Prediction of Patients in Coronary Care Units. *Comput Math Methods Med*. 2021;2021:5745304. doi:10.1155/2021/5745304
1156. Yang S, Sohn J, Lee S, Lee J, Kim HC. Estimation and Validation of Arterial Blood Pressure Using Photoplethysmogram Morphology Features in Conjunction With Pulse Arrival Time in Large Open Databases. *IEEE j biomed health inform*. 2021;25(4):1018-1030. doi:10.1109/jbhi.2020.3009658
1157. Yang H, Kuang L, Xia F. Multimodal temporal-clinical note network for mortality prediction. *J Biomed Semantics*. 2021;12(1):3. doi:10.1186/s13326-021-00235-3
1158. Yang B, Zhu Y, Lu X, Shen C. A Novel Composite Indicator of Predicting Mortality Risk for Heart Failure Patients With Diabetes Admitted to Intensive Care Unit Based on Machine Learning. *Front Endocrinol (Lausanne)*. 2022;13:917838. doi:10.3389/fendo.2022.917838
1159. Yang H, Li J, Liu S, Zhang M, Liu J. An interpretable DIC risk prediction model based on convolutional neural networks with time series data. *BMC Bioinformatics*. 2022;23(1):471. doi:10.1186/s12859-022-05004-2
1160. Yang F, Zhang J, Chen W, Lai Y, Wang Y, Zou Q. DeepMPM: a mortality risk prediction model using longitudinal EHR data. *BMC Bioinformatics*. 2022;23(1):423. doi:10.1186/s12859-022-04975-6
1161. Yang J, Lim HG, Park W, et al. Development of a machine learning model for the prediction of the short-term mortality in patients in the intensive care unit. *J Crit Care*. 2022;71:154106. doi:10.1016/j.jcrc.2022.154106
1162. Yang TM, Chen L, Lin CM, et al. Identifying Novel Clusters of Patients With Prolonged Mechanical Ventilation Using Trajectories of Rapid Shallow Breathing Index. *Front Med (Lausanne)*. 2022;9:880896. doi:10.3389/fmed.2022.880896
1163. Yang S, Cao L, Zhou Y, Hu C. A Retrospective Cohort Study: Predicting 90-Day Mortality for ICU Trauma Patients with a Machine Learning Algorithm Using XGBoost Using MIMIC-III Database. *J Multidiscip Healthc*. 2023;16:2625-2640. doi:10.2147/jmdh.S416943
1164. Yang J, Soltan AAS, Eyre DW, Clifton DA. Algorithmic fairness and bias mitigation for clinical machine learning with deep reinforcement learning. *Nat Mach Intell*. 2023;5(8):884-894. doi:10.1038/s42256-023-00697-3
1165. Yang J, Peng H, Luo Y, Zhu T, Xie L. Explainable ensemble machine learning model for prediction of 28-day mortality risk in patients with sepsis-associated acute kidney injury. *Front Med (Lausanne)*. 2023;10:1165129. doi:10.3389/fmed.2023.1165129

1166. Yang L, Li Z, Dai M, et al. Optimal machine learning methods for prediction of high-flow nasal cannula outcomes using image features from electrical impedance tomography. *Comput Methods Programs Biomed*. 2023;238doi:10.1016/j.cmpb.2023.107613
1167. Gaudin M, Kaur S, Sharma P, Kumar R. A Comparative Analysis of Machine Learning Models for the Classification of Heart Failure Patients in the Intensive Care Unit. *Recent Adv Electr Electron Eng*. 2024;doi:10.2174/0123520965312805240506113451
1168. Yang M, Liu S, Hao T, et al. Development and validation of a deep interpretable network for continuous acute kidney injury prediction in critically ill patients. *Artif Intell Med*. 2024;149doi:10.1016/j.artmed.2024.102785
1169. Yang M, Chen H, Hu W, et al. Development and Validation of an Interpretable Conformal Predictor to Predict Sepsis Mortality Risk: Retrospective Cohort Study. *J Med Internet Res*. 2024;26:e50369. doi:10.2196/50369
1170. Yao RQ, Jin X, Wang GW, et al. A Machine Learning-Based Prediction of Hospital Mortality in Patients With Postoperative Sepsis. *Front Med (Lausanne)*. 2020;7:445. doi:10.3389/fmed.2020.00445
1171. Ye G, Balasubramanian V, Li JK, Kaya M. Machine Learning-Based Continuous Intracranial Pressure Prediction for Traumatic Injury Patients. *IEEE J Transl Eng Health Med*. 2022;10:4901008. doi:10.1109/jtehm.2022.3179874
1172. Ye J, Yao L, Shen J, Janarthanam R, Luo Y. Predicting mortality in critically ill patients with diabetes using machine learning and clinical notes. *BMC Med Inf Decis Mak*. 2020;20:295. doi:10.1186/s12911-020-01318-4
1173. Ye Z, An S, Gao Y, et al. The prediction of in-hospital mortality in chronic kidney disease patients with coronary artery disease using machine learning models. *Eur J Med Res*. 2023;28(1):33. doi:10.1186/s40001-023-00995-x
1174. Ye RZ, Lipatov K, Diedrich D, et al. Automatic ARDS surveillance with chest X-ray recognition using convolutional neural networks. *J Crit Care*. 2024;82:154794. doi:10.1016/j.jcrc.2024.154794
1175. Yeh P, Pan Y, Sanchez-Pinto LN, Luo Y. Using Machine Learning to Predict Hyperchloremia in Critically Ill Patients. *Proceedings (IEEE Int Conf Bioinformatics Biomed)*. 2019;2019:1703-1707. doi:10.1109/bibm47256.2019.8982933
1176. Yeh P, Pan Y, Sanchez-Pinto LN, Luo Y. Hyperchloremia in critically ill patients: association with outcomes and prediction using electronic health record data. *BMC Med Inf Decis Mak*. 2020;20:302. doi:10.1186/s12911-020-01326-4
1177. Yen CT, Liao JX, Huang YK. Applying a Deep Learning Network in Continuous Physiological Parameter Estimation Based on Photoplethysmography Sensor Signals. *IEEE Sens J*. 2022;22(1):385-392. doi:10.1109/jsen.2021.3126744
1178. Yijing L, Wenyu Y, Kang Y, et al. Prediction of cardiac arrest in critically ill patients based on bedside vital signs monitoring. *Comput Methods Programs Biomed*. 2022;214:106568. doi:10.1016/j.cmpb.2021.106568
1179. Yildirim S, Sunecli O, Kirakli C. Mortality Prediction with Machine Learning in COVID-19 Patients in Intensive Care Units: A Retrospective and Prospective Longitudinal Study. *J Cri Int Care*. 2024;15(1):30-36. doi:10.14744/dcybd.2023.3691
1180. Yimer MA, Aktas Ö, Sevinç S, Sisman AR. Establishing Causee-Effect Relationships from Medical Treatment Data in Intensive Care Unit Settings. *Int Arab J Inf Technol*. 2023;20(5):693-701. doi:10.34028/iajit/20/5/1
1181. Yin Y, Chou CA. Multi-event survival analysis through dynamic multi-modal learning for ICU mortality prediction. *Comput Methods Programs Biomed*. 2023;235:107545. doi:10.1016/j.cmpb.2023.107545
1182. Yong L, Zhenzhou L. Deep learning-based prediction of in-hospital mortality for sepsis. *Sci Rep*. 2024;14(1):372. doi:10.1038/s41598-023-49890-9
1183. Yoon JH, Jeanselme V, Dubrawski A, Hravnak M, Pinsky MR, Clermont G. Prediction of hypotension events with physiologic vital sign signatures in the intensive care unit. *Crit Care*. 2020;24(1):661. doi:10.1186/s13054-020-03379-3
1184. Yoon D, Han C, Kim DW, et al. Redefining Health Care Data Interoperability: Empirical Exploration of Large Language Models in Information Exchange. *J Med Internet Res*. 2024;26:e56614. doi:10.2196/56614
1185. Young M, Holmes N, Robbins R, et al. Natural language processing to assess the epidemiology of delirium-suggestive behavioural disturbances in critically ill patients. *Crit Care Resusc*. 2021;23(2):144-153. doi:
1186. Young M, Holmes N, Kishore K, et al. Natural language processing diagnosed behavioral disturbance vs confusion assessment method for the intensive care unit: prevalence, patient

characteristics, overlap, and association with treatment and outcome. *Intensive Care Med*. 2022;48(5):559-569. doi:10.1007/s00134-022-06650-z
1187. Young M, Holmes NE, Kishore K, et al. Natural language processing diagnosed behavioural disturbance phenotypes in the intensive care unit: characteristics, prevalence, trajectory, treatment, and outcomes. *Crit Care*. 2023;27(1):425. doi:10.1186/s13054-023-04695-0
1188. Yu L, Li L, Bernstam E, Jiang X. A deep learning solution to recommend laboratory reduction strategies in ICU. *Int J Med Inf*. 2020;144:104282. doi:10.1016/j.ijmedinf.2020.104282
1189. Yu Q, Wang C, Xi J, et al. Intensive Care Unit False Alarm Identification Based on Convolution Neural Network. *IEEE Access*. 2021;9:81841-81854. doi:10.1109/access.2021.3086862
1190. Yu R, Wang S, Xu J, et al. Machine Learning Approaches-Driven for Mortality Prediction for Patients Undergoing Craniotomy in ICU. *Brain Inj*. 2021;35(14):1658-1664. doi:10.1080/02699052.2021.2008491
1191. Yu Y, Peng C, Zhang Z, et al. Machine Learning Methods for Predicting Long-Term Mortality in Patients After Cardiac Surgery. *Front cardiovasc med*. 2022;9:831390. doi:10.3389/fcvm.2022.831390
1192. Yu DX, Xiao J. Multilevel Information Granule Construction Model Based on Large Data Environment and Its Application in Time Series. *Mob Inf Syst*. 2022;2022doi:10.1155/2022/9465551
1193. Yu R, Kong X, Li Y. Optimizing the Diagnostic Algorithm for Pulmonary Embolism in Acute COPD Exacerbation Using Fuzzy Rough Sets and Support Vector Machine. *COPD J Chronic Obstructive Pulm Dis*. 2023;20(1):1-8. doi:10.1080/15412555.2022.2139671
1194. Yu Y, Rao J, Xu Q, et al. Phenotyping cardiogenic shock that showed different clinical outcomes and responses to vasopressor use: a latent profile analysis from MIMIC-IV database. *Front Med*. 2023;10doi:10.3389/fmed.2023.1186119
1195. Yu C, Huang Q. Towards more efficient and robust evaluation of sepsis treatment with deep reinforcement learning. *BMC Med Inf Decis Mak*. 2023;23(1):43. doi:10.1186/s12911-023-02126-2
1196. Yu J, Tasken AA, Flade HM, et al. Automatic assessment of left ventricular function for hemodynamic monitoring using artificial intelligence and transesophageal echocardiography. *J Clin Monit Comput*. Apr 2024;38(2):281-291. doi:10.1007/s10877-023-01118-x [pii] 10.1007/s10877-023-01118-x
1197. Yu M, Wang S, He K, et al. Predicting the complexity and mortality of polytrauma patients with machine learning models. *Sci Rep*. 2024;14(1):8302. doi:10.1038/s41598-024-58830-0
1198. Yuan KC, Tsai LW, Lai KS, Teng ST, Lo YS, Peng SJ. Using Transfer Learning Method to Develop an Artificial Intelligence Assisted Triaging for Endotracheal Tube Position on Chest X-ray. *Diagnostics (Basel)*. 2021;11(10)doi:10.3390/diagnostics11101844
1199. Yue S, Li S, Huang X, et al. Machine learning for the prediction of acute kidney injury in patients with sepsis. *J Transl Med*. 2022;20(1):215. doi:10.1186/s12967-022-03364-0
1200. Yun K, Oh J, Hong TH, Kim EY. Prediction of Mortality in Surgical Intensive Care Unit Patients Using Machine Learning Algorithms. *Front Med (Lausanne)*. 2021;8:621861. doi:10.3389/fmed.2021.621861
1201. Yun J, Ahn Y, Cho K, et al. Deep Learning for Automated Triaging of Stable Chest Radiographs in a Follow-up Setting. *Radiology*. 2023;309(1)doi:10.1148/radiol.230606
1202. Zabihi S, Rahimian E, Marefat F, Asif A, Mohseni P, Mohammadi A. BP-Net: Cuff-less and non-invasive blood pressure estimation via a generic deep convolutional architecture. *Biomed Signal Process Control*. 2022;78doi:10.1016/j.bspc.2022.103850
1203. Zahr RS, Mohammed A, Naik S, et al. Machine Learning Predicts Acute Kidney Injury in Hospitalized Patients with Sickle Cell Disease. *Am J Nephrol*. 2024;55(1):18-24. doi:10.1159/000534864
1204. Zappala S, Alfieri F, Ancona A, et al. Development and external validation of a machine learning model for the prediction of persistent acute kidney injury stage 3 in multi-centric, multi-national intensive care cohorts. *Crit Care*. 2024;28(1):189. doi:10.1186/s13054-024-04954-8
1205. Zardkoohi M, Fatemeh Molaeezadeh S. Long-term prediction of blood pressure time series using ANFIS system based on DKFCM clustering. *Biomed Signal Process Control*. 2022;74doi:10.1016/j.bspc.2022.103480
1206. Zarrin D, Suri A, McCarthy K, et al. Machine Learning Predicts Cerebral Vasospasm in Subarachnoid Hemorrhage Patients. *Res Sq*. Feb 5 2024;doi:rs.3.rs-3617246 [pii] 10.21203/rs.3.rs-3617246/v1
1207. Zeng Z, Yao S, Zheng J, Gong X. Development and validation of a novel blending machine learning model for hospital mortality prediction in ICU patients with Sepsis. *BioData Min*. 2021;14(1):40. doi:10.1186/s13040-021-00276-5

1208. Zeng Z, Tang X, Liu Y, He Z, Gong X. Interpretable recurrent neural network models for dynamic prediction of the extubation failure risk in patients with invasive mechanical ventilation in the intensive care unit. *BioData Min*. 2022;15(1):21. doi:10.1186/s13040-022-00309-7
1209. Zeng Z, Liu Y, Yao S, et al. Neural networks based on attention architecture are robust to data missingness for early predicting hospital mortality in intensive care unit patients. *Digit Health*. 2023;9:20552076231171482. doi:10.1177/20552076231171482
1210. Zeng GJ, Zhuang JH, Huang HF, et al. Use of Deep Learning for Continuous Prediction of Mortality for All Admissions in Intensive Care Units. *Tsinghua Sci Technol*. 2023;28(4):639-648. doi:10.26599/tst.2022.9010027
1211. Zeng J, Zhang M, Du J, et al. Mortality prediction and influencing factors for intensive care unit patients with acute tubular necrosis: random survival forest and cox regression analysis. *Front Pharmacol*. 2024;15:1361923. doi:10.3389/fphar.2024.1361923
1212. Zhai S, Wang H, Sun L, et al. Artificial intelligence (AI) versus expert: A comparison of left ventricular outflow tract velocity time integral (LVOT-VTI) assessment between ICU doctors and an AI tool. *J Appl Clin Med Phys*. 2022;23(8):e13724. doi:10.1002/acm2.13724
1213. Zhang S, Wu ZS, Chang W, et al. Classification of Patients With Sepsis According to Immune Cell Characteristics: A Bioinformatic Analysis of Two Cohort Studies. *Front Med*. 2020;7doi:10.3389/fmed.2020.598652
1214. Zhang D, Yin C, Zeng J, Yuan X, Zhang P. Combining structured and unstructured data for predictive models: a deep learning approach. *BMC Med Inf Decis Mak*. 2020;20(1):280. doi:10.1186/s12911-020-01297-6
1215. Zhang G, Yuan J, Yu M, Wu T, Luo X, Chen F. A machine learning method for acute hypotensive episodes prediction using only non-invasive parameters. *Comput Methods Programs Biomed*. 2021;200:105845. doi:10.1016/j.cmpb.2020.105845
1216. Zhang Z, Liu J, Xi J, Gong Y, Zeng L, Ma P. Derivation and Validation of an Ensemble Model for the Prediction of Agitation in Mechanically Ventilated Patients Maintained Under Light Sedation. *Crit Care Med*. 2021;49(3):e279-e290. doi:10.1097/ccm.0000000000004821
1217. Zhang K, Wang H, Du J, et al. An interpretable RL framework for pre-deployment modeling in ICU hypotension management. *NPJ Digit Med*. 2022;5(1)doi:10.1038/s41746-022-00708-4
1218. Zhang J, Bolanos Trujillo LD, Tanwar A, Ive J, Gupta V, Guo Y. Clinical utility of automatic phenotype annotation in unstructured clinical notes: intensive care unit use. *BMJ Health Care Inform*. 2022;29(1)doi:10.1136/bmjhci-2021-100519
1219. Zhang L, Wang Z, Zhou Z, et al. Developing an ensemble machine learning model for early prediction of sepsis-associated acute kidney injury. *iScience*. 2022;25(9):104932. doi:10.1016/j.isci.2022.104932
1220. Zhang X, Chen S, Lai K, Chen Z, Wan J, Xu Y. Machine learning for the prediction of acute kidney injury in critical care patients with acute cerebrovascular disease. *Ren Fail*. 2022;44(1):43-53. doi:10.1080/0886022x.2022.2036619
1221. Zhang X, Fei N, Wang Q, Fang Z. Machine Learning Prediction Models for Postoperative Stroke in Elderly Patients: Analyses of the MIMIC Database. *Front Aging Neurosci*. 2022;14:897611. doi:10.3389/fnagi.2022.897611
1222. Zhang H, Wang Z, Tang Y, et al. Prediction of acute kidney injury after cardiac surgery: model development using a Chinese electronic health record dataset. *J Transl Med*. 2022;20(1)doi:10.1186/s12967-022-03351-5
1223. Zhang L, Huang T, Xu F, et al. Prediction of prognosis in elderly patients with sepsis based on machine learning (random survival forest). *BMC Emerg Med*. 2022;22(1):26. doi:10.1186/s12873-022-00582-z
1224. Zhang Q, Wang J, Liu G, Zhang W. Artificial intelligence can use physiological parameters to optimize treatment strategies and predict clinical deterioration of sepsis in ICU. *Physiol Meas*. 2023;44(1)doi:10.1088/1361-6579/acb03b
1225. Zhang Y, Hu J, Hua T, Zhang J, Zhang Z, Yang M. Development of a machine learning-based prediction model for sepsis-associated delirium in the intensive care unit. *Sci Rep*. 2023;13(1):12697. doi:10.1038/s41598-023-38650-4
1226. Zhang SW, Duan YR, Hou FG, et al. Early prediction of sepsis using a high-order Markov dynamic Bayesian network (HMDBN) classifier. *Appl Intell*. 2023;53(22):26384-26399. doi:10.1007/s10489-023-04920-x
1227. Zhang Z, Wang SJ, Chen K, Yin AA, Lin W, He YL. Machine learning algorithms for improved prediction of in-hospital outcomes after moderate-to-severe traumatic brain injury: a Chinese retrospective cohort study. *Acta Neurochir (Wien)*. 2023;doi:10.1007/s00701-023-05647-x

1228. Zhang F, Wang H, Liu L, Su T, Ji B. Machine learning model for the prediction of gram-positive and gram-negative bacterial bloodstream infection based on routine laboratory parameters. *BMC Infect Dis*. 2023;23(1):675. doi:10.1186/s12879-023-08602-4
1229. Zhang Z, Wang J, Han W, Zhao L. Using machine learning methods to predict 28-day mortality in patients with hepatic encephalopathy. *BMC Gastroenterol*. 2023;23(1):111. doi:10.1186/s12876-023-02753-z
1230. Zhang Q, Li TH, Li DF, Lu W. A goal-oriented reinforcement learning for optimal drug dosage control. *Ann Oper Res*. 2024;doi:10.1007/s10479-024-06029-x
1231. Zhang N, Xie K, Yang F, Wang Y, Yang X, Zhao L. Combining biomarkers of BNIP3 L, S100B, NSE, and accessible measures to predict sepsis-associated encephalopathy: a prospective observational study. *Curr Med Res Opin*. 2024;40(4):575-582. doi:10.1080/03007995.2024.2322059
1232. Zhang M, Kuo TT. Early prediction of long hospital stay for Intensive Care units readmission patients using medication information. *Comput Biol Med*. 2024;174:108451. doi:10.1016/j.compbiomed.2024.108451
1233. Zhang J, Liu W, Xiao W, Liu Y, Hua T, Yang M. Machine learning-derived blood culture classification with both predictive and prognostic values in the intensive care unit: A retrospective cohort study. *Intensive Crit Care Nurs*. 2024;80:103549. doi:10.1016/j.iccn.2023.103549
1234. Zhang T, Qu Y, wang D, Zhong M, Cheng Y, Zhang M. Optimizing sepsis treatment strategies via a reinforcement learning model. *Biomed Eng Lett*. 2024;14(2):279-289. doi:10.1007/s13534-023-00343-2
1235. Zhang G, Shao F, Yuan W, et al. Predicting sepsis in-hospital mortality with machine learning: a multi-center study using clinical and inflammatory biomarkers. *Eur J Med Res*. 2024;29(1):156. doi:10.1186/s40001-024-01756-0
1236. Zhao QY, Liu LP, Luo JC, et al. A Machine-Learning Approach for Dynamic Prediction of Sepsis-Induced Coagulopathy in Critically Ill Patients With Sepsis. *Front Med (Lausanne)*. 2020;7:637434. doi:10.3389/fmed.2020.637434
1237. Zhao QY, Wang H, Luo JC, et al. Development and Validation of a Machine-Learning Model for Prediction of Extubation Failure in Intensive Care Units. *Front Med (Lausanne)*. 2021;8:676343. doi:10.3389/fmed.2021.676343
1238. Zhao X, Gu B, Li Q, et al. Machine learning approach identified clusters for patients with low cardiac output syndrome and outcomes after cardiac surgery. *Front cardiovasc med*. 2022;9:962992. doi:10.3389/fcvm.2022.962992
1239. Zhao X, Lu Y, Li S, et al. Predicting renal function recovery and short-term reversibility among acute kidney injury patients in the ICU: comparison of machine learning methods and conventional regression. *Ren Fail*. 2022;44(1):1326-1337. doi:10.1080/0886022x.2022.2107542
1240. Zhao X, Nie X, Pang G, et al. Prior Distribution Estimation of Monitored Information in the Intensive Care Unit with the Hidden Markov Model and Decision Tree Methods. *J Healthc Eng*. 2022;2022doi:10.1155/2022/7892408
1241. Zhao B, Huepenbecker S, Zhu G, Rajan SS, Fujimoto K, Luo X. Comorbidity network analysis using graphical models for electronic health records. *Front big data*. 2023;6:846202. doi:10.3389/fdata.2023.846202
1242. Zhao JO, Patel BK, Krishack P, et al. Identification of Clinically Significant Cytokine Signature Clusters in Patients With Septic Shock. *Crit Care Med*. 2023;51(12):e253-e263. doi:10.1097/ccm.0000000000006032
1243. Zhao J, Rong C, Lin C, Dang X. Multivariate time series data imputation using attention-based mechanism. *Neurocomputing*. 2023;542doi:10.1016/j.neucom.2023.126238
1244. Zhao Y, Chen C, Huang Z, et al. Prediction of upcoming urinary tract infection after intracerebral hemorrhage: a machine learning approach based on statistics collected at multiple time points. *Front Neurol*. 2023;14:1223680. doi:10.3389/fneur.2023.1223680
1245. Zhao YS, Lai QP, Tang H, et al. Identifying the risk factors of ICU-acquired fungal infections: clinical evidence from using machine learning. *Front Med (Lausanne)*. 2024;11:1386161. doi:10.3389/fmed.2024.1386161
1246. Zheng H, Zhu JH, Xie W, Zhong J. Reinforcement learning assisted oxygen therapy for COVID-19 patients under intensive care. *BMC Med Inform Decis Mak*. 2021;21(1)doi:10.1186/s12911-021-01712-6
1247. Zheng L, Lin Y, Fang K, Wu J, Zheng M. Derivation and validation of a risk score to predict acute kidney injury in critically ill cirrhotic patients. *Hepatol Res*. 2023;doi:10.1111/hepr.13907
1248. Zheng F, Wang L, Pang Y, et al. ShockSurv: A machine learning model to accurately predict 28-day mortality for septic shock patients in the intensive care unit. *Biomed Signal Process Control*. 2023;86doi:10.1016/j.bspc.2023.105146

1249. Zhi D, Zhang M, Lin J, Liu P, Wang Y, Duan M. Establishment and validation of the predictive model for the in-hospital death in patients with sepsis. *Am J Infect Control*. 2021;49(12):1515-1521. doi:10.1016/j.ajic.2021.07.010
1250. Zhong Z, Yuan X, Liu S, Yang Y, Liu F. Machine learning prediction models for prognosis of critically ill patients after open-heart surgery. *Sci Rep*. 2021;11(1):3384. doi:10.1038/s41598-021-83020-7
1251. Zhou Y, Zhao G, Li J, et al. A contrastive learning approach for ICU false arrhythmia alarm reduction. *Sci Rep*. 2022;12(1):4689. doi:10.1038/s41598-022-07761-9
1252. Zhou Y, Feng J, Mei S, et al. Machine Learning Models for Predicting Acute Kidney Injury in Patients with Sepsis-Associated Acute Respiratory Distress Syndrome. *Shock*. 2023;59(3):352-359. doi:10.1097/shk.0000000000002065
1253. Zhou L, Romero-García N, Badenes R, et al. Machine learning uncovers blood test patterns subphenotypes at hospital admission discerning increased 30-day ICU mortality rates in COVID-19 elderly patients. *medRxiv*. 2022;doi:10.1101/2022.05.10.22274889
1254. Zhou K, Yin ZX, Peng Y, Zeng ZL. Methods for Continuous Blood Pressure Estimation Using Temporal Convolutional Neural Networks and Ensemble Empirical Mode Decomposition. *Electronics*. 2022;11(9)doi:10.3390/electronics11091378
1255. Zhou X, Li X, Zhang Z, et al. Support vector machine deep mining of electronic medical records to predict the prognosis of severe acute myocardial infarction. *Front Physiol*. 2022;13:991990. doi:10.3389/fphys.2022.991990
1256. Zhou Y, Feng J, Mei S, et al. A deep learning model for predicting COVID-19 ARDS in critically ill patients. *Front Med (Lausanne)*. 2023;10:1221711. doi:10.3389/fmed.2023.1221711
1257. Zhou H, Liu L, Zhao Q, et al. Machine learning for the prediction of all-cause mortality in patients with sepsis-associated acute kidney injury during hospitalization. *Front Immunol*. 2023;14:1140755. doi:10.3389/fimmu.2023.1140755
1258. Zhou S, Lu Z, Liu Y, et al. Interpretable machine learning model for early prediction of 28-day mortality in ICU patients with sepsis-induced coagulopathy: development and validation. *Eur J Med Res*. 2024;29(1):14. doi:10.1186/s40001-023-01593-7
1259. Zhu Y, Fan X, Wu J, Liu X, Shi J, Wang C. Predicting ICU mortality by supervised bidirectional LSTM networks. 2018:
1260. Zhu SH, Pu J. A self-supervised method for treatment recommendation in sepsis. *Front Inform Technol Elect Eng*. 2021;22(7):926-939. doi:10.1631/fitee.2000127
1261. Zhu Y, Zhang J, Wang G, et al. Machine Learning Prediction Models for Mechanically Ventilated Patients: Analyses of the MIMIC-III Database. *Front Med (Lausanne)*. 2021;8:662340. doi:10.3389/fmed.2021.662340
1262. Zhu J, Shan Y, Li Y, Wu X, Gao G. Predicting the Severity and Discharge Prognosis of Traumatic Brain Injury Based on Intracranial Pressure Data Using Machine Learning Algorithms. *World Neurosurg*. 2024;185:e1348-e1360. doi:10.1016/j.wneu.2024.03.085
1263. Zhuang J, Huang H, Jiang S, Liang J, Liu Y, Yu X. A generalizable and interpretable model for mortality risk stratification of sepsis patients in intensive care unit. *BMC Med Inf Decis Mak*. 2023;23(1):185. doi:10.1186/s12911-023-02279-0
1264. Zou M, An Y, Kuang H, Wang J. LGTRL-DE: Local and Global Temporal Representation Learning with Demographic Embedding for in-hospital mortality prediction. *J Biomed Inform*. 2023;143:104408. doi:10.1016/j.jbi.2023.104408
1265. Zou H, Yang W, Wang M, et al. Predicting length of stay ranges by using novel deep neural networks. *Heliyon*. 2023;9(2):e13573. doi:10.1016/j.heliyon.2023.e13573
1266. Zou B, Ding Y, Li J, Yu B, Kui X. TGRA-P: Task-driven model predicts 90-day mortality from ICU clinical notes on mechanical ventilation. *Comput Methods Programs Biomed*. 2023;242doi:10.1016/j.cmpb.2023.107783
1267. Zwerwer LR, Luz CF, Soudis D, et al. Identifying the need for infection-related consultations in intensive care patients using machine learning models. *Sci Rep*. 2024;14(1):2317. doi:10.1038/s41598-024-52741-w
