# Supplementary material for: Operationalization of Artificial Intelligence Applications in the Intensive Care Unit: A Systematic Review
Source: JAMA Netw Open. 2025 Jul 23;8(7):e2522866. doi: 10.1001/jamanetworkopen.2025.22866 (PMC12287835; doi:10.1001/jamanetworkopen.2025.22866)
Supplement: Supplement 2. — Data Sharing Statement [file jamanetwopen-e2522866-s002.pdf]

Data Sharing Statement

Berkhout. Operationalization of Artificial Intelligence Applications in the Intensive Care Unit. *JAMA Netw Open*. Published July 23, 2025. doi:10.1001/jamanetworkopen.2025.22866

Data
Data available: Yes
Data types: Data (not involving human participants)
How to access data: Provided in the Supplementary Online Content, eAppendix 5.
When available: With publication

Supporting Documents
Document types: None

Additional Information
Who can access the data: N/A
Types of analyses: N/A
Mechanisms of data availability: N/A
